# ALFAssay: A feed‑forward neural network for quantitative fragmentomics‑based ctDNA profiling in breast cancer

**DOI:** 10.1371/journal.pcbi.1014505

**Published:** 2026-07-27

**Authors:** Alexandra Stanciu, Andrea Gombos, Elisa Agostinetto, Delphine Vincent, Laurence Buisseret, Andreas Papagiannis, Francoise Rothe, Nicola Occelli, Christos Sotiriou, David Venet, Michail Ignatiadis

**Affiliations:** Institut Jules Bordet and l’Université Libre de Bruxelles (U.L.B.), Brussels, Belgium; University of Helsinki: Helsingin Yliopisto, FINLAND

## Abstract

The analysis of cell‑free DNA (cfDNA) is transforming cancer diagnostics, yet quantifying the fraction of circulating tumour DNA (ctDNA) from shallow whole‑genome sequencing (sWGS) remains challenging in tumours with low copy‑number aberration burden. We introduce **ALFAssay**, a feed‑forward neural network that estimates ctDNA fraction from fragmentation profiles of cfDNA in breast cancer. Using cfDNA from 896 plasma samples spanning early and metastatic HR + /HER2– and triple‑negative breast cancer, and healthy controls, (id: NCT03616886, NCT02028364, NCT03065621) we extract 204 bin‑level fragmentation features by computing the ratio of short fragments (90–150 bp) to all reads within 5 Mb genomic windows, while accounting for coverage effects by incorporating the total number of reads for each bin as an additional input feature. The resulting 408‑dimensional vectors are input to a fully connected network trained against ichorCNA‑derived ctDNA fractions using five‑fold cross‑validation. ALFAssay demonstrates high sensitivity (0.87) and specificity (0.94) for ctDNA detection and correlates with ichorCNA (r = 0.89) and the fragmentation‑based tool Fragle (r = 0.81). Its ctDNA predictions stratify patients by progression‑free survival and add complementary prognostic value to available tools. ALFAssay thus expands the bioinformatics toolkit for ctDNA quantification and highlights the potential of fragmentation signatures to augment multi‑modal liquid‑biopsy workflows.

**Key Points**

- ALFAssay is a deep-learning–based method for estimating ctDNA fraction from cfDNA fragmentation profiles using shallow whole-genome sequencing data.
- ALFAssay introduces a pipeline combining GC-corrected fragmentomic preprocessing, and a feed-forward neural network to quantifiy ctDNA fraction in women with breast cancer.
- Using cfDNA data from 896 plasma samples across multiple breast cancer cohorts, we demonstrate that ALFAssay robustly quantifies ctDNA.
- Benchmarking against ichorCNA, Fragle, and mutation-based assays suggests that ALFAssay could provide fragmentation-based information alongside copy-number– and mutation-based ctDNA estimation approaches.

## Introduction

The analysis of circulating tumor DNA (ctDNA) has revolutionized non-invasive cancer monitoring, providing critical insights into tumor burden, therapeutic response, and disease progression [1–3]. However, quantification of ctDNA fraction within the pool of cell-free DNA (cfDNA) remains technically challenging, especially in cases where the tumor burden is low or genomic alterations are minimal [4,5]. Traditional methods such as targeted deep sequencing for mutation tracking and shallow whole-genome sequencing (sWGS) for copy number aberration (CNA) analysis have been widely adopted to infer ctDNA presence [6,7]. Tools like ichorCNA have demonstrated effectiveness in detecting ctDNA through CNA profiling across various cancer types [8]. Yet, their sensitivity diminishes in tumors with low CNA burden, a limitation particularly evident in hormone receptor-positive/HER2-negative (HR + /HER2−) breast cancer, which typically exhibits fewer genomic aberrations compared to more aggressive subtypes like triple-negative breast cancer [9,10].

To address these limitations, recent research has turned toward alternative cfDNA features—most notably fragmentation patterns — as complementary signals to copy-number–based approaches. Accumulating evidence indicates that cfDNA fragments derived from tumor and non-tumor cells exhibit distinct characteristics, with cancer patients displaying altered fragment size distributions and non-random cleavage profiles [11–13]. These fragmentation signatures are shaped by differences in chromatin organization, nucleosome positioning, and degradation dynamics between tumor and healthy cells [14,15]. Deep learning models have been developed to quantify tumor fraction from such fragmentomic profiles [16–19]. For example, Fragle uses fragment size distribution to estimate tumor fraction from cfDNA using a convolutional neural network. Similarly, iLLMAC, an instruction-tuned large language model, has been developed to detect cancer based on cfDNA end-motif profiles.

In this study, we introduce ALFAssay, a deep learning model tailored specifically for breast cancer, which estimates tumor fraction using cfDNA fragmentation patterns. Trained on datasets where tumor fractions were estimated using ichorCNA, ALFAssay learns to extract meaningful fragmentation-based representations of ctDNA. Our model is designed to use fragmentation signals and it is optimized for application in breast cancer. Through comprehensive evaluation, we demonstrate that ALFAssay offers another approach to existing ctDNA quantification methods, expanding the toolkit for minimally invasive cancer diagnostics.

## Results

### Overview of a neural network model for predicting ctDNA fraction from cfDNA

**Fig 1:**
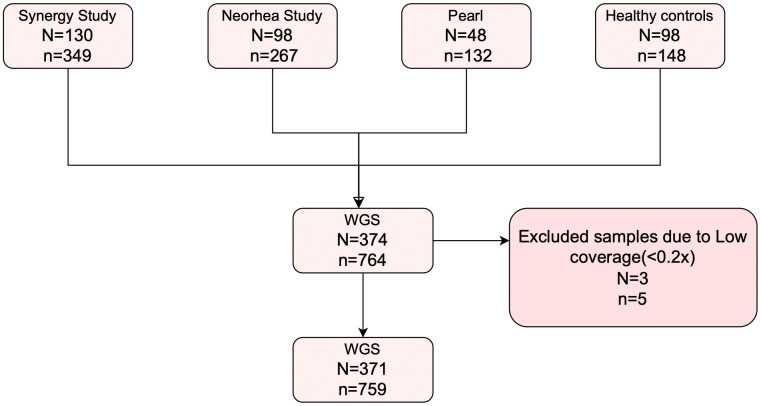
Flowchart diagram. Illustrates the studies included in the development of the ALFAssay model, along with the corresponding number of patients and samples analyzed. Three patients (representing five samples) were excluded due to low sequencing coverage (< 0.2×). A capital “N” denotes the number of patients, whereas a lowercase “n” denotes the number of samples.

**Fig 2:**
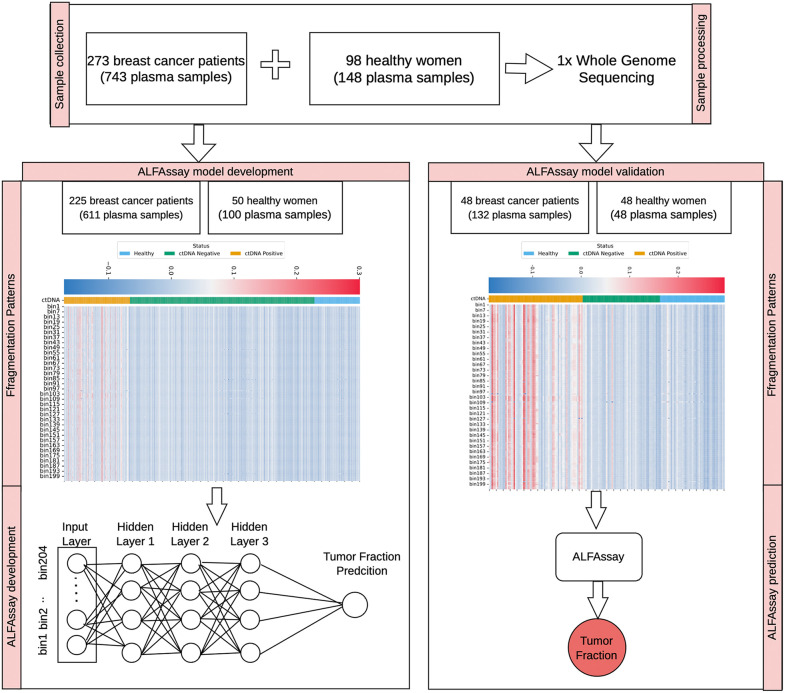
Overview of the ALFAssay pipeline for tumor fraction prediction based on cfDNA fragmentation patterns. Plasma samples from 273 breast cancer patients (743 samples) and 98 healthy women (148 samples) underwent shallow whole-genome sequencing (approx. 1 × sWGS). We derived fragmentation features across 204 genomic bins by incorporating both short-to-total fragment ratios and bin-level fragment counts as input features (see Methods)—and then used these features as input to a fully connected feed‐forward neural network. The ALFAssay model was trained and evaluated using a cross-validation framework on early (Neorhea cohort) and metastatic (Synergy cohort) breast cancer patient samples, and healthy women (healthy training) samples. Model performance was independently validated in samples from metastatic breast cancer patients (Pearl cohort) and healthy women. CtDNA negative vs positive sample was defined based on ichorCNA results.The model was trained using ichorCNA-derived tumor fractions as reference.

We developed ALFAssay, a feed-forward neural network designed to estimate tumor fraction directly from cfDNA fragmentation profiles derived from shallow whole-genome sequencing (sWGS). The pipeline was developed and evaluated on 896 plasma samples across three independent breast cancer cohorts and one healthy control cohort (Figs 1 and 2). Specifically, the dataset comprised the Neorhea cohort of early-stage HR + /HER2– breast cancer patients (98 individuals, 267 samples), the Synergy cohort of metastatic triple-negative breast cancer patients (127 individuals, 344 samples), the Pearl cohort of metastatic HR + /HER2– patients (48 individuals, 132 samples), and a healthy control cohort (98 individuals, 148 samples) [20,21]. This design allowed us to test ALFAssay across both early and metastatic settings, as well as across subtypes characterized by distinct genomic alteration landscapes.

To generate the input features, we partitioned the genome into 204 non-overlapping bins of 5 Mb each, retaining only high-confidence genomic regions after quality filtering. For each bin, we computed the ratio of short cfDNA fragments (90–150 bp) to all fragments (30–700 bp). These short-to-all ratios capture tumor-associated fragmentation patterns while partially controlling for overall cfDNA abundance. To account for coverage-related bias, we provided the total fragment count for each bin as an additional input to the neural network, enabling it to learn and correct for sequencing depth effects implicitly. Each plasma sample was thus represented by a 408-dimensional feature vector, consisting of 204 fragmentation ratios paired with their corresponding bin-level fragment counts. This step is part of data pre-processing and it is performed for each dataset separately.

These fragmentation profiles were used to train a fully connected feed-forward neural network (FFNN) configured for regression. The architecture consisted of four dense layers with ReLU activations and layer normalization, and a final linear output unit producing continuous tumor fraction estimates. Training was performed in a supervised manner, with ichorCNA-derived tumor fractions serving as the reference. Samples with ichorCNA tumor fraction <3% were assigned a value of zero, consistent with established confidence thresholds reported by ichorCNA. Model training was conducted using 5-fold cross-validation on the combined Neorhea and Synergy cohorts, together with a subset of healthy controls used exclusively for training, while the trained model was subsequently evaluated on the independent Pearl cohort and a separate external healthy control set.

### ALFAssay ctDNA fraction prediction across breast cancer subtypes and disease stages

**Fig 3:**
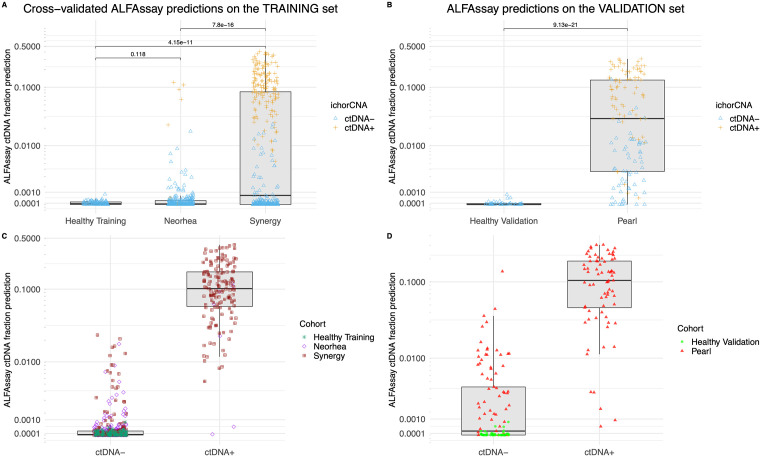
ALFAassay ctDNA fraction prediction in training(5-fold cross-validation) and validation data sets. A. Predicted ctDNA fractions from the training dataset, comprising healthy women, early breast cancer patients (Neorhea cohort) and metastatic breast cancer patients (Synergy cohort). Each datapoint is colored by ichorCNA detection status where the blue triangle is ctDNA negative and the orange cross is ctDNA positive (samples with ichorCNA tumor fraction >= 3% are considered ctDNA positive, negative otherwise) B. Predictions on an independent validation dataset (Pearl cohort) are similarly displayed, illustrating ctDNA fraction estimates across control and metastatic samples C. ALFAssay prediction on training dataset visualised based on ichorCNA detection positive/negative D. ALFAssay prediction on validation dataset visualised based on ichorCNA detection positive/negative. Each datapoint is colored by dataset. Box plots indicate medians and interquartile ranges; individual points correspond to per-sample ctDNA predictions generated by the ALFAssay model.

We evaluated the performance of ALFAssay in predicting ctDNA fractions. Fig 3 presents the model predictions on both training and validation datasets, demonstrating consistent performance across different clinical contexts.

In the training cohort (Fig 3A), ALFAssay distinguished between healthy controls and cancer patients, with healthy women showing consistently low predicted ctDNA fractions (median = 6.4*10–5, range = 0 - 0.0008). Early-stage breast cancer patients from the Neorhea cohort displayed low ctDNA fraction predictions (median= $\text{4}.\text{3}*{\text{1}\text{0}}^{-\text{5}}$, range = 0 - 0.012), while metastatic patients from the Synergy cohort exhibited the highest predicted fractions, reflecting the expected relationship between disease burden and circulating tumor DNA levels (median= $\text{7}.\text{2}*{\text{1}\text{0}}^{-\text{3}}$, range = 0 - 0.40). The model predictions show concordance with ichorCNA detection status, with ctDNA-positive samples clustering at higher predicted fractions compared to ctDNA-negative samples.

Independent validation on the Pearl cohort (Fig 3B) confirmed the generalizability of the model, showing discrimination patterns between healthy controls (median = 0, range = 0 - $\text{8}*{\text{1}\text{0}}^{-\text{4}}$) and metastatic HR + /HER2- breast cancer patients (median = 0.02, range = 0 - 0.30). The validation results maintained the separation between ctDNA-positive and ctDNA-negative samples, with ctDNA-positive cases consistently showing elevated predicted fractions.

When stratified by ichorCNA detection status (Fig 3C-3D), ctDNA-positive samples being defined as those with tumor fraction estimated by ichorCNA greater or equal than 0.03, ALFAssay demonstrated robust performance in both training and validation datasets. ctDNA-positive samples exhibited higher predicted fractions (training: median = 0.10, range = 0 - 0.40; validation: median = 0.10, range = 0 - 0.30) compared to ctDNA-negative samples (training: median= $\text{2}.\text{9}*{\text{1}\text{0}}^{-\text{5}}$, range = 0-0.002; validation: median=$\text{2}.\text{3}*{\text{1}\text{0}}^{-\text{3}}$, range = 0-0.13) across all cohorts. For each sample, S1 and S2 Tables present the tumor‐fraction predictions from both ALFAssay and ichorCNA in the training and validation sets.

Additionally, we benchmarked ALFAssay results on the validation dataset against a LASSO model that predicted tumor fraction from the same feature space that we used for training ALFAssay model. For both models, continuous outputs were converted into binary ctDNA calls (positive vs. negative) using decision thresholds that were locked based on the training data (cut-offs: 0.008 for ALFAssay and 0.01 for the LASSO model). The threshold was determined in the training set by tracing the ROC curve of predicted tumor fraction against ichorCNA-derived ground-truth labels and selecting the optimal cut-off using Youden’s J (see Methods). Ground-truth labels defined ctDNA-positive samples as those with ichorCNA tumor fraction ≥0.03 and ctDNA-negative samples as <0.03. Once established, this threshold was fixed and applied unchanged to all validation samples.

Using these locked thresholds, ALFAssay showed slightly better overall performance on the validation dataset compared to the LASSO model. It achieved a marginally higher accuracy (0.92 vs. 0.90), specificity (0.94 vs. 0.92), and PPV (0.91 vs. 0.89), while sensitivity(0.88 vs. 0.88) and NPV (0.92 vs. 0.92) remained identical between the two models. However, none of these differences reached statistical significance across the evaluated metrics (S3 Table).

Next, we compared ALFAssay results against a LASSO model, the Fragle model, and a composite ALFAssay+Fragle predictor (see Methods). All models were evaluated on Pearl cohort(validation dataset) against three independent references: (i) ichorCNA ctDNA detection (tumor fraction ≥ 0.03), (ii) OncoFollow, a mutation-based ctDNA detection assay (see Methods) - positive if any tested gene was mutated), and (iii) progression-free survival (PFS) dichotomized at 6 months. Across these benchmarks, ALFAssay, and the composite model(ALFAssay+FRAGLE) showed comparable performance and outperformed both the Fragle and LASSO models for the ichorCNA and PFS references, while for the OncoFOLLOW reference, ALFAssay and LASSO performed similarly and better than both Fragle and the composite model (S2 Fig in S1 File).

Against ichorCNA, the AUCs were 0.963 for ALFAssay, 0.931 for LASSO, 0.911 for Fragle, and 0.954 for ALFAssay+Fragle. Using OncoFollow as reference, AUCs were 0.806, 0.801, 0.737, and 0.788, respectively. For PFS ≤ 6 months vs. > 6 months, the AUCs were 0.769, 0.730, 0.733, and 0.765 (S6 Table).

### ALFAssay ctDNA fraction correlation with established tools

**Fig 4:**
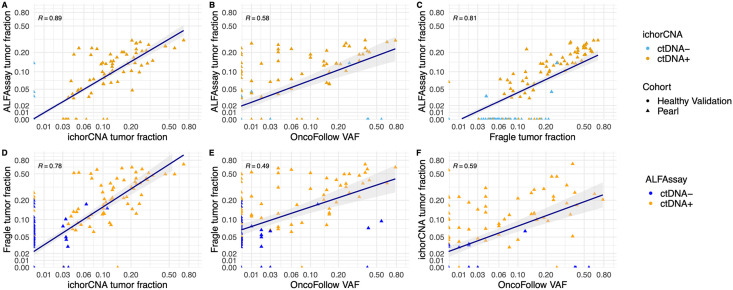
Concordance of ALFAssay with orthogonal ctDNA quantification and cross-model correlations on the Pearl cohort. A-C. ALFAssay-predicted tumor fraction vs A ichorCNA tumor fraction B OncoFollow variant allele frequency (VAF), and C Fragle tumor fraction. D-F. Pairwise correlations between D ichorCNA and Fragle tumor fractions, E Fragle tumor fraction and OncoFollow VAF, and F ichorCNA tumor fraction and OncoFollow VAF. Each point is a sample. In A-C, points are colored by ichorCNA detection (blue, ctDNA-; orange, ctDNA + ;ctDNA+ if ichorCNA tumor fraction >= 3%) and shaped by cohort (circles, healthy; triangles, metastatic breast cancer). In D-F, points are colored by ALFAssay detection (blue, ctDNA-; orange, ctDNA + ; ctDNA+ if ALFAssay tumor fraction>= 0.015) and shaped by cohort. Spearman correlation coefficients (R) are shown in each panel. Tumor-fraction values < 0.03 were set to 0.

To further assess the validity of our predictions, we compared ctDNA fractions estimated by ALFAssay with those generated by established tools(Figs 4A-4C and S3 in S1 File). The tumor fraction estimated by ichorCNA showed strong concordance with ALFAssay predictions (on Pearl cohort, R = 0.89, p < 0.0001), consistent with the fact that ALFAssay was trained on ichorCNA-derived tumor fractions and used as input the total number of reads together with fragmentation patterns. Additionally, predictions from Fragle demonstrated concordance with ALFAssay outputs (on Pearl cohort, R = 0.81, p < 0.0001).

Furthermore, for the validation cohort, we leveraged available results from ONCOFollow. Here, we used variant allele fraction (VAF) as a surrogate for ctDNA fraction and evaluated its correlation with ALFAssay tumor fraction predictions. The correlation was lower than with the sWGS-based tools (r = 0.58), suggesting the possibility that the two approaches are complementary.

To benchmark these estimates against other tools, we assessed cross-method concordance centered on ichorCNA in the validation cohort (Fig 4D-4F). ONCOFollow variant allele fractions (VAFs) were moderately correlated with ichorCNA tumor fraction (R = 0.59), consistent with the distinct biology captured by mutation-based versus sWGS-based readouts. In contrast, the fragmentation-derived tumor-fraction estimated by Fragle showed stronger agreement with ichorCNA (R = 0.78), and Fragle and ONCOFollow were themselves moderately correlated (R = 0.49). Tumor fraction values < 0.03 were set to zero in ALFAssay, ichorCNA, and Fragle to restrict analysis to quantifiable ctDNA levels above the detection threshold.

Interestingly, within the subset of PEARL samples(validation dataset) with undetected ichorCNA signal (ichorTF ≤ 0.03; n = 58), ALFAssay predicted higher ctDNA levels (PredictedValue ≥ 0.03) in 3 samples. Among these, 2 samples also exhibited detectable mutation-derived VAF (by OncoFOLLOW), while all 3 showed high Fragle ctDNA burden, indicating the presence of ctDNA-associated fragmentation signal in a subset of ctDNA negative samples calculated by ichorCNA that may not be readily detected by copy-number-based approaches alone.

### Risk stratification analysis based on ctDNA fraction prediction

We evaluated the clinical utility of ALFAssay for risk stratification by assessing its association with progression-free survival (PFS) in the Pearl validation cohort.

**Fig 5:**
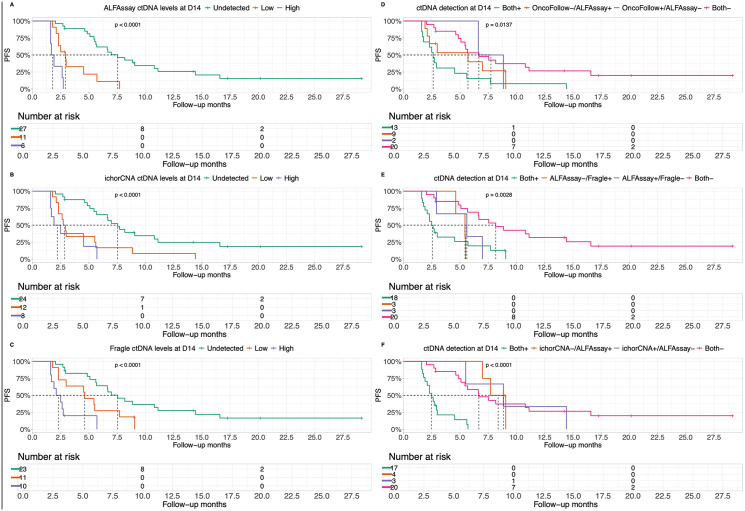
Kaplan–Meier estimates of progression-free survival (PFS) according to ctDNA levels and detection status at Day 14. (A–C) PFS curves stratified by ctDNA levels derived from ALFAssay (A), ichorCNA (B), and Fragle (C). For ALFAssay, ctDNA levels were defined as Undetected (< 0.03), Low (0.03 – 0.2), and High (> 0.2). For ichorCNA, ctDNA levels were defined as Undetected (< 0.03), Low (0.03 – 0.2), and High (> 0.2). For Fragle, thresholds were chosen to match the number of patients per category in ALFAssay: Undetected (< 0.08), Low (0.08–0.37), and High (> 0.37). (D–F) PFS curves stratified by pairwise ctDNA detection based on Day 14 ctDNA positivity (ALFAssay was considered positive if tumor fraction was ≥ 0.03, ichorCNA was considered positive if tumor fraction was ≥ 0.03, Fragle was considered positive if tumor fraction was ≥ 0.08) across two models: (D) ALFAssay + OncoFollow, (E) ALFAssay + Fragle, and (F) ALFAssay + ichorCNA. Curves show the proportion without progression over time (months). Log-rank p-values indicate between-group differences.

We stratified ctDNA tumor fraction into three categories: Undetected, Low, and High. For ALFAssay and ichorCNA, samples with ctDNA levels < 0.03 were defined as Undetected, between 0.03 and 0.2 as Low and > 0.2 as High. The lower threshold (0.03) corresponds to the reported limit of detection of ichorCNA and the upper threshold (0.2) was defined empirically to distinguish samples with relatively high ctDNA burden from those with lower levels. For Fragle, thresholds were chosen to match the number of patients per category by ALFAssay model: Undetected (< 0.08), Low (0.08–0.37), and High (> 0.37) (Figs 5A–5C and S4A–S4C in S1 File). Progression-free survival (PFS) was analyzed in the independent Pearl validation cohort and visualized using Kaplan–Meier curves. Across all three models, higher ctDNA tumor fractions were associated with shorter PFS, whereas patients with undetectable ctDNA showed more favorable outcomes. For the pairwise assay comparisons—ALFAssay + ichorCNA, ALFAssay + Fragle, and ALFAssay + OncoFollow—we applied a binary classification: samples were considered ctDNA-negative if classified as Undetected in both assays, and ctDNA-positive if they fell into either the Low or High category in at least one assay (Figs 5D–5F and S4D–S4F in S1 File). In all pairwise comparisons, ctDNA-positive classifications given by both models tended to be associated with shorter PFS, suggesting that each approach—whether combining different measurement modalities such as copy number aberrations with ichorCNA and fragmentation patterns with ALFAssay, or distinct fragmentomic models like ALFAssay and Fragle—captures partially complementary aspects of ctDNA signal that may enhance prognostic assessment. However, shared information between ALFAssay and ichorCNA due to the training procedure should be considered, as ALFAssay was trained on ichorCNA-derived tumor fractions, introducing a degree of dependency between the two methods that may contribute to their complementarity. Although nested Cox model comparisons (fm1 = coxph(Surv(PFS, status) ~ x); fm2 = coxph(Surv(PFS, status) ~ x + y); anova(fm1, fm2)) did not reach statistical significance at baseline for any model combination (all p > 0.07), at D14, few combinations reached statistical significance, indicating that ctDNA dynamics captured at D14 by different models can give complementary prognostic information (S5 Table).

Additionally, we performed a univariate Cox proportional hazards analysis at baseline and after 14 days of therapy (D14) timepoints (S6 Fig in S1 File) comparing ALFAssay with different ctDNA detection methods and their pairwise composite defined as being positive if either model is positive; negative only if both models are negative.

At both baseline and D14, all four single models showed statistically significant associations with progression (all p<=0.01). Pairwise combinations (see Methods) at baseline yielded hazard ratios between 2.56 and 3.73 (all p < 0.01). At D14, all pairwise combinations were statistically significant with hazard ratios between 2.42 and 3.29 (p<=0.01). These results indicate that ctDNA dynamics captured by ALFAssay, either alone or in combination with other models, can provide prognostic information, as patients with detectable ctDNA exhibited shorter (PFS).

We next performed pairwise comparisons of prognostic value between ALFAssay, Fragle, ichorCNA, and OncoFollow. For each model pair and each timepoint (baseline and D14), we fitted two Cox models on the same patient subset—a base model including a single marker and an extended model including that marker plus the second marker—and compared them using likelihood-ratio tests for PFS. As summarized in S4 Table, no pairwise comparison reached statistical significance at baseline (all p ≥ 0.06), although adding ichorCNA or Fragle to OncoFollow showed borderline improvements in model fit. At D14, the combination of OncoFollow plus ichorCNA significantly improved model fit compared with OncoFollow alone (LR χ² = 9.47, df = 1, p = 0.002), indicating that ichorCNA provides additional prognostic information beyond OncoFollow at this timepoint. Similarly, the combination of ALFAssay and ichorCNA also improved model fit compared with ALFAssay alone (LR χ² = 7.76, df = 1, p = 0.005), suggesting that ichorCNA adds complementary prognostic value to ALFAssay. All other comparisons at timpeoint D14 were non-significant.

## Discussion

This study presents ALFAssay, a deep learning model that leverages cfDNA fragmentation patterns to estimate tumor fraction in breast cancer patients. Our approach provides a complementary fragmentation-based strategy for ctDNA estimation in breast cancers characterized by relatively low copy number aberration burden [9,10]. Our model learns the relationship between copy-number-derived tumor fraction and cfDNA fragmentation patterns in the CNA-informative subset and projects this mapping to a fragmentation-based model. The model may provide complementary information when integrated with other approaches, although this complementarity should be interpreted cautiously given the shared training signal with ichorCNA. By training and validation on fragmentation features derived from sWGS data across 896 plasma samples from diverse breast cancer datasets, ALFAssay shows robust performance in distinguishing between healthy controls and metastatic breast cancer patients while providing quantitative tumor fraction estimates.

The architectural design of ALFAssay incorporates several technical approaches that aim at enhancing its performance and limit the technical bias. GC bias correction was performed at the fragment level using GCParagon, and stringent quality filters were applied to ensure analysis of high-confidence reads only. The inclusion of bin-level total fragment counts alongside fragmentation ratios is intended to allow the model to account for coverage-dependent effects during training while preserving potentially informative variation in local fragment abundance. In addition to helping distinguish biologically relevant fragmentation patterns from technical variability related to sequencing depth [22–24], total fragment counts may themselves contain predictive information related to cfDNA fragmentation and chromatin organization. The model therefore receives both fragmentation ratios and total fragment counts as complementary input features.

ALFAssay demonstrates performance characteristics comparable to existing tools for tumor fraction estimation in cfDNA. Although exploratory analysis identified a small subset of samples with low or undetected ichorCNA signal in which ALFAssay remained elevated and was supported by orthogonal fragmentation- and mutation-based evidence, the number of such cases was limited, precluding meaningful clinical stratification or outcome analysis. These observations nevertheless suggest that ALFAssay may capture ctDNA-associated signals in selected samples where CNA-based quantification underperforms. Further validation in larger cohorts enriched for low-CNA or low-shedding tumors will be required to determine whether fragmentation-based approaches such as ALFAssay provide added value beyond CNA-based methods in these settings. A key distinction of ALFAssay compared to other fragmentation-based approaches, such as Fragle, lies in its breast cancer–specific training and feature preprocessing pipeline. While Fragle uses convolutional neural networks to process raw fragment length distributions, ALFAssay employs a fully connected architecture optimized for a coverage-corrected feature space. This design choice balances predictive capacity with computational efficiency, making the method lightweight enough for integration into existing analysis workflows while retaining high classification accuracy (AUC = 0.96 in validation) and specificity (0.94). The model showed slightly higher accuracy and specificity compared to the LASSO model, suggesting that the deep learning architecture may capture additional non-linear relationships within the fragmentomic data [25,26].

The prognostic value of ALFAssay as a single model was assessed through PFS. At both baseline and D14 timepoints, ctDNA tumor fraction levels by ALFAssay demonstrated significant prognostic discrimination. These findings indicate that ALFAssay can provide meaningful prognostic information both pre-treatment and during therapy, with the latter reflecting the added value of capturing on-treatment dynamics.

Survival analyses on pairwise combinations of ALFAssay with ichorCNA, Fragle, or OncoFollow, revealed that patients whose samples were classified as ctDNA-positive by both models within a pair had shorter PFS. This pattern was visible not only for the complementary approaches based on copy number or mutation detection (ichorCNA and OncoFollow) but also for Fragle, which, like ALFAssay, relies on cfDNA fragmentation features. These findings suggest that although ALFAssay and Fragle are both fragmentation-based, they capture distinct and complementary aspects of the cfDNA signal, each contributing unique information to ctDNA assessment.

This complementarity may also reflect the fact that, although all models aim to minimize bias and maximize generalizability, each inevitably introduces its own biases and sensitivities when interpreting complex cfDNA signals [27]. Because it is difficult to correct for all sources of bias—arising from biological heterogeneity, sequencing variability, or modeling assumptions—combining outputs from multiple models addressing the same problem from different perspectives can help mitigate individual model limitations.

Similar ensemble or multimodal approaches have been shown to improve predictive robustness and reduce model-specific bias in cfDNA analysis and cancer detection by integrating multiple signals—such as fragmentation profiles, methylation patterns, and copy-number features—into stacked or composite models [28,29,30]. Although these frameworks typically combine distinct molecular features, the same principle applies to models that rely on the same underlying signal but extract and represent it differently. This reflects the general principle behind ensemble learning, where multiple imperfect or “weak” learners, each capturing different facets of the data, can collectively achieve higher accuracy and better generalization than any single model alone. In this context, the strength of ALFAssay lies in its ability to complement other approaches, contributing to more robust and reliable ctDNA assessment when integrated in a multi-model framework.

Correlation analyses further supported the complementary nature of the models, revealing that ALFAssay correlated strongly but incompletely with ichorCNA (R = 0.89) and Fragle (R = 0.81), while showing a weaker correlation with mutation-based variant allele fraction (VAF) from targeted sequencing (R = 0.58). These observations highlight two important points: first, fragmentation-based and CNA-based quantifications detect overlapping but also distinct biological signals; second, mutation-based ctDNA detection captures yet another orthogonal dimension of tumor biology. Mutation-based detection offers high specificity for known driver alterations but may miss tumors with low mutational burden or novel genetic alterations [31,32].

Several limitations of our study warrant discussion. First, the ground truth for model training relied on ichorCNA estimates with a 3% tumor fraction threshold [8], which introduces inherent uncertainty into the training process. This dependency on another computational method rather than orthogonal validation techniques may limit the absolute accuracy of ALFAssay predictions. As a result, ALFAssay cannot be considered an independent improvement over ichorCNA, and comparisons or combinations involving the two methods may be affected by partial circularity. Future studies incorporating spike-in control methods could provide more definitive validation of fragmentation-based tumor fraction estimates. Gulley et al. demonstrated that introducing synthetic spike-in normalizers into plasma DNA sequencing enables more precise quantification of both tumor markers and viral genomes, reducing variability introduced by sequencing depth and technical artifacts [33]. Applying such approaches to cfDNA fragmentation analysis would not only allow calibration of tumor fraction predictions across platforms but could be particularly valuable in the low ctDNA range (below the ~ 3% detection threshold of ichorCNA), where distinguishing biological signal from noise is most challenging. In parallel, as emphasized by Collins et al., rigorous and transparent reporting standards for artificial intelligence–based prediction models are essential to ensure reproducibility, generalizability, and clinical reliability [34]. Together, integrating technical spike-in controls with standardized model reporting could enhance both the analytical robustness and translational potential of methods like ALFAssay.

The cohort-specific performance variations observed in our analysis highlight potential generalizability challenges. While ALFAssay demonstrated consistent performance across the training cohorts (Neorhea and Synergy), the validation was limited to a single independent cohort (Pearl). The differences in cancer subtypes, treatment regimens, and sample collection protocols across these studies may influence fragmentation patterns in ways not fully captured by our current model [11,13]. Larger, more diverse validation cohorts will be essential for establishing the analytical validity of this approach [35].

The computational requirements and infrastructure needed for ALFAssay implementation represent important practical considerations. At this stage, although the model itself is relatively lightweight compared to more computationally intensive deep learning frameworks, the upstream data processing still demands bioinformatics expertise and dedicated computational resources, which may limit accessibility for broader use [36]. Streamlined and automated analysis pipelines will therefore be essential not only to facilitate wider implementation across research settings but also to ensure reproducibility and consistency of results across cohorts and sequencing platforms [37].

Several research directions emerge from our findings that could improve, consolidate and extend ctDNA tumor fraction estimation tools in the directions already outlined. Building larger and more diverse training datasets will be key to strengthening model generalizability, particularly across less common breast cancer subtypes and underrepresented populations. Another priority will be to embed fragmentation analysis within multi-modal frameworks, where integration with other molecular and imaging biomarkers could yield a more comprehensive view of tumor biology. In parallel, refining temporal sampling strategies and probing treatment-induced changes in fragmentation profiles may uncover new opportunities for response monitoring and resistance tracking. Finally, to move toward broader applicability, efforts should focus on developing standardized and automated pipelines, enabling reproducible integration of fragmentation-based tumor fraction estimation tools such as ALFAssay into multi-modal liquid biopsy workflows.

In summary, ALFAssay contributes to the growing set of bioinformatics approaches for tumor fraction estimation from cfDNA by introducing a fragmentation-based deep learning model applied to metastatic breast cancer. The model incorporates steps to reduce technical bias and is computationally efficient, while showing performance comparable and potentially complementary to other fragmentation-, CNA- and mutation-based methods. These characteristics suggest its potential usage within multi-modal ctDNA monitoring strategies. Nevertheless, further work is needed to calibrate tumor fraction estimates using orthogonal methods and to validate performance in larger and more diverse cohorts to establish robustness and clinical relevance.

## Methods

### Ethics statement

The trials were approved by the institutional review boards and ethical committees of each participating center. Written informed consent was obtained from all patients before enrolment. The studies were conducted in accordance with the principles of Good Clinical Practice and the provisions of the Declaration of Helsinki and applicable local regulations.

The NeoRHEA, PEARL, SYNERGY, and healthy donor studies were approved by the Ethics Committee of Institut Jules Bordet (Brussels, Belgium; accreditation number OM011), which served as the Institutional Review Board/Ethics Committee for the studies.

### Datasets description

We utilized shallow whole genome (sWGS) cfDNA sequencing data from three studies. We included 349 samples from the SYNERGY phase I/II clinical trial. The SYNERGY trial was registered on ClinicalTrials.gov (https://clinicaltrials.gov/study/NCT03616886) (ID: NCT03616886). This trial involved 128 pre- or post-menopausal women with previously untreated, locally recurrent inoperable, or metastatic triple-negative breast cancer (TNBC). As part of the translational research objectives of the SYNERGY trial, blood samples were collected at the following timepoints: at baseline before treatment initiation, during treatment at week 3, and at the end of chemotherapy at week 13 [20]. Samples with coverage below 0.2 were excluded from the cohort, resulting in the removal of five samples across three patients.

We analyzed 132 samples from the Pearl study, which included 48 women diagnosed with HR + /HER2- metastatic breast cancer who were treated with exemestane and everolimus. Blood samples were collected at three key timepoints: before treatment (BL), after 14 days of therapy (D14), and at the time of disease progression (PD). In this study, ctDNA was analyzed using the OncoFOLLOW assay—a targeted next-generation sequencing panel developed by OncoDNA Inc. The assay is a tumor-agnostic test designed to monitor tumor burden and treatment response through the detection of recurrent point mutations in a predefined set of cancer-related genes (covering 40 genes, including key breast cancer drivers such as *PIK3CA*, *ESR1*, and *TP53*). The results of this analysis have been previously published [21]. The Pearl trial study is registered on ClinicalTrials.gov (https://clinicaltrials.gov/ct2/show/NCT02028364) (ID: NCT02028364).

We included 267 samples from the Neorhea trial. Two additional manuscripts describing the main objectives of the trial and the ctDNA analysis are currently in press. The Neorhea trial (NCT03065621) was an open-label, multicenter, single-arm phase II study involving 98 pre- or post-menopausal women diagnosed with ER-positive, HER2-negative early breast cancer. Participants received four cycles of palbociclib at 125 mg (administered from Day 1 to Day 21 of each cycle, followed by a 7-day break), in combination with continuous endocrine therapy (administered from Day 1 to Day 28 of each cycle). Blood samples for plasma analysis were collected at multiple timepoints: after enrolment and before Day 1 of Cycle 1 (baseline), before Day 1 of Cycle 2 (C1D28), on the day of surgery (pre-operative), and one month after surgery (post-operative). The Neorhea trial study is registered on ClinicalTrials.gov (https://clinicaltrials.gov/study/NCT03065621) (ID: NCT03065621).

The control samples were collected from 98 healthy women who participated in the “Fragmentation patterns of plasma cell-free DNA for ctDNA detection and quantification. Healthy subject cohort” study at Jules Bordet’s cancer screening clinic. These women were asked to donate blood samples between 01.12.2021 - 01.10.2023 (EC3369). Initially, all 98 samples were sequenced in two separate runs. A third sequencing run was conducted with only 50 samples, which were processed alongside the Synergy samples.

### Sample collection, preparation, and sequencing

10 ml whole blood was collected in EDTA tubes. Plasma was isolated within max 30 minutes after blood collection by double centrifugation: first at 820 g at 4°C for 10 minutes and at full speed (18.000-20.000 g) for 10 more minutes thereafter. Plasma samples were stored at -70°C at the central laboratory at Institut Jules Bordet in Brussels.

cfDNA was extracted from blood using the Qiagen DNA Blood Mini Kit (Qiagen, Valencia, USA). DNA quantity was measured using the Qubit 2.0 Fluorometer (Thermo Fisher Scientific, Waltham, USA).

DNA extraction from plasma and DNA sequencing were performed at Brightcore, genomics core facility of the Free University of Brussels (VUB/ULB) (https://www.brightcore.be/). A total of 3–4 ml plasma was used for DNA isolation using the Promega Maxwell RSC cfDNA Plasma kit on the Maxwell RSC 48 extraction robot (Promega, Madison, WI, USA) according to manufacturer instructions. The maximum available amount of DNA (25 µl) was used in the KAPA Hyper Prep (Roche Sequencing, CA, USA) library preparation according to manufacturer recommendations, with four exceptions: (1) the volumes were reduced with a factor 2, (2) Unique Dual Indexed (UDI) adapters of own design (Integrated DNA Technologies, Coralville, IA, USA) were used at a concentration of 1,5 µM, (3) a 1x post ligation bead cleanup with AMPure XP beads (Beckman Coulter Life Sciences, IN, USA) was performed and (4) a total of 15 PCR cycles were applied to get sufficient library for sequencing. Final libraries were quantified on the PerkinElmer Victor Nivo 3F (PerkinElmer, Waltham, MA, USA) using the Qubit dsDNA BR Assay Kit (Life Technologies, CA, USA) and qualified on the PerkinElmer Caliper GX II with the HT DNA 5K kit (PerkinElmer, Waltham, MA, USA). Samples were sequenced on the Illumina NovaSeq 6000 system (Illumina Inc., CA, USA), with the NovaSeq 6000 S4 Reagent Kit. For this, 1,5 nM libraries were denatured according to the manufacturer instructions. The raw basecall files were converted to fastq files with Illumina’s bcl2fastq algorithm (version 2.19). Average coverage was around 1x for all samples and read length was 100 base pairs (bp) for 449 samples and 50 bp for 447 samples.

### Data pre-processing

All samples fastq files were pre-processed by the same in-house pipeline. Fastq files quality checks and adaptor trimming were done with fastp [38]. The same tool was used to trim the reads coming from samples with 100 bp read length to 50 bp. The reads were aligned to the hg38 genome version with bwa mem, version 0.7.17 [39] and the bam files were sorted with samtools [40]. Sambamba [41] was used to mark the duplicated and mosdepth [42] to assess the coverage. The GC correction was performed with GCParagon [43], specifically designed for GC correction on sequencing data coming from cfDNA. IchorCNA [8] was run with 1Mb window size and with a panel of normal created from the control samples.

### Tumor fraction prediction model

#### Fragment size ratios calculation (input) and tumor fraction (output).

Fragment sizes were calculated using an in-house script. The fragments extracted from the GC corrected bam files were further filtered to ensure only proper paired reads were selected. Fragments not within a list of high confidence genomic regions were filtered out. The list was created on genomic bins of a 5M bp window length using the github package asntech/QDNAseq.hg38@main [44]. Additionally, we filtered the genomic regions by selecting reads with mappability higher than 70, overlap with ENCODE blacklisted regions lower than 20%, and whether the bin should be used in subsequent analysis steps. This filtering step resulted in analyzing 204 genomic bins for each sample.

Next, we counted the number of short (90–150 bp), and all (30–700 bp) fragments within a genomic window of 5M base pairs across the genome for each sample. Each fragment counting was using the weighted fragments provided by the GCParagon tool. The fragment counts on genomic window bins per sample were further used to calculate the ratio of short fragments over all fragments. These features together with all fragments per bin (a matrix of shape 408) for each sample were used as input to our model.

The ground truth was considered as the tumor fraction calculated by ichorCNA based on their reported 0.03 level of confidence. Tumor fractions of all samples with tumor fraction lower than 0.03 were set at 0.

#### Model architecture.

The fragment‑ratio profiles (1 × 408 matrice per sample) were then flattened to 408‑dimensional vectors and passed to a fully connected feedforward neural network (FFNN) to model the supervised regression task of predicting tumor fraction from fragment size ratios. Training was carried out with a batch size of 30 samples.

The FFNN architecture comprises four fully connected layers with decreasing dimensionality. The first layer projects the 408-dimensional input to 304 units, followed by layer normalization and a ReLU activation function. This is followed by two hidden layers with 304 units each respectively, each followed by layer normalization and ReLU activation. The final output layer consists of a single linear unit producing a scalar value.

Mathematically, for a given input vector $x\in R^{\text{2}\text{0}\text{4}}$, the network computes:


$$\begin{array}{c} h_{\text{1}}\;=\;ReLU(LayerNorm(W_{\text{1}}\;x\;+b_{\text{1}})) \end{array}$$

$$\begin{array}{c} h_{\text{2}}\;=\;ReLU(LayerNorm(W_{\text{2}}\;h_{\text{1}}+b_{\text{2}})) \end{array}$$

$h_{\text{3}}\;=\;ReLU(LayerNorm(W_{\text{3}}\;h_{\text{2}}+\;b_{\text{3}}))$


$$\begin{array}{c} \hat{y}\;=\;W_{\text{4}}\;h_{\text{3}}\;+\;b_{\text{4}} \end{array}$$

where $W_{i}$ and $b_{i}$ are the weight matrices and bias vectors of the i-th layer, respectively. Layer normalization is applied across each sample prior to activation.

The network was trained to minimize the root mean squared error (RMSE) between the predicted outputs $\hat{y}$ and the ground truth values y. The loss function is defined as:


$$\begin{array}{c} L_{RMSE}\;=\sqrt{\frac{1}{N}\overset{\;}{\underset{\;}{\overset{\;}{\underset{\;}{\sum_{i=1}^{N}{\left(y_{i}-\hat{y_{i}}\right)}^{2}}}}}} \end{array}$$

where N is the number of samples in the batch.

Optimization was performed using the Adam optimizer [45], with weight decay applied to regularize the model and prevent overfitting. A learning rate scheduler was incorporated to automatically reduce the learning rate when the validation loss plateaued, enabling finer convergence during later training stages. Models were implemented using PyTorch.

#### Model training and assessment.

Model training was conducted using 5-fold cross-validation for 4500 epochs. For training we used the Synergy cohort, the Neorhea cohort and 100 healthy samples. The Pearl cohort and 48 control samples were used for model validation on unseen data.

### LASSO regression model

The LASSO regression model was trained to predict tumor fraction from genome-wide fragmentation features. The model was implemented in Python using scikit-learn (LassoCV). Input features consisted of the total fragments count and ratio of short to to total fragment counts values across genomic bins. Prior to model fitting, missing feature values were imputed using the median value of each feature, and features were standardized using z-score normalization with StandardScaler, such that each feature was centered to mean 0 and scaled to unit variance based on the training set.

The regularization strength parameter, alpha, was selected by five-fold cross-validation within the training cohort. The final model was then fitted on all training samples using the selected alpha and applied to the independent validation cohort. The ground-truth ichorCNA tumor fraction was converted to percentage scale, with values below 0.03 set to 0 and values equal to or above 0.03 multiplied by 100.

### Cut-off derivation for the ALFAssay and LASSO models

The binary ground truth was the presence of ctDNA calculated by ichorCNA with positive samples having tumor fraction>= 3% and negative otherwise. Predicted tumor fractions from ALFAssay and the LASSO model were used as continuous predictors.

For the primary comparison between ALFAssay and LASSO, classification thresholds were determined on the training cohorts (Synergy, NeoRhea, and healthy samples).

We selected a decision cut-off on the training set using Youden’s J statistic on the ROC curve:


$$\begin{array}{c} TPR(t)=TP(t)/(TP(t)+FN(t)), \end{array}$$

$$\begin{array}{c} FPR(t)=FP(t)/(FP(t)+TN(t)), \end{array}$$

$$\begin{array}{c} J(t)=TPR(t)-FPR(t). \end{array}$$

The optimal threshold was defined as:


$$\begin{array}{c} t^{*}\;=\;arg\;max\_t\;J(t), \end{array}$$

computed by sweeping all unique score values and computing (FPR(t), TPR(t)) via the ROC curve.

The resulting threshold was fixed and applied unchanged to the validation cohorts. Using these locked thresholds, we computed threshold-dependent performance metrics on the validation data, including accuracy, sensitivity, specificity, positive predictive value (PPV), and negative predictive value (NPV).

To quantify uncertainty, 95% confidence intervals (CIs) for validation metrics were estimated using a non-parametric percentile bootstrap (2,000 resamples with replacement at the sample level), with thresholds held fixed in each resample.

### Comparative analysis

We evaluated ALFAssay against three alternative models: a LASSO model, Fragle, and a composite ALFAssay+Fragle predictor. All models were trained (where applicable) on the same training cohorts and evaluated on the same independent validation datasets.

Two complementary comparison strategies were used.

First, a direct comparison between ALFAssay and the LASSO model was performed using thresholds derived on the training data, as described above. This enabled a consistent binary classification comparison using fixed decision rules across validation datasets

Second, a broader comparison including ALFAssay, LASSO, Fragle, and the composite ALFAssay+Fragle model was conducted using ROC curve analysis on the validation datasets. Since Fragle and the composite model do not have thresholds defined on the training data, model-specific thresholds were instead determined directly on the validation set using Youden’s J criterion. These thresholds were used solely to compute threshold-dependent metrics (accuracy, sensitivity, specificity, PPV, and NPV) for this comparison.

The Fragle model generated a continuous predicted tumor fraction. Before constructing the composite ALFAssay + Fragle metric, Fragle scores were normalized onto the ALFAssay scale using a linear calibration model. Specifically, for all samples with both measurements available, we fit a simple linear regression of the form:


$$\begin{array}{c} ALFAssay=a+b\times Fragle, \end{array}$$

where *a* and *b* are the fitted intercept and slope, respectively. Each Fragle value was then transformed using this calibration function, with results clipped to the [0,1] range. The composite score was computed as the arithmetic sum of the calibrated Fragle score and the ALFAssay predicted tumor fraction.

All four models—ALFAssay, Regression, Fragle, and ALFAssay+Fragle—were evaluated against three independent references: (i) ichorCNA ctDNA detection (tumor fraction ≥ 0.03), (ii) OncoFollow mutation-based detection (positive if any tested gene was mutated), and (iii) Progression-free survival (PFS) dichotomized at 6 months. Performance metrics were computed as defined above..

### Statistical analysis

The statistical tests used are always stated when a p-value is specified. Tests were considered as significant if p < 0.05. To assess the difference in tumor fraction prediction by ALFAssay in different cohorts Wilcoxon statistical test was used. Spearman correlations were used.

The comparison with a basic regression model was performed using two complementary paired tests: McNemar’s exact test (two-sided) and a paired bootstrap. Because both models were evaluated on the same individuals, all inferences explicitly accounted for pairing.

For accuracy, sensitivity, and specificity we applied McNemar’s exact test. For each of these metrics, we constructed paired hit/miss indicators on the relevant subset of samples (accuracy on all samples as correct versus incorrect; sensitivity within the ctDNA-positive subset with a hit defined as predicting 1; specificity within the ctDNA-negative subset with a hit defined as predicting 0). Let *b* denote the number of cases where the regression model was correct and ALFAssay was wrong, and *c* the number where the regression model was wrong and ALFAssay was correct. Under the null hypothesis of equal performance, min(*b*,*c*) ~ Binomial(*b* + *c*, 0.5); we report the corresponding two-sided exact binomial *p*-value.

To quantify effect sizes and uncertainty—and to handle metrics for which McNemar’s test is not applicable (PPV and NPV, which condition on model-dependent predicted sets)—we used a non-parametric paired bootstrap for accuracy, sensitivity, specificity, PPV, and NPV. The effect size for each metric was the paired difference Δ = ALFA − LASSO. Patients were resampled with replacement (preserving pairs) for 10,000 replicates. We formed 95% confidence intervals as percentile intervals from the bootstrap distribution of Δ and computed a two-sided bootstrap *p*-value as *p* = 2 × min{Pr(Δ ≤ 0), Pr(Δ ≥ 0)}.

Univariate Cox proportional hazards models were applied to each individual model (ALFAssay, OncoFollow, ichorCNA, Fragle) and to all pairwise combinations. Samples were classified as ctDNA‑positive by ALFAssay and ichorCNA when the estimated tumor fraction was greater than 0.03by Fragle when it was greater than 0.08, and by OncoFollow when at least one mutation was detected. Fragle thresholds were chosen to match the number of patients per category in ALFAssay related to PFS. In each pairwise analysis, a sample was considered positive if either model in the pair was positive. Models were fitted for progression‑free survival (PFS), yielding hazard ratios (HRs), 95% confidence intervals (CIs), and Wald p‑values. HRs and CIs are reported as “HR (lower–upper),” and p‑values as “p < 0.001” if below 0.001 or to three decimal places otherwise.

Patients were analyzed separately per timepoint and the survival outcome was PFS, with time defined by PFS and the event indicator set to 1 for progression/death (censoring otherwise). Model calls for ALFAssay, Fragle, ichorCNA, and OncoFollow were dichotomized (Positive = 1, Negative = 0). We used complete-case analysis after excluding rows with missing outcome or model values.

For each timepoint, we evaluated the prognostic contribution of ALFAssay, Fragle, ichorCNA, and OncoFollow using pairwise Cox proportional hazards model comparisons (Efron method for ties). For each unordered marker pair, we fit a base model containing a single assay and an extended model containing both assays, using identical patient subsets. Incremental predictive value was assessed with likelihood-ratio ANOVA tests (H₀: the added marker does not improve model fit). Reported p-values are unadjusted. All analyses were performed in R using the survival and car packages.

Survival analysis was visualized using a Kaplan-Meier curve and the significance was assessed using a log-rank statistical test. For patients enrolled in the Neorhea study, the breast cancer free survival event was defined as locoregional and distant relapse and the time was calculated from the date of first blood sample collection until the date of relapse. For patients enrolled in the Pearl study the PFS was defined as the time elapsed between the baseline and progression, or death, whichever occurs the first. Progression was documented based on traditional RECIST 1.1 criteria. For patients enrolled in the Synergy study, PFS was defined as the time from the first study drug administration to the first documented disease progression based on RECIST v1.1 or death due to any cause, whichever occurs first. Patients who were alive and progression-free at the time of analysis were censored at the time-point of their last tumor assessment by imaging.

All analysis was performed with Python 2.7.13 and R version 3.4.2.

## Supporting information

S1 File Supplementary figures, additional survival analyses, ROC analyses, cross-model correlations, Cox proportional hazards analyses, and CONSORT 2025 checklist for the NeoRHEA, SYNERGY, and PEARL studies. (DOCX)

S2 File Ethics committee approval document for the study “Fragmentation patterns of plasma cell-free DNA for ctDNA detection and quantification”. (PDF)

S1 Table Tumor fraction predictions for samples included in the training dataset, including ALFAssay and ichorCNA estimates across the NeoRHEA, SYNERGY, and healthy control cohorts. (CSV)

S2 Table Tumor fraction predictions for samples included in the validation dataset, including ALFAssay and ichorCNA estimates across the PEARL and healthy validation cohorts. (CSV)

S3 Table Performance comparison between ALFAssay and the LASSO regression model on the validation cohort, including accuracy, sensitivity, specificity, PPV, and NPV metrics. (CSV)

S4 Table Pairwise Cox proportional hazards model comparisons evaluating the incremental prognostic contribution of ALFAssay, Fragle, ichorCNA, and OncoFollow at baseline and Day 14. (CSV)

S5 Table Nested Cox model comparisons for pairwise combinations of ctDNA detection methods assessing complementary prognostic information for progression-free survival. (CSV)

S6 Table ROC-derived performance metrics and AUC values for ALFAssay, LASSO, Fragle, and composite ALFAssay+Fragle models using ichorCNA detection, OncoFollow detection, and progression-free survival classification as reference outcomes. (CSV)
