# Supplementary material for: ALFAssay: A feed‑forward neural network for quantitative fragmentomics‑based ctDNA profiling in breast cancer
Source: PLoS Comput Biol. 2026 Jul 27;22(7):e1014505. doi: 10.1371/journal.pcbi.1014505 (PMC13450836; doi:10.1371/journal.pcbi.1014505)
Supplement: Supplementary file 1 [file pcbi.1014505.s002.pdf]

Federal agency for medicines and health products
Eurostation II - Place Victor Horta 40/40
1060 Bruxelles
www.afmps.be

Iris Zwaenepoel
Tel. : + 32 (0)2 528 42 08
Fax : + 32 (0)2 528 40 01
e-mail : iris.zwaenepoel@fagg.be

Institut Jules Bordet
Frederic Henot
Boulevard de Waterloo 121
1000 Brussels

Your letter from	Your reference	Our reference	Annex	Dat
		FAGG/R&D/ISZ/kvk /98100 9		15 MARS 2017

Onderwerp Goedkeuring van een klinische proef op 15 februari 2017.
Titre de l'objet Approbation d'un essai clinique le 15 février 2017.
Subject Authorisation of a clinical trial dated 15 February 2017.

Neoadjuvant Biomarker ResearcH Study of Palbociclib Combined with Endocrine Therapy in Estrogen Receptor Positive/ HER2 Negative Breast Cancer

EudraCT : 2016-000879-24

Chère Madame, Cher Monsieur,

Conformément à l'article 12 de la Loi du 7 mai 2004 relative aux expérimentations sur la personne humaine, j'ai décidé d'autoriser l'essai clinique ci-dessus mentionné.

Salutations sincères,

Pour la Ministre des Affaires sociales et de la Santé publique

Geachte Mevrouw, Geachte Heer,

In overeenstemming met artikel 12 van de wet van 7 mei 2004 inzake experimenten op de menselijke persoon, heb ik besloten de hierboven vermelde klinische proef goed te keuren.

Met de meeste hoogachting,

Voor de Minister van Sociale zaken en volksgezondheid

Dr. Greet Musch

Unofficial translation:

In accordance with article 12 of the Law of 7 May 2004 concerning experiments on the human person, I have decided to authorise the above mentioned clinical trial.

Comité
d'Ethique

Tél 02/541.35.95

Email
Comite.ethique@
bordet.be

Président
Dr. D. Lossignol
Vice-Président
Dr. D. Bron
Secrétaire académique
Mme M. Paesmans
Membres
Dr. H. Bleiberg
Dr. L. Dal Lago
Dr. Gil
Dr. J. Klastersky
Dr. L. Moretti
Dr. L. Polastro
Dr. Th. Renard
M. P. Crombez
Mme B. Fernez
Mme M. Colin
(suppléante : Mme N. De Loore)
Mme D. van Vyve
(suppléantes :
Mme S. Greco,
Mme B. de Brabant)
Mme C. Deliens
(suppléante :
Mme M. Nevraumont)
Mme F. Lewis

OPINION OF THE ETHICS COMMITTEE OF THE INSTITUT JULES BORDET

Rue Héger Bordet, 1

1000 Brussels

Belgium

Accreditation number OM011

To the attention of Dr. Ignatiadis

Brussels, 23/03/2017

Eudract : 2016-000879-24

Internal reference : CE2520

National Coordinator : Dr. Ignatiadis

Principal investigators : les Docteurs Vuysteke (Ste Elisabeth Namur), Efira (CHU Brugmann)

Study title

NEORHEA IJB-BC-NEORHEA-2016 Neoadjuvant Biomarker ResearcH Study of Palbociclib Combined with Endocrine Therapy in Estrogen Receptor Positive/ HER2 Negative Breast Cancer

Supportive documentation

Internal Submission Form dd 11/01/2017
Cover letter dd 11/01/2017
Reply letter dd 23/02/2017
Clinical Trials Application form (with signature page) dd 11/01/2017
Insurance Certificate dd 11/01/2017
List of Participating Sites
CV of Coordinating Investigator and Principal Investigators of sites
Site Template Agreement
Synopsis v 1 (French)
Protocol v 1.0 dd 05/01/2017
ICF v 1.1 dd 22/02/2017 (French, Dutch, English)
Patient Diary v 1.1 dd 22/02/2017 Palbociclib/Tamoxifen (French, Dutch, English)
Patient Diary v 1.1 dd 22/02/2017 Palbociclib/Letrozole (French, Dutch, English)
Investigator Brochure Palbociclib dd 12/2015
Investigator Brochure Annual Review Memo Palbociclib dd 19/12/2016
SmPC Palbociclib last update dd 25/11/2016
SmPC Letrozole last update dd 12/11/2015
SmPC Tamoxifen last update dd 08/10/2015
SmPC Goserelin last update dd 09/06/2015

Dear Dr. Ignatiadis,

As Chair of the Ethics Committee of the Institut Jules Bordet, acting as Ethics Committee Providing Single Opinion, I am pleased to notify the opinion on the above referenced study and supportive documentation as listed above, which was discussed during the meeting held on 23/03/2017 (date of opinion):

Final Single Favourable Opinion

Kindly note that this opinion only applies to the French version of the Informed Consent Form and other patient documents.

I remind you that no deviations from, or changes of, the protocol should be initiated without the prior written approval of the Ethics Committee of an appropriate amendment, except when necessary to eliminate immediate hazards to the subjects or when the change(s) involves only logistical or administrative aspects of the study.

Hence, as principal investigator, you should promptly report to the Ethics Committee:

- Deviations from, or changes of, the protocol to eliminate immediate hazards to the subjects;
- Changes increasing the risk to subjects and/or affecting significantly the conduct of the study;
- All Suspected Unexpected Serious Adverse Reactions occurred in Belgium;
- New information that may affect adversely the safety of the subjects or the conduct of the study.

I hereby certify that the Ethics Committee operates according to ICH Good Clinical Practices and applicable local laws and regulations.

Signature of the Chair of the Ethics Committee
Name : DR. D. LOSSIGNOL

Appendix 1 - List of the Ethics Committee's members participating to the meeting of 23/03/2017

			Attendance
DR. D. LOSSIGNOL	Chairman	Physician	Yes
DR. D. BRON	Vice Chairman	Physician	No (*)
M. PAESMANS	Secretary Statistician	Physician	Yes
DR. H. BLEIBERG	External member	Physician	Yes
DR. L. DAL LAGO	Member	Physician	Yes
DR. TH. GIL	Member	Physician	Yes
DR. J. KLASTERSKY	Member	Physician	Yes
DR. L. MORETTI	Member	Physician	Yes
DR. L. POLASTRO	Member	Physician	Yes
DR. TH. RENARD	External member	General Practitioner	Yes
P. CROMBEZ	Member	Head Nurse	Yes
B. FERNEZ	Member	Head Nurse	Yes
M. COLIN	External member	Nurse / Logopedist	No
D. VAN VYVE	Member	Lawyer	Yes
S. GRECO	Member	Lawyer	No
B. DE BRABANT	Member	Lawyer	Yes
N. DE LOORE	External member	Lay person	No (*)
C. DELIENS	Member	Pharmacist	Yes
M. NEVRAUMONT	Member	Pharmacist	No
F. LEWIS	Member	Psychologist	Yes

(*) comments transmitted before the meeting

Notification of conflict of interest

Mrs Paesmans, van Vyve and de Brabant were not allowed to participate neither in the discussion nor in the voting.

Comité
d'Ethique

Tél 02/541.35.95

Email
Comite.ethique@
bordet.be

Président
Mme M. Paesmans
Vice-Président
Dr. L. Moretti
Secrétaire académique
Mme B. de Brabant
Membres
Dr. P. Aftimos
Dr. H. Bleiberg
Dr. D. Bron
Dr. L. Dal Lago
Dr. A. Ferster
Dr. Th. Gil
Dr. L. Polastro
Dr. Th. Renard
Mme L. Ameye
M. P. Crombez
M. F. Hoerner
Mme B. Cuylits
Mme B. de Brabant
Mme C. Deliens
M. R. Gioffredi
Mme S. Greco
Mme F. Lewis
M. P. Miqueu

OPINION OF THE ETHICS COMMITTEE
OF THE INSTITUT JULES BORDET – ECPSO/LEC
Accreditation number OM011

To the attention of Dr. Ignatiadis
Institut Jules Bordet

Brussels, 15/11/2021

BECT: B0792021000010

Internal reference: CE3369

Sponsor: Bordet

Principal investigator: Dr. IGNATIADIS

Study title

Fragmentation patterns of plasma cell-free DNA for ctDNA detection and quantification

Supportive documentation

- Cover letter dd 14/09/2021
- **Reply email dd 15/10/2021**
- Internal Submission Form dd 14/09/2021
- CPC approval letter dd 30/07/2021
- Insurance Certificate dd 21/09/2021 (Expiry dd 14/04/2025)
- CV Principal Investiigator : Dr. Ignatiadis
- CV Co-investigator : Dr. Gombos
- **Protocol v1.2 dd 15/10/2021**
- **Protocol summary v1.2 dd 15/10/2021**
- **ICF cfDNA Healthy v1.1 dd 15/10/2021 (French)**

Dear Dr. Ignatiadis,

As Chair of the Ethics Committee of the Institut Jules Bordet, I am pleased to notify the opinion on the above referenced study and supportive documentation as listed above, which was discussed during the meeting held on 23/09/2021 and on **04/11/2021** (date of opinion):

Final Single Favourable Opinion

The committee emphasizes that it is the responsibility of the sponsor to guarantee the conformity of informed consent forms in French and Dutch. We consider also it is the sponsor's responsibility to provide all the ethics committees with the final French and Dutch versions of the patient information sheet and informed consent form approved in the present letter.

I remind you that no deviations from, or changes of, the protocol should be initiated without the prior written approval of the Ethics Committee of an appropriate amendment, except when necessary to eliminate immediate hazards to the subjects or when the change(s) involves only logistical or administrative aspects of the study.

Hence, as principal investigator, you should promptly report to the Ethics Committee:

- Deviations from, or changes of, the protocol to eliminate immediate hazards to the subjects;
- Changes increasing the risk to subjects and/or affecting significantly the conduct of the study;
- All Suspected Unexpected Serious Adverse Reactions occurred in Belgium;
- New information that may affect adversely the safety of the subjects or the conduct of the study.

The Ethics Committee would like to remind you that ULB's Code of Ethics and Scientific Integrity in Research is available on ENNOV at the following link: http://vmappennov.bordet.be:8080/ennov/psprod/document/1368242

I hereby certify that the Ethics Committee operates according to ICH Good Clinical Practices and applicable local laws and regulations.

Signature of the Chair of the Ethics Committee

Name: Mrs. M. Paesmans

Appendix 1 - List of the Ethics Committee's members participating in the meeting on 04/11/2021

CE3369

			Attendance
Chairman, Statistician	Marianne PAESMANS	Member	No*
Vice-Chairman, Physician	Luigi MORETTI	Member	Yes
Academic Secretary Lawyer	Bénédicte de BRABANT	Member	Yes
Physicians	Harry BLEIBERG	External member	Yes
	Dominique BRON	Member	No*
	Lissandra DAL LAGO	Member	No*
	Thierry GIL	Member	No
	Laura POLASTRO	Member	No
	Philippe AFTIMOS	Member	No
General Practitioner	Thierry RENARD	External member	No
Physician Pediatrician	Alice FERSTER	External member,	No
Pharmacist	Coralie DELIENS	Member	No
Nurse	Patrick CROMBEZ	Member	No
	Frédéric HOERNER	External Member	No*
Psychologist	Florence LEWIS	Member	No
Lawyer	Bénédicte CUYLITS	Deputy member	No
	Stephania GRECO	Deputy member	No*
Statistician	Lieveke AMEYE	Deputy member	No
Patients Representatives	Roger GIOFFREDI	External member	Yes
	Patrick MIQUEU	Member	No

* *Written comments received before the meeting*

sociation Hospitalière de Bruxelles
Centre des Tumeurs de l'ULB
Association régie par la loi du 8 juillet 1976
Rue Héger-Bordet, 1- 1000 BRUXELLES
Tél. : 02 541 31 11
DEXIA 091-0097278-16

Comité d'Ethique

Tél : 02/541.35.95
Email secrétariat :
Comite.ethique@bordet.be

Président
Dr. Th. Gil

Vice-Président
Dr. D. Bron

Secrétaire
Dr. D. Lossignol

Membres
Dr. H. Bleiberg
Dr. E. de Azambuja
Dr. J. Klastersky
Dr. M. Sosnowski
Dr. Th. Renard
M. P. Crombez
(suppléante : Mme B. Fernez)
Mme M. Colin
Mme M. Paesmans
Mme N. De Loore
Mme D. van Vyve
Mme S. Greco
Mme C. Deliens
Mme M. Nevraumont

LIST OF DOCUMENTS REVIEWED BY THE MEDICAL ETHICS COMMITTEE OF INSTITUT JULES BORDET - LEC

Réf. : as
Date : 05/03/2015
Eudract : 2012-004860-22
Intern number : 2186

Coordinating investigator : DR. GOMBOS

Principal investigators : les Docteurs Huizing (UZA), Richard (CHU Ambroise Paré Mons), Vuylsteke (St Elisabeth Namur), Duhoux (UCL), Neven (KUL)

Title

The PEARL study : Pet imaging as a biomarker of Everolimus Added value in hormone Refractory postmenopausaL women

Including

Cover letter dd 02/02/2015
SA form
SmPC Afinitor 2.5 mg tablets dd 28/11/2014

LEADING ETHICS COMMITTEE'S DECISION

X Approval
☐ **Approval with conditions**
☐ **Need for extra information**
☐ **Disapproval**

Signature of the Chairman of the Central Ethics Committee

Name : Dr. Th. GIL

Date : 05/03/2015

List of members Ethics Committee (OM011)
(from 21/11/2013)

Meeting dd 05/03/2015

CE2186

			Attendance
DR. TH. GIL	Chairman	Physician	Yes
DR. D. BRON	Vice Chairman	Physician	Yes
DR. D. LOSSIGNOL	Secretary	Physician	Yes
DR. H. BLEIBERG	External member	Physician	Yes
DR. E. DE AZAMBUJA	Member	Physician	Yes
DR. J. KLASTERSKY	Member	Physician	No
DR. M. SOSNOWSKI	Member	Physician	Yes
DR. TH. RENARD	External member	General Practitioner	No
P. CROMBEZ	Member	Head Nurse	Yes
B. FERNEZ	Member	Head Nurse	No
M. COLIN	External member	Nurse / Logopedist	Yes
M. PAESMANS	Member	Statistician	Yes
D. VAN VYVE	Member	Lawyer	Yes
S. GRECO	Member	Lawyer	Yes
N. DE LOORE	External member	Lay person	Yes
C. DELIENS	Member	Pharmacist	No
M. NEVRAUMONT	Member	Pharmacist	No

Dr. de Azambuja and Mrs. Paesmans were not allowed to participate neither in the discussion nor in the voting.

The Ethics Committee operates according to ICH Good Clinical Practice and local applicable regulations

EudraCT Number: 2016-000879-24
Sponsor Protocol Number: IJB-BC-NEORHEA-2016
ClinicalTrials.gov Number: NCT03065621

Neoadjuvant Biomarker **R**esearc**H** Study of Palbociclib combined with Endocrine Therapy in **E**strogen Receptor Positive/HER2 Negative Breast C**A**ncer

"NeoRHEA"

Study Chair

Michail Ignatiadis, MD, PhD

Co- Study Chair

Christos Sotiriou, MD, PhD

Sponsor:
Institut Jules Bordet
Rue Héger-Bordetstraat 1,
1000 Brussels, Belgium
clinical_trial_neorhea@bordet.be

Protocol version 4.0

Issue date: 22/02/2019

CONFIDENTIAL

Information and data included in this protocol contain trade secrets and privileged or confidential information which is the property of the Institut Jules Bordet. No person is authorized to make it public without the written permission of the Institut Jules Bordet

PROTOCOL APPROVAL – Signature Page

EudraCT Number: 2016-000879-24

Sponsor Protocol Number: IJB-BC-NEORHEA-2016

ClinicalTrials.gov Number: NCT03065621

The signature below constitutes the approval of this protocol and the attachments, and provides the necessary assurances that this study will be conducted according to all stipulations of the protocol, including all statements regarding confidentiality, and according to national, local legal and regulatory requirements and applicable European regulations and ICH GCP guideline.

Study Chair and National coordinator

Title and Name	Approval date	Signature
Michail Ignatiadis, MD, PhD		

Site Principal Investigator

Title and Name	Function	Approval date	Signature
	Site Principal Investigator		

DOCUMENT HISTORY

Version	Protocol version release date	Short description of modifications	Substantial amendment
1.0	05/01/2017	Initial release	□ Yes<br>□ No
2.0	21/04/2017	• Inclusion of the additional hematology tests on day 14 of cycle 1 and day 14 of cycle 2<br>• Added Secondary Objectives and Secondary Endpoints<br>• Prohibiting use of preparations containing St. John's wort during drug administration	☒ Yes<br>□ No
3.0	14/03/2018	• Clarification of Allred score: positive is consider ≥3<br>• Clarification of Inclusion Criteria 5<br>• Change of required tumour size from 2 cm to 15 mm<br>• Post treatment ultrasound changed from 7-10 days before surgery to up to 14 days before surgery.<br>• Accept pre-study biopsies if available in the correct quantity<br>• On study ultrasounds changed from 3D to 2D.	☒ Yes<br>□ No
4.0	22/02/2019	• Addition of two secondary objectives and endpoints.<br>• Addition of one exploratory objective and endpoint.<br>• Note to IC 4<br>• Addition to Efficacy assessment<br>• Interim analysis on first 65 patients.<br>• Addition of translational research assessments<br>• Addition of GDPR information	☒ Yes<br>□ No

TABLE OF CONTENTS

LIST OF ABBREVIATIONS 17
1. BACKGROUND AND SCIENTIFIC RATIONALE 19
1.1. Background 19
1.2. Potential Risks: 22
1.3. Potential Benefits: 23
2. OBJECTIVES AND ENDPOINTS OF THE STUDY 23
2.1. Objectives 23
2.1.1. Primary objective: 23
2.1.2. Secondary objectives: 23
2.2. Endpoints 24
2.2.1. Primary endpoint 24
2.2.2. Secondary endpoints 24
3. STUDY DESIGN 25
4. SELECTION OF SUBJECTS 26
4.1. Inclusion criteria 26
4.2. Exclusion criteria 28
4.3. Subject registration process 28
5. SUBJECT COMPLETION AND WITHDRAWAL 29
5.1. Study treatment completion and withdrawal 29
5.2. Study visits completion and withdrawal 29
5.3. Subject’s consent withdrawal 29
5.4. Lost to follow-up 30
6. STUDY TREATMENTS 30
6.1. Investigational Medicinal Product: Palbociclib 30
6.1.1. Drug information 30
6.1.1.1. Formulation 31
6.1.1.2. Therapeutic regimen and dose 31
6.1.1.3. Stability and storage 31
6.1.1.4. Labelling 31
6.1.1.5. Drug logistics and accountability 31
6.1.2. Toxicity 32
6.1.3. Toxicities management 32
6.1.4. Dose and schedule modifications due to toxicity 36
6.2. Investigational Medicinal Product: Letrozole 37

6.2.1. Drug information 37
6.2.1.1. Formulation 37
6.2.1.2. Therapeutic regimen and dose 37
6.2.1.3. Stability and storage 38
6.2.1.4. Labelling 38
6.2.1.5. Drug logistics and accountability 38
6.2.2. Toxicity 38
6.2.3. Toxicities management 38
6.2.4. Dose and schedule modifications due to toxicity 38
6.3. Investigational Medicinal Product: Goserelin 39
6.3.1. Drug information 39
6.3.1.1. Formulation 39
6.3.1.2. Therapeutic regimen and dose 39
6.3.1.3. Stability and storage 40
6.3.1.4. Labelling 40
6.3.1.5. Drug logistics and accountability 40
6.3.2. Toxicity 40
6.3.3. Toxicities management 40
6.3.4. Dose and schedule modifications due to toxicity 40
6.4. Investigational Medicinal Product: Tamoxifen 40
6.4.1. Drug information 40
6.4.1.1. Formulation 41
6.4.1.2. Therapeutic regimen and dose 41
6.4.1.3. Stability and storage 41
6.4.1.4. Labelling 41
6.4.1.5. Drug logistics and accountability 41
6.4.2. Toxicity 41
6.4.3. Toxicities management 42
6.4.4. Dose and schedule modifications due to toxicity 42
6.5. Protocol surgery 42
6.5.1. Surgical treatment 42
6.5.2. Surgery toxicity 42
6.6. Contraception and menopausal status 43
6.6.1. Contraception 43
6.6.2. Menopausal status 43

6.7. Concomitant treatments 43
6.7.1. Allowed Concomitant treatments 43
6.7.2. Prohibited Concomitant treatments 44
6.7.3. Incompatibilities/Drug Interactions 44
6.7.3.1. Palbociclib 44
6.7.3.2. Letrozole 45
6.7.3.3. Goserelin 45
6.7.3.4. Tamoxifen 45
6.8. Study Treatment duration...45
7. SCHEDULE OF ASSESSMENTS...46
7.1. Screening period 46
7.2. Pre-treatment period 47
7.3. Study treatment period 48
7.4. End of study treatment visit..49
7.5. Patient Diary 49
8. ASSESSMENT OF EFFICACY 51
8.1. Efficacy parameters.. 51
8.2. Methods for assessing efficacy parameters.. 51
8.3. Progression of disease during study 52
9. ASSESSMENT OF SAFETY...52
9.1. Safety parameters and Methods for assessing safety parameters 52
9.2. Pharmacovigilance parameters definitions and reporting guidance...............53
9.2.1. Adverse Events...53
9.2.1.1. Definitions 53
9.2.1.2. AE intensity 54
9.2.1.3. Relationship to study treatments 54
9.2.1.4. Reporting of adverse events 55
9.2.2. Serious Adverse Events..55
9.2.2.1. Definitions 55
9.2.2.2. Reporting of serious adverse events...56
9.2.3. Exceptions to AEs/SAEs reporting 58
9.2.4. Adverse Event of Special Interest 58
9.2.4.1. Definitions 58
9.2.4.2. Reporting of adverse events of special interest 59
9.2.5. Pregnancy 59

9.2.6. Exposure during lactation 60
9.2.7. Overdose, misuse and abuse 60
9.2.7.1. Definitions 60
9.2.7.2. Reporting of overdose, misuse and abuse 60
9.2.8. Medication Error 60
9.2.8.1. Definition 60
9.2.8.2. Reporting of medication error 61
9.2.9. Reporting of exposure to study medications by non-study subjects 61
10. TRANSLATIONAL RESEARCH(ES) 61
10.1. Timelines 64
10.2. Biological material handling 65
10.3. Central biobanking of biological material collected during the trial and Destruction 65
10.4. Future undefined research 66
11. STATISTICAL CONSIDERATIONS 66
11.1. Statistical design 66
11.1.1. Sample size 66
11.1.2. Study duration 67
11.2. Statistical plan 67
11.3. End of study 67
12. DIRECT ACCESS TO SOURCE DATA/DOCUMENTS 68
13. QUALITY CONTROL AND QUALITY ASSURANCE 68
13.1. Quality control 68
13.2. Quality assurance 68
14. REGULATORY AND ETHICAL CONSIDERATIONS 68
14.1. Regulatory considerations 68
14.2. Informed consent form 69
14.3. Subject identification ..... **Error! Bookmark not defined.**
15. DATA HANDLING AND RECORD KEEPING 74
15.1. Investigator's files and subject's clinical source documents 74
15.2. Case Report Forms (CRF) 74
15.3. Retention of documents 75
16. STUDY SPONSORSHIP AND FINANCING 76
17. STUDY INSURANCE 76
18. PUBLICATION POLICY 76
19. REFERENCES 77

SYNOPSIS

**TITLE**	**Neo**adjuvant Biomarker **R**esearc**H** Study of Palbociclib Combined with Endocrine Therapy in **E**strogen Receptor Positive/ HER2 Negative Breast C**A**ncer
**SHORT NAME**	NeoRHEA
**SPONSOR**	Institut Jules Bordet (IJB) - Brussels, Belgium
**INDICATION(S)**	Primary breast cancer
**TARGET STUDY POPULATION**	Pre or post-menopausal female subjects with ER-positive/HER2-negative, primary breast cancer, with 15 mm or more, determined by ultrasound and N0 or N1 disease, who are candidates for preoperative treatment for breast cancer.
**PHASE**	Phase 2
**STUDY DESIGN**	This is an open-label, single arm, phase 2 trial that will include pre or post-menopausal female subjects, that have ER-positive, HER2-negative early breast cancer. Subject will receive 4 cycles of palbociclib 125 mg (each cycle of palbociclib consists of treatment from D1 to D21 followed by a week of rest) combined with endocrine therapy given continuously (each cycle of endocrine therapy consists of treatment from D1 to D28).<br>The endocrine therapy will be determined according to the menopausal status (per protocol definition as per protocol section 5.6.2) of the subject evaluated at the study screening.<br>For post-menopausal subjects: endocrine therapy will consist of letrozole 2.5 mg continuously.<br>For pre-menopausal or peri-menopausal subjects: endocrine therapy will consist of tamoxifen 20 mg continuously, combined or not with goserelin 3.6 mg monthly at the local investigator's discretion.<br>Endocrine therapy for both groups should continue until the last day of palbociclib treatment (i.e. the day before surgery).<br>Subject's response to therapy will be evaluated before and after the 4 cycles of treatment by ultrasound to determine response to therapy. Post treatment ultrasound should be performed within a maximum of 14 days before surgery.<br>Surgery will be performed 4 to 8 days after the end of the 4th cycle of study treatment. Between the end of the 4th cycle and surgery, subjects will continue to receive palbociclib and endocrine therapy, at the same doses for a maximum of 7 additional treatment days, with the last dose of endocrine therapy and palbociclib administered the day before surgery. Surgery should be performed no later than 4 months plus 8 days from beginning of study treatment. Should any adaptations to the schedule of palbociclib be necessary in order for the surgery to occur within this timeframe, they can be decided by the local investigator as long as subjects receive at least 3 days of endocrine therapy and palbociclib before surgery.

<table>
<tr>
<td></td>
<td>
Biopsy samples will be collected after the subject’s inclusion in the study and prior to Day 1 Cycle 1 of study treatment (pre-treatment biopsy) and will consist of collection of 4 core biopsies (2 FFPE and 2 Frozen). If 4 biopsies are already available for submission(2 FFPE and 2 Frozen), which were obtained outside the study due to the Site’s standard clinical practice, there is no need to repeat these biopsies after the subject’s inclusion in the study. These biopsies must have been obtained up to 6 weeks before the beginning of treatment.
FFPE and frozen material will be collected from the surgical material left over, after sufficient and relevant parts has been retained to establish, improve or complement the diagnosis or treatment of the subject.
One whole blood sample (1x10 mL) will also be collected after the subject’s inclusion and prior to Day 1 Cycle 1 of study treatment.
Blood samples for plasma processing (4x10 mL per time point) will be collected after the subject’s inclusion and prior to Day 1 Cycle 1, prior to Day 1 Cycle 2 of study treatment, at surgery day (prior to surgery) and one month after surgery.
All the biological samples collected (pre-treatment biopsy, material from surgery and all blood samples) within the study are mandatory. The imaging tumour assessment (ultrasound breast) performed prior to the signature of the study ICF is not admissible for assessment of study end-points and should be repeated within the required study time window.

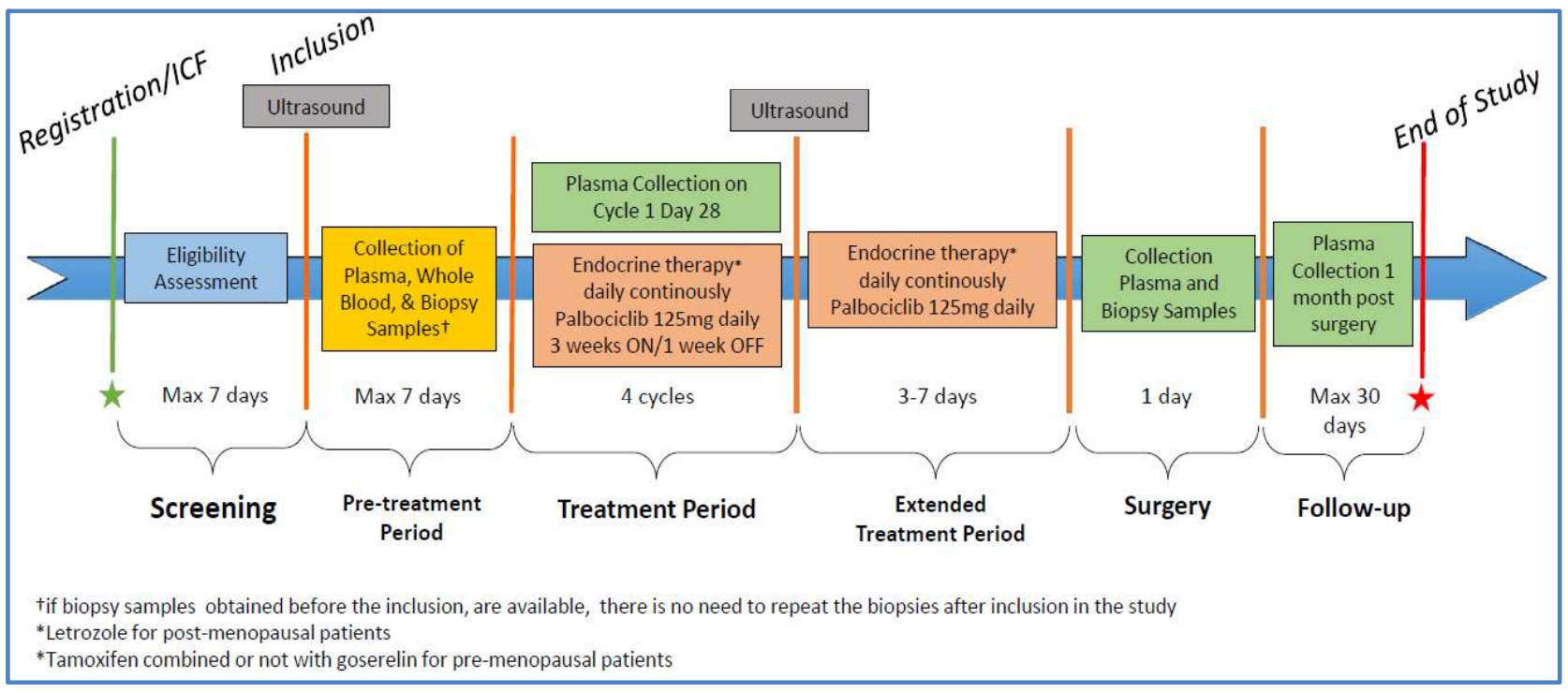

</td>
</tr>
<tr>
<td><u>STUDY RATIONAL</u></td>
<td>
Palbociclib is a CDK4 and CDK6 inhibitor that received accelerated approval by the FDA to be used in combination with endocrine therapy in metastatic ER+/HER2- breast cancer based on the results of the PALOMA 1 trial [(1)]. In this phase 2 trial, palbociclib significantly improved median progression-free survival (PFS) when added to letrozole in previously untreated women with estrogen receptor positive, HER2-negative breast cancer. The PALOMA-2 results, recently presented at ASCO confirm this PFs benefit. OS data is eagerly anticipated [(2)]. Moreover, in the PALOMA 3 trial, palbociclib improved PFS when added to fulvestrant in subjects with hormone receptor positive metastatic breast cancer who progressed on previous endocrine treatment [(3)]. In both trials toxicity was significantly more frequent in the palbociclib arm, with neutropenia being of special concern, even if few subjects had infections.
The role of palbociclib in different settings of breast cancer treatment is being explored in several trials, such as the PALLAS trial (NCT02513394) and the PENELOPE-B trial (NCT01864746), and it is likely that its use will be expanded in the coming years. Biomarkers
</td>
</tr>
</table>

	that help us predict response to palbociclib may lead to better subject selection, and thus avoid unnecessary toxicity and reduce costs.<br><br>In the present study, we will use RNA-sequencing of the baseline tumour biopsy to identify biomarkers of resistance to a 4 month preoperative treatment of palbociclib and endocrine therapy given prior to surgery. Resistance will be defined as lack of response (stable disease) at ultrasound after the 4-month treatment period, as well as progression anytime during the 4-month treatment period by ultrasound (response evaluation will be defined according to WHO criteria [(4)]). Resistance will be also defined as residual cancer burden (RCB) of 3 that has been associated with poor outcome after neoadjuvant treatment [(5)] or high tumour proliferation as evaluated by Genomic Grade Index (GGI) from RNA-seq data at surgery [(6,7)].<br><br>Recently, it has been demonstrated in breast cancer cell lines that the CDK4 T172 phosphorylation is associated with sensitivity to palbociclib and a signature of an 11-gene expression has been developed that can faithfully predict the CDK4 modification profile in breast tumours and breast cancer cell lines (Raspe et al Under Review). This 11-gene expression signature has been associated with sensitivity to CDK 4/6 inhibitors e.g. palbociclib in breast cancer cell lines (Patent : Method For determining sensitivity to a CDK4/6 inhibitor, filed the 18.05.16, European Patent Application No/patent No EP16170146.1-1403, Applicant : Université Libre de Bruxelles, Belgium. Unpublished; private communication).
**<u>OBJECTIVES</u>**	Primary objective:<br>• To identify biomarkers of resistance to a 4-month preoperative treatment of palbociclib plus endocrine therapy defined as stable or progressive disease by ultrasound (based on WHO criteria) using RNA-seq of the baseline tumour biopsy.<br><br>Secondary objectives:<br>• To identify biomarkers of resistance to a 4-month preoperative treatment of endocrine therapy and palbociclib by correlating tumour response by ultrasound (mandatory) or magnetic resonance (optional) imaging (response will be assessed as continuous or categorical variable) with RNA-seq and whole genome sequencing (WGS) of the baseline tumour biopsy<br>• To identify biomarkers of resistance to a 4-month preoperative treatment of palbociclib plus endocrine therapy defined as residual disease burden, RCB of 3 using RNA-seq and WGS of the baseline tumour biopsy<br>• To identify biomarkers of resistance to a 4-month preoperative treatment of endocrine therapy and palbociclib defined as GGI high by RNA-seq of the left over tumour tissue at surgery using RNA-seq and WGS of the baseline tumour biopsy<br>• To identify genomic and transcriptomic biomarkers of resistance to a 4-month preoperative treatment of palbociclib plus endocrine therapy defined as absence of complete cell cycle arrest (CCA, centrally reviewed Ki67 ≤2.7%) at surgery.<br>• To understand mechanisms of resistance to the combination of endocrine therapy and palbociclib by comparing the genome and transcriptome of tumours at baseline and at surgery using WGS and RNA-seq<br>• To evaluate the safety of the combination of palbociclib plus

	endocrine therapy<br>• To evaluate the role of plasma ctDNA in monitoring response/resistance to pre-operative treatment with endocrine therapy and palbociclib<br>• To validate/further refine an 11-gene expression signature associated with response/resistance to palbociclib and endocrine treatment<br>• To determine the effect of a pre-operative treatment with endocrine therapy and palbociclib on anti-tumour immune response<br>• To determine the effect of a pre-operative treatment with endocrine therapy and palbociclib on tumour senescence.<br>• To determine the effect of a pre-operative treatment with endocrine therapy and palbociclib on tumour proliferation, as measured by the Ki67 score.<br>Exploratory objectives:<br>• To compare changes in clonal composition, transcriptomic changes and changes in the open chromatin state of tumour cells using a combined genomic and transcriptomic (G&T) single tumour cell analysis and chromatin accessibility single tumour cell analysis of exceptional responders and exceptional non-responders. Based on the above results, additional bulk or single cell analyses in the entire study cohort could be performed.
**ENDPOINTS**	Primary endpoint:<br>• Baseline transcriptomic profile of resistance to 4 months of palbociclib and endocrine therapy defined as stable or progressive disease by ultrasound based on WHO criteria<br>Secondary endpoints:<br>• Baseline genomic and transcriptomic profile of resistance to 4 months of palbociclib and endocrine therapy assessed by ultrasound (mandatory) or magnetic resonance (optional) imaging (response will be assessed as continuous or categorical variable)<br>• Baseline genomic and transcriptomic profile of resistance to 4 months of palbociclib and endocrine therapy, defined as an RCB of 3.<br>• Baseline genomic and transcriptomic profile of resistance to 4 months of palbociclib and endocrine therapy, defined as an GGI high at surgery<br>• Baseline genomic and transcriptomic profile of resistance to 4 months of palbociclib and endocrine therapy, defined as absence of CCA at surgery<br>• Genomic and transcriptomic changes between pre-treatment and post-treatment tumour samples<br>• Safety<br>• Plasma ctDNA analysis to monitor response/resistance to pre-operative treatment with endocrine therapy and palbociclib<br>• Validation of 11-gene expression signature associated with response/resistance to palbociclib and endocrine treatment<br>• Changes in anti-tumour immune response between pre- and post-treatment tumour samples<br>• Changes in tumour senescence between pre- and post-treatment tumour samples

	• Changes in Ki67 scores between pre- and post-treatment tumour samples.<br>Exploratory endpoints:<br>• Changes in clonal composition, transcriptomic changes and changes in the open chromatin state of tumour cells using a combined genomic and transcriptomic (G&T) single tumour cell analysis and chromatin accessibility single tumour cell analysis of exceptional responders and exceptional non-responders. Depending on the results of these analyses, additional bulk or single cell analyses in the entire study cohort could be performed.
**NUMBER OF SUBJECTS**	• Estimated number of subjects to screen: 110 subjects<br>✓ Estimated number of subjects <64 y.o. :70<br>✓ Estimated number of subjects >64 y.o. : 40<br>• Estimated number of subjects to include: 100<br>• Number of evaluable subjects: 90 (we anticipate that 10 subjects may not be evaluable for the analysis of the primary objective due to failure to generate RNA-seq data or due to other reasons, e.g. subject compliance)
**INCLUSION CRITERIA**	Subjects will only be eligible for study participation if they meet all the following inclusion criteria:<br>1. Female<br>2. Age ≥ 18 years<br>3. Histological diagnosis of breast adenocarcinoma that is estrogen receptor-positive, and HER2- negative as per the updated American Society of Clinical Oncology (ASCO) - College of American Pathologists (CAP) guidelines according to local testing.<br>4. Multifocal unilateral or bilateral breast adenocarcinoma tumours are allowed provided that all tested foci are ER-positive and HER2-negative.<br>- ER-positive (ER+ is defined as having an IHC of 1% or more and/or an Allred of 3 or more and HER2-negative).<br>- HER2 negative (HER2 negative is defined as having an IHC of 0 or 1+ without ISH OR IHC 2+ and ISH non-amplified with ratio less than 2.0 and if reported, average HER2 copy number < 4 signals/cells OR ISH non-amplified with ratio less than 2.0 and if reported, average HER2 copy number < 4 signals/cells [without IHC]);<br>o Note: a IHC of 3+ is always considered HER2 positive, independently of the ISH result.<br>o Note: in discrepant ISH cases (e.g. ratio >2.0 and average HER2 copy number < 4 signals/cells), ASCO/CAP guidelines should be followed and tumours may still be considered HER2-negative, if concordant with the ASCO/CAP guidelines.<br>5. A primary non metastatic or locally advanced tumour of ≥15 mm or more, determined by ultrasound, N0 or N1 without prior treatment candidate for preoperative treatment<br>6. ECOG Performance Status (PS) 0 or 1.<br>7. Adequate Bone Marrow Function including:

	a. Absolute Neutrophil Count (ANC) ≥1500/μL or ≥1.5 x109/L;<br>b. Platelets ≥100000/μL or ≥100 x 109/L;<br>c. Hemoglobin ≥ 9 g/dL.<br>8. Adequate Renal Function including: Serum creatinine ≤ 1.5 x upper limit of normal (ULN) or estimated creatinine clearance ≥ 60 ml/min as calculated using the method standard for the institution.<br>9. Adequate Liver Function, including all of the following parameters:<br>a. Total serum bilirubin ≤ 1.0 x ULN unless the subject has documented Gilbert syndrome (in which case up to 3 x ULN is acceptable) ;<br>b. Aspartate and Alanine Aminotransferase (AST and ALT) ≤ 1.5 x ULN;<br>c. Alkaline phosphatase ≤ 2.5 x ULN.<br>10. Signed consent form<br>11. Willingness and ability to comply with the study scheduled visits, treatment plans, laboratory tests, radiological exams, tumour and blood specimen collection and other procedures.<br>12. Women who are not postmenopausal or have not undergone hysterectomy must have documented negative pregnancy test (serum) prior to inclusion.<br>13. Female subjects of child bearing potential and their partners, who are sexually active, must agree to the use of one highly effective form of contraception throughout the period of taking study treatment and for at least 90 days after last dose of study drug, or they must totally/truly abstain from any form of sexual intercourse. Use of oral hormonal contraceptive agents in this study is not permitted.
**EXCLUSION CRITERIA**	Participants who exhibit any of the following conditions at screening will not be eligible for admission into the study.<br>1. Clinical T4 disease including inflammatory breast cancer.<br>2. Prior history of invasive cancer including breast cancer except basal or squamous cell carcinoma of skin that has been definitively treated.<br>3. Known hypersensitivity to the study drugs or excipients.<br>4. Any illness or medical condition that is unstable or could jeopardize the safety of the subject or her compliance with study requirements.<br>5. Subjects unable to swallow oral medications.<br>6. Prior intake of letrozole, tamoxifen, or any CDK inhibitor or anti-cancer therapy.<br>7. Concurrent treatment with any of the drugs not permitted, i.e. strong CYP3A inhibitors/inducers and drugs known to cause QTc interval prolongation (see section 6.7 for specific instructions).<br>8. QTc exceeding 480 msec, family or personal history of long or short QT syndrome, Brugada syndrome or known history of QTc prolongation, or Torsade de Pointes (TdP).<br>9. Uncontrolled diabetes, according to investigator's clinical judgment.<br>10. Pregnant or lactating women.

**PARTICIPATING COUNTRY(-IES)**	Belgium
**INVESTIGATIONAL MEDICINAL PRODUCT(S) DOSE/ROUTE/REGIMEN**	- All subjects will receive palbociclib 125 mg for 4 cycles, orally, once a day, for 21 days followed by 7 days of rest (4 cycles of 28 day long). Each cycle begins (D1) at the first day of medication use, and finishes at the last day of medication rest (D28). After the final rest week (therefore post cycle 4), subjects will receive 3 to 7 additional days of palbociclib, as necessary, at the same dose and posology, until the day before curative intent surgery.<br>- Post-menopausal subjects will receive letrozole 2.5 mg continuously during palbociclib treatment (which consists of 4 cycles of 28 days), with an added 3 to 7 days as necessary, orally once a day until the day before curative intent surgery. There are no rest periods for letrozole (unless if toxicity occurs).<br>- Pre-menopausal subjects will receive tamoxifen 20 mg continuously during palbociclib treatment (which consists of 4 cycles of 28 days), with an added 3 to 7 days as necessary, orally once a day until the day before curative intent surgery. They may also receive goserelin, at the local investigator's discretion, 3.6 mg every 4 weeks during the same period. There are no rest periods for this regimen (unless if toxicity occurs).<br><br>In case of delays due to toxicity, the surgery should be performed no later than 4 months + 8 days from beginning of study treatment. Should any adaptations to palbociclib schedule be necessary due to toxicity or other reasons, these adaptations are left at the investigator's discretion, as long as the subject receives palbociclib for 3 days before surgery.<br><br>The last dose of endocrine therapy and palbociclib will be given the day (evening) before surgery.
**NON-MEDICINAL STUDY TREATMENT(S)**	Curative breast surgery to be performed according to local guidelines, 4 to 8 days after the end of fourth cycle of study treatment and no later than 4 months + 8 days from the beginning of study treatment.
**ASSESSMENT OF EFFICACY**	Primary endpoint assessment:<br><br>Subjects will be staged according to ultrasound before receiving study treatment. Recording of at least 2 dimensions (longest diameter x greatest perpendicular diameter) of the primary tumour will be performed and presence, number and size (not mandatory) of lymph nodes will be evaluated. After 4 cycles of treatment the same measurements will be taken, to establish whether the subject has responded, remained stable or progressed according to WHO criteria.<br><br>Secondary endpoints:<br><br>Subjects will be staged according to ultrasound (mandatory) and MRI (as additional option in case mandated by local guidelines before receiving study treatment and highly recommended). Recording of at least 2 dimensions (longest diameter x greatest perpendicular diameter) of the primary tumour will be performed and presence, number and size (not mandatory) of lymph nodes will be evaluated. After 4 cycles of treatment the same measurements will be taken.

	In order to calculate the RCB, the surgical material will be used and analysed according to the following parameters<br>1. Primary tumour bed dimensions<br>2. Cellularity fraction of invasive cancer<br>3. Size of largest positive lymph node<br>4. Number of positive lymph nodes<br>GGI is composed of 97 genes [6]. We will analyse pre and post-surgery left over tumour tissue via RNA-seq to determine the expression of the 97 genes and calculate the GGI. In order to evaluate the achievement of complete cell cycle arrest (CCA), Ki67 score will be centrally reviewed on residual tumour at surgery.
**ASSESSMENT OF SAFETY**	Clinical and laboratory adverse events (AEs) and serious adverse events (SAEs) will be graded according to the National Cancer Institute Common Terminology Criteria for Adverse Events (NCI-CTCAE) version 4.03.<br>Potential cases of drug-induced liver injury (potential Hy's law cases) are considered as adverse events of special interest.
**STATISTICAL ANALYSES**	The study primary objective is to develop a gene signature to predict resistance to neoadjuvant treatment of endocrine therapy and palbociclib using RNA sequencing of the baseline tumour biopsy. The following few assumptions are available:<br>- The overall response rate will be around 75%-80% [8]<br>- The resistance rate is therefore around 20%-25%<br>According to simulations carried out by Dobbin and Simon a sample size of at least 20 subjects in each class is required to develop a binary predictor [9].<br>Therefore, to get 20 subjects in the resistant group with an expected 22.5% resistance rate, around 90 evaluable subjects are needed.<br>To take into account a rate of 10% of non-evaluable subjects, 100 eligible subjects will be required for inclusion in the study.<br>We will perform an interim analysis on data from the first 65 enrolled patients (2/3 of the 100). The interim analysis will not focus on the primary objective but on selected secondary objectives and on the exploratory objective and will have no impact on the course of the trial.<br>The whole sample size will be used for predictor development. A further validation set will be required.
**TRANSLATIONAL RESEARCH(ES)**	All subjects will undergo a pre-treatment breast tumour biopsy (2 FFPE and 2 frozen cores). They must be obtained for all subjects after inclusion before start of the study treatment (Day 1 Cycle 1). However, if 4 samples are already available (2 FFPE and 2 Frozen), which were obtained before inclusion due to the Site's standard clinical practice, there is no need to repeat biopsies after the subject's inclusion in the study. These biopsies must have been obtained up to 6 weeks before the beginning of treatment.<br>In addition, one whole blood sample (1x10mL EDTA) will be collected after the study inclusion and before start of the study treatment (Day 1 Cycle 1) for isolation of normal DNA to exclude germline variants during our next-generation sequencing analyses.<br>Blood samples (4x10 mL EDTA per time point) will be also collected

	for plasma isolation after the study inclusion and prior to Day 1 Cycle 1, Day 1 Cycle 2, at the time of surgery and one month after surgery.<br><br>We will perform RNA sequencing on the pre-treatment frozen biopsy cores and surgical material for all the subjects.<br><br>RNA sequencing offers a more precise measurement of transcripts (including mRNA, non-coding RNAs, and small RNAs). Compared with microarrays it offers better quantification and larger dynamic range as it provides the absolute number of transcripts. Importantly, somatic mutations, gene fusions and chromosomal rearrangements can also be detected with this technology.<br><br>Batched RNA sequencing will be performed at the end of recruitment period on all subjects for whom the frozen biopsy cores and left over surgical material are evaluable in terms of tumour content and RNA quantity.<br><br>Due to the fast evolution within this field, the actual analytical methodology will only be specified and decided upon at the time these analyses will start.<br><br>Tumour derived DNA and Plasma ctDNA extractions will be performed at the end of recruitment period for all subjects.<br><br>Specific mutations genes will be identified using RNA-seq data as well as high coverage NGS in primary tumour DNA (pre-treatment biopsy). Mutations identified using this approach will be validated using ddPCR. Analysis of normal DNA using NGS will also be performed at the same time to exclude germline variants. Moreover mutation(s) (specific per subject) identified by NGS in the primary tumour will then be followed using ddPCR in plasma ctDNA. Analysis of normal DNA using ddPCR will also be performed at the same time.<br><br>We will also evaluate the possibility to detect point mutations directly in plasma ctDNA using high coverage NGS. Mutations identified using this approach will be validated using ddPCR.<br><br>Markers of senescence will also be investigated using IHCs on FFPE pre and post treatment tumour samples. Tumour proliferation, as measured by the Ki67 score, will be assessed using IHCs on FFPE pre and post treatment tumour samples.<br><br>As an exploratory objective, we will perform a combined genomic and transcriptomic (G&T) single tumor cell analysis and chromatin accessibility single tumor cell analysis of frozen tumour samples from exceptional responders and exceptional non-responders.
**LENGTH OF THE STUDY**	• Recruitment period: 33 months<br>• Treatment period for a subject: 4 months<br>• Follow-up period: 1 month<br>• Data Cleaning: 6 months<br>• Biological sample analysis starting at the end of recruitment period: 12 months
**END OF STUDY**	The end of study will be declared when all the following criteria will have been met:<br><br>• After last visit of the last subject (one month after the surgery day of the last subject)<br>• The trial is mature for the analysis of all the endpoints as defined in the protocol, if the trial reaches its endpoints<br>• The database has been fully cleaned and frozen for all analyses

LIST OF ABBREVIATIONS

Abbreviation	Description
ADL	Activities of Daily Living
AE	Adverse Event
AESI	Adverse Event of Special Interest
AI	Aromatase Inhibitor
ALT	Alanine aminotransferase
ANC	Absolute Neutrophil Count
AR	Adverse Reaction
ASCO	American Society of Clinical Oncology
AST	Aspartate aminotransferase
BC	Breast Cancer
CAP	College of American Pathologists
CDK	Cyclin-Dependent Kinase
(e)CRF	(Electronic) Case Report Form
ctDNA	circulating tumour Deoxyribonucleic Acid
ddPCR	droplet digital Polymerase Chain Reaction
ECOG	Eastern Cooperative Oncology Group
EOS	End Of Study
EOT	End Of Treatment
ER	Estrogen Receptor
EVCTM	EudraVigilance Clinical Trial Module
FFPE	Formalin-Fixed, Paraffin-Embedded
FSH	Follicle Stimulating Hormone
GGI	Genomic Grade Index
GMP	Good Manufacturing Practice
GP	General Practitioner
IB	Investigators Brochure
ICH-GCP	International Conference on Harmonisation – Good Clinical Practice
ICF	Informed Consent Form
IEC	Independent Ethical Committee
IJB	Institut Jules Bordet
IMP	Investigational Medicinal Product
IRB	Institutional Review Board

ISF	Investigator Site File
LODE	Lack of Drug Effect
MDT	Multi-Disciplinary Team
MedDRA	Medical Dictionary for Regulatory Activity
NCI-CTCAE	National Cancer Institute - Common Terminology Criteria for Adverse Events
NIMP	Non-Investigational Medicinal Product
NGS	Next Generation Sequencing
PEPI	Preoperative Endocrine Prognostic Index
PFS	Progression-Free Survival
PS	Performance Status
RCB	Residual Cancer Burden
RECIST	Response Evaluation Criteria In Solid Tumours
SAE	Serious Adverse Event
SAR	Serious Adverse Reaction
SmPC	Summary of Product Characteristics
SOC	System Organ Class
SUSAR	Suspected Unexpected Serious Adverse Reaction
TdP	Torsade de Pointes
UAR	Unexpected Adverse Reaction
ULN	Upper Limit Normal

1. BACKGROUND AND SCIENTIFIC RATIONALE

1.1. Background

Breast cancer (BC) is the most common type of cancer as well as the first cause of cancer death among women worldwide, with an estimated 1.7 million new cases and 521,900 deaths in 2012 [10]. In developed countries, after decades of increase in incidence rates, the last 15 years have seen a decrease, likely due to reduced use of postmenopausal hormone supplements [11]. Mortality in developed nations has steadily declined and is now at the lowest rates in the available historical series [11]. The causes of this decline are still under debate, but it is reasonable to ascribe it to a combination of improvements in treatment options and screening programmes (10–12). In developing nations, however, both incidence and mortality are still on the rise [13]. In the coming years, the increase in the elderly population and the rising costs of treatment are two major challenges to be faced in breast cancer treatment [14,15]. The way forward in facing these challenges certainly is better matching of subject and treatment through new and improved biomarkers that can predict the benefit subjects will derive from treatment.

Traditional forms of anti-cancer treatment, including surgery, radiotherapy, chemotherapy and hormonotherapy have been in development for over 150 years [16]. Hormonotherapy, specifically, has led to major clinical gains in both advanced and early disease, starting in the 19th century with oophorectomy [16], followed by the development of tamoxifen, to the more recent development of new classes of anti-hormonal agents such as aromatase inhibitors (anastrozole, letrozole, examestane) and estrogenic receptor degraders (fulvestrant) (17–19). Besides the improvement of results, hormonotherapy is regarded as having a more tolerable toxicity profile when compared to other forms of treatment.

Tamoxifen is a selective estrogen receptor modulator (SERM), that has been in use since approval in the 1970s in multiple settings. It acts by competitively inhibition of the binding of estrogen to ER [20]. Though surpassed as standard by other newer agents it remains the gold standard in adjuvant treatment of pre-menopausal women, alone or with goserelin [19,21,22]. Goserelin is a gonadotropin-releasing hormone (GNRH) agonist that leads to a long term blockade of the receptors and thus induces menopause in women. It has been in use in the advanced setting in BC and in the adjuvant setting for pre-menopausal after the results of the SOFT trial [19,21,23].

Aromatase inhibitors (AI) have been tested in the early setting, both in adjuvant and neoadjuvant trials with positive results [24,25]. They act by radically diminishing estrogen levels in post-menopausal agents, since estrogen production relies almost exclusively on conversion of androgens into estrogens by aromatase in this population [26].

The neoadjuvant setting has been the target of increasing interest recently, as it offers the possibility of direct evaluation of treatment effect on tumour size, better surgical results as well as for the possible research opportunities it provides via the comparative analysis of tumour biology and clinical outcomes before and after treatment [27,28]. This has led to the development of potential surrogate end points for long-term clinical outcome in women receiving preoperative endocrine treatment, such as the day 15 Ki67 (a detectable protein that correlates to cell proliferation) and the PEPI score that is evaluated at surgery. The PEPI (preoperative endocrine prognostic index) is a score that includes tumour stage, lymph node

stage, ER status according to Allred score and Ki67 status measured on the surgical specimen obtained post endocrine neoadjuvant therapy [29,30]. Both endpoints have been associated with event free survival in this setting. Historically, neoadjuvant hormonotherapy was first studied in fragile populations such as elderly women with impressive results, including complete responses and high overall survival rates [28]. AIs, tamoxifen and goserelin have been tested in this setting in multiple trials (18,25,28,30–33). Eiermann and colleagues compared tamoxifen and letrozole for 4 months in 337 women in a randomized study in which letrozole proved to be superior (55% versus 36% overall response rate, respectively) [34]. On the other hand, both the IMPACT study and the PROACT study comparing tamoxifen to anastrozole did not find a clear advantage for the AI arm [33,35]. A meta-analysis of these trials, with a total of 1,160 subjects favours the use of AI as standard neoadjuvant hormonotherapy in post-menopausal subjects [36]. Even so, considering the results in the adjuvant setting, tamoxifen and goserelin are also a valid therapeutic option in this setting for pre-menopausal subjects.

In more recent years, as in other areas of oncology, one of the key advances in field of BC has been the development of targeted therapy. Forty years of laboratory research have improved the understanding of the dysregulated signalling pathways, and connected DNA, RNA and protein alterations that drive the malignant behaviour of cancer cells. Targeting these alterations and pathways has led to great improvements in certain populations of subjects with BC. Agents such as trastuzumab, lapatinib and pertuzamab are now in clinical use for subjects with HER2 positive BC, and have led to more cures in early disease and extended survival in advanced disease (37–39), but also to growing concerns over the cost-effectiveness of new agents being used for BC [40,41].

The success of anti-Her2 therapy has led to interest in identifying new molecular targets in BC for other subtypes. Accumulating preclinical evidence suggests the cyclin dependent kinases (CDK), a family of molecules that play a key role in the control of cell division as an interesting therapeutic target. In the normal cell, inhibition of CDKs during repair of DNA copy errors is a central mechanism for avoiding transmission of mutated DNA. Auto-activation of these molecules can thus, not only lead to high proliferation, but also to increased transmission of altered DNA [42]. A large number of molecular aberrations affecting CDKs and their respective cyclins have been uncovered in BC. Cyclin D1 and cyclin E overexpression have been connected to worse prognosis in multiple studies [43,44]. Dysregulation of CDK signaling has moreover been strongly implicated in resistance to hormonotherapy. In one study high expression of cyclin E predicted a shorter response with endocrine therapy in cell lines [45]. This is specially clinically relevant in the luminal B subgroup that has usually a more aggressive natural history and worse prognosis (46–48). Thus, the relevance of the CDK signaling pathway in both driving more aggressive biological behavior and in causing resistance to treatment has led to the development of a new class of targeted agents, the CDK inhibitors. Palbociclib is the first agent in this class to be approved for clinical use.

Palbociclib (PD0332991) is an orally bioavailable highly selective inhibitor of CDK4/cyclinD1 kinase activity (IC50 = 11 nM; K_i = 2 nM). Palbociclib is selective for CDK4/6, with little or no activity against other CDKs. CDK6 is closely linked to CDK4 and performs the same functions. Thus to effective inhibit CDK4 function, CDK 6 must also be inhibited. Palbociclib inhibits CDK4/6 and thus cellular proliferation and DNA synthesis by preventing cells from entering S phase of the cell cycle. Available evidence shows that palbociclib leads to G1 cell

cycle arrest (and a corresponding loss of Ki67) and induced senescence, as long as cells areRetinoblastoma (Rb)-positive [(49)]. Phase I/II studies in multiple different tumour types have determined its therapeutic potential and a mild to moderate toxicity profile (50–53).

In February 2015, the FDA granted accelerated approval for palbociclib in combination with letrozole as 1st line treatment in metastatic HR-positive and HER2-negative breast cancer based on the results of the PALOMA 1 study.

In February 2016, the FDA approved palbociclib in combination with fulvestrant as a treatment for women with HR-positive and HER2-negative breast cancer progressing on previous endocrine treatment, based on the results of the PALOMA 3 trial [(1,3)].

In the PALOMA 1 phase 2 randomised placebo controlled trial, 165 subjects were randomised between letrozole and palbociclib versus letrozole in first line metastatic disease. The combination arm had improved progression free survival (PFS), 20.2 versus 10.2 months for the letrozole alone group [(1)]. PALOMA 2, a randomized phase 3 trial designed to validate the results of PALOMA 1 found very similar results – median PFS was 24.8 versus 14.5 months (HR=0.58 95 IC 0.46–0.72, P<0.000001) [(2)]. In PALOMA3, a randomised placebo controlled phase 3 trial in 521 subjects that had failed previous hormonotherapy, the comparison between fulvestrant and fulvestrant plus palbociclib favoured the combination. The median PFS, respectively, was 3.8 months and 9.2 months [(3)]. Toxicity in both trials was, however, significantly more intense in the combination arms. Neutropenia occurred in 78.8% of subjects in PALOMA 3 and 74% in PALOMA 1, though with a low number of infections and hospital admission secondary to these adverse events [(1,3)]. Evidence suggests that the causes of neutropenia associated in palbociclib are different than in neutropenia associated with chemotherapy [(54)]. Other common adverse events included anaemia, nausea and fatigue.

Other trials such as the PALLAS trial (NCT02513394) and the PENELOPE-B trial (NCT01864746) are being conducted in the adjuvant and metastatic settings to confirm previous results and inquire on the possibility of palbociclib proving beneficial in other treatment settings. Should these trials prove to be positive, palbociclib may become a treatment standard for the 70% of BC subjects that are estrogen receptor positive in multiple treatment settings.

So far in the neoadjuvant setting only small or medium sized phase 2 studies have been reported. The largest, by Ma and colleagues, with 39 subjects receiving endocrine therapy for one month followed by endocrine therapy and palbociclib for 3 months showed an overall response rate of 67% using ultrasound (according to WHO criteria). After 15 days of palbociclib plus endocrine therapy, reduction of Ki67 was marked (86% complete cell cycle arrest) [(55,56)].

Thus, the development of biomarkers in order to better define the population that benefits from the use of palbociclib, thereby also protecting both subjects that would not benefit from toxicity and the health care system from unnecessary spending would be beneficial. So far there are no available predictive biomarkers for palbociclib apart from ER status (with palbociclib being mostly effective in ER-positive tumours). In PALOMA 1, CCND1 amplification or p16 loss were evaluated but proved not to be useful. As previously

mentioned, the neoadjuvant setting is especially favourable for the development of biomarkers, as it allows for evaluation of both pre and post treatment tumour tissue.

Recently, it has been demonstrated in breast cancer cell lines that the CDK4 T172 phosphorylation is associated with sensitivity to palbociclib and a signature of an 11-gene expression has been developed that can faithfully predict the CDK4 modification profile in breast tumours and breast cancer cell lines (Raspe et al Under Review). This 11-gene expression signature has been associated with sensitivity to CDK 4/6 inhibitors e.g. palbociclib in breast cancer cell lines (Patent : Method For determining sensitivity to a CDK4/6 inhibitor, filed the 18.05.16, European Patent Application No/patent No EP16170146.1-1403, Applicant: Université Libre de Bruxelles, Belgium. Unpublished; private communication).

NeoRHEA is a Phase II open-label, single arm, clinical trial that will include female subjects regardless of menopausal status that have ER-positive, HER2-negative early breast cancer. Subject will receive 4 cycles of palbociclib 125 mg combined with endocrine therapy, followed by surgery. For post-menopausal subjects endocrine therapy will consist of letrozole 2.5 mg continuously, until the last day of palbociclib treatment (i.e. the day before surgery). For pre-menopausal (or peri-menopausal) subjects' treatment will consist of tamoxifen 20 mg every day continuously combined or not with goserelin 3.6 mg monthly. Endocrine therapy for both groups should continue until the last day of palbociclib treatment (i.e. the day before surgery).

Table 1. An 11-gene expression signature that is associated with response/resistance to palbociclib in breast cancer cell lines

Selected genes
CDKN2A
CCNE1
TAGLN2
TIMM17A
RAB31
CCDC99
NUP155
FBXL5
GSN
TP53TG1
PPP1R3C

1.2. Potential Risks:

- Increased Toxicity: the results of PALOMA 1 and 3 strongly suggest that the association of palbociclib and endocrine therapy is more toxic than endocrine therapy alone, with the most significant increased toxicities being neutropenia, anemia, nausea and fatigue [1,3].
- Increased Surgical Complications: So far experience with palbociclib and endocrine therapy in combination with surgery is limited, and though wound-healing issues have not been a major component of reported toxicities so far, this is possible.

1.3. Potential Benefits:

- Potentially Effective Treatment Regimen: Subjects entering this study will receive a regimen that has been shown to be superior to endocrine treatment alone in the metastatic setting, and that available data from one phase 2 study suggests is effective and well tolerated. Therefore it is possible that subjects that receive this regimen may derive better outcomes.
- Benefits of the Successful Development of the Biomarker: Unselected use of targeted agents can lead to worse outcomes, increased toxicity and waste of health care resources. The successful development of a genomic signature that can better select subjects that will respond can diminish the number of subjects that are harmed by the use of ineffective targeted therapy, as well as help diminish costs for health care systems.

2. OBJECTIVES AND ENDPOINTS OF THE STUDY

2.1. Objectives

2.1.1. Primary objective:

- To identify biomarkers of resistance to a 4-month preoperative treatment of palbociclib plus endocrine therapy defined as stable or progressive disease by ultrasound (based on WHO criteria) using RNA-seq of the baseline tumour biopsy.

2.1.2. Secondary objectives:

- To identify biomarkers of resistance to a 4-month preoperative treatment of endocrine therapy and palbociclib by correlating tumour response by ultrasound (mandatory) or magnetic resonance (optional) imaging (response will be assessed as continuous or categorical variable) with RNA-seq and whole genome sequencing (WGS) of the baseline tumour biopsy.
- To identify biomarkers of resistance to a 4-month preoperative treatment of palbociclib plus endocrine therapy defined as residual disease burden, RCB of 3 using RNA-seq and WGS of the baseline tumour biopsy.
- To identify biomarkers of resistance to a 4-month preoperative treatment of palbociclib plus endocrine therapy defined as GGI high by RNA-seq and WGS of the left over tumour at surgery using RNA-seq of the baseline tumour biopsy.
- To identify genomic and transcriptomic biomarkers of resistance to a 4-month preoperative treatment of palbociclib plus endocrine therapy defined as absence of complete cell cycle arrest (CCA, centrally reviewed Ki67 ≤2.7%) at surgery.
- To understand mechanisms of resistance to the combination of endocrine therapy and palbociclib by comparing the genome and transcriptome of tumours at baseline and at surgery using WGS and RNA-seq.
- To evaluate the safety of the combination of palbociclib plus endocrine therapy.
- To evaluate the role of plasma ctDNA in monitoring response/resistance to pre-operative treatment with endocrine therapy and palbociclib.

- To validate/further refine an 11-gene expression signature associated with response/resistance to palbociclib and endocrine treatment.
- To determine the effect of a pre-operative treatment with endocrine therapy and palbociclib on anti-tumour immune response.
- To determine the effect of a pre-operative treatment with endocrine therapy and palbociclib on tumour senescence.
- To determine the effect of a pre-operative treatment with endocrine therapy and palbociclib on tumour proliferation, as measured by the Ki67 score.

2.1.3. Exploratory objectives:

- To compare changes in clonal composition, transcriptomic changes and changes in the open chromatin state of tumour cells using a combined genomic and transcriptomic (G&T) single tumour cell analysis and chromatin accessibility single tumour cell analysis of exceptional responders and exceptional non-responders. Based on the above results, additional bulk or single cell analyses in the entire study cohort could be performed.

2.2. Endpoints

2.2.1. Primary endpoint

- Baseline transcriptomic profile of resistance to 4 months of palbociclib and endocrine therapy, defined as stable or progressive disease by ultrasound based on WHO criteria [4].

2.2.2. Secondary endpoints

- Baseline genomic and transcriptomic profile of resistance to 4 months of palbociclib and endocrine therapy accessed by ultrasound (mandatory) or magnetic resonance (optional) imaging (response will be assessed as continuous or categorical variable) with RNA-seq of the baseline tumour biopsy.
- Baseline genomic and transcriptomic profile of resistance to 4 months of palbociclib and endocrine therapy, defined as an RCB of 3.
- Baseline genomic and transcriptomic profile of resistance to 4 months of palbociclib and endocrine therapy, defined as a GGI high at surgery.
- Baseline genomic and transcriptomic profile of resistance to 4 months of palbociclib and endocrine therapy, defined as absence of CCA at surgery.
- Genomic and transcriptomic changes between pre-treatment and post-treatment tumour samples.
- Safety.
- Plasma ctDNA analysis to monitor response/resistance to pre-operative treatment with endocrine therapy and palbociclib.
- Validation of 11-gene expression signature associated with response/resistance to palbociclib and endocrine treatment.

- Changes in anti-tumour immune response between pre- and post-treatment tumour samples.
- Changes in tumour senescence between pre- and post-treatment tumour samples.
- Changes in Ki67 scores between pre- and post-treatment tumour samples.

2.1.3 Exploratory endpoints

- Changes in clonal composition, transcriptomic changes and changes in the open chromatin state of tumour cells using a combined genomic and transcriptomic (G&T) single tumour cell analysis and chromatin accessibility single tumour cell analysis of exceptional responders and exceptional non-responders.
    - Exceptional responders will be defined as patients with a RCB of 0/1 and/or either complete CCA at surgery or decrease above the median fold change in GGI between baseline and surgery for the patients analysed;
    - Exceptional non-responders will be defined as patients with either disease clinical progression and/or RCB 2/3 and/or no CCA and/or decrease below the median fold change in GGI between baseline and surgery for the patients analysed.

Depending on the results of the above analyses, additional bulk or single cell analyses in the entire study cohort could be performed.

3. STUDY DESIGN

This is an open-label, single arm, Phase 2 study that will include female subjects with ER-positive, HER2-negative early breast cancer who are candidates for neoadjuvant therapy. Subject will receive 4 cycles of palbociclib 125 mg (each cycle of palbociclib consists of treatment from D1 to D21 followed by a week of rest) combined with endocrine therapy given continuously (each cycle of endocrine therapy consists of treatment from D1 to D28).

Endocrine therapy will consist of:

- Post-menopausal subjects: All subjects will receive letrozole 2.5 mg continuously during palbociclib treatment (which consists of 4 cycles of 28 days), with an added 3 to 7 days as necessary, orally once a day every day until the day before curative intent surgery. There are no rest periods for letrozole (unless if toxicity occurs).
- Pre-menopausal subjects: tamoxifen 20 mg continuously during palbociclib treatment (which consists of 4 cycles of 28 days), with an added 3 to 7 days as necessary, orally once a day every day until the day before curative intent surgery. They may also receive goserelin, at local investigator's discretion, 3.6 mg monthly during the same period. There are no rest periods for this regimen (unless if toxicity occurs).

Subject's response to therapy will be evaluated before and after the 4 cycles of treatment by ultrasound to determine response to therapy. Pre-treatment ultrasound should be performed up to 7 days before the start of treatment. Post-treatment ultrasound should be performed within a maximum of 14 days before surgery. If case local guidelines mandate MRI follow-up for neoadjuvant subjects, these may be performed at the same time points in addition (and not instead off) ultrasound.

Surgery will be performed 4 to 8 days after the end of the 4th cycle of study treatment. Between the end of the 4th cycle and surgery, subjects will continue to receive palbociclib and

endocrine therapy, at the same doses, with the last dose of endocrine therapy and palbociclib administered the day before surgery with a maximum of 7 additional treatment days. In case of delays due to toxicity, the surgery should be performed no later than 4 months + 8 days from beginning of study treatment.

Biopsy samples should be collected after inclusion and prior to Day 1 Cycle 1 of study treatment and will consist of collection of 4 core biopsies (2 FFPE and 2 Frozen). However, if 4 biopsies are already available for submission (2 FFPE and 2 Frozen), which were obtained outside the study due to the Site's standard clinical practice, there is no need to repeat these biopsies after the subject's inclusion in the study. These biopsies must have been obtained up to 6 weeks before the beginning of treatment. FFPE and frozen material will be collected from surgical material after sufficient and relevant part has been retained for the diagnosis or treatment of the subject.

One whole blood sample (1x10 mL) will be collected after inclusion and prior to Day 1 Cycle 1 of study treatment.

Blood samples for plasma processing (4x10 mL per time point) will be collected after inclusion and prior to Day 1 Cycle 1, prior to Day 1 Cycle 2 of study treatment, at the time of surgery and one month after surgery.

All the biological samples collected (pre-treatment biopsy, material from surgery and all blood samples) within the study are mandatory. Pre-inclusion imaging assessment (ultrasound of the breast) is not admissible for assessment of study end-points.

Study design:

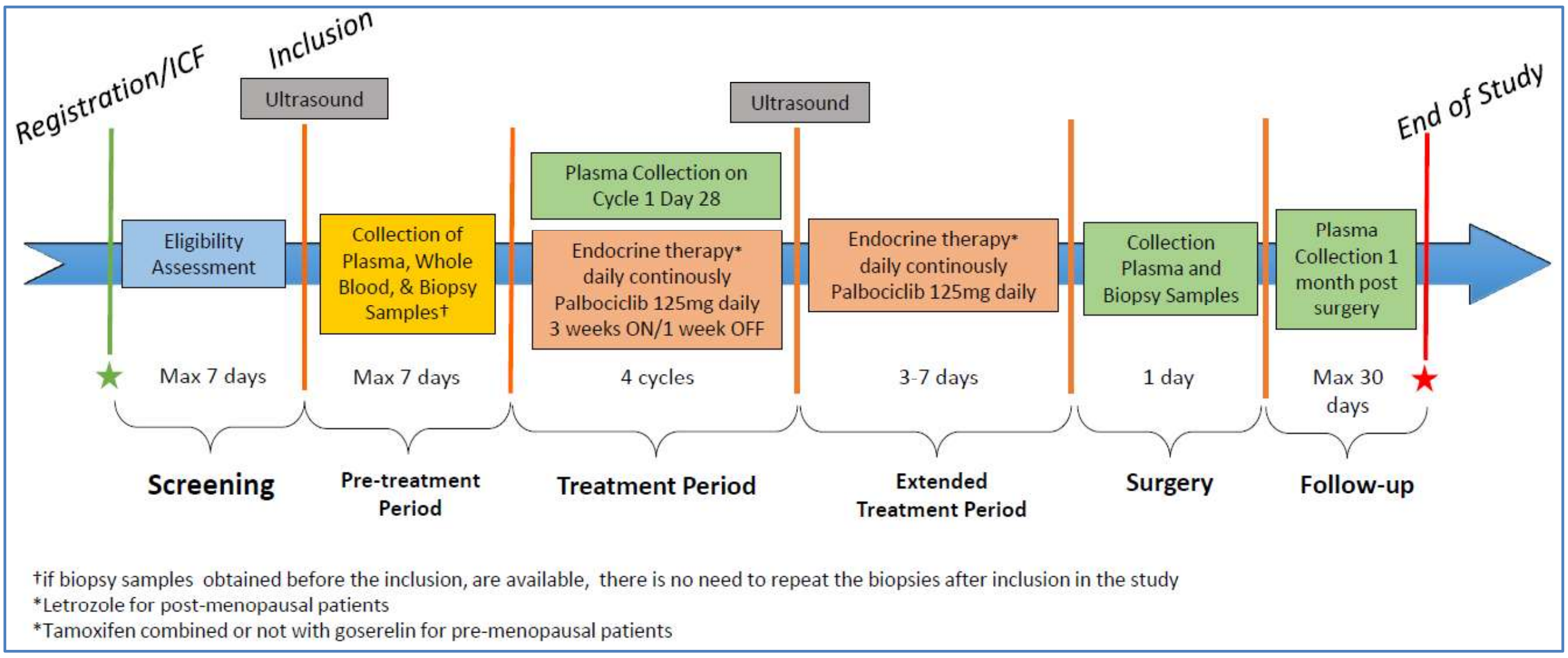

All subjects entering in the study should be followed according to the schedule of assessments detailed in chapter "Schedule of assessments" from informed consent signature up to 1 month after surgery. A dated and signed informed consent must be available before any study-specific assessment or procedure is initiated.

4. SELECTION OF SUBJECTS

4.1. Inclusion criteria

Subjects must meet all of the following criteria in order to be eligible for this study:

1. Female.

2. Age ≥ 18 years

3. Histological diagnosis of breast adenocarcinoma that is estrogen receptor-positive, and HER2- negative as per the updated American Society of Clinical Oncology (ASCO) - College of American Pathologists (CAP) guidelines according to local testing.

4. Multifocal unilateral or bilateral breast adenocarcinoma tumours are allowed provided that all tested foci are:

- ER-positive (ER+ is defined as having a IHC of 1% or more and/or and Allred of 3 or more and HER2-negative).
- HER2 negative (HER2 negative is defined as having an IHC of 0 or 1+ without ISH OR IHC 2+ and ISH non-amplified with ratio less than 2.0 and if reported, average HER2 copy number < 4 signals/cells OR ISH non-amplified with ratio less than 2.0 and if reported, average HER2 copy number < 4 signals/cells [without IHC];
    - Note: a IHC of 3+ is always considered HER2 positive, independently of the ISH result.
    - Note: in discrepant ISH cases (e.g. ratio >2.0 and average HER2 copy number < 4 signals/cells), ASCO/CAP guidelines should be followed and tumours may still be considered HER2-negative, if concordant with the ASCO/CAP guidelines.

5. A primary non metastatic or locally advanced tumour of ≥15 mm or more, assessed by ultrasound, N0 or N1 without prior treatment candidate for preoperative treatment

6. ECOG Performance Status (PS) 0 or 1.

7. Adequate Bone Marrow Function including:

a. Absolute Neutrophil Count (ANC) ≥1500/µL or ≥1.5 $x10^9$/L;

b. Platelets ≥100000/µL or ≥100 x 10^9/L;

c. Hemoglobin ≥ 9 g/dL.

8 Adequate Renal Function including: Serum creatinine ≤ 1.5 x upper limit of normal (ULN) or estimated creatinine clearance ≥ 60 ml/min as calculated using the method standard for the institution.

9. Adequate Liver Function, including all of the following parameters:

a. Total serum bilirubin ≤ 1.0 x ULN unless the subject has documented Gilbert syndrome (in which case up to 3 x ULN is acceptable);

b. Aspartate and Alanine Aminotransferase (AST and ALT) ≤ 1.5 x ULN;

c. Alkaline phosphatase ≤ 2.5 x ULN.

10. Signed consent form

11. Willingness and ability to comply with the study scheduled visits, treatment plans, laboratory tests, radiological exams, tumour and blood specimen collection and other procedures.

12. Women who are not postmenopausal or have not undergone hysterectomy must have documented negative pregnancy test (serum) prior to inclusion.

13. Female subjects of child bearing potential and their partners, who are sexually active, must agree to the use of one highly effective non hormonal form of contraception throughout the period of taking study treatment and for at least 90 days after last dose of study drug, or they must totally/truly abstain from any form of sexual intercourse. Use of oral hormonal contraceptive agents in this study is not permitted.

4.2. Exclusion criteria

Subjects meeting one of the following criteria are not eligible for this study:

1. Clinical T4 disease including inflammatory breast cancer.

2. Prior history of invasive cancer including breast cancer except basal or squamous cell carcinoma of skin that has been definitively treated.

3. Known hypersensitivity to the study drug or excipients.

4. Any illness or medical condition that is unstable or could jeopardize the safety of the subject or her compliance with study requirements.

5. Subjects unable to swallow oral medications.

6. Prior intake of letrozole, tamoxifen or any CDK inhibitor or anti-cancer therapy.

7. Concurrent treatment with any of the drugs not permitted, i.e. strong CYP3A inhibitors/inducers and drugs known to cause QT interval prolongation; (see section 6.7.3 for specific instructions).

8. QTc exceeding 480 msec, family or personal history of long or short QT syndrome, Brugada syndrome or known history of QTc prolongation, or Torsade de Pointes (TdP).

9. Uncontrolled diabetes, according to investigator's clinical judgment.

10. Pregnant or lactating women.

4.3. Subject registration process

The study inclusion will be in 2 steps: study registration and study inclusion.

As soon as the subject has signed the informed consent form, the subject should be registered in the web based Patient Registration tool (= step 1 registration). The system will assign the study subject number.

If the screened subject meets all eligibility criteria, the subject will be included via the web based Patient Registration tool (=step 2 inclusion). The patient must be included in the Registration Tool once the eligibility of the patient has been verified and before starting the pre-treatment period. If the subject does not meet all eligibility criteria, the subject will be considered a screening failure and that must also be reported via the Patient Registration tool as not included.

Screening and inclusion guidelines will be provided to the participating sites.

Screening failure will be defined as any subject that signed ICF and finally is not included in the study for any reason.

5. SUBJECT COMPLETION AND WITHDRAWAL

5.1. Study treatment completion and withdrawal

Investigational study treatment should be administered for 4 cycles plus the extended three to seven days before surgery or until discontinuation due to an unacceptable adverse event, disease progression or withdrawal for other reasons (e.g. subject refuses further treatment).

Once the subject has discontinued study treatment, the reason must be documented in the subject's medical records and CRF. The subject should continue to attend the study visits as per schedule of assessments, unless the subject withdraws consent from entire study.

In the case that the sponsor recommends discontinuation of treatment because of a protocol violation, the subject should remain on study and be followed in accordance with the schedule of assessments.

Once a subject permanently discontinues study treatment, the subject will not be allowed to be receiving further study treatment in the context of this study.

5.2. Study visits completion and withdrawal

Study visits should be performed until completion as per protocol schedule or until withdrawal for other reasons (e.g. subject refuses further study assessments). Once the subject has discontinued the study, the reason must be documented in the subject's medical records and CRF.

5.3. Subject's consent withdrawal

All best efforts shall be made by the participating institutions to keep subjects on study and continuing follow-up according to schedule of assessments. However, a subject may withdraw from the study treatment or study specific procedures or the entire study at any time during the duration of the study for any reason and without prejudicing their future care or future medical treatment or their relationship with the principal investigator.

While a subject can withdraw without needing to give a reason, as soon as a subject has triggered a withdrawal, the principal investigator has the responsibility to establish that the subject's decision is an informed choice and determine to what extent the subject might be willing to continue limited participation in the trial, (e.g., willing to continue being contacted or seen for providing follow-up information). The outcome of the discussion should be documented in both the subject's medical records and the CRF.

It is therefore important to clarify that subject's consent withdrawal is defined within two different scenarios that have a different impact on the study analysis and data collection:

- Consent withdrawal from study treatment: A subject may voluntarily withdraw from study treatment at any time. Subjects must be kept on study and followed up according to the protocol schedule of assessments until subject study completion.

The reason "subject decides to withdraw" for study treatment discontinuation must be recorded on the CRF.

Note: If the subject decides to withdraw from study treatment because of an adverse event (AE) not requiring study treatment discontinuation as per principal investigator judgment, the reason for treatment discontinuation should be reported as "subject decides to withdraw".

- Consent withdrawal from the entire study: Should a subject decide to withdraw from the study, all efforts will be made to complete and report the observations as thoroughly as possible. No further data will be collected after the date of withdrawal from study. The principal investigator should contact the subject or a responsible relative to establish as completely as possible the reason for the withdrawal if the subject is willing to provide the reason. A complete final evaluation at the time of the subject's withdrawal should be made with an explanation of why whenever possible (the subject can refuse to give a reason) the subject is withdrawing from the study. The reason "subject decides to withdraw" must be recorded on the CRF.

  In the presence of an AE that is ongoing at the time of consent withdrawal, appropriate safety evaluations should be attempted as clinically indicated, until resolution.

5.4. Lost to follow-up

In the case of subjects who fail to attend scheduled visits, several attempts should be made by the participating site to contact these subjects for follow up information. At least 3 attempts within a reasonable extent of time should be made to try to contact the subjects if they do not attend clinic visits.

If any of the trial subjects are missing consecutive scheduled visits, the study coordinator and the principal or sub investigator responsible for these subjects must initiate contact. Where these attempts are unsuccessful, the subject's general practitioner must be contacted and request that the subject or her/his family provide follow-up information to the participating site. It is only after sufficient unsuccessful attempts to contact the subject, that a subject may be declared "lost to follow-up". An excessive rate of withdrawals and/or subjects lost to follow-up can make the study uninterpretable; therefore, unnecessary subject attrition should be avoided.

6. STUDY TREATMENTS

6.1. Investigational Medicinal Product: Palbociclib

6.1.1. Drug information

Product code: PD-0332991-00

INN: Palbociclib

Molecular formula: $C_{24}H_{29}N_7O_2$

Molecular weight: 447.54 Daltons

Mechanism of action: Inhibition of CDK 4/6 blocks DNA synthesis by impeding progression of the cell cycle from G1 to S phase.

6.1.1.1. *Formulation*

Palbociclib will be provided as hard gelatin capsules. This formulation will be packaged in appropriate packaging material and should be stored in line with storage conditions (section 6.1.1.3).

Three formulations of capsules will be provided: 125 mg, 100 mg and 75 mg.

6.1.1.2. *Therapeutic regimen and dose*

The regimen consists of Palbociclib 125 mg, once a day, orally, for 4 cycles, to be taken with food, preferably in the evening at approximately the same time each day. Grapefruit, grapefruit juice and preparations containing St. John's Wort should be avoided. Each cycle is composed of 21 days of an "ON period", where the subject takes the medication according to the above description, followed by 7 days of an "OFF period" or "Pause period" where the subject does not take the medication. All efforts should be made so that the subject starts endocrine therapy and palbociclib on the same day. Missed doses cannot be made up. If a subject vomits she cannot retake the vomited dose. If a subject forgets one dose she cannot take two the following day.

Following the end of the 4 cycle period, the subject will receive from 3 to 7 extra days of palbociclib 125 mg, orally, once a day, until the day before surgery.

For subjects who may require dose adjustments, please refer to the chapter 6.1.4 "Dose and schedule modifications due to toxicity".

6.1.1.3. *Stability and storage*

Palbociclib capsules should be stored at controlled and monitored room temperature between 15 and 30 degrees Celsius, in their original container. Further details on the issues of storage and stability are stated in the palbociclib Investigator's Brochure.

It must be stored at the participating sites in a secure area in accordance with Good Clinical Practice (GCP) and Good Manufacturing Practice (GMP) requirements and must be accessible to authorised personnel only.

6.1.1.4. *Labelling*

Palbociclib will be labelled according to the regulatory requirements of each country, as well as with the Rules Governing Medicinal Products in the European Union, Volume 4, EU Guidelines to Good Manufacturing Practice, Medicinal Products for Human and Veterinary Use, Annex 13, Investigational Medicinal Products.

6.1.1.5. *Drug logistics and accountability*

The study drug will be provided free of charge to all participating sites labelled for investigational use only. The site will acknowledge receipt of the IMP by procedure detailed in the pharmacy guidelines. Any damage shipment will be replaced. Accurate records of the IMP reception, dispensing, return from the subject and destruction should be recorded on the Drug Accountability Form.

6.1.2. Toxicity

The adverse reactions observed for palbociclib can be found in the Summary of Product Characteristics.

6.1.3. Toxicities management

For grade 1 and grade 2 with a short duration (less than 4 weeks) palbociclib-related toxicities there are no specific dose adjustments to be followed. Investigators are expected to treat subjects according to their best clinical judgment, based on the clinical situation. Should any interruptions or reductions be determined in the case of grade 2 toxicities, the regulations stated in section 6.1.4 still apply in the same manner as for higher toxicity grades. In the event of multiple toxicities, dose modification should be based on the worst toxicity observed. Subjects should not hold or discontinue palbociclib for non-hematological side effects potentially or likely related to concomitant hormonotherapy therapy (e.g., grade 3 or long lasting grade 2 joint pain) as per the investigator's judgment.

For long lasting grade 2 events (more than 4 weeks), other than alopecia, as well as for grade 3 and 4 events, dose reductions are recommended according to Tables 3 and 4. Investigators are expected also to provide adequate clinical management of complications according to their best judgment. It should be stressed to subjects that experience such toxicities the importance of taking palbociclib with food.

Table 3: Doses and Reduction levels for Palbociclib	
Dose Level	Palbociclib Dose
Standard dose	125 mg/day
Level 1 reduction	100 mg/day
Level 2 reduction	75 mg/day
Any further reduction needed mandates interruption of palbociclib	

Table 4: Management of Toxicities Recommendations		
Febrile neutropenia (at Day1 of the next cycle)	**Grade 3** (ANC <1000/mm^3 with a single temperature of >38.3 degrees C (101 degrees F) or a sustained temperature of >=38 degrees C (100.4 degrees F) for more than one hour) **or**<br><br>**Grade 4** (Life-threatening consequences; urgent intervention indicated)	Hold until clinically stable, if neutrophils = or > 1000 mm^3 and subject has no fever then :<br>First event: resume at reduced dose level 1<br>Second event: resume at reduced dose level 2<br>From third event: interrupt definitively palbociclib
Neutrophil count decreased (at Day 1 of the next cycle)	**Grade 3** (ANC < 1000/mm^3)	Day 1 of cycle:<br>Hold until neutrophils = or > 1000 mm^3 and then repeat

		complete blood count monitoring within 1 week. When recovered to Grade ≤2,<br>First event: resume at same dose<br>Second event: resume at next lower dose<br>From third event: resume at next lower dose<br><br>Note: if the first "Neutrophil count decreased" event was a grade 4 and later in time the patient presents with a new grade 3 event, this is considered a "second event" and the patient should resume treatment at next lower dose.<br><br>Day 14 of first 2 cycles: Continue at current dose to complete cycle. Repeat complete blood count on Day 21.<br>Consider dose reduction in cases of prolonged (>1 week) recovery from Grade 3 neutropenia or recurrent Grade 3 neutropenia in subsequent cycles.
	**Grade 4** (ANC < 500 /mm^3)	Hold until neutrophils = or > 1000 mm^3 and then<br>First event: resume at next lower dose<br>Second event: resume at next lower dose<br>From third event: interrupt palbociclib<br>This also applies if grade 4 is noted at C1D14 or C2D43.
Platelet Count Decrease (at Day 1 of next cycle)	**Grade 3** (<50,000 - 25,000/mm^3)	Hold until platelets = or > 75.000 mm^3 and then:<br>First event: resume at same dose<br>Second event: resume at next lower dose<br>From third event: resume at next lower dose<br><br>Note: if the first "Platelet count decreased" event was a grade 4 and later in time the

		patient presents with a new grade 3 event, this is considered a "second event" and the patient should resume treatment at next lower dose.
	**Grade 4** (<25,000/mm³)	Hold until platelets = or > 75.000mm³ and then:<br>First event: resume at next lower dose<br>Second event: resume at next lower dose<br>From third event: interrupt palbociclib
Anemia (at Day 1 of next cycle)	**Grade 3** (Hgb <8.0 g/dL; transfusion indicated) **or**<br><br>**Grade 4 (**Life-threatening consequences; urgent intervention indicated**)**	Reduce to next lower dose in symptomatic cases considered to be related to palbociclib
ALT increased with slight total bilirubin increase (less than 2x ULN)	**Grade 3** (>5.0 - 20.0 x ULN)	Hold until clinically stable and recovered to baseline or less than grade 1 increase, then:<br>First event: resume at same dose<br>Second event: resume at next lower dose<br>From third event: interrupt palbociclib<br>(If a new increase in ALT does not occur within 4 weeks of palbociclib re-introduction consider re-escalating the dose by single level)<br><br>Note: if the first "ALT increased with slight total bilirubin increase" event was a grade 4 and later in time the patient presents with a new grade 3 event, this is considered a "second event" and the patient should resume treatment at next lower dose.
	**Grade 4** (>20.0 x ULN)	Hold until clinically stable and recovered to baseline or less than grade 1 increase, then:<br>First event: resume at next lower dose<br>Second event: interrupt

		definitively palbociclib
ALT increase to more than 3 x ULN and total bilirubin higher than 2x		Interrupt definitively palbociclib
QTc Prolongation	**Grade 2** (>480 and <500 msec)	1. Reversible cause identified: treat cause and ECG follow-up to the investigator's choice until QTc ≤480 msec. Maintain same dose level(2).<br>2. No reversible cause identified: ECG follow-up to the investigator's choice until QTc ≤480 msec and consult with cardiologist. Maintain same dose level(1).
	**Grade 3** QTc (= or > than 501 msec)	Withhold treatment until QTc <500 msec<br>1. Reversible cause identified: treat and ECG follow-up more frequently to the investigator's choice until QTc ≤480 msec. Maintain same dose level.<br>2. No identifiable cause identified: ECG follow up more frequently to the investigator's choice until QTc ≤480 msec and consult with cardiologist. Resume treatment at the next lower dose level(2).
	**Grade 4** QTc (= or > than 501 msec or > 60 ms change from baseline and life threatening signs including Torsades de pointes)	Interrupt definitively palbociclib
Hyperglycaemia	**Grade 2** (Fasting glucose value >160 - 250 mg/dL; Fasting glucose value >8.9 - 13.9 mmol/L)	Interrupt agent, evaluation with specialist for specific management, re-start at the same dose once case evaluated by specialist. Maintain follow-up in tandem with specialist (clinical and laboratorial) during rest of study treatment.
	**Grade 3** (>250 - 500 mg/dL; >13.9 - 27.8 mmol/L; hospitalisation indicated)	Management in ER or in hospital as per local guidelines. Drug must be interrupted until recovery. Evaluation with specialist for

		specific management after discharge. Maintain follow-up in tandem with specialist (clinical and laboratorial) during rest of study treatment. If recovery takes less than 14 days subject may start with one dose reduction. If longer than 14, permanently discontinue study treatment.
	**Grade 4** (>500 mg/dL; >27.8 mmol/L; life-threatening consequences)	Management as per local in subject guidelines. Evaluation with specialist for specific management after discharge. Maintain follow-up in tandem with specialist (clinical and laboratorial) during rest of study. Permanently discontinue study treatment.
	**Grade 3** or **Grade 4**	Withhold until return to baseline or grade 1, resume at the next lower level.

(1) If the QTc remains at >480 msec for more than 2 cycles or if Grade 2 QTc prolongation recurs in the absence of other alternative causes or despite correction of alternative causes, dose adjustment and/or discontinuation should be considered in consultation with a cardiologist and the medical team, taking into account the emerging safety data from palbociclib trials and the Investigator's best medical judgment.

(2) If the Grade 3 QTc prolongation occurs again after one dose reduction, further dose adjustment and/or discontinuation should be discussed with the medical team, in consultation with a cardiologist, taking into consideration the emerging safety data from palbociclib trials and the Investigator's best medical judgment.

Most common toxicities during use of palbociclib include neutropenia (febrile or not), thrombocytopenia and anemia (Table 4). Other significant toxicities reported in studies in small numbers include cataracts (3 cases in clinical studies), hyperglycaemia and QTc elongation (2 cases reported). Subjects in the study that report vision-related symptoms should temporarily stop study treatment and be evaluated by a specialist, due to risk of cataracts. Use of medications that may impact QTc is restricted as per section 5

6.7.3.1. Furthermore, electrolyte imbalances that may precipitate arrhythmias (such as hypocalcemia, hypokalemia, hypomagnesemia) should be managed with appropriate electrolyte supplementations.

6.1.4. Dose and schedule modifications due to toxicity

Whenever a dose reduction or interruption is necessary, as per section 6.1.3, the following rules should be observed:

(1) Should a dose reduction from standard dose to level 1 or 2 reduction (see Table 3) prove necessary, it will not be possible to return standard higher dose (except in the case of ALT increase as described in table 4).

(2) Temporary interruptions of one agent do not entail interruption of the other in cases where toxicity can be clearly connected to one agent only. Doses that are missed due to temporary interruptions in the middle of cycles will not be compensated for, i.e. – each "treatment" phase of a cycle ends 21 days after cycle day one, no matter how many days the subject actually took palbociclib. In case either of the agents is interrupted permanently the other will also be interrupted permanently and surgery will be planned within the study. Delays of more than 4 weeks will lead to the interruption of study treatment.

(3) In case of palbociclib toxicity-related interruptions in the middle of a cycle, as a general rule, treatment may only be resumed in the same cycle if AE is resolved or reduced to a grade 1 (see Table 3 for orientations on specific toxicities).

(4) At the start of each cycle, subjects should have recovered from any AEs (or they should be less than grade 1). Platelets should be at least $75{,}000/mm^3$ and neutrophils at least $1000/mm^3$. In case these criteria are not met, the start of next cycle may be delayed.

In case of delays due to toxicity, the surgery should be performed no later than 4 months + 8 days from beginning of study treatment. Should any adaptations be necessary to palbociclib schedule, they are left at the investigator's discretion, as long as the subjects receive palbociclib for 3 days before surgery.

6.2. Investigational Medicinal Product: Letrozole

6.2.1. Drug information

INN: Letrozole

Chemical name: 4, 4'-[(1H-1,`2, 4-triazol-1-yl)-methylene] bis-benzonitrile

Molecular formula: $C_{17}H_{11}N_5$

Mechanism of action: Letrozole is a non-steroidal aromatase inhibitor. It inhibits the aromatase enzyme by competitively binding to the haem of the aromatase cytochrome P450, resulting in a reduction of estrogen biosynthesis in all tissues where present.

6.2.1.1. *Formulation*

The drug is available as coated tablets containing 2.5 mg letrozole.

6.2.1.2. *Therapeutic regimen and dose*

The regimen consists of letrozole 2.5 mg, once a day, orally continuously until the day before surgery. All efforts should be made so that the subject starts letrozole and palbociclib on the same day

Letrozole can be taken with or without food. Proton pump inhibitors may be used.

A missed dose should be taken as soon as the subject remembers. However, if it is almost time for the next dose (within 2 or 3 hours), the missed dose should be skipped, and the subject should go back to her regular dosage schedule.

6.2.1.3. *Stability and storage*

Letrozole has a shelf life of 5 years. It should not be stored above 30°C and should be stored in the original package in order to protect from moisture.

It must be stored at the participating sites in a secure area in accordance with Good Clinical Practice (GCP) and Good Manufacturing Practice (GMP) requirements and must be accessible to authorised personnel only.

6.2.1.4. *Labelling*

Letrozole will be labelled according to the regulatory requirements of each country, as well as with the Rules Governing Medicinal Products in the European Union, Volume 4, EU Guidelines to Good Manufacturing Practice, Medicinal Products for Human and Veterinary Use, Annex 13, Investigational Medicinal Products.

6.2.1.5. *Drug logistics and accountability*

The study drug is taken from the commercial stocks of the hospital pharmacies and will be re-labeled by the pharmacists prior to providing them to the subjects. Accurate records of the IMP dispensing, return from the subject and destruction should be recorded on the Drug Accountability Form.

Detailed pharmacy guidelines will be provided to participating sites prior to the start of the trial.

6.2.2. Toxicity

The adverse reactions observed for letrozole can be found in the Summary of Product Characteristics.

6.2.3. Toxicities management

We expect the investigator to manage according to his best clinical judgment any AEs considered to be related to letrozole, of any grade. For subjects experiencing severe arthralgia with limitation or complete loss of ability for daily life activities (grade 2 or 3), use of analgesics is encouraged, and if necessary temporary interruption of letrozole. There are no dose reductions. In case letrozole is interrupted, it should be restarted within 10 days maximum once symptoms resolve. A second dose interruption is not allowed. Should letrozole treatment prove intolerable after first interruption subject should be taken off study treatment permanently. In case treatment with letrozole is interrupted treatment with palbociclib will also be interrupted.

6.2.4. Dose and schedule modifications due to toxicity

Letrozole should be taken continuously as per study regimen, at 2.5 mg per day every day. It should be started on the first day of the first cycle of palbociclib and stopped on the last day before surgery.

- Should a delay in the start of a palbociclib cycle be necessary for any reason, letrozole should be maintained.
- Temporary interruptions of one agent do not entail interruption of the other in cases were toxicity can be clearly connected to one agent only. Doses that are missed due to temporary interruptions in the middle of cycles will not be compensated. In case either of the agents is interrupted permanently the other will also be interrupted permanently and surgery will be planned within the study.
- In case of delays due to toxicity, the surgery should be performed no later than 4 months + 8 days from beginning of study treatment. Letrozole should be taken until the last day before surgery.

6.3. Investigational Medicinal Product: Goserelin

6.3.1. Drug information

INN: Goserelin

Other active substance name: Goserelin acetate

Chemical name: (2S)-N-[(2S)-1-[[(2S)-1-[[(2S)-1-[[(2S)-1-[[(2R)-1-[[(2S)-1-[[(2S)-1-[(2S)-2-[(carbamoylamino)carbamoyl]pyrrolidin-1-yl]-5-(diaminomethylideneamino)-1-oxopentan-2-yl]amino]-4-methyl-1-oxopentan-2-yl]amino]-3-[(2-methylpropan-2-yl)oxy]-1-oxopropan-2-yl]amino]-3-(4-hydroxyphenyl)-1-oxopropan-2-yl]amino]-3-hydroxy-1-oxopropan-2-yl]amino]-3-(1H-indol-3-yl)-1-oxopropan-2-yl]amino]-3-(1H-imidazol-5-yl)-1-oxopropan-2-yl]-5-oxopyrrolidine-2-carboxamide

Molecular formula: $C_{59}H_{84}N_{18}O_{14}$

Mechanism of action: goserelin is a synthetic analogue of naturally occurring LHRH. On chronic administration goserelin results in inhibition of pituitary LH secretion leading to a fall in serum estradiol concentrations in females. This effect is reversible on discontinuation of therapy. Initially, goserelin, like other LHRH agonists, may transiently increase serum estradiol concentration in women.

6.3.1.1. *Formulation*

The drug is available as implant, pre-filled syringe containing 3.6 mg of goserelin.

6.3.1.2. *Therapeutic regimen and dose*

The regimen consists of goserelin 3.6 mg, subcutaneously, at the beginning of each cycle for 4 monthly cycles. goserelin use is not mandatory for pre-menopausal subjects, but can occur at investigator's discretion. All efforts should be made so that the subject starts goserelin, tamoxifen and palbociclib on the same day. Injections of goserelin can be schedule for visit days for administrative ease and subject convenience.

Following the end of the 4 cycle period, the subject should receive an extra dose of goserelin that is equivalent to the extra "extended treatment period" for the other agents (total of 5 doses). If a subject misses one injection it can be given as soon as possible.

For subjects who may require dose interruptions, please refer to the section 6.3.4"Dose and schedule modifications due to toxicity".

6.3.1.3. *Stability and storage*

Goserelin has a shelf life of 36 months and should not be stored above 25°C.

It must be stored at the participating sites in a secure area in accordance with Good Clinical Practice (GCP) and Good Manufacturing Practice (GMP) requirements and must be accessible to authorised personnel only.

6.3.1.4. *Labelling*

Goserelin will be labelled according to the regulatory requirements of each country, as well as with the Rules Governing Medicinal Products in the European Union, Volume 4, EU Guidelines to Good Manufacturing Practice, Medicinal Products for Human and Veterinary Use, Annex 13, Investigational Medicinal Products.

6.3.1.5. *Drug logistics and accountability*

The study drug is taken from the commercial stocks of the hospital pharmacies and will be re-labeled by the pharmacists prior to providing them to the subjects. Accurate records of the IMP dispensing, return from the subject and destruction should be recorded on the Drug Accountability Form.

Detailed pharmacy guidelines will be provided to participating sites prior to the start of the trial.

6.3.2. Toxicity

The adverse reactions observed for goserelin can be found in the Summary of Product Characteristics.

6.3.3. Toxicities management

We expect the investigator to manage according to his best clinical judgment any AEs considered to be related to goserelin, of any grade. As the use of goserelin is optional, it can be interrupted due to any reason at investigator's discretion, temporarily or permanently. A permanent interruption of goserelin does not determine a permanent interruption of study treatment – tamoxifen and palbociclib can be continued.

6.3.4. Dose and schedule modifications due to toxicity

See above in 6.3.3

6.4. Investigational Medicinal Product: Tamoxifen

6.4.1. Drug information

INN: tamoxifen

Other active substance name: tamoxifen citrate

Chemical name: 2-[4-[(Z)-1,2-diphenylbut-1-enyl]phenoxy]-N,N-dimethylethanamine

Molecular formula: $C_{29}H_{29}NO$

Mechanism of action: tamoxifen is a non-steroidal, triphenylethylene-based drug which displays a complex spectrum of estrogen antagonist and estrogen agonist-like pharmacological effects in different tissues. In breast cancer subjects, at the tumour level, tamoxifen acts primarily as an antioestrogen, preventing estrogen binding to the estrogen receptor.

6.4.1.1. *Formulation*

The drug is available as tablet containing tamoxifen citrate 30.4 mg (equivalent to 20 mg tamoxifen).

6.4.1.2. *Therapeutic regimen and dose*

The regimen consists of tamoxifen 20 mg, once a day, orally continuously until the day before surgery. All efforts should be made so that the subject starts tamoxifen and palbociclib on the same day. Tamoxifen can be taken with or without food. Grapefruit and grapefruit juice should be avoided. A missed dose should be taken as soon as the subject remembers. However, if it is almost time for the next dose (within 2 or 3 hours), the missed dose should be skipped, and the subject should go back to her regular dosage schedule.

6.4.1.3. *Stability and storage*

Tamoxifen has a shelf life of 5 years. It should not be stored above 30°C and be stored in the original container.

It must be stored at the participating sites in a secure area in accordance with Good Clinical Practice (GCP) and Good Manufacturing Practice (GMP) requirements and must be accessible to authorised personnel only.

6.4.1.4. *Labelling*

Tamoxifen will be labelled according to the regulatory requirements of each country, as well as with the Rules Governing Medicinal Products in the European Union, Volume 4, EU Guidelines to Good Manufacturing Practice, Medicinal Products for Human and Veterinary Use, Annex 13, Investigational Medicinal Products.

6.4.1.5. *Drug logistics and accountability*

The study drug is taken from the commercial stocks of the hospital pharmacies and will be re-labeled by the pharmacists prior to providing them to the subjects. Accurate records of the IMP dispensing, return from the subject and destruction should be recorded on the Drug Accountability Form.

Detailed pharmacy guidelines will be provided to participating sites prior to the start of the trial.

6.4.2. Toxicity

The adverse reactions observed for tamoxifen can be found in the Summary of Product Characteristics.

6.4.3. Toxicities management

We expect the investigator to manage according to his best clinical judgment any AEs considered to be related to tamoxifen, of any grade. There are no dose reductions. In case tamoxifen is interrupted, it should be restarted within 10 days maximum once symptoms resolve. A second dose interruption is not allowed. Should tamoxifen treatment prove intolerable after first interruption subject should be taken off study treatment permanently. In case treatment with tamoxifen is permanently interrupted treatment with palbociclib will also be permanently interrupted.

6.4.4. Dose and schedule modifications due to toxicity

Tamoxifen should be taken continuously as per study regimen, at 20 mg per day every day. It should be started on the first day of the first cycle of palbociclib and stopped on the last day before surgery.

- Should a delay in the start of a palbociclib cycle be necessary for any reason, tamoxifen should be maintained.
- Temporary interruptions of one agent do not entail interruption of the other in cases were toxicity can be clearly connected to one agent only. Doses that are missed due to temporary interruptions in the middle of cycles will not be compensated. In case either of the agents is interrupted permanently the other will also be interrupted permanently and surgery will be planned within the study.
- In case of delays due to toxicity, the surgery should be performed no later than 4 months + 8 days from beginning of study treatment.

6.5. Protocol surgery

6.5.1. Surgical treatment

Curative Breast surgery will be performed 4–8 days after the end of the 4th cycle of study treatment. Subject should receive study therapy (extended period of 3-7 days of additional treatment) up to the day before surgery. In case of delays due to toxicity, the surgery should be performed no later than 4 months + 8 days from beginning of study treatment. Surgical technique is left at the discretion of the responsible surgeon. Tissue must be collected for analysis of end-points, and therefore it is very important that the surgeon be made aware of subject status as a participant in the study and of necessary study procedures, namely that FFPE and frozen material will be collected from the surgical material left over, after sufficient and relevant parts has been retained to establish, improve or complement the diagnosis or treatment of the subject.

6.5.2. Surgery toxicity

Table 8: Surgical Adverse Reactions

Very frequent	Seroma
Frequent	Pain (burning, aching), phantom breast syndrome, arm morbidity (arm numbness, arm stiffness, shoulder pain, shoulder stiffness, nerve injury)
Infrequent	Wound infection, lymphedema

6.6. Contraception and menopausal status

6.6.1. Contraception

Female subjects of childbearing potential must agree to always use one highly effective non hormonal method of contraception or alternatively will be asked to abstain from sex throughout the course of this study and for at least 90 days after the last administration of the IMP. Hormonal methods of contraception are not allowed.

The highly effective methods of contraception accepted are the following:

- Intrauterine device (IUD)
- Intrauterine hormone-releasing system (IUS)
- Bilateral tubal occlusion
- Vasectomised partner

6.6.2. Menopausal status

Post-menopausal status of subjects will be defined as follows:

- Age ≥60 years or,
- Age < 60 years and amenorrhea for 12 or more consecutive months in the absence of alternative pathological or physiological cause (including ovarian suppression) and FSH and serum estradiol levels within the laboratory's reference ranges for postmenopausal women.
- Prior bilateral oophorectomy (≥28 days prior to Day 1 of treatment)

Any subjects that are not post-menopausal, according to these criteria are considered to be pre-menopausal and should be treated as such.

6.7. Concomitant treatments

6.7.1. Allowed Concomitant treatments

Concomitant treatments include any medications (e.g. prescription medications, over-the-counter preparations, herbal/homeopathic remedies, nutritional supplements) used by the subject from 7 days prior to the first administration of study treatment through the subject end of study.

All concomitant treatments medications (including start/stop dates, dose, indication and any other details as per CRF completion guideline) should be recorded in the CRF up to 30 days following the last administration of study treatment(s).

All medication used for supportive care are allowed, unless they are part of the prohibited list provided below. Relevant classes of supportive care include antiemetic, antidiarrheal, corticosteroids, antihistamines, anti-inflammatory drugs and anti-depressants.

6.7.2. Prohibited Concomitant treatments

The following treatments are not allowed during the treatment period of the study and for at least 7 days prior to initiation of study treatment, unless otherwise specified below:

- Any and all cancer treatments (chemotherapy, targeted therapy, radiotherapy) besides study treatment are prohibited during the study.
- Granulocyte colony stimulating factor unless subject becomes clinically unstable due to neutropenia.
- Dexamethasone and other immunosuppressant agents should be avoided if possible.
- Herbal remedies should not be used if possible.
- Hormone replacement therapy – systemic or topic is also prohibited during study treatment.
- All medications listed in section 6.7.3 should not be used during study treatment. Any subject that receives a medication in this list (and that cannot switch to allowed medication in a timely fashion) cannot be entered into the trial.

6.7.3. Incompatibilities/Drug Interactions

6.7.3.1. *Palbociclib*

Strong CYP3A4 inhibitors: Avoid concomitant use with strong CYP3A4 inhibitors (eg, amprenavir, atazanavir, boceprevir, clarithromycin, conivaptan, delavirdine, diltiazem, erythromycin, fosamprenavir, indinavir, itraconazole, ketoconazole, lopinavir, mibefradil, miconazole, nefazodone, nelfinavir, posaconazole, ritonavir, saquinavir, telaprevir, telithromycin, voriconazole, and grapefruit or grapefruit juice) and consider alternatives with no or minimal CYP3A4 inhibition. If coadministration with a strong CYP3A4 inhibitor cannot be avoided, reduce palbociclib dose to 75 mg once daily. If the strong inhibitor is discontinued, increase palbociclib dose (after 3 to 5 inhibitor half-lives have elapsed) to the dose used prior to initiating the strong CYP3A4 inhibitor.

CYP3A4 inducers: Avoid concomitant use with moderate or strong CYP3A4 inducers such as carbamazepine, enzalutamide, felbamate, nevirapine, phenobarbital, phenytoin, primidone, rifabutin, rifampin, rifapentin, and St. John's wort.

Effect of other agents: The PPI rabepraszole may decrease palbociclib concentrations under fasted conditions (limited effect under fed conditions). Palbociclib may increase midazolam concentrations. The dose of sensitive CYP3A substrates with a narrow therapeutic index (e.g., alfentanil, cyclosporine, dihydroergotamine, ergotamine, everolimus, fentanyl, pimozide, quinidine, sirolimus, and tacrolimus) may need to be reduced when coadministered with palbociclib as palbociclib may increase their exposure.

Agents that elongate QTc: amiodarone, arsenic trioxide, astemizole, azithromycin bepridil, chloroquine, chlorpromazine, cisapride, citalopram, clarithromycin disopyramide, dofetilide, domperidone, droperidol, erythromycin, flecainide , halofantrine, haloperidol, ibutilide, levomethadyl, mesoridazine, methadone, moxifloxacin, pentamidine, pimozide, probucol, procainamide, quinidine, sotalol , sparfloxacin, terfenadine, thioridazine, vandetanib

6.7.3.2. *Letrozole*

Letrozole is a strong inhibitor of CYP2A6 and a weak inhibitor of CYP2C19. CYP2A6 substrates should be avoided, including disulfiram, valproic acid and halothane(57). Methadone can also have its plasma concentration increased and should be avoided.

6.7.3.3. *Goserelin*

Since estrogen deprivation treatment may prolong the QT interval, the concomitant use of goserelin with medicinal products known to prolong the QT interval or medicinal products able to induce Torsade de pointes such as class IA (e.g. quinidine, disopyramide) or class III (e.g. amiodarone, sotalol, dofetilide, ibutilide) antiarrhythmic medicinal products, methadone, moxifloxacin, antipsychotics, etc. should be carefully evaluated.

6.7.3.4. *Tamoxifen*

Tamoxifen is a substrate of CYP2A6 (minor), CYP2B6 (minor), CYP2C9 (major), CYP2D6 (major), CYP2E1 (minor), CYP3A4 (major); it Inhibits CYP2C8 (moderate), CYP2C9 (weak), P-glycoprotein. Use of SSRI antidepressants is not recommended during tamoxifen treatment such as fluoxetine paroxetine and sertraline. As tamoxifen is metabolised by cytochrome P450 3A4, care is required when co-administering with drugs, such as rifampicin, known to induce this enzyme as tamoxifen levels may be reduced. The clinical relevance of this reduction is unknown. When tamoxifen is used in combination with coumarin-type anticoagulants, a significant increase in anticoagulant effect may occur. Where such co-administration is initiated, careful monitoring of the subject is recommended

6.8. Study Treatment duration

Study treatment with endocrine therapy (continuous administration) and palbociclib (3 weeks on/1 week off) is composed of 4 cycles of 28 days, followed by a “bridge” period of 3-7 days of concomitant endocrine therapy and palbociclib until the day prior to surgery. For premenopausal women for which the treating physician choose to add goserelin to study treatment, the last injection of goserelin should be given at the beginning of the extended treatment period. The last dose of oral endocrine therapy and palbociclib should be given the day before surgery, in the evening. In case of delays due to toxicity, the surgery should be performed no later than 4 months + 8 days from beginning of study treatment. If any adaptations to palbociclib schedule are necessary, they are left at the local investigator’s discretion, as long as the subject receives palbociclib for 3 days before surgery.

7. SCHEDULE OF ASSESSMENTS

7.1. Screening period

During the screening period, following signature of the Informed Consent Form and registration, the principal investigator will confirm the subject's eligibility for the study by conducting the assessments described below and complete the corresponding CRF.

All screening evaluations are to be performed *within 7 days before inclusion*.

The evaluations and procedures to be conducted in the screening period include the following:

- Signature of ICF (as soon as the ICF signed, the subject should be registered in the study via Patient Registration Tool)
- Demographic data: gender, date of birth
- Eligibility check: review of inclusion and exclusion criteria
- Diagnosis: subjects with ER-positive/HER2-negative, Non-metastatic breast cancer, with 15 mm or more, determined by ultrasound, and N0 or N1 disease, who are candidates for preoperative treatment for breast cancer.
- Medical history:
  - Clinically significant prior and/or current medical conditions
  - Surgical procedures.
  - Menopausal status: Date of last menstrual period and FSH and estradiol levels for non post-menopausal patients
- Cancer history and prior cancer therapies
- Concomitant medications, therapies and procedures from 7 days prior to the first administration of study treatment.
- Physical examination and vital signs: body weight, height, blood pressure, and pulse (heart rate)
- Eastern Cooperative Oncology Group performance status
- Pregnancy test for pre-menopausal subjects: Serum
- Laboratory tests:
  - Haematology including leucocytes, neutrophils, platelets, hemoglobin, hematocrit and erythrocytes
  - Biochemistry including serum creatinine or estimated creatinine clearance, liver function tests (e.g: bilirubin as well as indirect and direct bilirubin, aspartate and alanine aminotransferase, alkaline phosphatase), electrolytes (potassium, sodium, magnesium and calcium).
  - Fasting glucose
- ECG
- SAEs as described in the paragraph "Safety definitions and reporting guidance".

The principal investigator or any designated staff member must review results of all screening evaluations and procedures to ensure that all eligibility criteria are satisfied.

Once eligibility of the patient in the study has been verified, she must be included in the Patient Registration Tool.

Screen failure is defined as any subject that signed the ICF and finally is not included in the study.

Any screen failure will also be recorded in the Patient Registration tool.

7.2. Pre-treatment period

The evaluations and procedures listed below are to be conducted **after Inclusion** and **Prior to Cycle 1**: Max 7 days.

- Breast ultrasound to be performed maximum one week prior to start of combined study treatment. Recording of at least 2 dimensions (longest diameter x greatest perpendicular diameter) of the primary tumour will be performed and presence, number and size (not mandatory) of lymph nodes will be evaluated.
- Optional MRI: In sites where an MRI is a standard part of evaluation and follow-up during neoadjuvant treatment, a pre-treatment MRI should be performed in addition to the standard pre-treatment ultrasound and is highly recommended. If an MRI is available within 4 weeks from treatment start, this will be accepted.
- SAEs as described in the paragraph "Safety definitions and reporting guidance"
- Concomitant medications, therapies and procedures from 7 days prior to the first administration of study treatment.
- Samples collection:
  - Biopsy samples (2 FFPE and 2 Frozen) via a new biopsy done for study purposes; note: if samples are already available (2 FFPE and 2 Frozen) which were obtained before Inclusion due to the Site's standard clinical practice, there is no need to repeat these biopsies after the subject's Inclusion in the study - these biopsies must have been obtained up to 6 weeks before the beginning of treatment.
  - Whole blood sample (1x10 mL)
  - Blood sample for plasma processing (4x10 mL)
- Glycosylated haemoglobin

For bilateral, multifocal or multicentric disease, the site selected for pre-treatment biopsy should correspond to the site of largest measurable disease meeting eligibility criteria. The location of tumour biopsy site (laterality, quadrant, position from the nipple and type of imaging modality to guide biopsy) should be collected.

The pre-treatment biopsy can be performed at any time during the pre-treatment period and no later than the day of study medication administration. If the pre-treatment biopsy is performed on the same day that the first study medication intake, it should be performed prior to this first medication intake.

- Important note: the post-treatment tumour biopsy obtained at the time of definitive surgery should be performed at the same site as the pre-treatment biopsy. Marking and identifying the pre-treatment biopsy site will follow local practice methods.

7.3. Study treatment period

The evaluations and procedures listed below are to be conducted in the study treatment period before every cycle of palbociclib begins (to be performed maximum 3 days before start of cycles 1, 2, 3, 4 and before surgery).

- Concomitant medications, therapies and procedures.
- Physical examination and vital signs: body weight, blood pressure and pulse (heart rate)
- ECG (only before cycle 2)
- Pregnancy test for pre-menopausal subjects: serum (can be omitted if realized during the screening period and ≤ 7 days before first day of cycle 1)
- Laboratory tests (except cycle 1)
    - Haematology including white blood cell count, absolute neutrophil count, platelets, haemoglobin, haematocrit and red blood cell count.
    - Biochemistry including serum creatinine or estimated creatinine clearance, liver function tests (e.g: total serum bilirubin as well as indirect and direct bilirubin, aspartate and alanine aminotransferase, alkaline phosphatase), electrolytes (potassium, sodium, magnesium and calcium).
    - Fasting glucose
    - Glycosylated haemoglobin (only before the 4th cycle)
- Surgical procedures: Curative breast surgery to be performed according to local guidelines between 4 and 8 days after the end of the fourth cycle of study treatment.
- Breast ultrasound to be performed within a maximum of 14 days before surgery. Recording of at least 2 dimensions (longest diameter x greatest perpendicular diameter) of the primary tumour will be performed and presence, number and size (not mandatory) of lymph nodes will be evaluated.
- Optional MRI (within a maximum of 14 days before surgery): In sites where an MRI is a standard part of evaluation and follow-up during neoadjuvant treatment and when pre-treatment MRI was performed, a pre-surgery MRI should be performed in addition to the standard pre-surgery ultrasound and is highly recommended.
- All AEs including SAEs documented according to NCI-CTCAE v4.03.
- All AESIs
- All pregnancies, lactations
- All ARs due to overdose, misuse, medication error
- Exposure to study medications by non-study subjects
- Samples collection:
    - Blood sample for plasma processing (4x10 mL) to be collected on C1D28 and at the time of surgery.
    - FFPE and Frozen samples (2 cores each) to be collected from surgical material left over that is not needed for diagnostic purposes.

- Important note: the post-treatment tumour biopsy obtained at the time of definitive surgery should be performed at the same site as the pre-treatment biopsy. Marking and identifying the pre-treatment biopsy site will follow local practice methods.

7.4. End of study treatment visit

When a subject completes or is discontinued from the treatment period, an end of treatment (EOT) visit should be performed within 30 days after the surgery or, in the case where surgery is no longer planned due to systemic progression, within 30 days after the end of study medication treatment. The following evaluations and procedures should be performed at this end of study treatment visit:

- Concomitant medications, therapies and procedures, as well if the patient has been proposed for adjuvant chemotherapy (specifying the regimen, with drugs and schedule).
- Physical examination and vital signs: *body weight, blood pressure and pulse (heart rate)*
- Pregnancy test for pre-menopausal subjects: serum
- Laboratory tests:
    - Haematology including white blood cell count, absolute neutrophil count, platelets, haemoglobin, haematocrit and red blood cell count.
    - Biochemistry including serum creatinine or estimated creatinine clearance and liver function tests (e.g: total serum bilirubin, aspartate and alanine aminotransferase, alkaline phosphatase), electrolytes (sodium, magnesium, calcium and potassium).
    - Fasting glucose
- All AEs including SAEs, AESIs documented according to NCI-CTCAE v 4.03.
- From 30 days after surgery, only SAEs and AESIs related to study treatments must be reported.
- All pregnancies, lactations
- Samples collection: Blood sample for plasma processing (4x10mL)

In this study, the EOT visit corresponds to the end of study visit (EOS) for the subject.

7.5. Patient Diary

Each subject will receive at the beginning of study a standard form patient diary. This diary should be filled during treatment, and taken with the subject for each study visit. In this diary the subject should write down daily dose of study treatment taken, time of use, symptoms or signs that occur during study treatment, as well as any drugs taken for said symptoms or signs.

Summary table schedules of assessments

	Screening	Pre-treatment[16]	Treatment Period								End of study
Time points	Prior to Inclusion	Prior to Treatment Start	First Cycle		Second Cycle		Third Cycle	Fourth cycle	Bridge period (3-7 days prior surgery)	Surgery	1 month post-surgery (end-of -study)
Day of Study			D1	D15	D29	D43	D57	D85	D113-- D119	D116-D120	D146-D150
Assessments											
Eligibility	X										
Informed Consent	X										
Registration in Patient Registration Tool	X										
Diagnosis	X										
Demography, Medical history	X										
Physical examination	X		X		X		X	X	X		X
Vital signs	X		X		X		X	X	X		X
ECOG performance status	X										
Laboratory Tests [1]	X				X		X	X	X		X
Hematology[2]				X[3]		X[3]					
Glycosylated haemoglobin		X						X			
Pregnancy Test (serum) [4]	X		X[5]		X		X	X	X		X
ECG	X				X						
Ultrasound evaluation [6]		X							X [7]		
MRI [8]		X							X [7]		
Treatment administration : Palbociclib [9]			3 weeks on/ 1 week off 125 mg						125 mg		
Treatment administration: Endocrine Therapy [9]			Continuously [10]								
Tissue Collection (2FFPE + 2 Frozen)		X[15]								X	
Blood for Plasma processing (4x10ml)		X	X [11]							X	X
Whole Blood (1x10ml)		X									
Adverse events (AEs)			Continuously								X
Serious adverse events (SAEs)	X [12]	X [12]	Continuously								X[13]
Adverse events of Special interest (AESI)			Continuously								X[13]
Procedures	X [14]	X [14]	Continuously								
Concomitant medications	X [14]	X [14]	Continuously								
Surgery										X	

1. Includes white blood cell count, absolute neutrophil count, platelets, haemoglobin, haematocrit, red blood cell count, serum creatinine or estimated creatinine clearance, liver function tests (e.g: total serum bilirubin as well as indirect and direct bilirubin, aspartate and alanine aminotransferase, alkaline phosphatase), electrolytes (potassium, sodium, magnesium and calcium) and fasting glucose
2. Hematology includes hemoglobin, white blood cell count (WBC), absolute neutrophils, platelet count
3. Results of hematology for C1D15 and C2D43 can be communicated by telephone for all patients, and can occur ±2 days around Day 14 and on Day 43. All patients should have AEs collected on the calls
4. Only for pre-menopausal patients
5. Serum pregnancy test for women of childbearing potential can be omitted if realized ≤ 7 days before first day of cycle 1.
6. With recording of at least 2 dimensions (longest diameter x greatest perpendicular diameter) of the primary tumour and the presence, number and size (not mandatory) of lymph nodes.
7. Breast ultrasound and optional MRI to be performed within a maximum of 14 days before surgery, in order to assess response, which should be recorded (complete response, partial response, stable disease or progressive disease).
8. Optional MRI: In sites where an MRI is a standard part of evaluation and follow-up during neoadjuvant treatment, a pre-treatment MRI should be performed in addition to the standard pre-treatment ultrasound and is highly recommended.
9. The last dose of endrocine therapy and palbociclib should be given the day before surgery
10. The goserelin is optional and given on the first day of each cycle and the first day of the bridge period
11. On C1 day 28
12. SAEs related to a protocol-mandated intervention (e.g.SAEs related to invasive procedures such as biopsies) should be reported on SAE form
13. From day 31 after study treatments, only SAEs and AESIs related to study treatment.
14. From 7 days prior to first administration of study treatment.
15. If 4 biopsies are already available for submission (2 FFPE and 2 Frozen), which were obtained outside the study due to the Site's standard clinical practice, there is no need to repeat these biopsies after the subject's inclusion in the study. These biopsies must have been obtained up to 6 weeks before the beginning of treatment
16. Only start when the patient has been included in the Registration Tool

8. ASSESSMENT OF EFFICACY

8.1. Efficacy parameters

The following efficacy parameters will be assessed:

For the primary endpoint:

Subjects will be staged according to ultrasound before receiving study treatment. Recording of at least 2 dimensions (longest diameter x greatest perpendicular diameter) of the primary tumour will be performed and presence, number and size (not mandatory) of lymph nodes will be evaluated. After 4 cycles of treatment the same measurements will be taken, to establish whether the subject has responded, remained stable or progressed according to WHO criteria.

For the secondary endpoint:

Subjects will be staged according to ultrasound (mandatory) and MRI (as additional option in case mandated by local guidelines before receiving study treatment). Recording of at least 2 dimensions (longest diameter x greatest perpendicular diameter) of the primary tumour will be performed and presence, number and size (not mandatory) of lymph nodes will be evaluated. After 4 cycles of treatment the same measurements will be taken.

In order to calculate the RCB, the surgical material will be used and analysed according to the following parameters:

- Primary tumour bed dimensions
- Cellularity fraction of invasive cancer
- Size of largest positive lymph node
- Number of positive lymph nodes

In order to evaluate Genomic Grade Index (GGI), RNA-seq will be performed on baseline and residual tumour at surgery, testing for the 97 genes that compose the GGI. In order to evaluate the achievement of complete cell cycle arrest (CCA), Ki67 score will be centrally reviewed on residual tumour at surgery.

8.2. Methods for assessing efficacy parameters

The main method for evaluation will be at least bi-dimensional (longest diameter x greatest perpendicular diameter of primary tumour) ultrasound before and after study treatment. Should any participating centres use both ultrasound and MRI of the breast as part of standard evaluation, MRI results (longest diameter x greatest perpendicular diameter of primary tumour) will also be assessed for secondary end-point.

Response criteria will be those of the World Health Organization (WHO) as evaluated through the above-mentioned imaging tests. Possible outcomes are as follows:

(1) Complete Response: Disappearance of the primary tumour. Any pathological lymph nodes (whether target or non-target) must have reduction in short axis to <10 mm.

(2) Partial Response: At least a 50% decrease in the surface of target lesion (primary tumour), taking as reference the baseline surface.

(3) Stable Disease: Neither sufficient shrinkage to qualify for partial response nor sufficient increase to qualify for progressive disease, taking as reference the starting surface.

(4) Progressive Disease: At least a 25% increase in the surface of target lesion (primary tumour), taking as reference the baseline surface on study. (Note: the appearance of one or more new lesions is also considered progression).

Though scheduled evaluations only include beginning and end of study treatment, should signs of progressions (signs or symptoms) be found during routine clinical evaluation subjects may be evaluated with imaging at investigator discretion according to local guidelines.

For biomarker analysis using RNA-seq we will define "responders" as subjects that have either a complete or partial response to treatment, and "non-responders" as subjects that have either stable or progressive disease. We will also perform exploratory biomarker analysis by correlating tumour response by ultrasound or magnetic resonance (optional) imaging (response will be assessed as continuous or categorical variable) with RNA-seq of the baseline tumour biopsy.

8.3. Progression of disease during study

Cases of disease progression during study treatment may occur. In cases where this occurs, the study central team should be contacted immediately to discuss the case. A priori, if the progression is restricted to previously existing lesions (primary tumour and/or regional nodes), subject should come off study treatment and undergo surgery within study. In cases where this cannot occur or cases where progression is systemic (distant metastasis) conduct in relation to study will be defined on a case by cases basis.

9. ASSESSMENT OF SAFETY

9.1. Safety parameters and Methods for assessing safety parameters

The following safety parameters will be assessed:

- Demographic data (for details please refer to the paragraph "Schedule of Assessments")
- Medical history (for details please refer to the paragraph "Schedule of Assessments")
- Cancer history (for details please refer to the paragraph "Schedule of Assessments")
- Prior cancer treatment (for details please refer to the paragraph "Schedule of Assessments")
- Concomitant medications (for details please refer to the paragraph "Schedule of Assessments")
- Physical examination and vital signs (for details please refer to the paragraph "Schedule of Assessments")
- ECOG performance status score (for details please refer to the paragraph "Schedule of Assessments")
- Laboratory tests (for details please refer to the paragraph "Schedule of Assessments")
- Test procedures (for details please refer to the paragraph "Schedule of Assessments")

- Adverse Events (AEs) (for details please refer to the paragraph “Pharmacovigilance parameters definitions and reporting guidance”)
- Adverse Event of Special Interests (AESIs) (for details please refer to the paragraph “Pharmacovigilance parameters definitions and reporting guidance”)
- Serious Adverse Events (SAEs) (for details please refer to the paragraph “Pharmacovigilance parameters definitions and reporting guidance”)
- Pregnancy, lactation (for details please refer to the paragraph “Pharmacovigilance parameters definitions and reporting guidance”)
- Overdose, abuse, misuse, medication error (for details please refer to the paragraph “Pharmacovigilance parameters definitions and reporting guidance”)
- Exposure to the study medications during pregnancy, exposure to the study medications during lactation, and occupational exposure to the study medications by non-study subjects.

9.2. Pharmacovigilance parameters definitions and reporting guidance

9.2.1. Adverse Events

9.2.1.1. *Definitions*

An ***adverse event (AE)*** is any untoward medical occurrence in a subject or clinical investigation subject receiving/undergoing the study treatments [Investigational medicinal products (palbociclib, tamoxifen, goserelin and letrozole) and protocol surgery] and which does not necessarily have a causal relationship with these study treatments.
An AE can therefore be any unfavourable and unintended sign (including an abnormal laboratory finding, for example), symptom or disease temporally associated with the study treatments, whether or not considered related to them.

An ***adverse reaction (AR)*** is any untoward and unintended response to an investigational medicinal product related to any dose administered and/or to the protocol surgery.

This definition covers also medication errors and uses outside what is foreseen in the protocol, including misuse and abuse of the product.

This definition implies a reasonable possibility of a causal relationship between the event and the investigational medicinal product (IMP)/protocol surgery.

An ***unexpected adverse reaction (UAR)*** is an adverse reaction, the nature or severity (intensity) of which is not consistent with the applicable product information (e.g. investigator’s brochure for an unauthorised investigational product or summary of product characteristics for an authorised product, protocol for surgery).

Remarks:

- Pre-existing conditions which worsen during a study are to be reported as AEs.
- Abnormal laboratory findings (e.g. haematology, haemostatic function, blood chemistry and serology) or other abnormal assessments (e.g. vital signs) that are judged by the principal investigator as clinically significant have to be recorded as

AEs. The clinical term rather than the laboratory term should be recorded (e.g. hyperkalaemia vs high potassium).

9.2.1.2. *AE intensity*

Intensity of all AEs will be graded according to the NCI-CTCAE version 4.03 on a five-point scale (Grade 1 to 5) and reported in detail on the CRF. Adverse events not listed in CTCAE should be graded as listed in Table 9.

Table 9: Grading for Adverse Events not listed in NCI-CTCAE

CTC Grade	Equivalent To:	Definition
Grade 1	Mild	Asymptomatic or mild symptoms; clinical or diagnostic observations only; intervention not indicated
Grade 2	Moderate	Minimal, local or non-invasive intervention indicated; limiting age-appropriate instrumental activities of daily living (ADL)*
Grade 3	Severe	Severe or medically significant but not immediately life threatening; hospitalization or prolongation of hospitalization indicated; disabling; limiting self-care ADL**
Grade 4	Life-threatening/disabling	Life-threatening consequences; urgent intervention indicated
Grade 5	Death	Death related to AE

*Instrumental ADL refer to preparing meals, shopping for groceries or clothes, using the telephone, managing money etc.

**Self-care ADL refer to bathing, dressing and undressing, feeding self, using the toilet, taking medications, and not bedridden.

9.2.1.3. *Relationship to study treatments*

The causality relationship of the AE to study therapy will be assessed by the principal investigator as either: related, unrelated.

If there is a reasonable suspected causal relationship to study treatment, i.e., there are facts (evidence) or arguments to suggest a causal relationship, treatment-event relationship should be assessed as **Related**.

The following criteria should be considered in order to assess the relationship as **Related (Reasonable possibility):**

- Reasonable temporal association with study treatment.
- It may or may not have been produced by the subject’s clinical state, environmental or toxic factors, or other modes of therapy administered to the subject.
- Known response pattern to study treatment.

- Disappears or decreases on cessation or reduction in dose.
- Reappears on re-challenge.

The following criteria should be considered in order to assess the relationship as **Unrelated (No reasonable possibility)**:

- It does not follow a reasonable temporal sequence from administration of study treatment.
- It may readily have been produced by the subject's clinical state, environmental or toxic factors, or other modes of therapy administered to the subject.
- It does not follow a known pattern of response to the study treatment.
- It does not reappear or worsen when the drug is re-administered.

9.2.1.4. *Reporting of adverse events*

From the first administration of study medications until 30 days after the protocol surgery, all AEs should be reported on the AE page of the CRF.

All AEs will be followed until one of the following occurs:

- Complete resolution
- Return to baseline condition
- Event is considered by the principal investigator to be unlikely to resolve
- Death
- Subject end of study

The final outcome of each AE must be recorded in the CRF.

The principal investigator has to follow-up any AEs that are not resolved after the subject's end of study visit as long as medically indicated but without further recording in the CRF.

Note: Any abnormal laboratory test result and any abnormal assessment (e.g. vital signs) result reported as AE should be followed until it has returned to the normal range, baseline value (value obtained during the screening) and/or an adequate explanation of the abnormality is found.

9.2.2. Serious Adverse Events

9.2.2.1. *Definitions*

A ***serious adverse event (SAE)*** or ***reaction (SAR)*** is any untoward medical occurrence at any dose results in any of the following outcomes:

- Death;
- Life-threatening;
- In-subject hospitalisation or prolongation of existing hospitalisation;
- Persistent or significant disability/incapacity;

- Congenital anomaly/birth defect;

Medical and scientific judgement should be exercised in deciding whether expedited reporting is appropriate in other situations, such as important medical events that may not be immediately life-threatening or result in death or hospitalisation but may jeopardise the subject or may require intervention to prevent one of the other outcomes listed in the definition above. These should also usually be considered serious (medically significant condition) (e.g. secondary primary malignancy).

The term “serious” is not synonymous with the term “severe”. The term "severe" is often used to describe the intensity (severity) of a specific event (as in mild, moderate, or severe myocardial infarction); the event itself, however, may be of relatively minor medical significance (such as severe headache). While the term "serious” is based on subject or event outcome or action criteria usually associated with events that pose a threat to a subject's life or functioning. Seriousness (not severity) serves as a guide for defining regulatory reporting obligations.

A ***suspected unexpected serious adverse reaction (SUSAR)*** is a suspected adverse reaction that is both unexpected and serious and is subject to expedited reporting. The expectedness assessment of a serious adverse reaction is done by the sponsor by using the Reference Safety Information.

In this study, the expectedness assessment will be performed by using the Reference Safety Information within:

- Summary of product characteristics for palbociclib.
- Summary of product characteristics for letrozole.
- Summary of product characteristics for tamoxifen.
- Summary of product characteristics for goserelin

Remarks:

- Death is an outcome and not an event therefore the CTCAE event(s) that caused the death should be reported as a SAE(s) except in case of sudden death (An unexpected cessation of life that cannot be attributed to a CTCAE term associated with Grade 5) or death of unknown origin.
- The term "Life-threatening" refers to an event in which the subject was at risk of death at the time of the event; it does not refer to an event which hypothetically might have caused death if it were more severe.
- An in-subject hospitalisation is a hospital stay equal or greater than 24 hours.
- Related second primary malignancies (SPM) [i.e. a cancer judged by the investigator to be related to palbociclib and/or letrozole and/or tamoxifen and/or goserelin] are to be reported as SAEs at any time regardless of the time elapsed since the last administration of study treatments.
- Progression of disease under study is reportable SAE if the subject dies of disease progression within the active reporting period.

9.2.2.2. *Reporting of serious adverse events*

From informed consent (IC) signature until initiation of the first study medications all SAEs related to a protocol-mandated intervention (e.g., SAEs related to invasive procedures such as biopsies) should be reported on a SAE form only.

All SAEs from the first administration of the study medications and up to 30 days after the protocol surgery must be collected and reported on a SAE form and on the AE page of the CRF.

After this period, only SAEs which have a reasonable possibility to be related to the study treatments (study medications or protocol surgery) (even if the study has been closed) must be reported on SAE form only.

All SAEs will be followed until one of the following occurs:

- Complete resolution
- Return to baseline condition
- Event is considered by the principal investigator to be unlikely to resolve
- Death
- End of trial

Note: Any abnormal laboratory test result and any abnormal assessment (e.g. vital signs) result reported as SAE should be followed until it has returned to the normal range, baseline value (value obtained during the screening) and/or an adequate explanation of the abnormality is found.

Table 10: SAE reporting

From IC signature up to 1st study medication administration	SAEs related to protocol-mandated intervention
From 1st study medication administration up to 30 days after protocol surgery	All SAEs
From day 31 after protocol surgery	Only SAEs related to study treatments (study medications or protocol surgery)

All SAEs must be reported to the sponsor immediately and in any case no later than 24 hours from the time the principal investigator or study staff member became aware of the event on the study-specific SAE form in English.

The SAE report has to be sent to the fax number or email address mentioned on the study-specific SAE form.

Any additional documents in local language have to be provided with an English translation of the relevant information.

To be compliant with regulatory reporting requirements, all initial SAE forms must contain the following mandatory information: one identifiable subject, one identifiable reporter, one adverse event, one seriousness criteria, the relationship to study medication, outcome at last observation. Within 7 calendars days, the principal investigator must provide complete information requested on the SAE form of any reported serious adverse event. If the completed form is not received within this deadline, the sponsor will issue queries to the principal investigator.

Any queries on the SAEs sent out by the sponsor need to be answered within 7 days.

All SAE forms need to be reviewed, dated and signed by the principal investigator or medical physician as specified in the study task delegation log.

The principal investigator should report SAEs that occur at their institution to their respective ethics committee according to the local committee's policies.

The SAE reporting and the SUSAR reporting will be in accordance with EU Directive 2001/20/EC and the CT-3 guidance on the collection, verification and presentation of adverse event/reaction reports arising from clinical trials on medicinal products for human use (2011/C 172/01). The sponsor will be responsible of the reporting of the SUSARs to the EudraVigilance Clinical Trial Module (EVCTM), the Competent Authorities, the Ethics Committees and the participating investigators.

9.2.3. Exceptions to AEs/SAEs reporting

Some hospitalisation scenarios do not require reporting as a SAE such as:

- Elective hospitalisation for pre-existing conditions that have not been exacerbated by study medication.
- Hospitalisation planned before the subject consented for study participation and where admission did not take longer than anticipated.
- Hospitalisations for reasons described in the protocol (e.g. hospitalisation for study medication administration, hospitalisation for study related procedures). However, event requiring hospitalisation or prolongation of hospitalisation as a result of a complication of study medication administration or study related procedures will be reported as SAE.
- Hospitalisation or prolonged hospitalisation in absence of an AE (social, technical, practical reason and/or convenience admission to a hospital, palliative care, rehabilitation).

Recurrence and/or progression of underlying malignancy is not reported as an AE or SAE if it is clearly consistent with the suspected recurrence or progression of the underlying cancer but will be reported on the CRF, except if subject died due to progression of underlying malignancy within the active reporting period.

Unrelated SPM [i.e. a cancer that is unrelated to the study treatment(s) and is not a metastasis from the initial malignancy] should not be reported as an AE or SAE but is has however to be reported on the CRF.

Hospitalisation due solely to the recurrence and/or progression of underlying malignancy should NOT be reported as an SAE.

Clinical symptoms of recurrence and/or confirmed progression of underlying malignancy should not be reported as AEs or SAEs unless the symptoms cannot be determined as exclusively due to the recurrence or confirmed progression of the underlying malignancy, or does not fit the expected pattern of recurrence or confirmed progression for the disease under study.

9.2.4. Adverse Event of Special Interest

9.2.4.1. *Definitions*

An ***adverse event of special interest*** (serious or non-serious) ***(AESI)*** is one of scientific and medical concern specific to the sponsor's product or programme, for which ongoing monitoring and rapid communication by the principal investigator to the sponsor could be appropriate. Such an event might require further investigation in order to characterise and understand it. Depending on the nature of the event, rapid communication by the trial sponsor to other parties (e.g., regulators) might also be warranted.

In this study, abnormal values in aspartate aminotransferase (AST) and/or alanine aminotransferase (ALT) levels concurrent with abnormal elevations in total bilirubin level that meet the criteria outlined below in the absence of other causes of liver injury are considered potential cases of drug-induced liver injury (potential Hy's law cases) and should always be considered as adverse event of special interest.

The threshold of laboratory abnormalities for a potential case of drug-induced liver injury depends on the subject's individual baseline values and underlying conditions. Subjects who present with the following laboratory abnormalities should be evaluated further to definitively determine the etiology of the abnormal laboratory values:

- Subjects with AST or ALT baseline values within the normal range who subsequently present with AST or ALT values ≥ 3 times the upper limit of normal (x ULN) concurrent with a total bilirubin value ≥ 2 x ULN with no evidence of hemolysis and an alkaline phosphatase value ≤ 2 x ULN or not available;

- Subjects with pre-existing AST or ALT baseline values above the normal range who subsequently present with AST or ALT ≥ 2 x the baseline values and ≥ 3 x ULN, or ≥ 8 x ULN (whichever is smaller) concurrent with a total bilirubin ≥ 2 x ULN and increased by 1 x ULN over baseline or ≥ 3 x ULN (whichever is smaller) with no evidence of hemolysis and an alkaline phosphatase ≤ 2 x ULN of normal or not available.

All cases confirmed on repeat testing as meeting the laboratory criteria defined above, with no other cause for liver function test (LFT) abnormalities identified at the time, should be considered potential Hy's Law cases irrespective of availability of all the results of the investigations performed to determine etiology of the abnormal LFTs. Such potential Hy's law cases should be reported as adverse event of special interest.

9.2.4.2. *Reporting of adverse events of special interest*

Any adverse event of special interest occurring during the study medication period and up to 30 days after the last administration of study medications must be reported to the sponsor on the SAE form and on CRF.

After this period, only AESIs which have a reasonable possibility to be related to study medications (even if the study has been closed) must be reported on the SAE form.

All AESIs must be reported immediately and no later than 24 hours from the time the principal investigator or an authorised staff member (i.e. as specified in the study task delegation log) became aware of the event to the sponsor on the study-specific SAE form in English. The report has to be sent to the fax number or email address mentioned on the Serious Adverse Event form.

9.2.5. Pregnancy

A female subject who becomes pregnant during the study must be instructed to stop taking study medications and immediately inform the principal investigator.

The principal investigator or an authorised staff member (i.e. as specified in the study task delegation log) should notify any pregnancy diagnosed in a female subject during the study medications and within 30 days after last study medications administration, immediately and no later than 24 hours from the time of awareness to the sponsor by using the pregnancy notification form. The pregnancy report has to be sent to the fax number or email address mentioned on the Pregnancy form.

The principal investigator should counsel the subject, and discuss the risks of continuing with the pregnancy and the possible effects on the foetus. The principal investigator should follow-up with the subject until conclusion of the pregnancy and should also monitor the development of the newborn and report any abnormality in the newborn within 30 days after the delivery.

If the involved parties sign the specific Informed Consent for data collection in case of pregnancy, the principal investigator should report the outcome of the pregnancy and report any abnormality in the newborn within 30 days after the delivery as a follow-up to the initial pregnancy notification to the sponsor. While the pregnancy itself is not considered to be an AE or SAE, any pregnancy complication, any spontaneous abortion, any abnormality in newborn and any child death (intrauterine death, stillbirth, post-delivery death) will be recorded as an AE or SAE (if any of the criteria for seriousness is met).

9.2.6. Exposure during lactation

An exposure during lactation occurs if an infant or child may have been exposed through breast milk to the study medication during lactation by a female subject.

The principal investigator or an authorised staff member (i.e. as specified in the study task delegation log) should notify any lactation occurring during the study medication administration to a female subject, immediately and no later than 24 hours from the time of awareness to the sponsor by using the lactation notification form in English. The lactation report has to be sent to the fax number or email address mentioned on the Lactation Notification form.

9.2.7. Overdose, misuse and abuse

9.2.7.1. *Definitions*

Overdose: this refers to the administration of a quantity of a medicinal product given per administration or cumulatively which is above the maximum recommended dose according to protocol. Clinical judgement should always be applied.

Misuse: this refers to situations where the medicinal product is intentionally and inappropriately used not in accordance with the protocol.

Abuse: This corresponds to the persistent or sporadic, intentional excessive use of a medicinal product, which is accompanied by harmful physical or psychological effects.

9.2.7.2. *Reporting of overdose, misuse and abuse*

The principal investigator or an authorised staff member (i.e. as specified in the study task delegation log) should report any adverse reaction due to overdose, misuse and abuse, occurring in a subject during the study medication administration period immediately and no later than 24 hours from the time of awareness to the sponsor by using the appropriate notification form. The report has to be sent to the fax number or email address mentioned on the notification form.

9.2.8. Medication Error

9.2.8.1. *Definition*

Medication error: this refers to any unintentional error in the prescribing, dispensing, or administration of a medicinal product while in the control of the healthcare professional, subject.

9.2.8.2. *Reporting of medication error*

The principal investigator or an authorised staff member (i.e. as specified in the study task delegation log) should report any adverse reaction due to medication error, occurring in a subject during the study medication administration period immediately and no later than 24 hours from the time of awareness to the sponsor by using the appropriate notification form. The report has to be sent to the fax number or email address mentioned on the notification form.

9.2.9. Reporting of exposure to study medications by non-study subjects

The principal investigator or an authorised staff member (i.e. as specified in the study task delegation log) should notify any exposure to the study medications during pregnancy, exposure to the study medications during lactation, and occupational exposure to the study medications by non-study subjects during the study medications administration period immediately and no later than 24 hours from the time of awareness to the sponsor by using the appropriate notification form. The report has to be sent to the fax number or email address mentioned on the notification form.

10. TRANSLATIONAL RESEARCH(ES)

Preclinical studies using breast tumour cell lines [50,51,52], mouse models [53] and ex vivo subject tumour cells [54] have shown the usefulness of CDK4/6 inhibition by palbociclib to durably arrest proliferation and induce tumour cell senescence. Resistance to this drug is mainly attributed to pRb loss [55,56]. Palbociclib combined with endocrine treatment improved progression-free survival compared to endocrine treatment alone in women with ER-positive/HER2-negative advanced breast cancer [57,58,59,60,61].

However, there are no clinically validated biomarkers of resistance to CDK4/6 inhibitors [62,63].

The main aim of the present study is to identify biomarkers of resistance to a 4-month preoperative treatment of palbociclib and endocrine therapy given prior to surgery.

In order to do so, we will collect pre-treatment breast tumour biopsy (2 FFPE and 2 frozen cores). The biopsies must be obtained for all subjects before start of the study treatment. In addition, FFPE and Frozen samples (at least one of each and maximum 2 cores each) must be collected from surgical material left over after sufficient and relevant part has been retained to establish, improve or complement the diagnosis or treatment of the subject.

In addition, one whole blood sample (10 mL EDTA) will be collected prior to Day 1 Cycle 1 of the study treatment for isolation of normal DNA to serve as control during our analyses.

Blood sample (4 x 10 mL EDTA per time point) will be also collected for plasma isolation prior to Day 1 Cycle 1, prior to Day 1 Cycle 2 of study treatment, at the time of surgery and one month after the surgery.

We will perform whole genome (WGS) and RNA sequencing on the pre-treatment frozen biopsy cores and surgical material for all the subjects.

WGS is the platform offering the most comprehensive and unbiased examination of the cancer genome and leads to the discovery of novel mutations or classifications, copy number estimation, mutational signatures, structural variants and chromosomal rearrangements, amongst many other things.

RNA sequencing offers a precise measurement of transcripts (including mRNA, non-coding RNAs, small RNAs). Compared with current microarrays it offers better quantification and larger dynamic range as it provides the absolute number of transcripts. Importantly, somatic mutations, gene fusions and chromosomal rearrangements can also be detected with this technology.

Batched RNA sequencing will be performed at the end of recruitment period on all subjects for whom the frozen biopsy cores and left over surgical material are evaluable in terms of tumour content and RNA quantity. Due to the fast evolution within this field, the actual analytical methodology will only be specified and decided upon at the time these analyses will start.

Using RNA sequencing data generated during the study, we can test any gene signature that is publicly available today or might become available.

Tumour derived DNA and Plasma ctDNA extractions will be performed at the end of recruitment period on all subjects. Specific mutations genes will be identified by high coverage NGS in primary tumour (pre-treatment biopsy). Mutations identified using this approach will be validated using ddPCR. Analysis of normal DNA using NGS will also be performed at the same time to exclude germline variants. Moreover mutation(s) (specific per subject) identified by NGS in the primary tumour will then be followed in plasma cell-free DNA using the best available technology. Analysis of normal DNA will also be performed at the same time. We will also evaluate the possibility to detect point mutations directly in plasma ctDNA using high coverage NGS. Mutations identified using this approach will be orthogonally validated.

Moreover, recent data in breast cancer mouse models have suggested that CDK4/6 inhibitors enhance antitumor immunity. However, these intriguing data are only supported by scarce data on human samples. In the present study, we aim to comprehensively study immune response before and after 4 months of neoadjuvant hormonotherapy and CDK4/6 inhibitors. Antitumour immune response will be assessed using several immunohistochemistry and/or Fluorescent Multiplex Immunohistochemistry (IF-IHC) in pre- and post-treatment tumour samples to further characterize changes intumour infiltrating lymphocytes (TILs) and more specifically in T and B cell subpopulations as well as other populations as macrophages following pre-operative treatment with endocrine therapy and palbociclib.

TILs will be assessed on the available H&E slides from FFPE pre and post treatment tumour samples. Markers, including but not restrictive to CD3, CD4, and CD8 for major T cell subpopulations; CD25, ICOS and PD-1 for activated T cell subpopulations; CD25, FOXP3, IL10 and TGFβ for regulatory T cells; CD19/CD20, CD38, IgD, for B cell subpopulations; CD21/CD35 for follicular dendritic cells, CD68 for tumour-associated macrophages, panCK for tumour cells and Ki67 for proliferating cells will be assessed by IHC or IF-IHC on FFPE pre and post treatment tumour samples. In addition, several specific immune gene signatures will be investigated using the generated RNA sequencing data.

Markers of senescence including but not restrictive to β-galactosidase activity will also be investigated using IHCs on FFPE pre and post treatment tumour samples. Tumour proliferation, as measured by the Ki67 score[58], will be assessed using IHCs on FFPE pre and post treatment tumour samples.

As an exploratory objective, we will perform a combined genomic and transcriptomic (G&T) single tumor cell analysis and chromatin accessibility single tumor cell analysis of frozen tumour samples from exceptional responders and exceptional non-responders.

10.1. Timelines

	Screening	Pre-treatment	Treatment Period						End of study
**Time points**	**Prior to Inclusion**	**Prior to Treatment Start**	**First Cycle**	**Second Cycle**	**Third Cycle**	**Fourth cycle**	**Bridge period (3-7 days prior surgery)**	**Surgery**	**1 month post-surgery (end of study)**
**Day of Study**			D1	D29	D57	D85	D113 - D119	D116-D120	D146-D150
**Assessments**									
Tissue Collection (2FFPE + 2 Frozen)		X [(1)]						X	
Blood for Plasma processing (4x10 ml)		X	X [(2)]					X	X
Whole Blood (1x10 ml)		X							

(1) If 4 biopsies are already available for submission (2 FFPE and 2 Frozen), which were obtained outside the study due to the Site's standard clinical practice, there is no need to repeat these biopsies after the subject's inclusion in the study; these biopsies must have been obtained up to 6 weeks before the beginning of treatment.

(2) on C1 day 28

10.2. Biological material handling

Standardised protocols will be applied consistently in preparing and storing biological samples to ensure quality and to avoid introducing confounders into research studies. See also the National Cancer Institute (NCI) best practice for biospecimens resource (http://biospecimens.cancer.gov/bestpractices/), the Recommendations for Collection and Handling of Specimens from Group Breast Cancer Clinical Trials (reference) and the BIG movie on improving translational research samples collection in international trials (available on http://www.youtube.com/user/BreastIntGroup).

Kits will be provided to all participating sites in order to perform biological samples collection. The kits will be provided by a central service provider located in Belgium.

Further details on collection, handling, local storage and shipping will be provided in a separate laboratory instruction manual.

10.3. Central biobanking of biological material collected during the trial and Destruction

Biological samples, material left over and/or derivatives (after molecular analyses embedded in the current trial have been performed) will be stored securely, for a minimum of 20 years or until exhaustion or for the maximum time allowed by local regulations or until a request is made for the biological samples to be returned or destroyed. Some of this biological material may be shipped for further analyses if necessary to other academic institutions or for-profit organizations.

To protect the subject's identity and privacy, the biological samples will be double-coded. First they are labelled with a unique study number or 'code' and this code does not carry any personal identifiers. Then the biological samples are relabelled with a second code, which is linked to the first code via a second coding key. The use of the second code provides additional confidentiality and privacy protection over the use of a single code. Access to both coding keys is needed to link any data or biological samples back to a subject identifier. In general, the principal investigator is responsible for maintaining the first coding key and does not have access to the second coding key. As the biological samples and associated data can be traced back indirectly via the use of both coding keys, it may be possible to undertake actions such as sample withdrawal. The principal investigator, his/her staff or those working with them (for example, other researchers) will only use the biological samples for the purpose stated in the current trial in accordance with the terms outlined in the Informed Consent Form. The principal investigator and his/her staff will require anyone who works with the biological samples to agree to hold the research information and any individual results in confidence.

Subjects may request that their biological samples as well as their material left over and/or derivatives be removed from storage and destroyed or returned to their institution in accordance with the terms outlined in the Informed Consent Form. Requests for destruction or return of the biological samples should be made in writing to the principal investigator. In turn, the principal investigator must promptly inform the Sponsor so that appropriate follow-up can be initiated. The principal investigator will be informed when the biological material is destroyed or returned and should notify the subject that biological material destruction is complete or that biological material is stored back in the institution.

Please note any molecular data that is already generated at time of biological material destruction or return request will be used for the purposes of the study. Please also note

that specific molecular and clinical data (generated within the study or future research (see below)) could be put on secured database accessible via internet. This can be done for the purpose of data sharing or for publication needs. Data will be double coded and no reference to subject name will be made. Accession to this data will be made available to any individuals making a specific request and following data access policy rules (specific per database) previously agreed by the sponsor.

Further details on the legal entity acting as data controller, the legal entity acting as data processor and the medical doctor responsible for the Belgian biobank will be provided in a separate laboratory instruction manual.

10.4. Future undefined research

Data and biological samples as well as material left over and/or derivatives (after molecular analyses embedded in the current trial have been performed), may be used for undefined future research. Proposals for access to such samples and data will have to be approved by the study team as well as the appropriate Ethics Committee(s). Access to any biological samples as well as material left over and/or derivatives thereof for future research is only possible if subject consented to the use of the biological material for future undefined research. Electronic documents to keep track of biological samples collection will be kept in participating sites and at study sponsor. These documents will contain the subject ID code with no reference to subject name, initials and address. Some of this biological material may be shipped for further analyses if necessary to other academic institutions or for-profit organizations.

11. STATISTICAL CONSIDERATIONS

11.1. Statistical design

11.1.1. Sample size

The study primary objective is to develop a gene signature to predict resistance to neoadjuvant treatment of endocrine therapy and palbociclib using RNA sequencing of the baseline tumour biopsy. The following few assumptions are available:

The overall response rate will be around 75%-80% (8)

The resistance rate is therefore around 20%-25%

According to simulations carried out by Dobbin and Simon a sample size of at least 20 subjects in each class is required to develop a binary predictor using high-dimensional data (9).

Therefore to get 20 subjects in the resistant group with an expected 22.5% resistance rate, around 90 evaluable subjects are needed.

To take into account a rate of 10% of non-evaluable subjects, 100 eligible subjects will be required for inclusion in the study. A further 10 subjects are added to this number in order to take into account subjects who will sign informed consent (registered in the study) without being included in the study.

We will perform an interim analysis on data from the first 65 enrolled patients (2/3 of the 100). The interim analysis will not focus on the primary objective, but on selected secondary

objectives (2.1.2.2., 2.1.2.3., 2.1.2.4., 2.1.2.5., 2.1.2.9., 2.1.2.10., and 2.1.2.11.) and on the exploratory objective. This analysis will have no impact on the course of the trial.

The whole sample size will be used for predictor development and a further validation set will be required.

11.1.2. Study duration

- Recruitment period: 33 months
- Treatment period for a subject: 4 months
- Follow-up period (post-surgery follow up) : 1 month
- Data Cleaning: 6 months
- Biological sample analysis: 12 months

11.2. Statistical plan

A descriptive analysis will be performed on all the eligible subjects (baseline subjects and tumour characteristics).

Treatment administration (duration, delays, dose reductions) will be descriptively reported on all eligible subjects having received at least one dose of treatment.

Safety analysis will be descriptive (tabulation of occurrence of grades I-II/III-IV overall and related to treatment) and will be done on the same subject population.

Resistance to treatment using the different definitions will be reported as estimated proportions with 95% confidence intervals on the populations of evaluable subjects. A subject will be considered as evaluable if the baseline and after treatment required examinations have been done for assessment and when all the elements needed to assess resistance or not will be available. Sensitivity analysis will be done considering that all non-evaluable subjects are resistant.

The search for molecular signatures predicting resistance will be done on evaluable subjects only. Differences between resistant subjects and responding subjects will be assessed using t-tests or Wilcoxon tests Adjustment for multiplicity testing will be done using the Benjamini-Hochberg method. False discovery rate will be set to 5%. The signature for resistance will be derived using logistic regression with stepwise variable selection. The characteristics of the signature will be evaluated.

Other publicly available signatures might be also tested for their performance related to the study endpoints.

11.3. End of study

The end of study will be declared when all the following criteria will have been met:

- After last visit of the last subject (one month after surgery day of last subject)
  - The trial is mature for the analysis of all the endpoints as defined in the protocol, if the trial reaches its endpoints
  - The database has been fully cleaned and frozen for all analyses

12. DIRECT ACCESS TO SOURCE DATA/DOCUMENTS

The participating site and the principal investigator shall allow the monitors, the employees of the Sponsor (provided that subject confidentiality is protected) or persons subcontracted by the Sponsor responsible for auditing, the representatives of the Ethics Committees, and of the Competent Authorities/ Regulatory agencies to have direct access to source data / documents/ complete study records.

13. QUALITY CONTROL AND QUALITY ASSURANCE

13.1. Quality control

Throughout the study the dedicated sponsor study team members will verify the data to ensure that:

- the rights and well-being of subjects are protected
- the reported trial data are accurate, complete and verifiable from source documents
- the conduct of the trial is compliant with the IHC-GCP, the applicable regulatory requirements, the study protocol and the study guidelines.

Quality control activities combine central monitoring and clinical site monitoring. Monitoring activities may be performed remotely and/or on site according to the study needs.

13.2. Quality assurance

To ensure compliance with the protocol, CRF completion guidelines, study documentation, SOPs, GCP and all applicable regulatory requirements, the Sponsor may conduct a quality assurance audit on participating site. Regulatory agencies may also conduct a regulatory inspection of the study. The principal investigator must inform the Sponsor in case an Inspection from Regulatory agencies has been scheduled at their sites

Such audits/inspections can occur at any time during or after completion of the study. If an audit or inspection occurs, the investigator and institution must agree to allow the auditor/inspector direct access to all relevant documents and to allocate his/her time and the time of his/her staff to the auditor/inspector to discuss findings and any relevant issues in the presence of the sponsor, as needed or required.

14. REGULATORY AND ETHICAL CONSIDERATIONS

14.1. Regulatory considerations

The study will be submitted by the principal investigator(s) or the national coordinator(s) or the Sponsor (or its legal representative), in accordance with local regulations, to and approved by an appropriate Independent Ethical Review Committee (IEC)/ Institutional Review Board (IRB) and a Regulatory Authority if required by the national laws of the countries where the study will be conducted. Local regulatory approval may also be required.

The study will not start in a participating site before written approval by corresponding Ethics Committee(s) has been obtained and the local regulatory requirements have been complied with.

The Sponsor will provide a copy of the final protocol, subject information sheets, consent forms and all other relevant study documentation for local required submissions.

The principal investigator and the Sponsor will ensure that the study is conducted in full conformance with the principles of the "Declaration of Helsinki" 1964, as revised from time to time and with the laws and regulations of the country in which the research is conducted, whichever affords the greater protection to the individual. The study must fully adhere to the principles outlined in "Guideline for Good Clinical Practice" ICH-E6 Tripartite Guideline (January 1997) and with national laws.

For the Study conducted in the EU/EEA countries, the principal investigator will ensure compliance with the EU Clinical Trial Directive (2001/20/EC)/ICH GCP and with the EU General Data Protection Regulation (GDPR).

In other countries where "Guidelines for Good Clinical Practice" exist, the Sponsor and the principal investigators will strictly ensure adherence to the stated provisions.

14.2. Informed consent form and procedure for obtaining patient's consent

14.2.1. Informed consent form

The informed consent form (ICF) is based on the Belgian national template which is available on the web site of competent authority (AFMPS-FAGG). It is identified by a version number and a date, and it makes reference to the associated protocol (title of the clinical trial, EudraCT number, protocol number).

The ICF describes in details the following items: the investigator and the sponsor, objectives and description of the study protocol (procedures, methods, IMP/interventions), course of the study, drugs received as treatment, risks and discomforts (possible adverse events and potential hazards of the study including risks related to a pregnancy), benefits, costs, notification of new information, interest to participate in the study, alternative treatment, withdrawal of the study, treatment after stopping or at the end of the study, publication of study results, contact details at the hospital, translational research, data protection clause insurance and liability, signatures.

The ICF is translated into the national language(s) of the participating country(ies) under the responsibility of the sponsor or its authorized delegate. Then the translations must be approved by an appropriate independent ethics committee/independent review board (IEC/IRB). Once approved, the ICF and the IRB/IEC's written approval/favourable opinion of the ICF are provided by the sponsor to the principal investigators in the national language(s) of their countries.

The principal investigators are not allowed to make any changes in the approved version of the ICF unless these changes are non-substantial for the sponsor, such as but not limited to adding the hospital logo or crest, completing a contact person's phone number.

The ICF to be provided to patients shall be revised whenever important new information becomes available that may be relevant to the patient's consent. Any revised ICF shall receive the IRB/IEC's approval/favourable opinion in advance of use.

14.2.2. The procedure for obtaining patient's consent

NeoRHEA is an single country, multicenter clinical trial. The terms and conditions described below are subject to certain variations depending on the country and its national laws, but also on the hospitals where the participants will be recruited.

There will be no direct advertising for research subjects, i.e., advertising that is intended to be seen or heard by prospective subjects to solicit their participation in a study. Direct advertising includes, but is not necessarily limited to: newspaper, radio, TV, bulletin boards, posters, and flyers.

However, for the sake of transparency, a description of this clinical trial will be available at http://www.ClinicalTrials.gov (USA). The information will be also available in a similar European website, www.clinicaltrialsregister.eu.

The informed consent process can only start once the study has been given a written positive approval/favourable opinion by the IRB/IEC's and has been approved by the National Competent Authorities if required by national laws of the participating country(-ies). Also the Investigator’s site initiation process must have been completed by the Sponsor or its authorized subcontractor(s) (including a signed contract between the site and the Sponsor and/or CRO). Patients should be identified as potentially eligible by a healthcare professional that has clinical responsibility for the patient. This is to ensure medical confidentiality is adhered to. Patients will be also assessed for their potential eligibility by a healthcare professional that is authorised to recruit to the study.

Once a patient has been assessed and identified as potentially eligible for the study, the patient should be approached and informed to the study.

The person informing the patient on the study must be familiar with all aspects of the study as described in the latest IEC/IRB approved version of the protocol.

The investigator, or another member of the investigating team, should discuss the study with the patient whereby the patient is given the opportunity to understand the objectives, risks and inconveniences of the trial and the conditions under which it is to be conducted. The information should be provided to patients in both oral and written forms. The language used to inform the patient, both oral and written, should be concise, described in layman’s terms and should be understandable to the patient and impartial witness/interpreter, when applicable. The person obtaining the informed consent must have the patient’s medical notes and the current IEC/IRB approved versions of the ICF available during the discussion. Other study documents may also be used during the interview (e.g. patient diaries, study schedule sheets).

All patients receive the appropriate version of the written information and are asked to read and review it.

Usually, if the patient is considering participating in a clinical trial, she may take the consent document home to discuss with family, friend or advocate.

All prospective patients must have the cognitive ability to provide legally effective informed consent. For patients not qualified to give or incapable of giving consent, written consent must be obtained from the legal representative. The legal representative is an individual authorized under applicable law to consent on behalf of a prospective patient, to the patient’s participation in the clinical study. This person should be designated by judicial decision or by a mandate (signed and dated by the two parties). In default, the authorized person who may consent on behalf of a patient is, in decreasing order: (i) the spouse, legal cohabitant and (ii) child (adult), parent, brother or sister (adult). In the case where both the patient and her legal representative are unable to read, an impartial witness should be present during the entire informed consent discussion. An impartial witness is a person, who is independent of the study, who cannot be unfairly influenced by people involved in the study, who attends the informed consent process if the patient cannot read the ICF. After the patient and/or legal representative has/have orally consented to participation in the trial, the witness’s

signature on the form will attest that the information in the consent form was accurately explained and understood.

Neither the investigator nor any member of the study team shall coerce or unduly influence a patient to participate or to continue to participate in a study. The principal investigator or designee also explains to the patient that the participation to the study is voluntary and that the patient is free to refuse to enter the study or to withdraw from it at any time, for any reason without any impact on the patient's subsequent care. No claims shall be made, either explicitly or implicitly, that the drug, biologic or device is safe or effective for the purposes under investigation, or that the drug, biologic or device is known to be equivalent or superior to any other drug, biologic or device. It is the responsibility of the principal investigator or a person designated by the principal investigator (if acceptable by local regulations) to obtain a signed written informed consent from each potential patient prior any study related procedure being carried out. The informed consent is applicable throughout the patient's participation to the study. It is commonly accepted that patient should have a minimum of 24 hours between the date the ICF is provided to the patient and the actual date when the patient (and/or the patient's legally designated representative) signs the informed consent. The date and time the trial was discussed and the date the ICF was given to the patient must be documented in the medical notes.

The consent form must be signed prior to any study related pre-screening or screening procedures being performed. The person obtaining the patient's consent should also inform relevant team members of the treatment decision for the patient.

The written ICF must be dated and personally signed by the principal investigator or authorised sub-investigator and the patient giving consent (or patient legal representative or impartial witness).

The original copy of the signed ICF will be retained in the investigator's study file (ISF) and must be made available for monitoring, audit or inspection. A copy of the signed ICF is given to the patient.

In case of new information that might affect the patient's willingness to continue participating in the study or results in significant changes in the risk/benefit assessment, the ICF should be reviewed and updated if necessary. All patients, including those already being treated, should be informed of the new information, given a copy of the revised form, and give their consent to continue in the study.

14.3. Subject identification

The principal investigator or any authorised study member staff must assure that subjects' identity is maintained confidential and that their identities are protected from unauthorised parties. Personal medical information is always treated as confidential.

On CRFs or any other study document, subjects shall not be identified by their names or initials or social security number or patient chart number, but always by the assigned subject study identification code and their date of birth (either complete or partial as allowed by national laws) in order to avoid identification errors.

The principal investigator should create and maintain up-to-date in the Investigator's Study File (ISF) the Subject screening/enrolment/identification log. This ICH-GCP log is required for documenting chronically any screened subject, their name, their assigned study number in case of study enrolment or the reason in case of non-enrolment. A note is made in the hospital medical records that the subject is participating in the study.

The name of a subject is neither asked for nor recorded by the sponsor. If subjects' names or any other confidential subject information are included by error on copies of documents submitted to the sponsor, they are obliterated and the assigned subject study identification code added to the document by the sponsor. Moreover, the sponsor asks to the principal

investigator or any authorised study member staff to also obliterate this information on the documents that he/she has submitted.

Documents (e.g. subjects' written Informed Consent Forms) not for submission to sponsor should be maintained by the principal investigator in strict confidence.

14.4. Privacy Guarantee

Personal data

If a patient participates in the study, his/her personal data will be processed; "processing" includes the collection, storage, structuring, transmission and any other use or provision. Personal data includes the following information:

- information that identifies the patient directly (such as his/her name, address, telephone number, or health insurance number);
- the age and sex of the patient;
- information relating to the patient's state of health, including medical history;
- the patient's medical treatments and his/her response to medical treatments;
- for women who become pregnant during the study: data about the pregnancy and childbirth;
- information about the patient's biological samples and the results of analyses performed on these;
- information about the patient's medical images and the results of the evaluation of these.

There will be different groups of data:

- The data group containing all data about the patient collected at the study hospital, including information that identifies him/her directly. This data group is called "**unencoded data**" and will be kept only in the study hospital's medical records.
- There will also be groups of "**encoded data**". The principle of data encoding entails that if a patient participates in the study, a code will be assigned to him/her. It will be unique, sequential and composed of 4 digits and it will replace all directly (surname, first name) or indirectly (telephone number or other number) identifying data on documents used for data collection or biological samples collected for the study. This encoding must be applied before any transmission to a third party (the manager of the database collected here is the sponsor, i.e. Institut Jules Bordet, Brussels, Belgium).

Legal basis for the processing of patient data

The protection of patient data and the related rights are guaranteed by the General Data Protection Regulation (European Regulation 2016/679), by the law of 22 August 2002 concerning patient rights in Belgium, by the law of 30 july 2018 concerning the data privacy as well as any (new) applicable legislation in the participating countries.

Data controller of patient data

The sponsor (Institut Jules Bordet, Brussels, Belgium) is the data controller of the patient's encoded data. The investigator and the hospital are, on the one hand, subcontractors of the sponsor with regard to the processing of the patient's encoded data and, on the other hand, data controllers of the patient's unencoded data collected both as part of the study and outside it.

Consultation and use of unencoded data

Unencoded data can be consulted by the investigator and other individuals from the hospital who are working on the study or providing care to the patient. Individuals accessing the data

Confidential

are subject to professional secrecy. In addition, a limited number of the sponsor's employees, or its contractual partners, ethics committees and regulatory authorities may consult the unencoded data, but solely for the purpose of verifying that the study is being conducted properly. These persons are bound by an obligation of confidentiality.

Consultation and use of encoded data

Encoded data will be transmitted to the sponsor. The encoded data can also be transmitted to service providers on the basis of contractual agreements. In addition, the encoded data may be shared with one or more partners collaborating with the sponsor with respect to the investigational drug and the diagnostic tests associated with this study's field of research.

The aforementioned recipients may use the encoded data for the following purposes:

- to answer scientific questions;
- to learn more about the investigational drug and how this group of drugs (i.e. drugs that may act in a similar way in the body) acts;
- to learn more about cancer and related health issues, and to develop corresponding diagnostic tests;
- to contribute to the correct planning of future studies;
- to develop scientific analysis methods;
- to publish the anonymised results of the study in scientific articles or presentations and to use them for educational purposes;
- to provide the sponsor of the study with informational materials on the study, the investigational drug, cancer and related health issues and diagnostic tests.

The patient's encoded data may be transmitted to regulatory bodies (including ethics committees) for regulatory purposes, for example for reporting relevant safety information, in order to receive marketing authorisation or for discussions on the reimbursement and marketing of the drug being tested and diagnostic tests. Regulatory bodies may also use the encoded data to learn more about cancer and related health issues.

Transfer of personal data to other countries

Some recipients of the data may be located outside your country and outside the European Economic Area (United States, for example). These may be countries whose level of data protection has not been confirmed by the European Commission as adequate. In this case, security measures equivalent to the security measures required by Belgian and European regulations will be taken in order to protect patient rights in terms of data confidentiality, by entering into specific contractual agreements.

Storage of encoded data

Encoded data will be kept for at least 25 years after the end of the study. Subsequently, they may be kept for an additional period of time, for the above-mentioned scientific purposes or for any legal reason (change of obligations with regard to storage, for example).

Patient rights related to personal data

The patient may exercise the following rights related to his/her personal data:

- Request information about the processing of data about him/her; however, he/she may not be able to access some data before the end of the study, without the risk of being excluded from the study. This measure may be necessary to protect the scientific integrity of the study.
- Request the correction of the data about him/her if they are incorrect or incomplete. The patient has the right to restrict the processing of data about him/her.

- Request the transfer of his/her personal data to himself/herself or to someone else in a commonly usable format.
- Withdraw his/her consent at any time without giving a reason. The patient also has the right to end his/her participation in the study by no longer coming, without having to justify himself/herself. However, this is not withdrawal in this case. Withdrawal means that the patient is actively withdrawing from the study and withdraws consent for data processing. This shall not affect the legality of processing data about him/her based on his/her consent given before withdrawal. However, after his/her withdrawal, data about him/her will no longer be collected.
- At the same time as his/her withdrawal, the patient has the right to request the erasure of data about him/her if it is no longer necessary for the purposes of processing or if there is no other legal basis for their further processing.
- The patient also has the right to lodge a complaint concerning the way in which his/her data is processed with the supervisory authority of his/her country responsible for ensuring compliance with data protection legislation.

If the patient wishes to exercise one of his/her rights relating to data about him/her, or if the patient wishes to know more about the measures to protect his/her personal data he/she can send a request to the investigator or to the data protection officer of the study hospital.

Although the patient does not have to justify himself/herself, it could be very useful for him/her to explain to the investigator why he/she is stopping the study treatment or wants to withdraw from the study, especially if he/she has experienced discomfort. These explanations would help to guarantee a full and detailed evaluation of the investigational drug and of the study. The patient will be asked to undergo an end of treatment visit as well as a safety assessment visit (safety follow-up) with his/her investigator. It may be possible that he/she may also be invited to participate in long-term follow-up visits performed by telephone. He/she is free to refuse such follow-up at any time.

15. DATA HANDLING AND RECORD KEEPING

15.1. Investigator’s files and subject’s clinical source documents

The principal investigator must maintain adequate and accurate records to enable the conduct of the study to be fully documented and the study data to be subsequently verified. These documents should be classified into two separate categories: (1) investigator’s study file; and (2) subject clinical source documents.

The investigator’s study file should be established according to the ICH-GCP E6 and should include, among other documents, the participating site staff delegated study task log established by the principal investigator, the subject screening/inclusion and identification log and signed ICF.

Subject clinical source documents (paper or electronic) are the documents where the data are recorded for the first time and include, but are not limited to, subject hospital/clinic records; physician’s and nurse’s notes; appointment book; original laboratory reports;; consultant letters and pharmacy dispensing records.

15.2. Case Report Forms (CRF)

Data for this study will be captured by using an electronic CRF (eCRF) via a sponsor designated electronic data capture system for each subject included. The site will receive training, an eCRF completion guidelines for appropriate eCRF handling and completion and personal access code by the sponsor.

The eCRF should only be completed by designated and trained site staff and submitted electronically to the sponsor.

The principal investigator is responsible for creating and maintaining up to date the Site Staff Delegated Study Tasks Log (named Signature Log in ICH GCP) mentioning the responsible persons completing, updating and/or signing the eCRF.

Once completed, the eCRF must be reviewed and electronically signed by the principal investigator or an authorised delegate from the study staff. This also applies to records for those subjects who fail to complete the study. If a subject withdraws from the study treatment or does not complete the follow-up, the reason must be noted in the eCRF. If a subject withdraws from the entire study, the withdrawal of consent should be noted in the eCRF. The principal investigator should ensure the accuracy, completeness, legibility and timeliness of the data reported in the eCRF and in all required reports. Data reported in the CRF must be derived from source documents and should be consistent with the source documents.

An audit trail will maintain a record of the initial entries and changes made, reasons for change (if necessary), time and date of entry, and user name of the person adding or changing an entry.

15.3. Retention of documents

Following closure of the study, the principal investigator must maintain all site study records, except for those required by local regulations to be maintained by someone else, in a safe and secure location. The records must be maintained to allow easy and timely retrieval, when needed (e.g., audit or inspection), and, whenever feasible, to allow any subsequent review of data in conjunction with assessment of the facility, supporting systems, and staff. Where permitted by local laws/regulations or institutional policy, some or all of these records can be maintained in a format other than hard copy (e.g., microfiche, scanned, electronic); however, caution needs to be exercised before such action is taken. The principal investigator must assure that all reproductions are legible and are a true and accurate copy of the original and meet accessibility and retrieval standards, including re-generating a hard copy, if required. Furthermore, the principal investigator must ensure there is an acceptable back-up of these reproductions and that an acceptable quality control process exists for making these reproductions.

The Sponsor will inform the principal investigator of the time period for retaining these records to comply with all applicable regulatory requirements. The minimum retention time will meet the strictest standard applicable to that participating site for the study, as dictated by any institutional requirements or national laws or regulations; otherwise, the retention period will default to 20 years after the completion of the study and/or 2 years after approval by relevant Health Authorities, whichever is longer.

Should the principal investigator wish to assign the study records to another party or move them to another location, the Sponsor must be notified in advance. If the principal investigator cannot guarantee this archiving requirement at the participating site for any or all of the documents, special arrangements must be made between the principal investigator and the Sponsor to store these in a sealed container(s) outside of the participating site so that they can be returned sealed to the principal investigator, in case of a regulatory audit. Where source documents are required for the continued care of the

Confidential

subject; appropriate copies should be made before storing outside of the participating site.

16. STUDY SPONSORSHIP AND FINANCING

Within the European Union, Institut Jules Bordet will be the legal sponsor of the Study, as that term is defined under the Belgian law of 7 May 2004 concerning experiments on the human person, implementing the Directive 2001/20/EC of the European Parliament and of the Council of 4 April 2001 on the approximation of the laws, regulations and administrative provisions of the Member States relating to the implementation of good clinical practice in the conduct of clinical trials on medicinal products for human use ("EU Clinical Trials Directive").

Institut Jules Bordet is solely responsible for any and all safety reporting associated with the conduct of the Study, as well as any and all regulatory obligations associated with the conduct of the Study, except for those obligations expressly identified in specific Agreements made with third parties

The contact details of the study chair at the Institut Jules Bordet are:

Michail Ignatiadis

Institut Jules Bordet

Rue Héger-Bordetstraat 1

1000 Bruxelles/Brussel

Belgique/België

Phone: +32 2 541 72 81

e-mail: *michail.ignatiadis@bordet.be*

This study is supported by a grant from Pfizer.

Pfizer will also provide the palbociclib free of charge to all study subjects.

17. STUDY INSURANCE

The Sponsor took an insurance policy for the study according to the laws of the countries where the study is conducted. An insurance certificate must be available to the participating sites at the time of study initiation. Clinical study insurance is only valid in participating sites authorised by the sponsor.

The insurance of the sponsor does not relieve the participating site and the principal investigator of any obligation to maintain their own liability insurance policy.

18. PUBLICATION POLICY

Publication and presentations of any results from this study shall be in accordance with accepted scientific practice, academic standards and customs and in accordance with the specific policy developed for this study if any.

The final publication of the study results will be written by the study chair or designated active principal investigators of the study on the basis of the final analysis performed at the Sponsor Headquarters (Institut Jules Bordet, Brussels, Belgium),

The Sponsor recognises right of utilising data derived from the Study for teaching purposes, communication at congresses and scientific publications.

Nevertheless, in order to ensure the accuracy and scientific value of the information, while preserving the independence and accountability of the study chair and the confidentiality of the information, only clear, checked and validated data shall be used. To that effect, it is essential that the study chair and the Sponsor exchange and discuss, prior to any publication or communication, any draft publication or communication made by the study chair.

All manuscripts will include an appropriate acknowledgment section, mentioning all principal investigators who have contributed to the study, the staff of the Clinical Trial Support Unit involved in the study, as well as the supporting bodies. The number of acknowledgments per participating entity shall depend on the journal's rules and be based on fair and practical considerations.

19. REFERENCES

1. Finn RS, Crown JP, Lang I, Boer K, Bondarenko IM, Kulyk SO, et al. The cyclin-dependent kinase 4/6 inhibitor palbociclib in combination with letrozole versus letrozole alone as first-line treatment of oestrogen receptor-positive, HER2-negative, advanced breast cancer (PALOMA-1/TRIO-18): a randomised phase 2 study. Lancet Oncol. 2015 Jan;16(1):25–35.

2. Finn et al, PALOMA-2: Primary results from a phase III trial of palbociclib (P) with letrozole (L) compared with letrozole alone in postmenopausal women with ER+/HER2– advanced breast cancer (ABC). J Clin Oncol 34, 2016 (suppl; abstr 507).

3. Turner NC, Ro J, André F, Loi S, Verma S, Iwata H, et al. Palbociclib in Hormone-Receptor–Positive Advanced Breast Cancer. N Engl J Med. 2015 Jul 16;373(3):209–19.

4. Miller AB, Hoogstraten B, Staquet M, Winkler A. Reporting results of cancer treatment. Cancer. 1981 Jan 1;47(1):207–14.

5. Symmans WF, Peintinger F, Hatzis C, Rajan R, Kuerer H, Valero V, et al. Measurement of Residual Breast Cancer Burden to Predict Survival After Neoadjuvant Chemotherapy. J Clin Oncol. 2007 Sep 4;25(28):4414–22.

6. Sotiriou C, Wirapati P, Loi S, Harris A, Fox S, Smeds J, et al. Gene expression profiling in breast cancer: understanding the molecular basis of histologic grade to improve prognosis. J Natl Cancer Inst. 2006 Feb 15;98(4):262–72.

7. Bedard PL, Singhal SK, Ignatiadis M, Bradbury I, Haibe-Kains B, Desmedt C, et al. Low residual proliferation after short-term letrozole therapy is an early predictive marker of response in high proliferative ER-positive breast cancer. Endocr Relat Cancer. 2011 Dec;18(6):721–30.

8. Result Content View [Internet]. [cited 2016 Jan 27]. Available from: http://www.abstracts2view.com/sabcs15/view.php?nu=SABCS15L_1264&terms=

9. Dobbin KK, Simon RM. Sample size planning for developing classifiers using high-dimensional DNA microarray data. Biostat Oxf Engl. 2007 Jan;8(1):101–17.

10. Torre LA, Bray F, Siegel RL, Ferlay J, Lortet-Tieulent J, Jemal A. Global cancer statistics, 2012. CA Cancer J Clin. 2015 Mar;65(2):87–108.

11. Toriola AT, Colditz GA. Trends in breast cancer incidence and mortality in the United States: implications for prevention. Breast Cancer Res Treat. 2013 Apr;138(3):665–73.

12. Bleyer A, Welch HG. Effect of three decades of screening mammography on breast-cancer incidence. N Engl J Med. 2012 Nov 22;367(21):1998–2005.

13. DeSantis CE, Bray F, Ferlay J, Lortet-Tieulent J, Anderson BO, Jemal A. International Variation in Female Breast Cancer Incidence and Mortality Rates. Cancer Epidemiol Biomark Prev Publ Am Assoc Cancer Res Cosponsored Am Soc Prev Oncol. 2015 Oct;24(10):1495–506.

14. Anderson WF, Katki HA, Rosenberg PS. Incidence of breast cancer in the United States: current and future trends. J Natl Cancer Inst. 2011 Sep 21;103(18):1397–402.

15. Mariotto AB, Robin Yabroff K, Shao Y, Feuer EJ, Brown ML. Projections of the Cost of Cancer Care in the United States: 2010-2020. JNCI J Natl Cancer Inst. 2011 Jan 19;103(2):117–28.

16. Evolution of cancer treatments: Hormone therapy | American Cancer Society [Internet]. [cited 2016 Apr 27]. Available from: http://www.cancer.org/cancer/cancerbasics/thehistoryofcancer/the-history-of-cancer-cancer-treatment-hormone-therapy

17. Sini V, Cinieri S, Conte P, De Laurentiis M, Leo AD, Tondini C, et al. Endocrine therapy in post-menopausal women with metastatic breast cancer: From literature and guidelines to clinical practice. Crit Rev Oncol Hematol. 2016 Apr;100:57–68.

18. van Dam PA, van Dam VCN, Altintas S, Papadimitriou K, Rolfo C, Trinh XB. Neoadjuvant endocrine treatment in early breast cancer: An overlooked alternative? Eur J Surg Oncol EJSO. 2016 Mar;42(3):333–42.

19. Burstein HJ, Temin S, Anderson H, Buchholz TA, Davidson NE, Gelmon KE, et al. Adjuvant endocrine therapy for women with hormone receptor-positive breast cancer: american society of clinical oncology clinical practice guideline focused update. J Clin Oncol Off J Am Soc Clin Oncol. 2014 Jul 20;32(21):2255–69.

20. Clemons M, Danson S, Howell A. Tamoxifen (‘Nolvadex’): a review. Cancer Treat Rev. 2002 Aug;28(4):165–80.

21. Rugo HS, Rumble RB, Macrae E, Barton DL, Connolly HK, Dickler MN, et al. Endocrine Therapy for Hormone Receptor-Positive Metastatic Breast Cancer: American Society of Clinical Oncology Guideline. J Clin Oncol [Internet]. 2016 May 23 [cited 2016 Jun 26]; Available from: http://jco.ascopubs.org/cgi/doi/10.1200/JCO.2016.67.1487

22. Francis PA, Regan MM, Fleming GF, Láng I, Ciruelos E, Bellet M, et al. Adjuvant Ovarian Suppression in Premenopausal Breast Cancer. N Engl J Med. 2015 Jan 29;372(5):436–46.

23. Burstein HJ, Lacchetti C, Anderson H, Buchholz TA, Davidson NE, Gelmon KE, et al. Adjuvant Endocrine Therapy for Women With Hormone Receptor-Positive Breast Cancer: American Society of Clinical Oncology Clinical Practice Guideline Update on Ovarian Suppression. J Clin Oncol. 2016 May 10;34(14):1689–701.

24. Early Breast Cancer Trialists' Collaborative Group (EBCTCG), Dowsett M, Forbes JF, Bradley R, Ingle J, Aihara T, et al. Aromatase inhibitors versus tamoxifen in early breast cancer: patient-level meta-analysis of the randomised trials. Lancet Lond Engl. 2015 Oct 3;386(10001):1341–52.

25. Barroso-Sousa R, Fonseca Reis Silva DD, Machado Alessi JV, Mano MS. Neoadjuvant endocrine therapy in breast cancer: current role and future perspectives. ecancermedicalscience [Internet]. 2016 Jan 7 [cited 2016 Apr 27];10. Available from: http://www.ecancer.org/journal/10/full/609-neoadjuvant-endocrine-therapy-in-breast-cancer-current-role-and-future-perspectives.php

26. Brueggemeier RW, Hackett JC, Diaz-Cruz ES. Aromatase inhibitors in the treatment of breast cancer. Endocr Rev. 2005 May;26(3):331–45.

27. Snoj N, Bedard PL, de Azambuja E, Cardoso F, Piccart M. Are we HER-ting for innovation in neoadjuvant breast cancer trial design? Breast Cancer Res BCR. 2009;11(1):201.

28. Zardavas D, Piccart M. Neoadjuvant therapy for breast cancer. Annu Rev Med. 2015;66:31–48.

29. Ellis MJ, Tao Y, Luo J, A'Hern R, Evans DB, Bhatnagar AS, et al. Outcome Prediction for Estrogen Receptor-Positive Breast Cancer Based on Postneoadjuvant Endocrine Therapy Tumor Characteristics. JNCI J Natl Cancer Inst. 2008 Oct 1;100(19):1380–8.

30. Dowsett M, Smith IE, Ebbs SR, Dixon JM, Skene A, Griffith C, et al. Short-term changes in Ki-67 during neoadjuvant treatment of primary breast cancer with anastrozole or tamoxifen alone or combined correlate with recurrence-free survival. Clin Cancer Res Off J Am Assoc Cancer Res. 2005 Jan 15;11(2 Pt 2):951s-8s.

31. Shimizu D, Ishikawa T, Tanabe M, Sasaki T, Ichikawa Y, Chishima T, et al. Preoperative endocrine therapy with goserelin acetate and tamoxifen in hormone receptor-positive premenopausal breast cancer patients. Breast Cancer Tokyo Jpn. 2014 Sep;21(5):557–62.

32. Masuda N, Sagara Y, Kinoshita T, Iwata H, Nakamura S, Yanagita Y, et al. Neoadjuvant anastrozole versus tamoxifen in patients receiving goserelin for premenopausal breast cancer (STAGE): a double-blind, randomised phase 3 trial. Lancet Oncol. 2012 Apr;13(4):345–52.

33. Smith IE, Dowsett M, Ebbs SR, Dixon JM, Skene A, Blohmer J-U, et al. Neoadjuvant treatment of postmenopausal breast cancer with anastrozole, tamoxifen, or both in combination: the Immediate Preoperative Anastrozole, Tamoxifen, or Combined with Tamoxifen (IMPACT) multicenter double-blind randomized trial. J Clin Oncol Off J Am Soc Clin Oncol. 2005 Aug 1;23(22):5108–16.

34. Eiermann W, Paepke S, Appfelstaedt J, Llombart-Cussac A, Eremin J, Vinholes J,

et al. Preoperative treatment of postmenopausal breast cancer patients with letrozole: A randomized double-blind multicenter study. Ann Oncol Off J Eur Soc Med Oncol ESMO. 2001 Nov;12(11):1527–32.

35. Cataliotti L, Buzdar AU, Noguchi S, Bines J, Takatsuka Y, Petrakova K, et al. Comparison of anastrozole versus tamoxifen as preoperative therapy in postmenopausal women with hormone receptor-positive breast cancer: the Pre-Operative "Arimidex" Compared to Tamoxifen (PROACT) trial. Cancer. 2006 May 15;106(10):2095–103.

36. Seo JH, Kim YH, Kim JS. Meta-analysis of pre-operative aromatase inhibitor versus tamoxifen in postmenopausal woman with hormone receptor-positive breast cancer. Cancer Chemother Pharmacol. 2009 Jan;63(2):261–6.

37. Piccart-Gebhart MJ, Procter M, Leyland-Jones B, Goldhirsch A, Untch M, Smith I, et al. Trastuzumab after adjuvant chemotherapy in HER2-positive breast cancer. N Engl J Med. 2005 Oct 20;353(16):1659–72.

38. Piccart-Gebhart M, Holmes E, Baselga J, de Azambuja E, Dueck AC, Viale G, et al. Adjuvant Lapatinib and Trastuzumab for Early Human Epidermal Growth Factor Receptor 2-Positive Breast Cancer: Results From the Randomized Phase III Adjuvant Lapatinib and/or Trastuzumab Treatment Optimization Trial. J Clin Oncol [Internet]. 2015 Nov 23 [cited 2015 Dec 29]; Available from: http://jco.ascopubs.org/cgi/doi/10.1200/JCO.2015.62.1797

39. Swain SM, Baselga J, Kim S-B, Ro J, Semiglazov V, Campone M, et al. Pertuzumab, trastuzumab, and docetaxel in HER2-positive metastatic breast cancer. N Engl J Med. 2015 Feb 19;372(8):724–34.

40. Durkee BY, Qian Y, Pollom EL, King MT, Dudley SA, Shaffer JL, et al. Cost-Effectiveness of Pertuzumab in Human Epidermal Growth Factor Receptor 2-Positive Metastatic Breast Cancer. J Clin Oncol Off J Am Soc Clin Oncol. 2015 Sep 8;

41. Fleeman N, Bagust A, Boland A, Dickson R, Dundar Y, Moonan M, et al. Lapatinib and trastuzumab in combination with an aromatase inhibitor for the first-line treatment of metastatic hormone receptor-positive breast cancer which over-expresses human epidermal growth factor 2 (HER2): a systematic review and economic analysis. Health Technol Assess Winch Engl. 2011;15(42):1–93, iii–iv.

42. Malumbres M, Barbacid M. Cell cycle, CDKs and cancer: a changing paradigm. Nat Rev Cancer. 2009 Mar;9(3):153–66.

43. Arnold A. Cyclin D1 in Breast Cancer Pathogenesis. J Clin Oncol. 2005 Jun 20;23(18):4215–24.

44. Keyomarsi K, Tucker SL, Buchholz TA, Callister M, Ding Y, Hortobagyi GN, et al. Cyclin E and survival in patients with breast cancer. N Engl J Med. 2002 Nov 14;347(20):1566–75.

45. Caldon CE, Sergio CM, Kang J, Muthukaruppan A, Boersma MN, Stone A, et al. Cyclin E2 overexpression is associated with endocrine resistance but not insensitivity to CDK2 inhibition in human breast cancer cells. Mol Cancer Ther. 2012 Jul;11(7):1488–99.

46. Ades F, Zardavas D, Bozovic-Spasojevic I, Pugliano L, Fumagalli D, de Azambuja E, et al. Luminal B Breast Cancer: Molecular Characterization, Clinical Management, and Future Perspectives. J Clin Oncol. 2014 Sep 1;32(25):2794–803.

47. Span PN, Tjan-Heijnen VCG, Manders P, Beex LVAM, Sweep CGJ. Cyclin-E is a strong predictor of endocrine therapy failure in human breast cancer. Oncogene. 2003 Jul 31;22(31):4898–904.

48. Stendahl M, Kronblad A, Rydén L, Emdin S, Bengtsson NO, Landberg G. Cyclin D1 overexpression is a negative predictive factor for tamoxifen response in postmenopausal breast cancer patients. Br J Cancer. 2004 May 17;90(10):1942–8.

49. Michaud K, Solomon DA, Oermann E, Kim J-S, Zhong W-Z, Prados MD, et al. Pharmacologic inhibition of cyclin-dependent kinases 4 and 6 arrests the growth of glioblastoma multiforme intracranial xenografts. Cancer Res. 2010 Apr 15;70(8):3228–38.

50. Sherr CJ, Beach D, Shapiro GI. Targeting CDK4 and CDK6: From Discovery to Therapy. Cancer Discov [Internet]. 2015 Dec 11 [cited 2016 Feb 12]; Available from: http://cancerdiscovery.aacrjournals.org/cgi/doi/10.1158/2159-8290.CD-15-0894

51. Schwartz GK, LoRusso PM, Dickson MA, Randolph SS, Shaik MN, Wilner KD, et al. Phase I study of PD 0332991, a cyclin-dependent kinase inhibitor, administered in 3-week cycles (Schedule 2/1). Br J Cancer. 2011 Jun 7;104(12):1862–8.

52. Flaherty KT, Lorusso PM, Demichele A, Abramson VG, Courtney R, Randolph SS, et al. Phase I, dose-escalation trial of the oral cyclin-dependent kinase 4/6 inhibitor PD 0332991, administered using a 21-day schedule in patients with advanced cancer. Clin Cancer Res Off J Am Assoc Cancer Res. 2012 Jan 15;18(2):568–76.

53. Dickson MA, Tap WD, Keohan ML, D'Angelo SP, Gounder MM, Antonescu CR, et al. Phase II trial of the CDK4 inhibitor PD0332991 in patients with advanced CDK4-amplified well-differentiated or dedifferentiated liposarcoma. J Clin Oncol Off J Am Soc Clin Oncol. 2013 Jun 1;31(16):2024–8.

54. Hu W, Sung T, Jessen B, Thibault S, Finkelstein MB, Khan NK, et al. Mechanistic investigation of bone marrow suppression associated with palbociclib and its differentiation from cytotoxic chemotherapies. Clin Cancer Res Off J Am Assoc Cancer Res. 2015 Dec 2;

55. Ellis MJ, Suman VJ, Hoog J, Lin L, Snider J, Prat A, et al. Randomized Phase II Neoadjuvant Comparison Between Letrozole, Anastrozole, and Exemestane for Postmenopausal Women With Estrogen Receptor-Rich Stage 2 to 3 Breast Cancer: Clinical and Biomarker Outcomes and Predictive Value of the Baseline PAM50-Based Intrinsic Subtype--ACOSOG Z1031. J Clin Oncol. 2011 Jun 10;29(17):2342–9.

56. Ma, CX S6-05 A PHASE II TRIAL OF NEOADJUVANT PALBOCICLIB, A CYCLIN-DEPENDENT KINASE (CDK) 4/6 INHIBITOR, IN COMBINATION WITH ANASTROZOLE FOR CLINICAL STAGE 2 OR 3 ESTROGEN RECEPTOR

POSITIVE HER2 NEGATIVE (ER+HER2-) BREAST CANCER (BC). SABCS 2015.

57. Raunio H, Rautio A, Gullstén H, Pelkonen O. Polymorphisms of CYP2A6 and its practical consequences. Br J Clin Pharmacol. 2001 Oct;52(4):357–63.

58. Dowsett M, Nielsen TO, A'Hern R, Bartlett J, Coombes RC, Cuzick J, et al. Assessment of Ki67 in breast cancer: recommendations from the International Ki67 in Breast Cancer working group. J Natl Cancer Inst. 2011 Nov 16;103(22):1656–64.

EudraCT Number: 2012-004860-22

Sponsor Protocol Number: IJB-BCTL:20120306

The PEARL Study

Pet imaging as a biomarker of **E**verolimus **A**dded value in hormone **R**efractory postmenopausa**L** women

Study Coordinators: Andrea Gombos, Géraldine Gebhart

Andrea Gombos	Géraldine Gebhart
Institute Jules Bordet<br>125 Boulevard de Waterloo<br>Brussels, BELGIUM 1000<br>Phone: +32 2 541 72 32<br>E-mail: andrea.gombos@bordet.be	Institute Jules Bordet<br>125 Boulevard de Waterloo<br>Brussels, BELGIUM 1000<br>Phone: +32 2 541 3240<br>E-mail: geraldine gebhart@bordet.be

Protocol design: Martine Piccart, Stefan Michiels, Patrick Flamen

Study Statistician: Marianne Paesmans

Sponsor:

Institut Jules Bordet

Rue Héger-Bordetstraat 1, 1000 Bruxelles/Brussel

Belgique/België

Protocol version 3

Issue date: 28/Apr/2017

CONFIDENTIAL

Information and data included in this protocol contain trade secrets and privileged or confidential information which is the property of the Institut Jules Bordet. No person is authorised to make it public without the written permission of the director, Institut Jules Bordet. These restrictions on disclosure will apply equally to all future information supplied to you, which is indicated as privileged or confidential. This material may be disclosed to and used by your staff and associates as may be necessary to conduct the clinical study.

PROTOCOL WRITING COMMITTEE

Institut Jules Bordet	Lina Pugliano<br>Felipe Ades Moraes<br>Andrea Gombos<br>Patrick Flamen<br>Camilo Garcia<br>Ioannis Karfis<br>Evandro de Azambuja<br>Michail Ignatiadis<br>Geraldine Gebhart<br>Martine Piccart<br>Magalie Krieguer<br>Stefan Michiels<br>Ahmad Awada<br>Marianne Paesmans<br>Laura Belenguer Querol<br>Thomas Guiot<br>Lieveke Ameye<br>Aline Kayumba<br>Chloé Velghe
Novartis	Karen Detournay<br>Aurelia Lefever

PROTOCOL APPROVAL – Signature Page

EudraCT Number: 2012-004860-22

Sponsor Protocol Number: IJB-BCTL:20120306

ClinicalTrials.gov Number: NCT02028364

The signature below constitutes the approval of this protocol and the attachments, and provides the necessary assurances that this trial will be conducted according to all stipulations of the protocol, including all statements regarding confidentiality, and according to local legal and regulatory requirements and applicable European regulations and ICH guidelines.

Study Coordinators:

Title and Name	Approval date	Signature
Andrea Gombos, MD		

Title and Name	Approval date	Signature
Géraldine Gebhart, MD, PhD		

Study Statistician

Title and Name	Approval date	Signature
Marianne Paesmans, MSc		

Site Principal Investigator

Title and Name	Function	Approval date	Signature
	Site Principal Investigator		

DOCUMENT HISTORY

Version	Protocol version release date	Short description of modifications	Submission as substantial amendment
1	22-Oct-2013		☒ Yes<br>☐ No
2	14-Apr-2014	- Clarification of haematology baseline assessments<br>- Clarification of PET timelines<br>- Change of efficacy assessment time point form every 8 weeks to 12 weeks to be in accordance with the reimbursement conditions for Afinitor in combination with exemestane.	☒ Yes<br>☐ No
3	28-Apr-2017	- Add Pilot phase results (Second FDG PET/CT timepoint selected for the main phase)<br>- Main phase sample size's recalculation<br>- Modification of schedule of assessment<br>- Modification of samples collection and translational researches<br>- Clarification inclusion/exclusion criteria	☒ Yes<br>☐ No

TABLE OF CONTENTS

PROTOCOL APPROVAL – Signature Page 3
DOCUMENT HISTORY 4
TABLE OF CONTENTS 5
SYNOPSIS 9
LIST OF ABBREVIATIONS 13
1. BACKGROUND AND SCIENTIFIC RATIONALE 16
1.1. Epidemiology of breast cancer 16
1.2. Treatment options for endocrine responsive breast cancer 16
1.3. Bidirectional cross talk of ER 17
1.4. Overview of Everolimus 18
1.4.1. Mechanism of action 18
1.4.2. Pharmacokinetics of Everolimus 18
1.5. Overview of Exemestane 19
1.6. Clinical trial experience: Everolimus 19
1.7. The role of FDG-PET in BC 20
1.8. Study rationale 21
2. STUDY OBJECTIVES 21
2.1. Primary Objective 21
2.2. Secondary Objective 21
3. STUDY DESIGN AND ENDPOINTS 21
3.1. Overview of Study Design 21
3.2. Screening Phase 22
3.3. Treatment 22
3.3.1. Pilot Phase 22
3.3.1.1. Endpoints for pilot phase 23
3.3.2. Main Study 23
3.3.2.1. Endpoints for main study phase 24
3.3.3. Definitions 24
3.3.3.1. Definition of metabolic target lesions on FDG PET/CT 25
3.3.3.2. Definition of FDG-PET/CT response 25
3.3.3.3. ORILaB – Imaging CoreLAB 25
3.3.3.4. Imaging follow-up 26
3.4. Follow-up 26
3.5. Survival Data Collection 26
3.6. End of Study 26
3.7. Number of patients 26
3.8. Centres 26

4. SELECTION AND WITHDRAWAL OF SUBJECTS 27
4.1. Overview 27
4.2. Inclusion Criteria 27
4.3. Exclusion Criteria 28
4.4. Withdrawal of subjects 29
5. SCHEDULE OF ASSESSMENTS AND PROCEDURES 30
5.1. Screening and baseline 30
5.2. Treatment 30
5.2.1. Pilot phase 30
5.2.1. Main phase 31
5.3. End of treatment visit 31
5.4. Survival Assessment 31
5.5. Biomarker Assessment 31
5.5.1. Background 31
5.5.2. Primary tumor / Blood-based translational research 32
5.5.2.1. Aims 32
5.5.2.2. Samples collection: 32
5.5.2.3. Methods 33
5.5.3. Tissue samples 33
5.5.3.1. Methods 33
5.5.3.2. Statistical analysis 33
5.5.4. Not yet specified translational research 33
5.6. Table of schedule of assessments 34
6. INVESTIGATIONAL PRODUCT OR STUDY MEDICATION 40
6.1. Investigational drugs 40
6.2. Study drug preparation, supply and dispensation 40
6.2.1. Treatment duration 40
6.3. Known undesirable effects of study treatment 40
6.3.1. Everolimus 40
6.3.2. Exemestane 41
6.4. Management of Everolimus specific toxicities 41
6.4.1. Management of stomatitis/oral mucositis/mouth ulcers 41
6.4.2. Management of dermatological toxicities 42
6.4.3. Management of hyperlipidemia and hyperglycaemia 42
6.4.4. Management of Diarrhoea 43
6.4.5. Management of non-infectious pneumonitis 43
6.4.6. Management of infections 44
6.4.7. Management of haematological toxicities 45
6.4.8. Management of metabolic events 45

6.5. Follow-up of toxicities ... 45
6.6. Permitted study drug adjustments ... 45
6.7. Dosing modifications ... 46
6.8. Concomitant Medication and Treatment ... 47
6.9. Allowed therapies ... 47
6.10. Excluded therapies ... 48
7. ASSESSMENT OF EFFICACY ... 49
8. ASSESSMENT OF SAFETY ... 49
8.1. Safety parameters ... 49
8.1.1. Adverse Event ... 49
8.1.1.1. Definition ... 49
8.1.1.2. AE Intensity ... 50
8.1.1.3. Relationship to study medication ... 50
8.1.2. Serious Adverse Event (SAE) ... 51
8.1.3. Disease Progression ... 51
8.2. Handling of Safety Parameters ... 52
8.2.1. Reporting of Adverse Events ... 52
8.2.2. Reporting of Serious Adverse Events ... 52
8.2.3. Reporting of deaths ... 53
9. STATISTICAL CONSIDERATIONS AND ANALYTICAL PLAN ... 53
9.1. Statistical Model ... 53
9.1.1. Analysis of Primary Variable ... 53
9.2. Sample Size ... 54
10. DIRECT ACCESS TO SOURCE DATA/DOCUMENTS ... 54
10.1. Confidentiality of study documents and patient records ... 55
11. QUALITY CONTROL ... 55
11.1. Quality control: study monitoring ... 55
11.2. Quality control: on-site monitoring ... 55
11.3. Quality control ... 55
11.4. Regulatory agencies Inspections ... 55
12. REGULATORY AND ETHICAL CONSIDERATIONS ... 56
12.1. Regulatory considerations ... 56
12.2. Informed Consent Process ... 56
12.2.1. General ... 56
12.2.2. Process of Informed Consent ... 57
13. DATA HANDLING AND RECORD KEEPING ... 57
13.1. Investigator’s file ... 57
13.2. Case report form (CRF) ... 58
13.3. Retention of documents ... 58

Confidential

14. PUBLICATION POLICY 58
15. FINANCING AND INSURANCE 59
16. SCIENTIFIC REFERENCES 59
17. APPENDICES 65
Appendix 1: Screening, Monitoring and management for HBV 65
Screening HBV 65
Serologic Markers to be evaluated for screening of HBV 65
Monitoring and Prophylactic treatment of Hepatitis B reactivation 66
Management of HBV reactivation 66
Appendix 2: Screening, Monitoring and management for HCV 67
Screening for HBC 67
Serologic Markers to be evaluated for screening of HCV 67
Monitoring for HCV 67

SYNOPSIS

**STUDY TITLE**	**P**ET imaging as a biomarker of **E**verolimus **A**dded value in hormone **R**efractory postmenopausa**L** women.
**SHORT TITLE**	The PEARL Study
**SPONSOR**	Institut Jules Bordet (IJB) - Brussels, Belgium
**INDICATION(S)**	Locally advanced or metastatic hormone receptor positive and HER2 negative breast cancer refractory to non-steroidal aromatase inhibitors (NSAI) (i.e. anastrozole or letrozole) and treated with everolimus and exemestane. NSAI "Resistance" defined as:<br>a. Recurrence while on, or within 12 months of end of adjuvant treatment with anastrozole or letrozole; OR<br>b. Progression while on, or within one month of end of anastrozole or letrozole or treatment for locally advanced or metastatic breast cancer
**TARGET STUDY POPULATION**	Locally advanced or metastatic endocrine receptor positive and HER2 negative breast cancer refractory to non-steroidal aromatase inhibitors (NSAI) (i.e. anastrozole or letrozole)
**PHASE**	Eligible and consenting patients will be enrolled into the study and receive the study treatment: Everolimus 10mg and exemestane 25mg daily orally until disease progression or treatment withdrawal for any other reasons.<br>FDG-PET/CT scan will be performed at baseline and at the end of week 2 (day 14) and at the end of week 4 (day 28) in the pilot phase and at baseline and either at the end of week 2 (day 14) or at the end of week 4 (day 28) in the main study, following results from the pilot phase.<br>After the completion of the pilot phase and the review of the results, it was decided to perform a FDG-PET/CT at the end of week 2 (day 14) in the main study (reasons are explained in the protocol in section regarding the study design).<br>At time of FDG injection glycemia will be measured as everolimus can impact on insulinemia/glycemia and therefore indirectly on the SUV of the FDG uptake.
**STUDY DESIGN**	Single arm, multi-centre, open label
**OBJECTIVES**	• Primary objectives : To evaluate if early metabolic response (MR) using FDG-PET/CT is associated with progression free survival (PFS) in ER+, HER2 negative ABC or MBC patients treated with exemestane plus everolimus.<br>• Secondary objectives : To evaluate if early metabolic response (MR) using FDG-PET/CT is associated with overall survival (OS) in ER+, HER2 negative locally advanced or MBC patients treated with exemestane plus everolimus.
**ENDPOINTS**	<u>For the pilot phase:</u><br>• Primary endpoint: Proportion of FDG-PET/CT metabolic non responders/FDG-PET/CT metabolic responders<br>• Secondary endpoint: estimate the most suitable second FDG-

	PET/CT time point (2 weeks versus 4 weeks after initiation of treatment).<br><br><u>For the main phase:</u><br>• Primary endpoints : Progression free survival (PFS) (based on RECIST 1.1 criteria)<br>• Secondary endpoints : Overall survival (OS)
**NUMBER OF SUBJECTS**	<u>For the pilot phase:</u><br>30 patients were initially planned for this phase. Out of 44 patients screened 27 patients were finally considered as evaluable (Accrual was stopped after 30 evaluable patients included but 3 patients were denoted inevaluable afterwards).<br><br><u>For the main phase:</u><br>Pilot phase's results showed that the metabolic non-response rate on Day 14 and Day 28 was similar: 59% (16/27) (Kappa coefficient for concordance: 0.85 (95% CI, 0.64 to 1); mean SUV decrease at day 14: 26%, at day 28: 32%-p-0.03). According to this metabolic non-response rate higher than previously assumed (i.e. 59% instead of 20%), 42 PFS event are needed. At an accrual rate of 1-2 evaluable patients per month, 46 evaluable patients in total are needed to answer the primary endpoint of the trial. This means 19 (=46-27(pilot)) more evaluable patients have to be recruited during the main phase. If we consider the screen failure rate observed in the pilot phase, about 30 patients need to be screened to complete the study.
**INCLUSION CRITERIA**	Patients must meet ALL of the following criteria to be eligible for this study:<br>1. Adult women (≥18 years of age) with locally advanced or metastatic breast cancer not amenable to curative treatment by surgery or radiotherapy.<br>2. Histological or cytological confirmation of estrogen-receptor positive (ER+), HER2 negative breast cancer.<br>3. Postmenopausal female defined as:<br>a. Age ≥ 55 years and one year or more of amenorrhoea<br>b. Age <55 years and one year or more of amenorrhoea, with an estradiol assay <20pg/ml<br>c. Surgical menopause with bilateral oophorectomy<br>4. Breast cancer that is refractory to non-steroidal aromatase inhibitors (NSAI) (i.e. anastrozole or letrozole) defined as:<br>a. Recurrence while on, or within 12 months of end of adjuvant treatment with anastrozole or letrozole; OR<br>b. Progression while on, or within one month of end of anastrozole or letrozole or treatment for locally advanced or metastatic breast cancer.<br>5. FDG-PET/CT measurable disease defined as: at least one target lesion fulfilling following criteria:<br>a. Size ≥1.5cm; AND<br>b. FDG-PET/CT avid lesion with uptake above the background liver uptake as described below:<br>i.e. with a marked accumulation of FDG, at least 1.5-fold greater than liver SUV mean + 2 SDs (in 3cm spherical ROI in normal right lobe of liver). If liver is abnormal, target lesion should have uptake > 2.0 x SUV mean of blood pool in 1cm diameter ROI in descending thoracic aorta.<br>6. The patient must have at least 1 FDG-PET/CT measurable lesion also measurable according to RECIST 1.1 criteria.<br>7. Radiological or clinical evidence of recurrence or progression

	on last systemic therapy prior to enrolment.<br>8. Adequate bone marrow function as shown by:<br>a. Haemoglobin (HgB) ≥ 9.0 g/dL<br>b. ANC ≥ 1,500/mm3 (≥1.5 x 109/L)<br>c. Platelets ≥ 100,000/mm3 (≥ 100x 109/L)<br>9. Adequate liver function as shown by:<br>a. Alanine aminotransferase (ALT) and aspartate aminotransferase (AST) ≤2.5xULN (or ≤ 5 if hepatic metastases are present)<br>b. Total serum bilirubin ≤ 1.5 x ULN (≤ 3 ULN for patients known to have Gilbert Syndrome)<br>10. Adequate renal function as shown by Serum creatinine≤ 1.5 x ULN<br>11. Fasting serum cholesterol, triglycerides and glucose<br>a. Fasting serum cholesterol ≤ 300 mg/dL or 7.75 mmol/L<br>b. Fasting triglycerides ≤ 2.5 x ULN<br>c. Fasting glucose < 1.5 x ULN<br>12. Eastern Co-operative Oncology Group (ECOG) performance status ≤ 2.<br>13. Written and signed informed consent obtained before any trial related activity.<br>14. Availability of a FFPE core of primary breast tumor<br>15. Possibility to obtain the mandatory blood samples for the translational research studies.<br>16. For patients with accessible metastatic lesions, possibility to obtain the mandatory biopsy (FFPE and frozen) of a metastatic lesion
**EXCLUSION CRITERIA**	Patients meeting any **ONE** of the following criteria are **NOT** eligible for the study:<br>1. HER2-overexpressing patients by local laboratory testing (IHC 3+ staining or in situ hybridization positive).<br>2. Patients with only non-measurable lesions by FDG-PET/CT (e.g. pleural effusion, ascites etc.).<br>3. Symptomatic visceral disease for example liver, pulmonary metastases or lymphangitis carcinomatosis.<br>4. Known hypersensitivity to mTOR inhibitors, e.g. sirolimus (rapamycin).<br>5. Another malignancy within 5 years prior to enrolment, with the exception of adequately treated in-situ carcinoma of the cervix, uteri, basal or squamous cell carcinoma or non-melanomatous skin cancer.<br>6. Radiotherapy within four weeks prior to enrolment except in case of localized radiotherapy for analgesic purpose or for lytic lesions at risk of fracture, which can then be completed within two weeks prior to enrolment. Patients must have recovered from radiotherapy toxicities prior to enrolment.<br>7. Currently receiving hormone replacement therapy, unless discontinued prior to enrolment.<br>8. Symptomatic brain metastases or other central nervous system metastases which are not controlled by local treatments.<br>9. Patients receiving concomitant immunosuppressive agents or chronic corticosteroid use at the time of study entry except in cases outlined below:<br>a. Topical applications (e.g. rash)<br>b. Inhaled sprays (e.g. obstructive airways disease)<br>c. Eye drops<br>d. Local injections (e.g. intra-articular)

	e. Stable low dose of corticosteroids for at least two weeks before enrolment<br>10. Patients with known HIV seropositivity. Screening for HIV infection at baseline is not required<br>11. Acute and chronic, active infectious disorders (except for Hepatitis B and Hepatitis C positive patients).<br>12. Active bleeding diathesis, or on oral anti-vitamin K medication (except low dose warfarin, LMWH and acetylsalicylic acid or equivalent, as long as the INR is ≤ 2.0).<br>13. Any severe uncontrolled medical conditions such as:<br>a. Unstable angina pectoris, symptomatic congestive heart failure, myocardial infarction ≤ 6 months prior to enrolment, uncontrolled cardiac arrhythmia<br>b. Uncontrolled diabetes as defined by fasting glucose > 1.5 x ULN<br>c. Acute and chronic, active infectious disorders and non-malignant medical illnesses that are uncontrolled or whose control may be jeopardized by the complications of this study therapy.<br>d. Symptomatic deterioration of lung function<br>14. Patients being treated with drugs recognized as being strong inhibitors or inducers of the isoenzyme CYP3A (Rifabutin, Rifampicin, Clarithromycin, Ketoconazole, Itraconazoleonazole, Voriconazole, Ritonavir, Telithromycin) within the last 5 days prior to enrolment (see list of clinically relevant drug interaction listed in section 6.10).<br>15. History of non-compliance to medical regimens.<br>16. Patients unwilling or unable to comply with the protocol.<br>17. Concurrent anti-cancer treatment in another investigational trial, including hormonal therapy, immunotherapy or targeted agents other than those administered in this study.
**INVESTIGATIONAL MEDICINAL PRODUCT(S) DOSE/ROUTE/REGIMEN**	Everolimus 10 mg orally daily given in conjunction with exemestane 25 mg orally daily until disease progression or treatment discontinuation for any other reasons.
**ASSESSMENT OF EFFICACY**	Primary Endpoint: Progression free survival based on RECIST criteria 1.1. The metabolic response will be correlated to PFS based on the RECIST criteria 1.1
**ASSESSMENT OF SAFETY**	Clinical and laboratory events will be reported according to the National Cancer Institute-Common Terminology Criteria for Adverse Events (NCI-CTCAE) version 4.0.
**PROCEDURES SUMMARY**	Eligible and consenting patients will be enrolled into the study and receive the study treatment: Everolimus 10mg and exemestane 25mg daily orally until disease progression or treatment discontinuation for any other reasons.<br>FDG-PET/CT scan will be performed at baseline and at the end of week 2 (day 14) and at the end of week 4 (day 28) in the pilot phase.<br>For the main phase, following results from the pilot phase, FDG-PET/CT scan will be performed at baseline and at the end of week 2 (day 14) Day 14 FDG-PET/CT was chosen for the second phase, given the high concordance between the two time points used in the pilot phase and the aim to reduce at maximum treatment exposure and costs.<br>At time of FDG injection glycemia will be measured as everolimus can impact on insulinemia/glycemia and therefore indirectly on the

	SUV of the FDG uptake.
**STATISTICAL ANALYSES**	We assume that the FDG-PET/CT responders have a similar PFS to the patients treated with exemestane plus everolimus in the BOLERO-2 trial (10.6 months) and that non FDG-PET/CT responders will have a similar PFS as patients treated with exemestane alone in the BOLERO-2 trial (4.1 months). Under exponential survival this translated to a hazard ratio of 0.36. According to the pilot phase (N= 27 evaluable patients) non-FDG-PET/CT responders represent 59% (instead of the assumed 20% at the initial design) of the population and that their 6 months PFS is given by 37% (estimated from BOLERO-2 survival curves, control group).<br><br>In order to show an increase in 6-months PFS from 37% for non-FDG-PET/CT responders to 70% for FDG-PET/CT responders (corresponding to a hazard ratio of 0.36) with 90% power at a two-sided significance level 0.05 using a logrank test, and with the pilot phase's results, a total of 42 PFS events are required in the analysis (instead of the 62 PFS events calculated in the initial design).<br><br>At an accrual rate of 1-2 evaluable patients per month, 46 evaluable patients in total are needed. This means 19 (=46-27(pilot)) more evaluable patients are needed to answer the primary endpoint of the trial. If we consider the screen failure rate observed in the pilot phase, about 30 patients need to be screened to complete the study.<br><br>A Cox regression model will be applied to assess the effect of FDG-PET/CT metabolic response rate on PFS and OS. Multivariate analyses will be performed, adjusting for maximum three factors.
**TRANSLATIONAL RESEARCH(ES)**	The translational research project aims to identify biomarkers predicting resistance to exemestane and everolimus therapy, by studying primary tumor and metastatic tumor tissue when available using techniques including but not restrictive to DNA and RNA sequencing. We will also collect plasma for circulating tumor DNA, proteomic and metabolomic analysis and for further translational research purposes.<br>• Mandatory primary breast tumor will be collected at screening<br>• Mandatory blood samples will be collected several times during the study and at progression.<br>• The biopsy of metastatic lesions will be performed if accessible lesions are identified at baseline. Biopsies after the second FDG-PET/CT (D14) and at progression are optional and recommended in the case of easily accessible lesions, like subcutaneous lesions.
**LENGTH OF THE STUDY**	Pilot phase: Accrual was anticipated to proceed for 18 to 24 months, but took 29 months.<br><br>Main phase: Accrual is anticipated to proceed for 12 months.
**END OF STUDY**	This is an event driven analysis. The analysis for the primary endpoint will be performed after 42 PFS events have occurred. The study will end when all OS events have been reported and the database has been fully cleaned and frozen for all analyses.

LIST OF ABBREVIATIONS

Abbreviation	Description

ABC	Advanced breast cancer
ADL	Activities of Daily Living
AE	Adverse Event
AI	Aromatase inhibitor
Akt	Also known as Protein Kinase B
ANC	Absolute Neutrophil Count
AR	Adverse Reaction
AUC	Area under the curve
BAL	Bronchoalveolar lavage
BC	Breast Cancer
BIG	Breast International Group
CL	Drug clearance
C_{max}	Peak concentration
CTC	Circulating Tumour Cell
CYP3A4	Cytochrome P450 isoenzyme 3A4
DFS	Disease Free Survival
DLCO	Diffusing Capacity of the Lung for Carbon Monoxide
(e)CRF	(Electronic) Case Report Form
ECOG	Eastern Cooperative Oncology Group
EGFR-1	Epidermal Growth Factor Receptor 1
EOT	End Of Treatment
ER	Endocrine Estrogen Receptor
ER+	Endocrine Responsive tumors
EVCTM	EudraVigilance Clinical Trial Module
FDG-PET	Fluorodeoxyglucose-positron emission tomography
F	Oral availability, fraction of the dose of drug given orally that reaches the systemic circulation
FFPE	Formalin Fixed Paraffin embedded
GP	General Practitioner
HBcAb	Hepatitis B core antibody
HBsAb	Hepatitis B surface antibody
HBsAg	Hepatitis B surface antigen
HBV	Hepatitis B virus
HBV-DNA	Serum hepatitis B DNA assay
HCV	Hepatitis C virus
HER2	Human Epidermal Growth Factor Receptor 2
IB	Investigators Brochure
ICH-GCP	International Conference on Harmonisation – Good Clinical Practice
ICF	Informed Consent Form
IEC	Independent Ethical Committee
IJB	Institut Jules Bordet
IMP	Investigational Medicinal Product
IRB	Institutional Review Board
LLN	Lower limit of normal
MedDRA	Medical Dictionary for Regulatory Activities
MBC	Metastatic breast cancer
MNR	Metabolic non-response

MNRP	Metabolic non responder patients
MR	Metabolic Response
MRP	Metabolic responder patients
mTOR	Mammalian target of rapamycin
mTORC1	Mammalian target of rapamycin complex 1
NCI-CTCAE	National Cancer Institute - Common Terminology Criteria for Adverse Events
NSAI	Non-steroidal aromatase inhibitor
OS	Overall Survival
PFS	Progression Free Survival
PgP	P-glycoprotein
PI3K	Phosphatidylinositol 3-kinases
PR	Progesterone receptor
PS	Performance Status
PT	Preferred Term
RECIST	Response Evaluation Criteria In Solid Tumors
ROI	Region of Interest
S6K1	S6 ribosomal protein kinase
SAE	Serious Adverse Event
SAR	Serious Adverse Reaction
SERM	Selective Estrogen Receptor Modulator
SmPC	Summary of Product Characteristics
SUSAR	Suspected Unexpected Serious Adverse Reaction
SUV	Standardized Uptake Value
T_{max}	Time to peak concentration
TTP	Time to Progression
Vd	Volume of distribution

1. BACKGROUND AND SCIENTIFIC RATIONALE

1.1. Epidemiology of breast cancer

Breast cancer is the leading cause of cancer related death in western countries[1]. In Europe, new breast cancer diagnoses made up approximately 13% of all new cases of cancer diagnosed in 2004[2]. Endocrine responsive (ER+) tumors, encompassing estrogen receptor (ER) and/or progesterone receptor (PR) positive tumors constitute approximately 70% of all breast cancers (BC) and thus anti-estrogen therapies have been developed[3,4]. Despite these treatments approximately 30 % of patients diagnosed with early breast cancer will develop metastatic disease[5].

1.2. Treatment options for endocrine responsive breast cancer

Endocrine therapies namely selective estrogen receptor modulator (SERM) agents tamoxifen and fulvestrant, and aromatase inhibitors (AI) anastrozole, letrozole and exemestane, have been the hallmark of ER+ disease producing tremendous improvements in both recurrence (approximately 30%) and mortality (approximately 20%) in early stage disease as well as significant gains in clinical outcomes in advanced disease [6,7].

Tamoxifen antagonises endocrine receptor functions. It has been the main form of anti-estrogen therapy for approximately 30 years and is indicated across all aspects of endocrine responsive breast cancer.

Aromatase inhibitors in contrast reduce peripheral estrogen synthesis by blocking the conversion of androgens to estrogens, which is the primary estrogen production pathway in post-menopausal women. Third generation aromatase inhibitors are currently approved for postmenopausal women with hormone receptor positive BC in early and advanced breast cancer (ABC). AI's are classified into two groups: non-steroidal aromatase inhibitors (NSAI), letrozole (Femara®) and anastrozole (Arimidex®) and steroidal aromatase-inactivator, exemestane (Aromasin®).

Anastrozole is indicated across the entire continuum of breast cancer. In the adjuvant setting efficacy has been shown in the treatment of postmenopausal women with hormone receptor positive early breast cancer (prolonged DFS with anastrozole vs. tamoxifen, HR 0.83, 95% CI 0.73 to 0.94, p = 0.0058, 9366 patients) (ATAC) [8]. In Europe, it is indicated in the adjuvant treatment of early breast cancer in hormone receptor positive postmenopausal women who have received 2 to 3 years of adjuvant tamoxifen (higher event free survival for anastrozole, HR 0.60, 95% CI 0.44 to 0.81; p = 0.0009) [9]. In ABC and MBC it is indicated as first-line treatment of postmenopausal women who are hormone receptor positive (superiority was shown for anastrozole vs. tamoxifen for progression free survival (PFS) HR: 0.90; 95% CI: 0.82 to 0.99; p=0.025) [10,11]. In the USA, anastrozole is also indicated for the treatment of ABC in postmenopausal women with disease progression following tamoxifen therapy based on the significant survival advantage of anastrozole 1 mg over megestrol acetate (HR 0.78, 97.5% CI 0.60 to < 1.0, P < 0.025) [12].

Letrozole, like anastrozole, is also indicated across the entire continuum of breast cancer. In the adjuvant setting it has been compared with tamoxifen. The results show that letrozole significantly prolonged disease-free survival (HR 0.81; 95% CI 0.70-0.93; p = 0.003, 8010 patients) (BIG- 1-98)[13]. Extended adjuvant treatment with letrozole after 5 years of adjuvant tamoxifen has shown improvements in local or metastatic recurrence and new contralateral breast cancers in postmenopausal women (HR in favour of letrozole vs. placebo: 0.57, 95% CI, 0.43 to 0.75; p = 0.00008, 5187 patients)[14]. In ABC and MBC, letrozole is approved for the first-line treatment of postmenopausal women with hormone receptor positive due to improvements in the time to progression (TTP) compared to

tamoxifen, (HR = 0.72, $p < 0.0001$, 907 patients) [6]. Additionally it is used as second line therapy after anti-estrogen therapy in postmenopausal women with ABC or MBC (Risk Ratio for TTP in favor of letrozole vs. megestrol acetate: 0.80; 95% CI 0.63 to 1.02; p = 0.07) [15].

Exemestane is an irreversible steroidal aromatase inactivator that has demonstrated efficacy in the treatment of postmenopausal patients with ABC and MBC. It is indicated for adjuvant treatment of postmenopausal women with estrogen receptor positive early BC who have received two to three years of tamoxifen and are switched to exemestane for completion of a total of five consecutive years of adjuvant hormonal therapy (HR 0.76 for disease-free survival in favor of exemestane vs. tamoxifen, 95% CI 0.66 to 0.88, p = 0.0001, 4727 patients)[16]. In postmenopausal women with ABC exemestane is indicated if disease has progressed following tamoxifen therapy (in the USA) or following anti-estrogen therapy (in Europe). In a study of exemestane versus megestrol acetate in postmenopausal women who experienced failure of tamoxifen, exemestane showed improved survival over megestrol acetate (74.6 weeks vs. 55.0 weeks respectively) yielding an approximately 4.5-month survival advantage[17].

Fulvestrant (Faslodex®), an estrogen receptor antagonist, is also approved for the treatment of hormone receptor positive metastatic breast cancer (MBC) in postmenopausal women with disease progression following anti-estrogen therapy. In ABC whose disease has progressed on prior endocrine treatment, fulvestrant was compared to anastrozole and the results revealed no significant difference for time to progression between the two treatment groups (HR, 0.92; 95% CI 0.74 to 1.14; p = 0.43) [18]. However, a phase II trial in postmenopausal women with measurable disease who had experienced disease progression after treatment with a third-generation AI and, at most, one additional hormonal agent, revealed anti-tumor activity of fulvestrant with an observed response rate of 14.3% (90% CI 8.2% to 22.5%) [19].

Despite these innovations, a significant number of patients do not benefit from these treatments. Approximately 50% of ER+ BC does not respond initially to anti-estrogens thereby exhibiting *de novo* resistance [20]. Particular patients with ER+ advanced BC benefit from long term sequencing of endocrine therapy and thus delaying the initiation of more toxic treatment options such as chemotherapy. However, the problem arises when these patients develop resistance to hormone therapies (acquired resistance). As a consequence of exhausted hormonal manipulations and limited other therapeutic alternatives, these patients are then initiated on chemotherapy in order to continue to control their disease and minimize disease progression. Whilst physicians can diagnose this phenomenon of endocrine resistance, to date no alternatives to chemotherapy are available in clinical practice.

1.3. <u>Bidirectional cross talk of ER</u>

Significant research efforts have been made into targeted therapy for BC patients. Along with these molecular discoveries has come an increased understanding of endocrine resistance pathways in ER+ disease. It is now well established that the ER has the capability for bidirectional cross talk with other signalling pathways. EGFR-1 and HER-2 along with intracellular kinases, notably PI3K/Akt/mTOR are implicated in escape mechanisms that bypass endocrine responsiveness via activating nuclear ER transcriptional activity [21]. The PI3 kinase/Akt/mTOR pathway is activated in aromatase inhibitor resistant and long-term estrogen deprived BC cells [22–24]. Selective inhibitors of mTOR, sirolimus or rapamycin, demonstrated a significant growth inhibition particularly in long-term estrogen deprivation BC cells [25].

More specifically this transcription activation is thought to occur via the 40S ribosomal S6 Kinase 1 (S6K1), one of the best characterized downstream targets of mTOR ultimately highlighting a potential targeted co-therapy option [26].

Both preclinical and clinical work supports the rationale for combination of endocrine therapy with mTOR inhibitors. The combination of letrozole and everolimus produced maximal and synergistic effects on inhibition of tumor growth in estrogen dependent BC cells (MCF-7 wild type and MCF-7 aromatase expressing) [27]. Interestingly activation of Akt and the downstream mTOR pathway can cause resistance to tamoxifen. In experiments where MCF-7 cells were able to proliferate in conditions of estrogen deprivation, these cells became resistant to growth inhibition from tamoxifen. However, co-treatment with temsirolimus (a mTOR inhibitor) was able to restore sensitivity to tamoxifen further highlighting the close association of the mTOR pathway with endocrine resistant disease [28].

1.4. Overview of Everolimus

Everolimus was originally developed as an immunosuppressive agent in solid organ transplantation. Since 2002 it has been developed as an anti-cancer agent.

1.4.1. Mechanism of action

Everolimus is a selective mTOR (mammalian target of rapamycin) inhibitor[29]. mTOR is a key serine-threonine kinase, the activity of which is known to be up-regulated in a number of human cancers[30,31]. Everolimus binds to the intracellular protein FKBP-12, forming a complex that inhibits mTOR complex-1 (mTORC1) activity[32]. Inhibition of the mTORC1 signaling pathway interferes with the translation and synthesis of proteins by reducing the activity of S6 ribosomal protein kinase (S6K1) and eukaryotic elongation factor 4E-binding protein (4EBP-1) that regulate proteins involved in the cell cycle, angiogenesis and glycolysis[30]. Everolimus reduces levels of vascular endothelial growth factor (VEGF), which potentiates tumorangiogenic processes[33–36]. Everolimus is a potent inhibitor of the growth and proliferation of tumor cells, endothelial cells, fibroblasts and blood-vessel-associated smooth muscle cells and has been shown to reduce glycolysis in solid tumors in vitro and in vivo[36–38].

1.4.2. Pharmacokinetics of Everolimus

In patients with advanced solid tumors, peak everolimus concentrations (C_{max}) are reached at a median time of 1 hour after daily administration of 5 and 10 mg everolimus under fasting conditions or with a light fat-free snack. C_{max} is dose-proportional between 5 and 10 mg.

Everolimus is mainly metabolized by the cytochrome P450 isoenzyme 3A4 (CYP3A4) in the liver and to some extent in the intestinal wall. Everolimus is also a substrate of P-glycoprotein (PgP). Therefore, medicinal products that interact with CYP3A4 and/or PgP may influence absorption and subsequent elimination of systematically absorbed everolimus. Strong CYP3A inhibitors (such as ketoconazole, itraconazole, ritonavir) and inducers (such as rifampicin, rifabutin) should be avoided[29].

In healthy subjects, high fat meals reduced systemic exposure to everolimus 10 mg (as measured by AUC) by 22% and the peak plasma concentration C_{max} by 54%. Light fat meals reduced AUC by 32% and C_{max} by 42%. Food, however, had no apparent effect on the post absorption phase concentration-time profile.

The blood-to-plasma ratio of everolimus, which is concentration-dependent over the range of 5 to 5000 ng/ml, is 17% to 73%. Approximately 20% of the everolimus concentration in whole blood is confined to plasma of cancer patients given everolimus 10 mg/day. Plasma protein binding is approximately 74% both in healthy subjects and in patients with moderate hepatic impairment. In patients with advanced solid tumors, V_d was 191 L for the apparent central compartment and 517 for the apparent peripheral compartment.

Mean CL/F of everolimus after 10 mg daily dose in patients with advanced solid tumors was 24.5 l/h. The mean elimination half-life of everolimus is approximately 30 hours. No specific excretion studies have been undertaken in cancer patients; however, data are available from the studies in transplant patients. Following the administration of a single dose of radiolabelled everolimus in conjunction with cyclosporine, 80% of the radioactivity was recovered from the faeces, while 5% was excreted in the urine. The parent substance was not detected in urine or faeces.

After administration of everolimus in patients with advanced solid tumors, steady-state AUC0-τ was dose proportional over the range of 5 to 10 mg daily dose. Steady state was achieved within two weeks. C_{max} is dose proportional between 5 and 10 mg. t_{max} occurs at 1 to 2 hours post-dose. There was a significant correlation between $AUC_{0-\tau}$ and pre-dose trough concentration at steady-state.

1.5. Overview of Exemestane

See section 1.2 for indications for exemestane.

The recommended daily dose of exemestane is 25 mg via oral administration. Exemestane is rapidly absorbed from the gastrointestinal tract. Its bioavailability is limited by first-pass metabolism, but is increased when taken with food. Exemestane is widely distributed, and is extensively bound to plasma proteins. It appears to be more rapidly absorbed in women with breast cancer (t_{max} of 1.2 hours) than in healthy women (t_{max} of 2.9 hours). The terminal half-life for exemestane is 18-24 hours. The time needed to reach maximal E2 suppression is 7 days[39,40]. Exemestane is metabolized by CYP3A4 and aldoketoreductases. It does not inhibit any of the major CYP isoenzymes, including CYP 1A2, 2C9, 2D6, 2E1 and 3A4. While no formal drug-drug interaction studies have been conducted, significant effects on exemestane clearance by CYP isoenzyme inhibitors appear unlikely [39,41].

1.6. Clinical trial experience: Everolimus

Everolimus is currently indicated for metastatic renal cell carcinoma. The RECORD-1, a phase III, international, multicentre, randomised, double-blind study comparing everolimus 10 mg/day and placebo, both in conjunction with best supportive care, was conducted in patients with metastatic renal cell carcinoma whose disease had progressed on or after treatment with VEGFR-TKI (vascular endothelial growth factor receptor tyrosine kinase inhibitor) therapy (sunitinib, sorafenib, or both sunitinib and sorafenib). After documented radiological progression, patients could be unblinded by the investigator: those randomised to placebo were then able to receive open-label everolimus 10 mg/day. The Independent Data Monitoring Committee recommended termination of this trial at the time of the second interim analysis as the primary endpoint had been met.

In total, 416 patients were randomised 2:1 to receive everolimus (n=277) or placebo (n=139). The median duration of blinded study treatment was 141 days (range 19-451 days) for patients receiving everolimus and 60 days (range 21-295 days) for those receiving placebo. Everolimus was superior to placebo for the primary endpoint of progression-free survival (hazard ratio 0.30, 95% CI 0.22-0.40, p<0.0001),with a statistically significant 67% reduction in the risk of progression or death[42].

RADIANT-3 (study CRAD001C2324), a phase III, multicentre, randomised, double-blind study of Everolimus plus best supportive care (BSC) versus placebo plus BSC in patients with advanced neuroendocrine tumors of pancreatic origin (pNET), demonstrated a statistically significant clinical benefit of everolimus over placebo by a 2.4-fold prolongation of median progression-free-survival (PFS) (11.04 months versus 4.6 months), (HR 0.35; 95% CI: 0.27, 0.45; p<0.0001)[43].

Clinically, some of the most compelling evidence for ER cross talk arises from BOLERO-2 which was designed to further explore overcoming endocrine resistance mechanisms by coupling an aromatase and mTOR inhibitor[44]. This was a Phase III, double-blind, multi-centre randomized controlled trial of 724 patients with ER+ advanced BC comparing everolimus plus exemestane versus exemestane plus placebo (patients were randomly assigned in a 2:1 ratio) in patients who had a recurrence or progression while receiving NSAI therapy in the adjuvant setting or to treat advanced disease or both. The primary endpoint was PFS. Approximately 50% of patients in both arms had used ≥ 3 previous therapies. An interim analysis revealed that the median progression-free survival was 10.6 months with everolimus plus exemestane and 4.1 months with placebo plus exemestane, by central assessment (hazard ratio for progression or death, 0.36; 95% CI 0.27 to 0.47; P<0.001). Response rates on the basis of central assessment were 7.0% and 0.4% in the combination group and exemestane alone groups respectively (P<0.001). Overall survival results were immature at the time of the interim analysis, with a total of 83 deaths: 10.7% in the combination therapy group and 13% in the exemestane alone group died.

There is also evidence for a benefit of Everolimus in the phase II neoadjuvant trial of letrozole plus everolimus versus letrozole alone with improvements in response rates by clinical palpation for the combination arm (68.1% *v* 59.1% single side P=0.062) [45,46].

From these trials it is evident that the addition of everolimus to aromatase inhibitors results in improved clinical outcomes. However this comes at a cost of increased toxicity. Serious adverse events in BOLERO-2 were double in the combination (23% vs. 12%) with the most common toxicities of any grade, in the combination arm.The most common adverse events for combination therapy were stomatitis (56% vs. 11%), rash (36% vs. 6%), fatigue (33% vs. 26%), diarrhea (30% vs. 16%), decreased appetite (29% vs10%), nausea (27% vs. 27%), cough (22% vs. 11%), dysgeusia (21% vs5%), headache (19% vs. 13%), decreased weight (19% vs. 5%). A significantly higher percentage of patients discontinued treatment in the combination arm (19% vs. 4%) with 11% vs. 1% of the discontinuation attributed to study treatment in the combination and exemestane alone arms respectively[44]. This significantly increased number of adverse events was also seen in the neoadjuvant trial of letrozole plus everolimus versus letrozole plus placebo where 22.6% of patients in the combination arm experienced a grade 3 or 4 adverse event versus 3.8% in the letrozole alone arm [46].

1.7. <u>The role of FDG-PET in BC</u>

To date few studies have evaluated the utility of Fluorodeoxyglucose-Positron Emission Tomography (FDG-PET) in advanced BC with fewer studies of FDG-PET in BC patients treated with targeted therapies. Small studies of less than 20 patients have been conducted in chemotherapy treated BC patients with promising results for the predicative potential of FDG-PET [47,48].

An early FDG-PET study of 11 patients treated with neoadjuvant letrozole conducted by Ueda et al in Japan demonstrated the correlation between FDG-PET metabolic responders at 4 weeks and change in tumor size as measured by ultrasound, pathological response, and change in Ki-67 labelling index compared to FDG-PET metabolic non-responders [49]. This is supported by two small studies [50,51]; one in 22 patients with MBC treated with tamoxifen, an AI or progestogen and the second where patients were treated with neoadjuvantletrozole and bevacizumab. Not only was FDG-PET metabolic response correlated to clinical/pathological outcomes, it is also important to note that these AI studies of PET, although small, did not demonstrate the metabolic flare previously observed with tamoxifen[52]. This is an essential concern to clarify in AI treated patients as early PET evaluations with modern day EORTC criteria of metabolic response could derive false negatives due to the metabolic flare phenomenon [53].

An exploratory sub-study of NeoALTTO, a neoadjuvant trial evaluating combinations of trastuzumab and/or lapatinib with paclitaxel, reviewed the role of FDG-PET as a possible biomarker in HER2 + disease in 77 of the 455 patients included in NeoALTTO[54]. Metabolic changes in the primary tumor in patients with invasive operable BC treated with anti-HER2 therapies were detected as early as after 2 weeks of treatment. Additionally, pathological complete response (pCR) rates were twice as high in PET responders compared to non-responders according to EORTC criteria (41% versus 21% at week 2 and 43% versus 19% at week 6). While this sub-study is too small to draw definitive conclusions, FDG-PET early response may be a promising tool particularly for clinical development of new drugs or combinations[55]. The role of FDG-PET as a biomarker is reinforced by early patient and xenograft studies exposed to an mTOR inhibitor in various tumor groups [56–60].

1.8. Study rationale

Given this higher level of adverse events in trials combining aromatase inhibitors with mTOR inhibitors, **selecting patients who will achieve a benefit and thus justify the burden of treatment is of upmost importance**. It is clear that this drug combination has a role in endocrine resistant patients. What is unclear is in which patients the clinical benefit rationalizes the potential toxicity. What is problematic is that unlike HER2+ BC, there is an absence of established biomarkers in this population that will identify patients that will sustain this benefit and thus it is unclear whom to expose to this increased toxicity.

FDG-PET/CT offers a possible solution to early identification of patients who will NOT benefit from treatment with exemestane and everolimus. Thus a phase II trial evaluating the use of FDG-PET/CT as a biomarker in patients with ER+ locally advanced and MBC treated with exemestane plus everolimus will answer the question of whether patients who will NOT derive benefit from this combination of therapy can be selected early in the therapeutic process using FDG-PET/CT metabolic response thus minimizing unnecessary toxicities in these patients and high costs to society.

2. STUDY OBJECTIVES

2.1. Primary Objective

To evaluate if early metabolic response (MR) using FDG-PET/CT is associated with progression free survival (PFS) in ER+, HER2 negative ABC or MBC patients treated with exemestane plus everolimus.

2.2. Secondary Objective

To evaluate if early metabolic response (MR) using FDG-PET/CT is associated with overall survival (OS) in ER+, HER2 negative locally advanced or MBC patients treated with exemestane plus everolimus.

3. STUDY DESIGN AND ENDPOINTS

3.1. Overview of Study Design

This study is a prospective phase II single arm, open label, multicentre study evaluating early FDG-PET/CT response as a predictor of treatment response to exemestane (25mg daily orally) and everolimus (10mg daily orally) in patients refractory to NSAI in locally advanced and MBC.

3.2. Screening Phase

After signed consent patients will undergo screening as outlined in tables section 5.6.

Screening for hepatitis B (HBV) and hepatitis C (HCV) is mandatory due to the risk of HBV and HCV reactivation and associated mortality **(See Appendix 1 and 2)**.

Screening evaluations will be performed within 21 days prior to treatment Day 1 except for patients with positive baseline hepatitis B or C results for whom the screening period can be extended to 28 days to allow time for prophylactic treatment (see Appendix 1 and 2).

To participate in the trial, a patient must fulfil all inclusion criteria and must not meet any of the exclusion criteria.

3.3. Treatment

3.3.1. Pilot Phase

The aim of the pilot phase is to verify the hypothesis that roughly 1 in 5 patients is an FDG-PET/CT non-responder and to determine the optimum timing for the second FDG-PET/CT assessment (2 weeks versus 4 weeks after initiation of treatment). Thus, patients will undergo 3 FDG-PET/CT scans. Concordance-analysis using Kappa coefficient (c.f. Koch and Landis scale) between FDG-PET-responses seen at 2 versus 4 weeks will be performed. In case of concordance rate of more than 90% week 2 FDG-PET/CT will be chosen for the main study. In the case of non-concordance we will choose the timepoint where the mean absolute change in SUV_{max} will be the highest. This decision rule will be applied blinded from any clinical data so that these patients can be used in the main study.

The pilot phase was conducted and recruited 27 evaluable patients (instead of the 30 patients initially planned. Accrual was stopped after 30 evaluable patients included but 3 patients were denoted inevaluable afterwards). All patients received everolimus 10mg daily plus exemestane 25mg daily and treatment has been continued until disease progression or unacceptable toxicity or consent withdrawal, whichever came first.

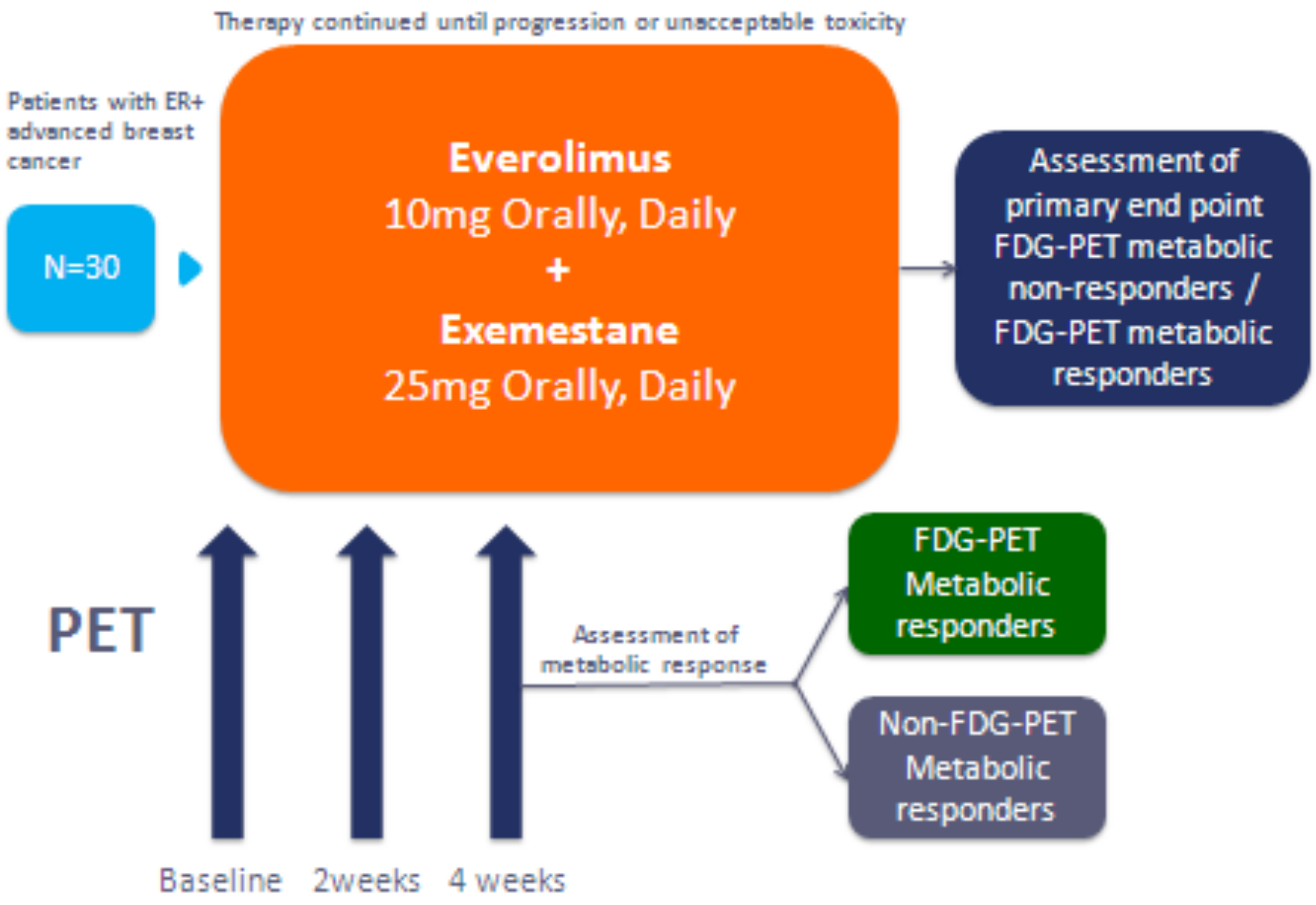

Figure 1. Pilot Study Schema

3.3.1.1. Endpoints for pilot phase

Primary endpoint: Proportion of FDG-PET/CT metabolic non-responders/FDG-PET/CT metabolic responders

Secondary Endpoint: Estimate the most suitable second FDG-PET/CT time point (2 weeks versus 4 weeks after initiation of treatment).

3.3.2. Main Study

As mentioned above the aim of the pilot phase was to characterize FDG-PET/CT responses in locally advanced or metastatic breast cancer patients treated with everolimus and exemestane and to establish which is the most suitable time point for the early-FDG-PET/CT (D14 vs D28 after the initiation of treatment). Results of this pilot phase are now available and led to the following findings:

- The metabolic non-response rate on Day 14 and Day 28 was similar: 59% (16/27) (Kappa coefficient for concordance: 0.85 (95% CI, 0.64 to 1); mean SUV decrease at day 14: 26%, at day 28: 32%-p-0.03). The assessment of response was blinded from any clinical data so that these patients can be used for the main study
- Patient population highly representative of real life regarding the number of previous treatments, metastatic sites and toxicity profile of everolimus. The sample size is too small to perform any subgroup analysis at this stage
- Median PFS in all 27 patients: 6.6 months, the disease control rate at the first RECIST evaluation was 66%: (18% of patients had partial response and 48% stable disease). The median duration of everolimus treatment was 6 month (range: 1-17 month). We do not have enough statistical power with this sample size to assess PFS in FDG-PET-responders and non-responders separately, hence the need to proceed to the expansion phase

After the completion of the pilot study the results were reviewed in collaboration with the Novartis® team and the decision to proceed to the full patient enrolment of the main study was taken. The Investigators will remain blinded to these results.

We decided to continue the day 14 FDG-PET/CT for the second phase, given the high concordance between the two time points used in the pilot phase and the aim to reduce at maximum treatment exposure and costs. According to the observed metabolic non-response rate in the pilot phase (59%), 19 more evaluable patients are needed to complete the trial (meaning approximately 30 patients screened) and to answer the primary endpoint.

Again, all patients will receive everolimus 10mg daily plus exemestane 25mg daily. In this instance patients will have 2 FDG-PET/CT: at baseline and at the time point determined by the results of the pilot study, which will be at the end of week 2 (D14). Note that the clinician will be blinded to the FDG-PET/CT result and the FDG-PET/CT physicians to the clinical follow-up.

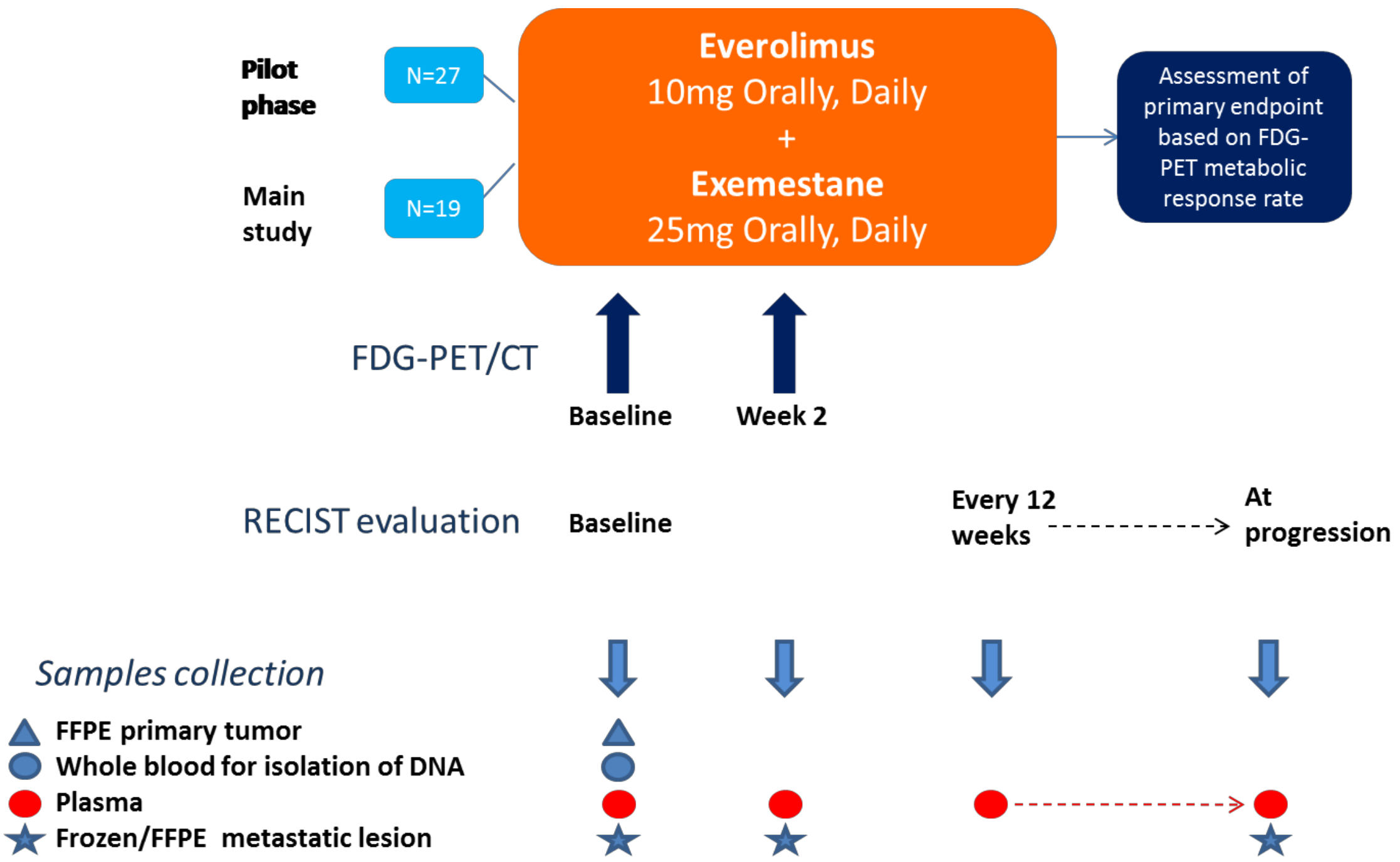

Figure 2. Main Study Schema

Study treatment will continue until disease progression, unacceptable toxicity or consent withdrawal. Further treatment after progression will be at the investigators discretion.

3.3.2.1. Endpoints for main study phase

<u>Primary Endpoint</u>: Progression free survival (PFS) (based on RECIST 1.1 criteria)

<u>Secondary Endpoint</u>: Overall survival (OS)

3.3.3. Definitions

This study is aimed at early identification of metabolic non-responders. FDG-PET/CT response will use the following methodology based on dominance response and PERCIST criteria [53,61].

Response assessment will be performed by 2 independent and blinded FDG-PET/CT experts. Consensus reading will be organized in case of discordant assessments. All analyses will be organized and coordinated by a central Imaging Core Lab (ORILaB).

In order to ensure image quality and reproducibility, standardization and harmonization, the participating FDG-PET/CT centres are required to have FDG-PET/CT Accreditation through EARL (EANM Research Ltd.) and need to comply with the requirements of continued quality

control (QC) of acquisition and reconstruction on regular basis as outlined in EARL's accreditation manual. More information could be obtained via the EARL's web site: http://earl.eanm.org.

3.3.3.1. Definition of metabolic target lesions on FDG PET/CT

Target lesions are defined on the baseline FDG-PET/CT based on all of the following criteria:

a. Lesion with a very high probability of malignancy (all lesions potentially corresponding with a benign pathology should not be selected); AND
b. Size ≥1.5 cm (preferably in anatomic images); AND
c. FDG-PET avid lesion with uptake above the background liver uptake as described below:
   i.e. with a marked accumulation of FDG, at least 1.5-fold greater than liver SUV mean + 2 SDs (in 3-cm spherical ROI in normal right lobe of liver). If liver is abnormal, target lesion should have uptake > 2.0 x SUV mean of blood pool in 1-cm-diameter ROI in descending thoracic aorta)

3.3.3.2. Definition of FDG-PET/CT response

Different response classification methodologies will be performed:

a. *Dominance Method* (*Serial FDG-PET/CT for early outcome prediction in patients with metastatic colorectal cancer undergoing chemotherapy, Hendlisz et al. Annals of Oncology 2011*)
   Patient-based classification of metabolic response within 4 classes:
   Class 1: All target lesions do respond (reduction of SUV_{max} of more than 25% seen in all lesions)
   Class 2: Mixed response with majority of whole body tumor load that responds
   Class 3: Mixed response with majority of whole body tumor load that does not respond
   Class 4: All target lesions do not respond OR presence of a progressive lesion (i.e. increase of SUV_{max} of more than 25% in a known lesion or appearance of a new FDG positive lesion)
b. *PERCIST-based response assessment*:
   A maximum of 10 target lesions (maximum of 2 per organ) will be identified. If more than 2 target lesions within one organ are available: only 2 lesions with the highest SUV will be defined as target lesions.
   SUV_{peak} at baseline and during the treatment will be calculated for all target lesions.
   Significant lesion response is defined as a relative decrease of SUV_{peak} of more than 30% therefore patient will be classified as followed:
   - Metabolic responder patient (MRP): the difference between the SUV_{peak} of the hottest lesion at baseline and during treatment is more than 30%;
   - Metabolic NON responder patient (MNRP): patient that does not fulfil the definition of MRP.

The primary method for defining response is the dominance method.

3.3.3.3. ORILaB – Imaging CoreLAB

FDG-PET/CT images generated in this study are managed by the Oncology Related Imaging coreLAB (ORILaB) from the Institute Jules Bordet. ORILaB will provide participating centers a Standard Procedures Imaging Manual (SPIM) describing the procedures involved in patient preparation, data acquisition and reconstruction, data transfer and storage. ORILaB will be in charge of controlling the quality of the imaging data and coordinating the central review.

3.3.3.4. Imaging follow-up

Patients will have imaging with MRI or CT scan at baseline and every 12 weeks to assess progression according to RECIST 1.1; whenever possible the same imaging method should be used along the study. In addition patients with easily accessible metastases should have a biopsy at baseline and an optional one at disease progression and/or blood samples collection (see Section 5.5 Biomarker Assessment for details).

3.4. Follow-up

Patients will be followed for safety until 28 days after study treatment discontinuation (end of treatment visit). After treatment discontinuation for any reasons the next line of chemotherapy should be captured and the patient will no longer be followed as per schedule of assessments. Only survival information will be collected until death.

For patients who discontinue study treatment for reasons other than progression, tumor assessments and patient visits will continue with the same schedule. (see Section 5).

3.5. Survival Data Collection

After progression or after study treatment discontinuation, which means after the end of treatment visit, patients will continue to be followed for survival every three months.

3.6. End of Study

This is an event driven analysis. The analysis for the primary endpoint will be performed after 42 PFS events have occurred. The study will end when all OS events have been reported and the database has been fully cleaned and frozen for all analyses.

3.7. Number of patients

For the pilot phase:

As already mentioned before, the pilot phase planned to include 30 patients but finally 27 evaluable patients were included out of 44 patients screened (Accrual was stopped after 30 evaluable patients included but 3 patients were denoted inevaluable afterwards).

For the main phase:

Pilot phase's results showed that the metabolic non-response rate on Day 14 and Day 28 was similar: 59% (16/27) (Kappa coefficient for concordance: 0.85 (95% CI, 0.64 to 1); mean SUV decrease at day 14: 26%, at day 28: 32%-p-0.03). According to this metabolic non-response rate higher than previously assumed (i.e. 59% instead of 20%), 42 PFS event are needed. At an accrual rate of 1-2 evaluable patients per month, 46 evaluable patients in total are needed. This means 19 (=46-27(pilot)) more evaluable patients are needed to answer the primary endpoint of the trial. If we consider the screen failure rate observed in the pilot phase, about 30 patients need to be screened to complete the study.

3.8. Centres

Patients will be enrolled in 6 centres across Belgium for the pilot phase. For the main phase the enrolment will follow in 5 centres.

4. SELECTION AND WITHDRAWAL OF SUBJECTS

4.1. Overview

The study population for this trial is patients who have locally advanced or metastatic hormone receptor positive, HER2 negative breast cancer that is endocrine resistant and that will be treated with exemestane and everolimus.

4.2. Inclusion Criteria

Patients must meet ALL of the following criteria to be eligible for this study:

1. Adult women (≥18 years of age) with locally advanced or metastatic breast cancer not amenable to curative treatment by surgery or radiotherapy.
2. Histological or cytological confirmation of estrogen-receptor positive (ER+), HER2 negative breast cancer.
3. Postmenopausal female defined as:
   a. Age ≥ 55 years and one year or more of amenorrhoea
   b. Age <55 years and one year or more of amenorrhoea, with an estradiol assay <20pg/ml
   c. Surgical menopause with bilateral oophorectomy

   NOTE: Ovarian radiation or treatment with a luteinizing hormone-releasing hormone (LHRH) agonist (goserelin or leuprolide) is not permitted for induction of ovarian suppression.
2. Breast cancer that is refractory to non-steroidal aromatase inhibitors (NSAI) (i.e. anastrozole or letrozole) defined as:
   a. Recurrence while on, or within 12 months of end of adjuvant treatment with anastrozole or letrozole; OR
   b. Progression while on, or within one month of end of anastrozole or letrozole or treatment for locally advanced or metastatic breast cancer

   NOTE: Letrozole or anastrozole do not have to be the last treatment prior to enrolment.
3. FDG-PET/CT measurable disease defined as: at least one target lesion fulfilling following criteria:
   a. Size ≥ 1.5 cm; AND
   b. FDG-PET avid lesion with uptake above the background liver uptake as described below:

   i.e. with a marked accumulation of FDG, at least 1.5-fold greater than liver SUV mean + 2 SDs (in 3-cm spherical ROI in normal right lobe of liver). If liver is abnormal, target lesion should have uptake > 2.0 x SUV mean of blood pool in 1-cm-diameter ROI in descending thoracic aorta).
4. The patient must have at least 1 FDG-PET/CT measurable lesion also measurable according to RECIST 1.1 criteria.
5. Radiological or clinical evidence of recurrence or progression on last systemic therapy prior to enrolment.
6. Adequate bone marrow function as shown by:
   a. Haemoglobin (HgB) ≥ 9.0 g/dL
   b. ANC ≥ 1,500/mm3 (≥1.5) x 109/L)
   c. Platelets ≥ 100,000/mm3 (≥ 100x 109/L)
7. Adequate liver function as shown by:
   a. Alanine aminotransferase (ALT) and aspartate aminotransferase (AST) ≤ 2.5xULN (or ≤ 5 if hepatic metastases are present)
   b. Total serum bilirubin ≤ 1.5 x ULN (≤ 3 ULN for patients known to have Gilbert Syndrome)

8. Adequate renal function as shown by serum creatinine ≤ 1.5 x ULN
9. Fasting serum cholesterol, triglyceride s and glucose
   a. Fasting serum cholesterol ≤ 300 mg/dL or 7.75 mmol/L
   b. Fasting triglycerides ≤ 2.5 x ULN
   c. Fasting serum glucose < 1.5 x ULN

   NOTE: in the case any of these thresholds are exceeded, the patient can only be included after initiation of statin/insulin or oral hypoglycemic therapy and when the above mentioned values have been achieved.
10. Eastern Co-operative Oncology Group (ECOG) performance status ≤ 2.
11. Written and signed informed consent obtained before any trial related activity.
12. Availability of a FFPE core of primary breast tumor
13. Possibility to obtain the mandatory blood samples for the translational research studies.
14. For patients with accessible metastatic lesions, possibility to obtain the mandatory biopsy (FFPE and frozen) of a metastatic lesion.

4.3. Exclusion Criteria

Patients meeting any ONE of the following criteria are NOT eligible for the study:

1. HER2-overexpressing patients by local laboratory testing (IHC 3+ staining or in situ hybridization positive).
2. Patients with only non-measurable lesions by FDG-PET/CT (e.g. pleural effusion, ascites etc.).
3. Symptomatic visceral disease for example liver, pulmonary metastases or lymphangitis carcinomatosis.
4. Known hypersensitivity to mTOR inhibitors, e.g. sirolimus (rapamycin).
5. Another malignancy within 5 years prior to enrolment, with the exception of adequately treated in-situ carcinoma of the cervix, uteri, basal or squamous cell carcinoma or non-melanomatous skin cancer.
6. Radiotherapy within four weeks prior to enrolment except in case of localized radiotherapy for analgesic purpose or for lytic lesions at risk of fracture, which can then be completed within two weeks prior to enrolment. Patients must have recovered from radiotherapy toxicities prior to enrolment.
7. Currently receiving hormone replacement therapy, unless discontinued prior to enrolment.
8. Symptomatic brain metastases or other central nervous system metastases which are not controlled by local treatments.
9. Patients receiving concomitant immunosuppressive agents or chronic corticosteroid use at the time of study entry except in cases outlined below:
   a. Topical applications (e.g. rash)
   b. Inhaled sprays (e.g. obstructive airways disease)
   c. Eye drops
   d. Local injections (e.g. intra-articular)
   e. Stable low dose of corticosteroids for at least two weeks before enrolment.
10. Patients with known HIV seropositivity. Screening for HIV infection at baseline is not required.
11. Acute and chronic, active infectious disorders (except for Hepatitis B and Hepatitis C positive patients).
12. Active bleeding diathesis, or on oral anti-vitamin K medication (except low dose warfarin, LMWH and acetylsalicylic acid or equivalent, as long as the INR is ≤ 2.0).
13. Any severe uncontrolled medical conditions such as:
   a. Unstable angina pectoris, symptomatic congestive heart failure, myocardial infarction ≤ 6 months prior to enrolment, uncontrolled cardiac arrhythmia

b. Uncontrolled diabetes as defined by fasting glucose > 1.5 x ULN
c. Acute and chronic, active infectious disorders and non-malignant medical illnesses that are uncontrolled or whose control may be jeopardized by the complications of this study therapy
d. Symptomatic deterioration of lung function

14. Patients being treated with drugs recognized as being strong inhibitors or inducers of the isoenzyme CYP3A (Rifabutin, Rifampicin, Clarithromycin, Ketoconazole, Itraconazoleonazole, Voriconazole, Ritonavir, Telithromycin) within the last 5 days prior to enrolment (see list of clinically relevant drug interaction listed in section 6.10).
15. History of non-compliance to medical regimens.
16. Patients unwilling or unable to comply with the protocol.
17. Concurrent anti-cancer treatment in another investigational trial, including hormonal therapy, immunotherapy or targeted agents.

4.4. Withdrawal of subjects

A patient may withdraw from the study or study specific procedures at any time during the entire duration of the study for any reason and without prejudicing future medical treatment.

Whilst a patient can withdraw without needing to give a reason, as soon as a patient has triggered a withdrawal, the investigator has the responsibility to establish that the patient's decision is an informed choice and to ascertain to what extent the patient might be willing to continue limited participation in the trial, e.g., being willing to continue being contacted or seen to provide follow-up information.

The outcome of the discussion should be documented in both the patient's medical records and the CRF.

It is therefore important to clarify that patient's withdrawal is defined within 4 different scenarios that have a different impact on the study analysis and data collection:

1. Withdrawal from study treatment but decision to continue with protocol assessments: the decision to withdraw from treatment can be taken by the patient or by the investigator. Patients must be kept on study and followed up according to the protocol schedule of assessments until study completion. When study treatment is prematurely discontinued, patients should complete the end of treatment visit 28 days after the last administration of study treatment. Moreover the reason for treatment discontinuation must be recorded on the CRF.
   a. If the patient decides to withdraw from treatment because of an AE, the reason for treatment discontinuation should be reported as "Adverse Event" even in the case the investigator does not consider the AE as qualifying for treatment withdrawal as per protocol.
   b. Investigators may withdraw patients from study treatment in the event of intercurrent illness, AEs, treatment failure, protocol violation, administrative reasons or for other reasons.
2. Withdrawal from the entire study: should a patient decide to withdraw from the study, all efforts will be made to complete and report the observations as thoroughly as possible. No further data will be collected after the date of withdrawal from study The Investigator should contact the patient or a responsible relative by telephone or through a personal visit to establish as completely as possible the reason for the withdrawal. A complete final evaluation at the time of the patient's withdrawal should be made with an explanation of why the patient is withdrawing from the study.
3. Partial withdrawal from the study, with consent to allow collection of progression and survival status information: all of the above points (withdrawal from the entire study) are applicable to this scenario with the exception that the patient accepts to be contacted for further information on progression as per the primary study endpoint

and survival status. It should be documented in both the medical records and in the CRF that the patient accepted to be contacted for survival despite she withdraw the study consent.

4. Lost to follow-up: In the case of patients who miss scheduled visits, several attempts should be made by the site to contact these patients for follow up information. The collection of follow up data is extremely important in regards to the reliable estimation of study endpoints, therefore at least 3 attempts within a reasonable extent of time should be made to try to contact the patients if they do not attend clinic visits. If any of the trial patients are lost to follow up, contact will initially be attempted through the trial research nurse or the lead investigator at each centre. Where this attempt is unsuccessful, the patient's GP will be contacted and asked to contact the patient or her/his family and provide follow-up information to the recruiting centre. It is only after sufficient attempts at contact have been unsuccessful, that a patient may be declared "Lost to follow-up".

5. SCHEDULE OF ASSESSMENTS AND PROCEDURES

The complete schedule of assessments is outlined in the tables section 5.6. and assessments marked with an 'X' indicates when they are to be performed, those marked with brackets [X] are optional, at investigator's discretion.

Tests procedures and visits should occur on schedule ± 7 days unless otherwise stated.

5.1. Screening and baseline

Screening assessments to confirm eligibility must be performed prior to the first dose of study drug. Physical examination including performance status, height and weight must be performed within 21 days prior to the first dose of study treatment. Patients with potassium, sodium and or calcium levels that are below the LLN at screening must have their potassium, sodium and/or calcium replenished through supplementation and the levels must be within normal limits prior to the first dose of study drug. The last reading will be recorded in the CRF.
At baseline haematology, biochemistry, calcium, magnesium and phosphate, liver function tests, LDH, lipid profile and fasting blood glucose level should be completed within 7 days of commencement of study treatment.
In addition pulmonary functions tests (PFTs) can be performed at screening or Day 1 (prior to the administration of study drug) and if clinically indicated to exclude or manage non-infectious pneumonitis.

Baseline FDG-PET/CT will be completed at a maximum of 7 days prior to starting treatment with everolimus and exemestane.

Remark: Patients who previously received CDK 4/6 inhibitors are eligible.

5.2. Treatment

5.2.1. Pilot phase

Patients will undergo a screening visit, followed by a visit on day 1 of week 1, week 5 (Day 29) and week 9 (Day 57) and then every 8 weeks until treatment completion. Additional to this, patients will need to present for haematology and biochemistry blood sampling on week 2 (day 8).

FDG-PET/CT will be performed at the end of week 2 (day 14 - day17) and at the end of week 4 (day 28 - day31) during the pilot phase. At time of FDG injection glycemia will be measured as everolimus can impact on insulinemia/glycemia and therefore indirectly on the SUV of the FDG uptake.

All attempts should be made to complete the visits within ± 7 days of the scheduled study visit as indicated on the schedule of assessments.

All patients will be followed for safety from the first day of treatment until the end of treatment visit (28 days after cessation of everolimus and exemestane treatment).

5.2.1. Main phase

Patients will undergo a screening visit, followed by a visit on day 1, day 14, day 28 and then every 12 weeks (from study treatment start) until treatment completion.

FDG-PET/CT will be performed at the end of week 2 (day 14 – day 17). At time of FDG injection glycemia will be measured as everolimus can impact on insulinemia/glycemia and therefore indirectly on the SUV of the FDG uptake.

All attempts should be made to complete the visits within ± 7 days of the scheduled study visit as indicated on the schedule of assessments.

All patients will be followed for safety from the first day of treatment until the end of treatment visit (28 days after cessation of everolimus and exemestane treatment).

5.3. End of treatment visit

End of treatment (EOT) visit is 28 days after the cessation of everolimus and exemestane.

5.4. Survival Assessment

Patients will continue to be followed for survival every 3 months (started from EOT visit) until:

- death OR
- consent withdrawal or
- the patient is lost to follow-up or
- the study ends (i.e. when 90% of OS have been reported, excluding patients lost to follow-up).

Patients who have withdrawn from the treatment or who have partially withdrawal from the study, with consent to allow collection of progression and survival status information, will also be followed for survival every 3 months. Survival information can be obtained via phone contact and should be documented in the patient's file and the CRF.

5.5. Biomarker Assessment

5.5.1. Background

The advent of next generation sequencing (NGS) has allowed investigators to decipher the genomic architecture of the tumour and link treatment resistance with tumor heterogeneity[62].

Currently, there are no available biomarkers to identify patients that could benefit from the combination of exemestane + everolimus and for whom the potential increased toxicity is justified or conversely identify patients that could be spared from the increased treatment toxicity. The BOLERO 2 team conducted also a robust translational research project

analysing by next generation sequencing 302 archival tumor samples collected from patients participating to the study. The efficacy of everolimus was independent of the activation of commonly mutated genes in luminal breast cancer, such as PIK3CA, FGFR and CCND1. To note that only 20% of the samples originated from metastatic lesions (Hortobagyi et al, J Clin Oncol 2016).

5.5.2. Primary tumor / Blood-based translational research

5.5.2.1. Aims

To identify biomarkers predicting resistance to exemestane and everolimus therapy by studying primary tumor, metastatic biopsies and serial plasma samples.

The results will be correlated with the results of early FDG-PET/CT data in order to better characterise the non-responders.

5.5.2.2. Samples collection:

The following samples will be collected: (See tables section 5.6.).

Samples processing procedures will be described in the "Guidelines for human biological material handling" that will be provided to the sites at the time of site activation.

- FFPE of the primary breast tumor will be collected at screening (mandatory for all patients enrolled in the PEARL study).
- Biopsy of metastatic lesions is mandatory at baseline for patients with accessible lesions (preferably FDG-PET scan avid lesions) enrolled in the PEARL study.
  Biopsies after the D14 FDG-PET/CT and at progression are optional. It is recommended that these biopsies to be collected from accessible ***non-metabolic responding*** sites.
  For each timepoint (at screening, at D14 FDG-PET/CT examination and at disease progression) the following samples will be collected:
  - Biopsy: FFPE tissue sample, (1 core);
  - Biopsy: frozen tissue, (2 cores).
- Whole blood: 7.5 ml of whole blood will be collected at screening in EDTA tubes and frozen at -20°C to be later processed for isolation of normal DNA (mandatory for all patients).
- Plasma: 7.5 ml of whole blood will be collected in EDTA tubes at the following time points:
  - At screening
  - At the second FDG-PET/CT examination i.e. at the end of week 2 (day 14) for both phases (pilot and main).
  - at all CT or MRI assessments
  - at disease progression

  These samples will be processed for plasma isolation (mandatory for all patients)
- Only for the pilot phase: Whole blood for CTC counting: 7.5ml of whole blood will be collected in CellSave tubes during screening for detection and isolation of CTCs. (mandatory and applicable only for patients enrolled at the Institute Jules Bordet, IJB).
  For patients with ≥5CTCs detected in the7.5ml screening sample, two additional blood draws of 7.5ml will be collected in CellSave tubes:
  - at the second FDG-PET/CT evaluation i.e.at the end of week 2 (day 14)
  - at disease progression.

  After the pilot phase, it was decided to not going further with the CTC analysis due to futility.

All samples will be shipped to the central laboratory (Jules Bordet Institute)

5.5.2.3. Methods

Plasma Samples

Plasma samples will be collected at the above mentioned timepoints and will be stored until the end of the recruitment period and will be shipped to the central laboratory (IJB) at the end of the study. Circulating tumor DNA, plasma proteomics and metabolomics will be analysed with the best technology available for this purpose at the end of the study.

Circulating Tumor Cells (CTCs)

Sample collection for CTC analysis was performed only for some patients enrolled at the Institute Jules Bordet, IJB in the pilot phase. Due to futility, it was decided to not going further with the CTC analysis.

5.5.3. Tissue samples

5.5.3.1. Methods

The samples, both the primary and the metastatic lesion biopsies when available, will be analysed using DNA, RNA sequencing and spatial transcriptomics with state of the art technologies. Results will be correlated with early-FDG-PET response and long term outcome. In selected cases, whole genome sequencing will be performed.

5.5.3.2. Statistical analysis

Molecular analysis of serial tumor samples (primary tumor and metastatic biopsies) associated to the analysis of serial plasma samples will allow to identify potential resistance mechanisms. The translational research is exploratory and hypothesis-generating. A more extensive statistical analysis plan will be prepared prior to data analysis.

5.5.4. Not yet specified translational research

The use of remaining biological material for not yet specified future research will be under the auspices of Institut Jules Bordet. The feasibility and validity of future translational proposals will be assessed by a trial specific research committee including the two principal investigators and members of the Breast Cancer Translational Laboratory and pending approval on the non-specified research by EC.

5.6. Table of schedule of assessments

Table 1: Pilot phase: Schedule of assessments

Non-mandatory assessments are shown in []	**Screening**	**Treatment Period**							**End of treatment visit[r]**	**Survival[s]**
**Week**	Prior to enrolment	**1**	**2**	**3**	**5**	**9**	**17 →… visit every 8 weeks**	**Every 12 weeks from study treatment start**		
**Day**		**1**	**8**	**15**	**29**	**57**				
Informed Consent[a]	X									
ER status[b]	X									
Demographic, Medical history[c]	X									
Physical examination, height and weight[d]	X	X			X	X	X		X	
ECOG[d]	X	X			X	X	X		X	
ECG[e]	X	[X]			[X]	[X]	[X]		[X]	
Pulmonary function tests (PFTs)[f]	[X]	[X]			[X]	[X]	[X]		[X]	
Haematology, Coagulation, Biochemistry, Calcium, magnesium, phosphate, liver function tests, LDH, lipid profile, fasting blood glucose level[g,h]	X	[X]	X		X	X	X		X	
Urinalysis[i]	X	[X]			[X]	[X]	[X]		[X]	
Hepatitis B and C serology (HBV-DNA, HBsAg, HBs Ab, HCV-RNA-PCR)[j]	X	[X]			[X]	[X]	[X]		[X]	
**Response Assessment**										
CT or MRI imaging (chest and abdomen) and/or CT/MRI brain[k]	X							X		
FDG-PET/CT[l]	X			X[m]	X[n]					
**Safety Assessment**										
Adverse events (AE's)[o]	X if applicable	Continuously								
Serious adverse events (SAE's)[p]	X if applicable	Continuously								
Concomitant medications[q]	X if applicable	Continuously								
**Survival**										
Survival status										X

a	Written informed consent must be obtained before any study specific screening assessments are performed.
b	ER status must be provided either on primary tumor pathology report or subsequent biopsies for metastatic disease. Repeat assessment/central testing is NOT required
c	Prior antineoplastic therapy (medication, radiation, surgery) will be collected
d	Physical examination and ECOG performance status are to be measured at baseline and monitored throughout the study.
e	Baseline standard 12 lead ECG and must be performed ≤14 days of the first dose of study medication and may be repeated at the investigators discretion during the study
f	PFTs can be performed at screening or Day 1 (prior to the administration of study drug) and if clinically indicated to exclude or manage non-infectious pneumonitis.
g	Haematology includes counts of haemoglobin, white blood cells, neutrophils, and platelets. Biochemistry includes serum creatinine, urea and electrolytes. Liver function tests include ALP, AST, ALT, total bilirubin. Coagulation: INR. Lipid profile: total serum cholesterol and triglycerides. Patients must be in a fasting state for lipid profile and fasting blood glucose level investigations. At baseline haematology, biochemistry, calcium, magnesium and phosphate, liver function tests, LDH, lipid profile and fasting blood glucose level **should be completed within 7 days of commencement of study treatment**. If haematology/biochemistry was already performed within1 week prior the start of the treatment, these tests do not need to be repeated at Day 1.
h	Haematology, biochemistry, calcium, magnesium, phosphate, liver function tests, LDH, lipid profile, fasting blood glucose level can be completed up to 7 days prior to next cycle of therapy while on study treatment.
i	Standard urinalysis dipstick assessment must include: protein, glucose, blood, ketones, and leukocytes and should be performed at local laboratory. This may be repeated and be supplemented with laboratory quantification of any potentially relevant abnormalities, after baseline as per investigator's discretion if clinically indicated.
j	Screening for hepatitis risk factors and any past illnesses of HBV and HCV is mandatory. See Appendix 1 and 2 for screening, monitoring and management of HBV and HCV
k	Chest, abdomen, pelvis CT's need to be repeated at baseline and every 12 weeks (including if negative at baseline) ± 7 days of scheduled date. Scans for complete and partial responses must be repeated at least at 4 weeks but no later than the next scheduled tumor assessment following the first documented response. Skin lesions should be photographed in addition to measuring. Brain scan (CT scan or MRI with I.V. contrast) at baseline if brain metastases are known and must also be performed if CNS symptoms are present. The imaging technique used for response assessment along the study should be the same method as used at screening.
l	All patients will have FDG-PET/CT at baseline (up to a maximum of 2 weeks before starting treatment). Patients will have 2 additional FDG-PET/CT scans: one after 2 weeks and one after 4 weeks after commencement of treatment.
m	This FDG-PET/CT will be performed at the end of week 2 (day 14 - day 17). At time of FDG injection glycemia and C-peptide will be measured as everolimus can impact on insulinemia/glycemia and therefore indirectly on the SUV of the FDG uptake.
n	This FDG-PET/CT will be performed at the end of week 4 (day 28 - day 31). At time of FDG injection glycemia and C-peptide will be measured as everolimus can impact on insulinemia/glycemia and therefore indirectly on the SUV of the FDG uptake.
o, p	AE's and SAE's will be collected from the signature of informed consent. All non-serious AE's occurring prior to study treatment will be reported in the medical history, unless AE reporting is deemed more appropriate. Adverse events are to be monitored continuously during the study treatment. All AE's occurring during the study and until the end of study treatment/treatment discontinuation visit 28 days after the last dose of study medication are to be recorded; thereafter only drug-related SAE's and AE's/SAE's that qualify for long term reporting should continue to be collected (see section 8.2).
q	Concomitant medication will be recorded in beginning 7 days prior to study enrolment until the end of the treatment period, and thereafter as specified in section 6.8.

r	End of treatment visit is 28 days after the last administration of study drug
s	After end of treatment visit, patients will continue to be followed for survival every 3 months until death from any cause. Survival information can be obtained via phone contact and should be documented in the patient's CRF

Table 2: Main phase: Schedule of assessments

Non-mandatory assessments are shown in []	**Screening**	**Treatment Period**			**End of treatment visit[p]**	**Survival[q]**
**Day**	Prior to enrolment and within 21 days prior first study treatment	**D14**	**D28**	**Every 12 weeks from study treatment start**		
Informed Consent[a]	X					
ER status[b]	X					
Demographic, Medical history[c]	X					
Physical examination, height and weight[d]	X		X	X	X	
ECOG[d]	X		X	X	X	
ECG[e]	X		[X]	[X]	[X]	
Pulmonary function tests (PFTs)[f]	[X]		[X]	[X]	[X]	
Haematology, Coagulation, Biochemistry, Calcium, magnesium, phosphate, liver function tests, LDH, lipid profile, fasting blood glucose level[g,h]	X	X	X	X	X	
Hepatitis B and C serology (HBV-DNA, HBsAg, HBs Ab, HCV-RNA-PCR)[i]	X		[X]	[X]	[X]	
**Response Assessment**						
Radiological examinations: CT or MRI imaging (chest and abdomen) and/or CT/MRI brain[j]	X			X		
FDG-PET/CT[k]	X	X[l]				
**Safety Assessment**						
Adverse events (AE's)[m]	X if applicable	Continuously				
Serious adverse events (SAE's)[n]	X if applicable	Continuously				
Concomitant medications[o]	X if applicable	Continuously				
**Survival**						
Survival status						X

a	Written informed consent must be obtained before any study specific screening assessments are performed.
b	ER status must be provided either on primary tumor pathology report or subsequent biopsies for metastatic disease. Repeat assessment/central testing is NOT required
c	Prior antineoplastic therapy (medication, radiation, surgery) will be collected
d	Physical examination and ECOG performance status are to be measured at baseline and monitored throughout the study.
e	Baseline standard 12 lead ECG and must be performed ≤21 days of the first dose of study medication and may be repeated at the investigators discretion during the study
f	PFTs can be performed at screening or Day 1 (prior to the administration of study drug) and if clinically indicated to exclude or manage non-infectious pneumonitis.
g	Haematology includes counts of haemoglobin, white blood cells, neutrophils, and platelets. Biochemistry includes serum creatinine, urea and electrolytes. Liver function tests include ALP, AST, ALT, total bilirubin. Lipid profile: total serum cholesterol and triglycerides. Patients must be in a fasting state for lipid profile and fasting blood glucose level investigations. At baseline haematology, biochemistry, calcium, magnesium and phosphate, liver function tests, LDH, lipid profile and fasting blood glucose level **should be completed within 7 days of commencement of study treatment**. If haematology/biochemistry was already performed within1 week prior the start of the treatment, these tests do not need to be repeated at Day 1.
h	Haematology, biochemistry, calcium, magnesium, phosphate, liver function tests, LDH, lipid profile, fasting blood glucose level can be completed up to 7 days prior to next cycle of therapy while on study treatment.
i	Screening for hepatitis risk factors and any past illnesses of HBV and HCV is mandatory. See Appendix 1 and 2 for screening, monitoring and management of HBV and HCV
j	Chest, abdomen, pelvis CT's need to be repeated at baseline and every 12 weeks (including if negative at baseline) ± 7 days of scheduled date. Skin lesions should be photographed in addition to measuring. Brain scan (CT scan or MRI with I.V. contrast) at baseline if brain metastases are known and must also be performed if CNS symptoms are present. The imaging technique used for response assessment along the study should be the same method as used at screening.
k	All patients will have FDG-PET/CT at baseline (up to a maximum of 7 days before starting treatment). In the pilot study patients will have 2 additional FDG-PET/CT: one after 2 weeks and one after 4 weeks after commencement of treatment. In the main study, following results from the pilot phase, patients will have only 1 additional FDG-PET/CT after 2 weeks (D14) after the commencement of study treatment.
l	This FDG-PET/CT will be performed at the end of week 2, ideally on day 14 (acceptable timeframe day 13 - day 17). At time of FDG injection glycemia will be measured as everolimus can impact on insulinemia/glycemia and therefore indirectly on the SUV of the FDG uptake.
m, n	AE's and SAE's will be collected from the signature of informed consent. All non-serious AE's occurring prior to study treatment will be reported in the medical history, unless AE reporting is deemed more appropriate. Adverse events are to be monitored continuously during the study treatment. All AE's occurring during the study and until the end of study treatment/treatment discontinuation visit 28 days after the last dose of study medication are to be recorded; thereafter only drug-related SAE's and AE's/SAE's that qualify for long term reporting should continue to be collected (see section 8.2).
o	Concomitant medication will be recorded in beginning 7 days prior to study enrolment until the end of the treatment period, and thereafter as specified in section 6.8.
p	End of treatment visit is 28 days after the last administration of study drug
q	After end of treatment visit, patients will continue to be followed for survival every 3 months until death from any cause. Survival information can be obtained via phone contact and should be documented in the patient's CRF

Table 3: For Pilot and Main phase: Schedule for biological samples for translational research

Non-mandatory assessments are shown in [ ]	**Screening**	**Treatment Period**			**Disease progression**
**Study time point**	Screening	FDG-PET/CT D14	FDG-PET/CT D28 **Only applicable for pilot phase**	CT or MRI assessments	
**Week**		**end of week 2**	**End of week 4**	**visit every 12 weeks**	
**Day**		**14**	**28**		
FFPE primary tumor[a]	X				
Whole blood for isolation of normal DNA[b]	X				
Plasma [c]	X	X	X	X	X
Whole blood for CTC counting[d]	X	[X]	[X]		[X]
Frozen metastatic lesion[e]	X	[X]	X		[X]
FFPE metastatic lesion[e]	X	[X]	X		[X]

a	Mandatory for all patients: one FFPE block from the primary breast tumor.
b	Mandatory for all patients: 7.5ml of blood will be collected in EDTA tubes at screening. This will be later processed for isolation of normal DNA.
c	Mandatory for all patients: 7.5ml of blood will be collected in EDTA tubes:<br>• at screening AND<br>• at the second FDG-PET/CT examination (at the end of week 2 (day 14)) AND<br>• at ALL CT or MRI assessments AND<br>• at disease progression.<br>This will be processed for plasma isolation.
d	Only for the pilot phase: Mandatory and applicable only for patients enrolled at the Institute Jules Bordet in the pilot phase: 7.5ml of blood will be collected in CellSave tubes at screening for detection of CTC's.<br>For patients with ≥5CTCs/7.5ml from the screening sample, additional blood draws of 7.5ml will be collected in CellSave tubes at the second FDG-PET/CT examination (at the end of week 2 (day 14)) AND at disease progression.<br>After the pilot phase, it was decided to not going further with the CTC analysis due to futility.
e	For all patients with accessible lesions: FFPE tissue sample (1 core)/Frozen tissue (2 cores) biopsies of metastatic lesions will be collected:<br>• Mandatory: At baseline, prior to study treatment for patient with accessible lesions<br>• Optional: serial biopsies after D14 FDG-PET/CT and at progression are optional. It is recommended that these biopsies to be collected from accessible **non-metabolic responding** sites (strongly recommended for patients with easily accessible subcutaneous lesion).

6. INVESTIGATIONAL PRODUCT OR STUDY MEDICATION

6.1. Investigational drugs

The investigational drugs used in the course of this trial are everolimus and exemestane. Everolimus and exemestane are taken from the commercial stocks of the hospital pharmacies and will be re-labeled by the pharmacists prior providing them to the patients. For additional information regarding IMPs handling and accountability please refer to procedure manuals.

6.2. Study drug preparation, supply and dispensation

All doses prescribed to the patient and all dose changes during the study must be recorded on the treatment administration page of the CRF.

- Everolimus is formulated as tablets of 5.0 mg and 10 mg strength for oral administration. Everolimus (RAD001) will be purchased locally according to local regulations. Everolimus will be packaged in blister packs. The blisters for everolimus tablets should be opened only at the time of administration as the drug is both hygroscopic and light sensitive. All blisters will conform to local regulatory requirements. Complete guidelines for management and administration of Everolimus can be found either in the package insert or in the SmPC.
- Exemestane is formulated as tablets of 25 mg strength for oral administration. Exemestane will be purchased locally according to local regulations. Complete guidelines for management and administration of exemestane can be found either in the package insert or in the SmPC.

Study treatments will be received by a designated person at the study site, handled and stored safely and properly, and kept in a secured location to which only the investigator and designated assistants have access.

- Everolimus should be stored according to the instructions specified on the commercial package or SmPC.
- Exemestane should be stored according to the information specified on the commercial package insert or SmPC.

Receipt and dispensing of study treatments must be recorded by an authorized person at the investigator's site.

6.2.1. Treatment duration

Patients will be treated with everolimus (RAD001) and exemestane until progression of disease, unacceptable toxicity, death, discontinuation for any other reason.

6.3. Known undesirable effects of study treatment

6.3.1. Everolimus

Patients with a variety of solid and hematologic malignancies have received everolimus as monotherapy and in combination with other agents. The largest completed Phase III trial of everolimus (10 mg) and exemestane (25 mg) was the Bolero-2 trial, which randomised 724 hormone receptor positive patients with endocrine resistance in a 2:1 ratio to receive everolimus (10 mg) and exemestane (25 mg) versus exemestane (10 mg) and placebo. The most common adverse events for combination therapy (>10% incidence) were stomatitis,

rash, fatigue, diarrhea, decreased appetite, nausea, cough, dysgeusia, headache, decreased weight, dyspnea, arthralgia, anemia, epistaxis, vomiting, peripheral edema, pyrexia, AST level increase, constipation, hyperglycemia, pneumonitis, thrombocytopenia, asthenia, ALT level increase, pruritus, insomnia, back pain. The most common grade 3 or 4 toxicities occurring with at least 3% incidence in the combination group were stomatitis, anemia, hyperglycemia, AST and ALT level increase, fatigue, diarrhea, and pneumonitis. For additional information on notable events please refer to the SmPC and for the management of specific toxicities please refer to section 6.4 of the protocol.

6.3.2. Exemestane

The most frequently reported adverse effects for exemestane are gastrointestinal disturbances, hot flushes, arthralgia, myalgia, sweating, fatigue, and dizziness. Other reported effects include headache, insomnia, somnolence, depression, skin rashes, alopecia, asthenia, and peripheral and leg edema. Thrombocytopenia and leucopenia have been reported occasionally. Reductions in bone mineral density can occur with long-term use of exemestane. A total of 1058 patients were treated with exemestane 25 mg once daily in the clinical trials program. Exemestane was generally well tolerated, and adverse events were usually mild to moderate. Adverse events occurring in greater than 10% of patients include hot flushes (14%), nausea (11.9%), insomnia, headache, increased sweating, joint and musculoskeletal pain, and fatigue (USPI; Aromasin SmPC August 2008 (UK as RMS for EU MRP)). Androgenic effects were reported in a limited number of patients (4.3%)[39]. Refer to the package insert of the local supply of exemestane for more details.

6.4. Management of Everolimus specific toxicities

6.4.1. Management of stomatitis/oral mucositis/mouth ulcers

Mouth ulcers, stomatitis and oral mucositis have been seen in patients treated with everolimus. Stomatitis/oral mucositis/mouth ulcers due to everolimus should be treated using local supportive care. Please note that investigators in earlier trials have described the oral toxicities associated with everolimus as mouth ulcers, rather than mucositis or stomatitis. If examination reveals mouth ulcers rather than a more general inflammation of the mouth, please classify the adverse event as such. Please follow the paradigm below for treatment of stomatitis/oral mucositis/mouth ulcers. See table 6-1 for prevention, management and dose adjustments:

1. For mild toxicity (grade 1), use conservative measures such as non-alcoholic mouthwash or salt water (0.9%) mouthwash several times a day until resolution.
2. For more severe toxicity (grade 2 in which case patients have pain but are able to maintain adequate oral alimentation, or grade 3 in which case patients cannot maintain adequate oral alimentation), the suggested treatments are topical analgesic mouth treatments (i.e., local anesthetics such as benzocaine, butyl aminobenzoate, tetracaine hydrochloride, menthol, or phenol) with or without topical corticosteroids, such as triamcinolone oral paste 0.1% (Kenalog in Orabase®).
3. Agents containing hydrogen peroxide, iodine, and thyme derivatives may tend to worsen mouth ulcers. It is preferable to avoid these agents.
4. Antifungal agents must be avoided unless a fungal infection is diagnosed. In particular, systemic imidazole antifungal agents (ketoconazole, fluconazole, itraconazole, etc.) should be avoided in all patients due to their strong inhibition of everolimus metabolism, therefore leading to higher everolimus exposures. Therefore, topical antifungal agents are preferred if an infection is diagnosed. Similarly, antiviral agents such as acyclovir should be avoided unless a viral infection is diagnosed.

6.4.2. Management of dermatological toxicities

Rash and similar dermatologic AEs were common among patients receiving everolimus. The proportion of patients experiencing any rash related AE was 36% in the everolimus and exemestane treatment group in BOLERO-2 [44]. See table 6-1 for prevention, management and dose adjustments.

Table 6-1: Management of dermatological toxicities and dose adjustments

Type of Skin Reaction	Prevention	Action
Oral mucositis/ Stomatitis[67]	• Use salt and baking soda mouthwash<br>• Consume a soft diet	**Any Grade**<br>• Use mucosal-covering agents<br>• Use topical lidocaine solutions<br>• If oral candidiasis present: use oral fluconazole or local clotrimazole troche<br>**Grade 1**<br>Non-alcoholic mouthwash or salt water (0.9%) mouthwash<br>**Grade 2**<br>Topical analgesic mouth wash with or without topical corticosteroids<br>Interrupt everolimus until resolution to ≤ grade 1. Restart at the same dose<br>**Grade 3**<br>Interrupt everolimus until resolution to ≤ grade 1. Restart at dose level -1 (see table 6-3). If stomatitis does not recover to ≤ grade 1 within 4 weeks, discontinue everolimus treatment<br>**Grade 4**<br>Discontinue everolimus treatment
Rash[68]	• Use moisturizer twice daily (alcohol-free)<br>• Avoid abrasive clothing/detergents/shampoo<br>• Avoid direct sunlight<br>• Use SPF 30 lotion/clothing	**Grade 1**<br>Topical hydrocortisone cream 1%<br>**Grade 2**<br>Interrupt everolimus until resolution to ≤ grade 1. Restart at the same dose<br>**Grade 3**<br>Interrupt everolimus until resolution to ≤ grade 1. Restart at dose level -1 (see table 6-3).<br>Oral prednisone<br>**Grade 4**<br>Discontinue everolimus treatment and oral prednisone until ≤ grade 1.

6.4.3. Management of hyperlipidemia and hyperglycaemia

Treatment of hyperlipidemia should take into account the pre-treatment status and dietary habits. Blood tests to monitor hyperlipidemia must be taken in the fasting state. Hyperlipidemia and hypertriglyceridemia should be treated according to local best clinical practice. Patients should be monitored clinically and through serum biochemistry for the development of rhabdomyolysis and other adverse events as required in the product label/data sheets for HMG-CoA reductase inhibitors.

Hyperglycemia has been reported in clinical trials. Monitoring of fasting serum glucose is recommended prior to the start of everolimus therapy and periodically thereafter. Optimal glycemic control should be achieved before starting trial therapy.

6.4.4. Management of Diarrhoea

Appearance of diarrhea attributed to everolimus toxicity may be treated with loperamide. Other medications for diarrhea may be used as needed. These medications will be recorded on the CRF.

6.4.5. Management of non-infectious pneumonitis

Non-infectious pneumonitis is a class effect of rapamycin derivatives. Cases of non-infectious pneumonitis (including interstitial lung disease) have also been described in patients taking everolimus. Some of these have been severe and on rare occasions, a fatal outcome was observed.

A diagnosis of non-infectious pneumonitis should be considered in patients presenting with non-specific respiratory signs and symptoms such as hypoxia, pleural effusion, cough or dyspnea, and in whom infectious, neoplastic and other non-medicinal causes have been excluded by means of appropriate investigations. Patients should be advised to report promptly any new or worsening respiratory symptoms.

Non-infectious pneumonitis is a known side effect of rapamycin analogues. Clinically significant pneumonitis is typically accompanied by non-specific symptoms including dyspnea, nonproductive cough, fatigue, and fever. Diagnosis is generally suspected in individuals receiving mTOR inhibitors who develop these symptoms or in asymptomatic individuals in whom a routine chest CT scan reveals a new ground glass or alveolar infiltrate.

In the BOLERO-2 trial, 12% of patients experienced pneumonitis of any grade with 3% experiencing grade 3 and 0% experiencing grade 4 pneumonitis [44]. An analysis of 4 Novartis-sponsored studies (C2222, Baselga 2009; C2108, Awada 2008; J2101, Hurvitz 2009; J2102 Cardoso 2009) totaling 238 patients did not exceed 3%[66]. In a fifth study involving 49 patients (NCIC CTG, Ellard 2009); the incidence was approximately 10-fold higher. Although such a marked difference remains poorly explained, some considerations can be addressed:

- Different monitoring methods across the studies have been applied: more extensive chest assessment by CT scan in NCIC CTG, J2101, and J2102 studies
- Sponsor management guidelines for non-infectious pneumonitis based on CTCAE grade were developed in 2006, therefore fully implemented only in the J2101 and J2102 studies
- The regular premedication with corticosteroids before the infusion of weekly chemotherapy could in part explain the low incidence observed in J2101 and J2102 studies

The frequency of symptomatic pulmonary toxicity (all grades) was approximately 13% in a phase III study of RAD001 in patients with metastatic renal cell carcinoma. Severe (CTC grade 3) pneumonitis occurred in 4% of patients, and an occasional fatality was reported. The lung toxicity was partly or completely reversible in the majority of cases with interventions including drug interruption, discontinuation and the use of corticosteroids. Individuals participating in this trial will be routinely questioned as to the presence of new or changed pulmonary symptoms consistent with lung toxicity. CT scans and pulmonary function test should be done, as clinically indicated, if there are symptoms that indicate that the patient has developed non-infectious pneumonitis. If non-infectious pneumonitis develops, the guidelines in Table 6-2 should be followed. Dose modification instructions are also provided in Table 6-2 Consultation with a pulmonologist is recommended for any case

Confidential

of pneumonitis that develops during the study.

Table 6-2: Management of non-infectious pneumonitis

Worst Grade Pneumonitis	Required Investigations	Management of Pneumonitis	Everolimus Dose Adjustment
**Grade 1** Asymptomatic, radiographic findings only	CT scans with lung windows. Repeat at least every 12 weeks until return to within normal limits.	No specific therapy is required	Administer 100% of treatment dose.
**Grade 2** Symptomatic not interfering with ADL	CT scans with lung windows. Consider pulmonary function testing includes: spirometry, DLCO, and room air O2 saturation at rest. Repeat at least every three-cycles12 weeks until return to within normal limits. Consider a bronchoscopy with biopsy and / or BAL.	Symptomatic only. Consider corticosteroids if symptoms are troublesome.	Reduce treatment dose by 1 dose level (see Table 6-3) until recovery to ≤ Grade 1. Study treatment may also be interrupted if symptoms are troublesome. Patients will discontinue study treatment if they fail to recover to ≤ Grade 1 within 3 weeks.
**Grade 3** Symptomatic, interfering with ADL; O2 indicated	CT scan with lung windows and pulmonary function testing includes: spirometry, DLCO, and room air O2 saturation at rest. Repeat at least every two to 6 weeks cycles until return to within normal limits. Bronchoscopy with biopsy and / or BAL is	Consider corticosteroids if infective origin is ruled out. Taper as medically indicated.	Hold treatment until recovery to ≤ Grade 1. May restart study treatment within 3 weeks at a reduced dose (by one level, see table 6-3) if evidence of clinical benefit
**Grade 4** Life-threatening; ventilatory support indicated	CT scan with lung windows and required pulmonary function testing, if possible, includes: spirometry, DLCO, and room air O2 saturation at rest. Repeat at least every two-cycles6 weeks until return to within normal limits. Bronchoscopy with biopsy and / or BAL is recommended if possible.	Consider corticosteroids if infective origin is ruled out. Taper as medically indicated.	Discontinue treatment.

6.4.6. Management of infections

Everolimus has immunosuppressive properties and may predispose patients to bacterial, fungal, viral or protozoan infections, including infections with opportunistic pathogens. Localized and systemic infections, including pneumonia, other bacterial infections, invasive fungal infections, such as aspergillosis or candidiasis and viral infections including reactivation of hepatitis B virus (HBV), have been described in patients taking everolimus. Some of these infections have been severe (e.g. leading to respiratory or hepatic failure) and

occasionally have had a fatal outcome.

Physicians and patients should be aware of the increased risk of infection with everolimus. Treat pre-existing infections prior to starting treatment with everolimus. While taking everolimus, be vigilant for symptoms and signs of infection; if a diagnosis of infection is made, institute appropriate treatment promptly and consider interruption or discontinuation of everolimus.

If a diagnosis of invasive systemic fungal infection is made, discontinue everolimus and treat with appropriate antifungal therapy.

Refer to Appendix 1 and 2 for screening, monitoring and management of HBV and HCV.

6.4.7. Management of haematological toxicities

A reduction in blood cell counts is frequent when everolimus therapy is initiated. Without clinical significance and infrequently, anemia and thrombocytopenia have been reported. Patients should have regular hematological monitoring, especially at the beginning of treatment. Suspected drug-related hemorrhages have been exceptional. Nevertheless, everolimus should be considered as predisposing patients to hemorrhage, potentially fatal, should they develop severe drug-related thrombocytopenia. Patients with ongoing thrombocytopenic or with a known bleeding diathesis should be subject to careful evaluation and more frequent monitoring. Please refer to the Investigator Brochure or SmPC for more details.

6.4.8. Management of metabolic events

Elevations of serum creatinine, usually mild, have been reported in clinical trials. Monitoring of renal function, including measurement of blood urea nitrogen (BUN) or serum creatinine, is recommended prior to the start of everolimus therapy and periodically thereafter. Hypercholesterolemia, hypertriglyceridemia, had at least a 2-fold increase in incidence in patients receiving everolimus compared with placebo.

6.5. Follow-up of toxicities

Patients whose treatment is interrupted or permanently discontinued due to an adverse event or abnormal laboratory value must be followed at least once a week for 4 weeks, and subsequently at 4-week intervals, until resolution or stabilization of the event. All patients will be followed for onset of any new serious adverse events for 28 days following the last dose of study drug treatment.

All patients who discontinue study treatment for any reason other than progression will continue to follow the protocol tumor assessments and study visits until progression. After progression, patient will be followed for survival every 3 months.

If, because of toxicity, a patient requires a dose delay of > 4 weeks from the intended day of dose, then the patient must discontinue study treatment. However, the patient will continue to be followed for toxicity, tumor assessments and study visits as previously described.

6.6. Permitted study drug adjustments

For patients who may require dose adjustments because of concomitant medication, please refer to Section 6.10.

For patients who are unable to tolerate the protocol-specified dosing schedule, dose adjustments are permitted (only for everolimus) in order to keep the patient on study drug. No dose adjustment is allowed for exemestane. The following guidelines should be followed.

These changes must be recorded on the CRF.

If treatment is interrupted due to toxicity, study drug should not be resumed until recovery to Grade ≤ 1. Then it could be reintroduced at the initial dose or a lower dose level depending on the toxicity type and grade (see Table 6-1, Table 6-2, Table6-4 and 6-5).

These changes must be recorded on the CRF.

Table 6-3: Study drug dose adjustments for everolimus

Dose level	Dose and Schedule
0=starting dose	10 mg daily
−1 dose level	5 mg
−2 dose level	5 mg every other day

If a patient has already decreased 2 dose levels, no further dose reduction is permitted. Patients requiring an additional dose reduction will be required to discontinue study treatment.

Patients who interrupt therapy for more than 4 weeks must be discontinued from the study.

Remark: If the treatment is interrupted, the investigator should assure that:

- The patient takes a minimum of 10 days of treatment between the Baseline FDG-PET/CT and the D14 FDG-PET/CT.
- The D14 FDG-PET/CT must be performed while the patient is under the study medication since at least 4 days.

6.7. Dosing modifications

Table 6-1, Table 6-2, Table 6-4 and 6-5 provide the procedure to be followed for dose modification of treatment in the event of toxicities suspected to be related to the everolimus. Included are also instructions for re-initiation of study drug once sufficient recovery of toxicity is seen.

Table 6-4: Everolimus dose modification outlines for non-hematologic toxicities

Toxicity	Action
**Dermatological toxicity**	See table 6-1
**Hyperlipidaemia and/or hypertriglyceridemia**	**Any grade:**<br>Treat according to best clinical practice. No specific dose reductions are needed.
**Hyperglycaemia**	**Any grade:**<br>Treat according to best clinical practice. No specific dose reductions are needed
**Pneumonitis**	See table 6-2
**Other toxicities (including dermatological toxicities)**	**Any grade:**<br>Treat according to best clinical practise. Dose reductions can be realized to the discretion of the investigator.
**Toxicity requiring interruption for >4 weeks**	Permanently discontinue treatment

No specific dose adjustments are recommended for Grade 1 toxicity. However, physicians should always manage patients according to their medical judgment based on the particular clinical circumstances.

Table 6-5: Dose modifications for hematologic toxicities

Toxicity	Actions
**Thrombocytopenia platelet count**	≥ 75000/mm^3 (G1)<br>No change<br>50000/ mm^3 to 75000/ mm^3 (G2)<br>Hold everolimus treatment until recovery to ≥ 75000/ mm^3 Reintroduce everolimus at the same dose level<br>< 50000/ mm^3 (G3)<br>Hold everolimus treatment until recovery to ≥ 75000/ mm^3 Reintroduce everolimus at the next lower dose level, if available.
**Absolute Neutrophil count (ANC)**	≥ 1000/ mm^{3}to 1500/ mm^3(G2)<br>No change<br>500/ mm^3 to 1000/ mm^3 (G3)<br>Hold everolimus treatment until recovery to ≥ 1000/ mm3 Reintroduce everolimus at the same dose level<br>< 500/ mm^3 (G4)<br>Hold until recovery to ≥ 1000/ mm^3. Reintroduce everolimus at the next lowest dose level, if available.]
**Febrile Neutropenia**	Hold further dosing until ANC ≥ 1250/ mm^3 and no fever. Then resume dosing at the next lower dose level if available.
**Toxicity requiring interruption for >4 weeks**	Permanently discontinue treatment
Physicians should always manage patients according to their medical judgment based on the particular clinical circumstances.	

6.8. Concomitant Medication and Treatment

All concomitant medications, medical procedures and prior treatments for breast cancer must be reported (within 7 days of study entry) in the CRF, including:

- Date and extent of primary surgery
- Any loco-regional radiation therapy (extent or volume and total dose)
- Any prior hormonal therapy and/or surgical and radiation-induced ovarian ablation and drug induced ovarian suppression (type, drug name, dose and schedule)
- Bisphosphonate therapy (type, drug name, dose and schedule)
- Any additional medication that is necessary for the management of the patient may be used at the discretion of the Investigator but must be reported in the CRF. This includes insulin, oral hypoglycemics, anti-lipid medication
- All concomitant medications are to be reported until the end of study treatment visit (28 days after the last dose of study treatment).
- Refer to section 6.10 for dose modifications of everolimus for concomitant use with CYP3A4/PgP inhibitors and CYP3A4 inducers.

6.9. Allowed therapies

Concomitant treatments are any prescription medications, over-the-counter preparations herbal medications/remedies or radiotherapy used by a patient in the interval beginning 7

days prior to the patient being enrolled into the study and continuing through to 28 days after the discontinuation of study treatment for any reasons. ALL concomitant medications should be reported to the Investigator and reported on the eCRF. In general, all medications taken by the patients for concomitant diseases should continue during the study treatment period and should be recorded on the eCRF.

The following treatments are permitted during the study:

- H1 and H2 antagonists (e.g. diphenhydramine, cimetidine)
- Cardiovascular medications: angiotensin-converting enzyme (ACE)-inhibitors, angiotensin receptor blockers, diuretics, beta-blockers, digoxin, thrombocyte aggregation inhibitors
- Analgesics/anti-inflammatories (e.g. paracetamol/acetaminophen, meperidine, opioids, topical analgesics)
- Corticosteroids under the following conditions:
  - Low dose continuous administration defined as ≤ 1.0mg of dexamethasone or equivalent per day
  - Short term high dose administration defined as ≤20mg of dexamethasone or equivalent per day for ≤7 days
  - Topical applications (e.g. rash)
  - Inhaled spays (e.g. obstructive sleep apnoea)
  - Eye drops or local injections (e.g. intra-articular)
- Medication to treat diarrhoea (e.g. loperamide)
- Oral hypoglycemics and subcutaneous insulin formulations
- Vitamin and mineral supplements
- Bisphosphonate (to be used in accordance with the approved labelled indication and/or nationally recognised treatment guidelines). Indication for bisphosphonate must be recorded.
- Local radiotherapy for analgesic purposes or for lytic lesions at risk of fracture may be carried out if required. No dose modification of study treatment is needed during radiotherapy.

6.10. <u>Excluded therapies</u>

The following therapies are excluded during the study treatment period:

- Investigational or anti-cancer therapies other than those administered in this study, including cytotoxic chemotherapy, radiotherapy, immunotherapy and biological anti-cancer therapy
- Any targeted anti-cancer therapy (e.g.trastuzumab, lapatinib, etc.)
- Corticosteroids under the following conditions:
  - Short-term corticosteroid use>20mg of dexamethasone or equivalent per day for >7 days) or
  - Continuous administration of > 1mg of dexamethasone or equivalent per day
- Any investigational agent, except those within the study
- Haematopoietic growth factors (e.g. erythropoietin's, G-CSF and GM-CSF) are not to be administered prophylactically. Use of these should be reserved to cases of severe neutropenia and anaemia as per the labelling of these agents
- Potent CYP3A4/PgP inhibitors including: Ketoconazole, itraconazole, posaconazole, voriconazole, Telithromycin, clarithromycin, Nefazodone, ritonavir, atazanavir, saquinavir, darunavir, indinavir, nelfinavir
- Moderate CYP3A4/PgP inhibitors including: Erythromycin, verapamil, cyclosporine oral, fluconazole, diltiazem, amprenavir, fosamprenavir, should be used with caution. If a patient requires co-administration of moderate CYP3A4 inhibitors/PgP inhibitors, reduce the dose of everolimus to half the currently used dose. Additional dose

reductions to every other day may be required to manage toxicities. If the inhibitor is discontinued the everolimus dose should be returned to the dose used prior to initiation of the moderate CYP3A4/PgP inhibitor

- Seville orange, star fruit, grapefruit and their juices affect P450 and PgP activity. Concomitant use should be avoided.
- Potent CYP3A4 inducers including: rifampicin, carbamazepine, phenobarbital, phenytoin, efavirenz, nevirapine, and St John's wart. If the patient requires co-administration of strong CYP3A4 inducers an increase in the dose of everolimus up to twice the currently used daily dose should be considered, using 5 mg increments. Enzyme induction usually occurs within 7-10 days; therefore everolimus dose should be increased by one increment 7 days after the start of the inducer therapy. If no safety concerns are seen within the next 7 days the dose can be increased again one additional increment up to a maximum of twice the daily dose used prior to initiation of the strong CYP3A4 inducer.
- This dose adjustment of everolimus is intended to achieve similar AUC to the range observed without inducers. However, there are no clinical data with this dose adjustment in patients receiving strong CYP3A4 inducers. If the strong inducer is discontinued the everolimus dose should be returned to the dose used prior to the initiation of the strong CYP3A4/PgP inducer.
- Live vaccines. Examples include: intranasal influenza, measles, mumps, rubella, oral polio, BCG (bacillus Calmette-Guerin), yellow fever, varicella and TY21a typhoid vaccines

7. ASSESSMENT OF EFFICACY

Assessment of efficacy will occur every 12 weeks and will be made via CT and/or MRI with RECIST 1.1 criteria and PERCIST criteria (refer to section 5 for detailed list of assessments).

8. ASSESSMENT OF SAFETY

8.1. Safety parameters

Safety assessments will consist in monitoring and recording adverse events and serious adverse events; measurement of protocol-specified hematology, clinical chemistry and measurement of protocol-specified vital signs. Progression of disease will not be reported as an adverse event but will be reported on the eCRF.

Safety of the patients participating in this protocol must be reported according to ICH E2A guidance. Serious Adverse Events occurring during this interventional protocol should also be reported according to ICH-E2A guidance, even after the drug is marketed. Serious adverse effect, serious adverse reactions, or suspected unexpected serious adverse reactions (SUSAR) will be reported as per European Clinical Trial directive (2001/20/EC).

8.1.1. Adverse Event

8.1.1.1. Definition

An adverse event (AE) is any untoward medical occurrence in a patient or clinical investigation patient administered a pharmaceutical product and which does not necessarily have a causal relationship with this treatment. An AE can therefore be any unfavourable and

unintended sign (including an abnormal laboratory finding), symptom, or disease temporally associated with the use of a medicinal (investigational) product, whether or not considered related to the medicinal (investigational) product. Pre-existing conditions, which worsen during a study, are to be reported as AEs.

8.1.1.2. AE Intensity

Intensity of all AEs will be graded according to the NCI-CTCAE version 4.0 on a five-point scale (Grade 1 to 5) and reported in detail on the eCRF. Adverse events not listed in CTCAE should be graded as listed in Table 8-1.

Table 8-1: Grading for Adverse Events Not Listed in NCI-CTCAE

CTC Grade	Equivalent To:	Definition
Grade 1	Mild	Asymptomatic or mild symptoms; clinical or diagnostic observations only; intervention not indicated
Grade 2	Moderate	Minimal, local or non-invasive intervention indicated; limiting age-appropriate instrumental activities of daily living (ADL)*
Grade 3	Severe	Severe or medically significant but not immediately life-threatening; hospitalization or prolongation of hospitalization indicated; disabling; limiting self-care ADL**
Grade 4	Life-threatening/disabling	Life-threatening consequences; urgent intervention required
Grade 5	Death	Death related to AE

*Instrumental ADL refer to preparing meals, shopping for groceries or clothes, using the telephone, managing money etc.

**Self-care ADL refer to bathing, dressing and undressing, feeding self, using the toilet, taking medications, and not bedridden.

8.1.1.3. Relationship to study medication

The causality relationship of the AE to the investigational study medication will be assessed by the Investigator as either:

Yes or No

If there is a reasonable suspected causal relationship to the exemestane or everolimus, i.e., there are facts (evidence) or arguments to suggest a causal relationship, drug-event relationship should be assessed as **Yes**.

The following criteria should be considered in order to assess the relationship as **Yes:**

- Reasonable temporal association with drug administration.
- It may or may not have been produced by the patient’s clinical state, environmental or toxic factors, or other modes of therapy administered to the patient.
- Known response pattern to suspected drug.
- Disappears or decreases on cessation or reduction in dose.
- Reappears on re-challenge.

The following criteria should be considered in order to assess the relationship as No:

- It does not follow a reasonable temporal sequence from administration of the drug.
- It may readily have been produced by the patient’s clinical state, environmental or toxic factors, or other modes of therapy administered to the patient.

- It does not follow a known pattern of response to the suspected drug.
- It does not reappear or worsen when the drug is re-administered.

8.1.2. Serious Adverse Event (SAE)

A serious adverse event is any experience that suggests a significant hazard, contraindication, side effect or precaution. It is any adverse event that at any dose:

- Is fatal (results in death; NOTE: death is an outcome, not an event);
- Is life-threatening (NOTE: the term "Life-threatening" refers to an event in which the patient was at immediate risk of death at the time of the event; it does not refer to an event which could hypothetically have caused death had it been more severe)
- Requires in-patient hospitalisation or prolongation of existing hospitalisation; unless hospitalisation is for:
    - o Routine treatment or monitoring of the studied indication, not associated with any deterioration in condition
    - o Elective or pre-planned treatment for a pre-existing condition that is unrelated to the indication under study and has not worsened since signing the informed consent
    - o Treatment on an emergency outpatient basis for an event not fulfilling any of the definitions of a SAE given above and not resulting in hospital admission
    - o Social reasons and respite care in the absence of any deterioration in the patient's general condition
- Results in persistent or significant disability/incapacity;
- Is a congenital anomaly/birth defect;
- Is medically significant or requires intervention to prevent one or other of the outcomes listed above.

In this study, the expectedness assessment will be performed by the sponsor by using the following reference documents:

- Everolimus : summary of product characteristics
- Exemestane: summary of product characteristics

The study will comply with all local regulatory requirements. The full requirements of the ICH Note for guidance on Clinical Safety Data Management, Definitions and Standards for Expedited Reporting, Topic E2A, will be adhered to.

8.1.3. Disease Progression

Progression of underlying malignancy is not reported as an AE if it is clearly consistent with the suspected progression of the underlying cancer. Hospitalization due solely to the progression of underlying malignancy should NOT be reported as an SAE/AE. Clinical symptoms of progression may be reported as AEs or SAEs if the symptom cannot be determined as exclusively due to the progression of the underlying malignancy, or does not fit the expected pattern of progression for the disease under study.

If there is any uncertainty about an AE being due to the disease under study, it should be reported as an AE or SAE. Clinical symptoms of underlying malignancy, even if they meet a seriousness criteria, should not be reported as SAE unless the investigator considers them as more severe than expected.

Secondary primary malignancies are to be reported as SAEs at any time regardless of the time elapsed since the last dose of investigational product or study medication.

8.2. Handling of Safety Parameters

8.2.1. Reporting of Adverse Events

During the period after signing the informed consent and study day 1 (administration of study medication), any non-serious AEs that occur will be reported in the medical history, unless AE reporting is deemed more appropriate; SAEs caused by a protocol mandated intervention will be collected (e.g., SAEs related to invasive procedures such as biopsies, medication washout, or no treatment run-in).

All adverse events (related and unrelated) occurring during the study conduct and **up to 28 days after the last dose of study medication** must be reported on the eCRF. The occurrence of adverse events should be sought by non-directive questioning of the patient (subject) during the screening process after signing informed consent and at each visit during the study. Adverse events also may be detected when they are volunteered by the patient (subject) during the screening process or between visits, or through physical examination, laboratory test, or other assessments.

All adverse events should be treated appropriately. If a concomitant medication or non-drug therapy is given; this action should be recorded on the eCRF.

Once an adverse event is detected, it should be followed until its resolution or until it is judged to be permanent, and assessment should be made at each visit (or more frequently, if necessary) of any changes in severity, the suspected relationship to the study treatment, the interventions required to treat it, and the outcome.

8.2.2. Reporting of Serious Adverse Events

Any clinical AE or abnormal laboratory test value that is *serious* and which occurs during the course of the study must be reported to the Sponsor (Institut Jules Bordet) immediately and no later than *24 hours* of the Investigator becoming aware of the event.

Information about all SAEs is collected and recorded on the Serious Adverse Event Report Form. The investigator must assess and record the relationship of each SAE to study treatment, complete the SAE Report Form in English, and send the completed, signed form to the Sponsor (IJB) by using the fax number or email address mentioned on the form.

The original copy of the SAE Report Form and the fax confirmation sheet or the e-mail if SAE sent as scanned copy must be kept with the case report form documentation at the study site.

Follow-up information is sent to the same contact(s) to which the original SAE Report Form was sent, using a new SAE Report Form stating that this is a follow-up to the previously reported SAE and giving the date of the original report or using the query form sent by the Sponsor. Each re-occurrence, complication, or progression of the original event should be reported as a follow-up to that event regardless of when it occurs. The follow-up information should describe whether the event has resolved or continues, if and how it was treated, and whether the patient continued or withdrew from study participation.

Recurrent episodes, complications, or progression of the initial SAE must be reported as follow-up to the original episode within 24 hours of the investigator receiving the follow-up information. An SAE occurring at a different time interval or otherwise considered completely unrelated to a previously reported one should be reported separately as a new event.

For all serious adverse events, the Sponsor will pursue and provide information to Novartis within 24 working hours after reception. In addition, the Sponsor may be requested by Novartis to obtain specific additional follow-up information from investigator in a diligent fashion. This information may be more detailed than that captured on the adverse event

case report form. In general, this will include a description of the adverse event in sufficient detail to allow for a complete medical assessment of the case and independent determination of possible causality. Information on other possible causes of the event, such as concomitant medications and illnesses must be provided. In the case of a patient death, a summary of available autopsy findings must be submitted as soon as possible, if conducted, to the study coordinator or its designated representative.

Related SAEs must be collected and reported regardless of the time elapsed from the last study medication administration, even if the study has been closed.

Unrelated SAEs must be collected and reported during the study and for up to 28 days after the last dose of study medication.

SAEs occurring during screening, after ICF signature, will also be reported.

It is the responsibilities of the sponsor to report SUSARs to EudraVigilance Clinical Trial Module (EVCTM), to Competent Authorities, Central Ethics Committees and to participating investigators.

The sponsor will submit the Development Safety Update Report to Competent Authorities, and Central Ethics Committees.

The reporting of SAEs and SUSARs may be subject to change to comply with updated regulations and/or study procedures.

8.2.3. Reporting of deaths

A death occurring during treatment with study medication or within 28 days after stopping treatment, whether considered to be treatment-related or not, must be reported.

- All deaths thought to be related to study drug at any time should be reported as a SAE regardless of the time elapsed since the last dose of investigational product.
- Deaths related to progression of the underlying disease during the course of the study will not be reported as a SAE, but should be reported on the appropriate eCRF page (unless the patient has withdrawn consent).
- Deaths from other causes should be reported on the appropriate eCRF page.

9. STATISTICAL CONSIDERATIONS AND ANALYTICAL PLAN

9.1. Statistical Model

9.1.1. Analysis of Primary Variable

The primary endpoint is PFS defined as the time elapsed between the FDG-PET/CT at D14 and progression, second primary or death, whichever occurs the first. Progression will be documented on the basis of traditional RECIST 1.1 criteria. The primary goal of the analysis will be to compare PFS distributions between early FDG-PET metabolic responders and non-responders. **The primary method for defining response is the dominance method**. The final analysis will take place using a Cox regression model after approximately 42 PFS events have occurred, assuming the 59% metabolic non-response rate. A data cut-off date will be determined when this number of events occurs, and the clinical data on or prior to the data cut-off date will be thoroughly cleaned. First a univariate analysis will be performed followed by a multivariate analysis, adjusting for up to 3 known prognostic variables in breast cancer. The variables for adjustment will be decided before carrying out any analysis. Secondary analyses will include Cox regression analyses for OS.

9.2. Sample Size

We assume that the FDG-PET responders have a similar PFS to the patients treated with exemestane plus everolimus in the BOLERO-2 trial (10.6 months) and that non FDG-PET responders will have a similar PFS as patients treated with exemestane alone in the BOLERO-2 trial (4.1 months). Under exponential survival this translates to a hazard ratio of 0.36. According to the pilot phase (N= 27 evaluable patients) non-FDG-PET responders represent 59% (instead of the assumed 20% at the initial design) of the population and that their 6 months PFS is given by 37% (estimated from BOLERO-2 survival curves, control group).

In order to show an increase in 6-months PFS from 37% for non-FDG-PET responders to 70% for FDG-PET responders (corresponding to a hazard ratio of 0.36) with 90% power at a two-sided significance level 0.05 using a logrank test, and with the pilot phase's result, a total of 42 PFS events are required in the analysis (instead of the 62 PFS event calculated in the initial design). At an accrual rate of 1-2 evaluable patient per month, 46 evaluable patients are needed to answer the primary endpoint of the trial. This means 19 (=46-27(pilot)) more evaluable patients have to be recruited during the main phase. If we consider the screen failure rate observed in the pilot phase, about 30 patients need to be screened to complete the study.

A Cox regression model will be applied to assess the effect of FDG-PET metabolic response rate on PFS and OS. Multivariate analyses will be performed, adjusting for maximum three factors.

10. DIRECT ACCESS TO SOURCE DATA/DOCUMENTS

Each participating site will maintain appropriate medical and research records for this trial, in compliance with Section 4.9 of the ICH E6 GCP, and regulatory and institutional requirements for the protection of confidentiality of subjects. As part of participating in the trial, each site will permit authorised representatives of the sponsor(s) and regulatory agencies to examine (and when required by applicable law, to copy) clinical records for the purposes of quality assurance reviews, audits or inspections.

Source data are all information, original records of clinical findings, observations, or other activities in a clinical trial necessary for the reconstruction and evaluation of the trial. Examples of these original documents and data records include, but are not limited to, hospital records, clinical and office charts, laboratory notes, memoranda, subjects' diaries or evaluation checklists, pharmacy dispensing records, recorded data from automated instruments, copies or transcriptions certified after verification as being accurate and complete, microfiches, photographic negatives, microfilm or magnetic media, x-rays, and subject files and records kept at the pharmacy, at the laboratories, and medico-technical departments involved in the clinical trial.

Data collection is the responsibility of the clinical trial staff at the site under the supervision of the site Principal Investigator. The study case report form (eCRF) is the primary data collection instrument for the study. The investigator should ensure the accuracy, completeness, legibility, and timeliness of the data reported in the eCRFs and all other required reports. Data reported on the eCRF, that are derived from source documents, should be consistent with the source documents or the discrepancies should be explained. All data requested on the eCRF must be recorded. Any missing data must be explained.

The investigator/institution should maintain the trial documents as specified in Essential Documents for the Conduct of a Clinical Trial (ICH E6 Section 8) and as required by applicable regulations and/or guidelines. The investigator/institution should take measures to prevent accidental or premature destruction of these documents.

Essential documents (written and electronic) should be retained for a period of not less than twenty (20) years from the completion of the clinical trial unless the sponsor provides written permission to dispose of them or, requires their retention for an additional period of time because of applicable laws, regulations and/or guidelines.

10.1. Confidentiality of study documents and patient records

The investigator must ensure anonymity of the patients; patients must not be identifiable by names in any documents submitted. Signed informed consent forms and patient enrolment log must be kept strictly confidential to enable patient identification at the site.

11. QUALITY CONTROL

11.1. Quality control: study monitoring

In accordance with applicable regulations, GCP and any contracts governing the study, a representative from IJB will contact the site prior to the start of the study to review with the site and staff the protocol, study requirements, and their responsibilities to satisfy regulatory, ethical, and IJB requirements.

The appointed Clinical Research Administrator/Monitor will monitor the study consistent with the demands of the study and site activity to verify that the:

Data are authentic, accurate, and complete;

Study is conducted in accordance with the currently approved protocol, study contracts, GCP, and all applicable regulatory requirements.

Throughout the study, the joint study management team will review data according to the Data Management Plan.

11.2. Quality control: on-site monitoring

Accurate, complete and reliable data collection will be assured by verification and cross–check of the eCRFs against the Investigator's records by the study monitor (source document verification), and the maintenance of a drug-dispensing log by the Investigator.

The study monitor verifies that safety and rights of subjects are protected.

The investigator and the head of the medical institution (where applicable) agrees to allow the monitor direct access to all relevant documents.

11.3. Quality control

The overall procedures for quality assurance of clinical study data are described in the Sponsor SOPs and SOPs of the BREAST Data Centre of the Institute Jules Bordet.

11.4. Regulatory agencies Inspections

Regulatory Agencies may also conduct a regulatory inspection of the study.

Such audits/inspections can occur at any time during or after completion of the study. If an audit or inspection occurs, the investigator and institution agree to allow the auditor/inspector direct access to all relevant documents and to allocate his/her time and the time of his/her staff to the auditor/inspector to discuss findings and any relevant issues.

In case of Inspection from Regulatory Agencies is planned, the Investigator should promptly notify Jules Bordet Institute, the Sponsor.

12. REGULATORY AND ETHICAL CONSIDERATIONS

12.1. Regulatory considerations

The study is submitted by the principal investigator(s) or the national coordinator(s) or the sponsor (or its legal representative), in accordance with local regulations, to and approved by an appropriate Independent Ethical Review Committee (IEC)/ Institutional Review Board (IRB) and a Regulatory Authority if required by the national laws of the countries where the study is conducted. Local regulatory approval may also be required.

The study cannot start in a participating site before written approval by corresponding Ethics Committee(s) has been obtained and the local regulatory requirements have been complied with.

The sponsor provides a copy of the final protocol, subject information sheets, consent forms and all other relevant study documentation for local required submissions.

The principal investigator and the sponsor ensure that the study is conducted in full conformance with the principles of the “Declaration of Helsinki” 1964, as revised from time to time and with the laws and regulations of the country in which the research is conducted, whichever affords the greater protection to the individual. The study must fully adhere to the principles outlined in “Guideline for Good Clinical Practice” ICH-E6 Tripartite Guideline (January 1997) and with national laws.

For the Study conducted in the EU/EEA countries, the principal investigator ensures compliance with the EU Clinical Trial Directive (2001/20/EC)/ICH GCP and with the EU Data Protection Directive (95/46/EC).

In other countries where “Guidelines for Good Clinical Practice” exist, the sponsor and the principal investigators strictly ensure adherence to the stated provisions.

12.2. Informed Consent Process

12.2.1. General

Informed consent is a process initiated prior to an individual agreeing to participate in a trial and continues throughout the individual’s participation. In obtaining and documenting informed consent, the investigator should comply with applicable regulatory requirements and should adhere to GCP and to the ethical principles that have their origin in the Declaration of Helsinki.

Potential participants will generally be identified during consultation and cases discussed during the Multi-Disciplinary Team (MTD) meetings. The investigator will provide potential participants with written information about the trial; including contact details of the research team should they require further information, and arrange for an outpatient clinic appointment in the usual way.

Patient Information and Consent forms (PIC), approved by the central ethical committee (CEC), will be provided by the investigator. The PIC will describe in detail the trial procedures the interventions/products, and potential risks/benefits. All patients will receive

the appropriate version of the written information and be asked to read and review it. The PIC will emphasise that participation in the trial is voluntary and that the patient may, without being subject to any resulting detriment, withdraw from the trial at any time by revoking the informed consent. The rights and welfare of the patients will be protected by emphasising to them that the quality of medical care will not be adversely affected if they decline to participate in this study. All patients will be given the opportunity to ask questions and will be given sufficient time to consider trial entry before consenting.

The consent form will request permission for the patient's General Practitioner to be informed of their involvement in the trial and also permission for personnel involved in the research or from regulatory authorities to have access to the individual's medical records.

For patients not qualified to give or incapable of giving legal consent, written consent must be obtained from the legal representative. In the case where both the patient and his or her legal representative are unable to read, an impartial witness should be present during the entire informed consent discussion.

12.2.2. Process of Informed Consent

The consent process will be carried out by a member of the research team identified in the trial signature and delegation log. Discussion of objectives and potential inconveniences of the intervention period, and the conditions under which it is to be conducted, are to be provided to patients by staff with appropriate experience. Patients consent will be obtained **prior** to any study related procedure being carried out.

Both the researcher taking consent and the patient must personally sign and date the form.

The original copy of the signed consent form will be retained in the patient's medical notes and must be made available for monitoring, audit or inspection. A copy will be put in the investigator site file. A further copy of the signed consent/assent forms will be given to the patient.

If new safety information results in significant changes in the risk/benefit assessment, or if the protocol undergoes substantial changes and an amendment is released, the consent form should be reviewed and updated if necessary. All patients, including those already being treated, should be informed of the new information, given a copy of the revised form, and give their consent to continue in the study.

13. DATA HANDLING AND RECORD KEEPING

13.1. Investigator's file

The investigator must maintain adequate and accurate records to enable the conduct of the study to be fully documented and the study data to be subsequently verified. These documents should be classified into two different separate categories: (1) Investigator's study file; and (2) patient clinical source documents.

The investigator's study file should be established according to the ICH-GCP E6 and should include a delegation log established by the investigator.

Subject clinical source documents would include:
- subject hospital/clinic records;
- physician's and nurse's notes;
- appointment book;

Confidential

- original laboratory reports;
- signed informed consent forms;
- consultant letters;
- the screening and enrolment logs.

13.2. Case report form (CRF)

For each subject enrolled, a CRF must be completed and signed by the principal investigator or authorized delegate from the study staff. This also applies to records for those subjects who fail to complete the study. If a subject withdraws from the study, the reason must be noted on the CRF. The investigator should ensure the accuracy, completeness, legibility and timeliness of the data reported in the CRFs and in all required reports.

Before the start of the study, the list of staff members authorized to signed case report (with a sample of their signature) must be completed and retained in the investigator's file.

13.3. Retention of documents

Following closure of the study, the investigator or the head of the medical institution (where applicable) must maintain all site study records, except for those required by local regulations to be maintained by someone else, in a safe and secure location. The records must be maintained to allow easy and timely retrieval, when needed (e.g., audit or inspection), and, whenever feasible, to allow any subsequent review of data in conjunction with assessment of the facility, supporting systems, and staff. Where permitted by local laws/regulations or institutional policy, some or all of these records can be maintained in a format other than hard copy (e.g., microfiche, scanned, electronic); however, caution needs to be exercised before such action is taken. The investigator must assure that all reproductions are legible and are a true and accurate copy of the original and meet accessibility and retrieval standards, including re-generating a hard copy, if required. Furthermore, the investigator must ensure there is an acceptable back-up of these reproductions and that an acceptable quality control process exists for making these reproductions.

IJB will inform the investigator of the time period for retaining these records to comply with all applicable regulatory requirements. The minimum retention time will meet the strictest standard applicable to that site for the study, as dictated by any institutional requirements or local laws or regulations; otherwise, the retention period will default to 20 years after the completion of the study and/or 2 years after approval by relevant Health Authorities, whichever is longer.

Should the Investigator wish to assign the study records to another party or move them to another location, the Sponsor must be notified in advance. If the Investigator cannot guarantee this archiving requirement at the investigational site for any or all of the documents, special arrangements must be made between the Investigator and the Sponsor to store these in a sealed container(s) outside of the site so that they can be returned sealed to the Investigator, in case of a regulatory audit. Where source documents are required for the continued care of the patient; appropriate copies should be made before storing outside of the site.

14. PUBLICATION POLICY

All participating centres will be entitled to authorship on full manuscripts provided they contribute at least six fully evaluable patients.

The designers/coordinators of the trial – e.g. the medical oncology and nuclear medicine departments of Institute Jules Bordet – will share first and last authorship.

15. FINANCING AND INSURANCE

The sponsor has taken out a liability insurance policy to cover its liability as required by applicable law and especially in accordance with the Belgian Law relating to experiments in human dated May 7, 2004, its subsequent amendments and Royal Decree of execution. Upon request, the sponsor will provide the Investigator with a certificate of insurance. The insurance of the sponsor does not relieve the Institution and the Investigator of any obligation to maintain their liability insurance policy. Clinical trial insurance is only valid in centres authorized from the coordinating institution.

For details refer to site study agreements for detailed information.

16. SCIENTIFIC REFERENCES

1. World Health Organization, GLOBCAN 2008: Cancer incidence, mortality and prevalence worldwide in 2008

2. Boyle P, Ferlay J, Cancer incidence and mortality in Europe, 2004, Annals of oncology : official journal of the European Society for Medical Oncology / ESMO 2005;16:481–8.

3. Smith IE, Dowsett M, Aromatase inhibitors in breast cancer, The New England journal of medicine 2003;348:2431–42.

4. Jensen EV, Jordan VC, The estrogen receptor: a model for molecular medicine, Clinical cancer research : an official journal of the American Association for Cancer Research 2003;9:1980–9.

5. O'Shaughnessy J, Extending survival with chemotherapy in metastatic breast cancer, The oncologist 2005;10 Suppl 3:20–9.

6. Mouridsen H, Gershanovich M, Sun Y, Perez-Carrion R, Boni C, Monnier A, et al, Phase III study of letrozole versus tamoxifen as first-line therapy of advanced breast cancer in postmenopausal women: analysis of survival and update of efficacy from the International Letrozole Breast Cancer Group, Journal of clinical oncology : official journal of the American Society of Clinical Oncology 2003;21:2101–9.

7. Trialists' EBCTCG, Tamoxifen for early breast cancer: an overview of the randomised trials. Early Breast Cancer Trialists' Collaborative Group, Lancet 1998;351:1451–67.

8. Forbes JF, Cuzick J, Buzdar A, Howell A, Tobias JS, Baum M, Effect of anastrozole and tamoxifen as adjuvant treatment for early-stage breast cancer: 100-month analysis of the ATAC trial, The lancet oncology 2008;9:45–53.

9. Jakesz R, Jonat W, Gnant M, Mittlboeck M, Greil R, Tausch C, et al, Switching of postmenopausal women with endocrine-responsive early breast cancer to anastrozole after 2 years' adjuvant tamoxifen: combined results of ABCSG trial 8 and ARNO 95 trial, Lancet366:455–62.

10. Bonneterre J, Thürlimann B, Robertson JF, Krzakowski M, Mauriac L, Koralewski P, et al, Anastrozole versus tamoxifen as first-line therapy for advanced breast cancer in 668 postmenopausal women: results of the Tamoxifen or Arimidex Randomized Group Efficacy and Tolerability study, Journal of clinical oncology : official journal of the American Society of Clinical Oncology 2000;18:3748–57.

11. Nabholtz JM, Buzdar A, Pollak M, Harwin W, Burton G, Mangalik A, et al, Anastrozole is superior to tamoxifen as first-line therapy for advanced breast cancer in postmenopausal women: results of a North American multicenter randomized trial. Arimidex Study Group, Journal of clinical oncology : official journal of the American Society of Clinical Oncology 2000;18:3758–67.

12. Buzdar AU, Jonat W, Howell A, Jones SE, Blomqvist CP, Vogel CL, et al, Anastrozole versus megestrol acetate in the treatment of postmenopausal women with advanced breast carcinoma: results of a survival update based on a combined analysis of data from two mature phase III trials, Arimidex Study Group, Cancer 1998;83:1142–52.

13. Thürlimann B, Keshaviah A, Coates AS, Mouridsen H, Mauriac L, Forbes JF, et al, A comparison of letrozole and tamoxifen in postmenopausal women with early breast cancer, The New England journal of medicine 2005;353:2747–57.

14. Goss PE, Ingle JN, Martino S, Robert NJ, Muss HB, Piccart MJ, et al, A randomized trial of letrozole in postmenopausal women after five years of tamoxifen therapy for early-stage breast cancer, The New England journal of medicine 2003;349:1793–802.

15. Dombernowsky P, Smith I, Falkson G, Leonard R, Panasci L, Bellmunt J, et al, Letrozole, a new oral aromatase inhibitor for advanced breast cancer: double-blind randomized trial showing a dose effect and improved efficacy and tolerability compared with megestrol acetate, Journal of clinical oncology : official journal of the American Society of Clinical Oncology 1998;16:453–61.

16. Coombes RC, Kilburn LS, Snowdon CF, Paridaens R, Coleman RE, Jones SE, et al, Survival and safety of exemestane versus tamoxifen after 2-3 years' tamoxifen treatment (Intergroup Exemestane Study): a randomised controlled trial, Lancet 2007;369:559–70.

17. Kaufmann M, Bajetta E, Dirix LY, Fein LE, Jones SE, Zilembo N, et al, Exemestane is superior to megestrol acetate after tamoxifen failure in postmenopausal women with advanced breast cancer: results of a phase III randomized double-blind trial, The Exemestane Study Group. [Internet]. . Journal of clinical oncology : official journal of the American Society of Clinical Oncology 2000;18:1399–411.

18. Osborne CK, Pippen J, Jones SE, Parker LM, Ellis M, Come S, et al, Double-blind, randomized trial comparing the efficacy and tolerability of fulvestrant versus anastrozole in postmenopausal women with advanced breast cancer progressing on prior endocrine therapy: results of a North American trial, Journal of clinical oncology : official journal of the American Society of Clinical Oncology 2002;20:3386–95.

19. Ingle JN, Suman VJ, Rowland KM, Mirchandani D, Bernath AM, Camoriano JK, et al, Fulvestrant in women with advanced breast cancer after progression on prior aromatase inhibitor therapy: North Central Cancer Treatment Group Trial N0032, Journal of clinical oncology : official journal of the American Society of Clinical Oncology 2006;24:1052–6.

20. Osborne CK, Tamoxifen in the treatment of breast cancer, The New England journal of medicine 1998;339:1609–18.

21. Schiff R, Massarweh SA, Shou J, Bharwani L, Mohsin SK, Osborne CK, Cross-talk between estrogen receptor and growth factor pathways as a molecular target for overcoming endocrine resistance, Clinical cancer research : an official journal of the American Association for Cancer Research 2004;10:331S–6S.

22. Campbell RA, Bhat-Nakshatri P, Patel NM, Constantinidou D, Ali S, Nakshatri H, Phosphatidylinositol 3-kinase/AKT-mediated activation of estrogen receptor alpha: a new model for anti-estrogen resistance, The Journal of biological chemistry 2001;276:9817–24.

23. Santen RJ, Song RX, Zhang Z, Kumar R, Jeng M-H, Masamura S, et al, Adaptive hypersensitivity to estrogen: mechanisms and clinical relevance to aromatase inhibitor therapy in breast cancer treatment, The Journal of steroid biochemistry and molecular biology 2005;95:155–65.

24. Tokunaga E, Kimura Y, Oki E, Ueda N, Futatsugi M, Mashino K, et al, Akt is frequently activated in HER2/neu-positive breast cancers and associated with poor prognosis among hormone-treated patients, International journal of cancer Journal international du cancer 2006;118:284–9.

25. Yue W, Fan P, Wang J, Li Y, Santen RJ, Mechanisms of acquired resistance to endocrine therapy in hormone-dependent breast cancer cells, The Journal of steroid biochemistry and molecular biology 2007;106:102–10.

26. Yamnik RL, Digilova A, Davis DC, Brodt ZN, Murphy CJ, Holz MK, S6 kinase 1 regulates estrogen receptor alpha in control of breast cancer cell proliferation, The Journal of biological chemistry 2009;284:6361–9.

27. Rudolf J, Boulay A, Zumstein-Mecker S, Evans DB, O'Reily T LH, The mTOR pathway in estrogen response: a potential for combining the rapamycin derivative RAD001 with the aromatase inhibitor letrozole in breast carcinomas, Proc Am Assoc Cancer Res 2004;45:Abstract A5619.

28. deGraffenried LA, Friedrichs WE, Russell DH, Donzis EJ, Middleton AK, Silva JM, et al, Inhibition of mTOR activity restores tamoxifen response in breast cancer cells with aberrant Akt Activity, Clinical cancer research : an official journal of the American Association for Cancer Research 2004;10:8059–67.

29. Novartis Oncology: Affinitor, Investigator Brochure 2012;

30. Bjornsti M-A, Houghton PJ, The TOR pathway: a target for cancer therapy, Nature reviews Cancer 2004;4:335–48.

31. Abraham RT, Gibbons JJ, The mammalian target of rapamycin signalling pathway: twists and turns in the road to cancer therapy, Clinical cancer research : an official journal of the American Association for Cancer Research 2007;13:3109–14.

32. Sarbassov DD, Ali SM, Kim D-H, Guertin DA, Latek RR, Erdjument-Bromage H, et al, Rictor, a novel binding partner of mTOR, defines a rapamycin-insensitive and raptor-

independent pathway that regulates the cytoskeleton, Current biology : CB 2004;14:1296–302.

33. Guba M, von Breitenbuch P, Steinbauer M, Koehl G, Flegel S, Hornung M, et al, Rapamycin inhibits primary and metastatic tumor growth by antiangiogenesis: involvement of vascular endothelial growth factor, Nature medicine 2002;8:128–35.

34. Tsutsumi N, Yonemitsu Y, Shikada Y, Onimaru M, Tanii M, Okano S, et al, Essential role of PDGFRalpha-p70S6K signaling in mesenchymal cells during therapeutic and tumor angiogenesis in vivo: role of PDGFRalpha during angiogenesis, Circulation research 2004;94:1186–94.

35. Mabuchi S, Altomare DA, Connolly DC, Klein-Szanto A, Litwin S, Hoelzle MK, et al, RAD001 (Everolimus) delays tumor onset and progression in a transgenic mouse model of ovarian cancer, Cancer research 2007;67:2408–13.

36. Lane HA, Wood JM, McSheehy PMJ, Allegrini PR, Boulay A, Brueggen J, et al, mTOR inhibitor RAD001 (everolimus) has antiangiogenic/vascular properties distinct from a VEGFR tyrosine kinase inhibitor, Clinical cancer research : an official journal of the American Association for Cancer Research 2009;15:1612–22.

37. Yu Y, Sato JD, MAP kinases, phosphatidylinositol 3-kinase, and p70 S6 kinase mediate the mitogenic response of human endothelial cells to vascular endothelial growth factor, Journal of cellular physiology 1999;178:235–46.

38. Viñals F, Chambard JC, Pouysségur J, p70 S6 kinase-mediated protein synthesis is a critical step for vascular endothelial cell proliferation, The Journal of biological chemistry 1999;274:26776–82.

39. Buzdar A, Pharmacology and pharmacokinetics of the newer generation aromatase inhibitors, Clinical cancer research : an official journal of the American Association for Cancer Research 2003;9:468S–72S.

40. Demers LM, Lipton A, Harvey HA, Kambic KB, Grossberg H, Brady C, et al, The efficacy of CGS 20267 in suppressing estrogen biosynthesis in patients with advanced stage breast cancer, The Journal of steroid biochemistry and molecular biology 1993;44:687–91.

41. Hutson PR, Love RR, Havighurst TC, Rogers E, Cleary JF, Effect of exemestane on tamoxifen pharmacokinetics in postmenopausal women treated for breast cancer, Clinical cancer research : an official journal of the American Association for Cancer Research 2005;11:8722–7.

42. Motzer RJ, Escudier B, Oudard S, Hutson TE, Porta C, Bracarda S, et al, Efficacy of everolimus in advanced renal cell carcinoma: a double-blind, randomised, placebo-controlled phase III trial, Lancet 2008;372:449–56.

43. Yao JC, Shah MH, Ito T, Bohas CL, Wolin EM, Van Cutsem E, et al, Everolimus for advanced pancreatic neuroendocrine tumors, The New England journal of medicine 2011;364:514–23.

44. Baselga J, Campone M, Piccart M, Burris HA, Rugo HS, Sahmoud T, et al, Everolimus in postmenopausal hormone-receptor-positive advanced breast cancer, The New England journal of medicine 2012;366:520–9.

45. Awada A, Cardoso F, Fontaine C, Dirix L, De Grève J, Sotiriou C, et al, The oral mTOR inhibitor RAD001 (everolimus) in combination with letrozole in patients with advanced breast cancer: results of a phase I study with pharmacokinetics, European journal of cancer (Oxford, England : 1990) 2008;44:84–91.

46. Baselga J, Semiglazov V, van Dam P, Manikhas A, Bellet M, Mayordomo J, et al, Phase II randomized study of neoadjuvant everolimus plus letrozole compared with placebo plus letrozole in patients with estrogen receptor-positive breast cancer, Journal of clinical oncology : official journal of the American Society of Clinical Oncology 2009;27:2630–7.

47. Dose Schwarz J, Bader M, Jenicke L, Hemminger G, Jänicke F, Avril N, Early prediction of response to chemotherapy in metastatic breast cancer using sequential 18F-FDG PET,Journal of nuclear medicine : official publication, Society of Nuclear Medicine 2005;46:1144–50.

48. Jansson T, Westlin JE, Ahlström H, Lilja A, Långström B, Bergh J, Positron emission tomography studies in patients with locally advanced and/or metastatic breast cancer: a method for early therapy evaluation? Journal of clinical oncology : official journal of the American Society of Clinical Oncology 1995;13:1470–7.

49. Ueda S, Tsuda H, Saeki T, Omata J, Osaki A, Shigekawa T, et al, Early metabolic response to neoadjuvant letrozole, measured by FDG PET/CT, is correlated with a decrease in the Ki67 labeling index in patients with hormone receptor-positive primary breast cancer: a pilot study,Breast cancer (Tokyo, Japan) 2011;18:299–308.

50. Mortazavi-Jehanno N, Giraudet A-L, Champion L, Lerebours F, Le Stanc E, Edeline V, et al, Assessment of response to endocrine therapy using FDG PET/CT in metastatic breast cancer: a pilot study, European journal of nuclear medicine and molecular imaging 2012;39:450–60.

51. Forero-Torres A, Saleh MN, Galleshaw JA, Jones CF, Shah JJ, Percent IJ, et al, Pilot trial of preoperative (neoadjuvant) letrozole in combination with bevacizumab in postmenopausal women with newly diagnosed estrogen receptor- or progesterone receptor-positive breast cancer, Clinical breast cancer 2010;10:275–80.

52. Mortimer JE, Dehdashti F, Siegel BA, Trinkaus K, Katzenellenbogen JA, Welch MJ, Metabolic flare: indicator of hormone responsiveness in advanced breast cancer, Journal of clinical oncology : official journal of the American Society of Clinical Oncology 2001;19:2797–803.

53. Young H, Baum R, Cremerius U, Herholz K, Hoekstra O, Lammertsma AA, et al, Measurement of clinical and subclinical tumour response using [18F]-fluorodeoxyglucose and positron emission tomography: review and 1999 EORTC recommendations. European Organization for Research and Treatment of Cancer (EORTC) PET Study Group, European journal of cancer (Oxford, England : 1990) 1999;35:1773–82.

54. Baselga J, Bradbury I, Eidtmann H, Di Cosimo S, Aura C, de Azambuja E, Gomez H, Dinh P, Fauria K, Van Dooren V, Paoletti P, Goldhirsch A, Chang TW, Lang I, Untch M GR and P-GM, First results of the Neo-ALTTO trial (BIG 01-06 / EGF 106903): A phase III, randomized, open label, neoadjuvant study of lapatinib, trastuzumab, and their combination plus paclitaxel in women with HER2-positive primary breast cancer; in : San Antonio Breast Cancer Conference (SABC), 2010, pp Abstract S3–3.

55. Gámez C, Flamen P, Holmes E RJ, Gebhart G, Di Cosimo S EH, Piccart-Gebhart M BJ and de AE, Team on behalf of the N-AS: FDG-PET/CT for Early Prediction of Response to Neoadjuvant Lapatinib, Trastuzumab, and their Combination in HER2 Positive Breast Cancer Patients: The Neo-ALTTO PET Study Results; European Society for Medical Oncology Annual Meeting. 2011, p Abstract S–013.

56. Fuereder T, Wanek T, Pflegerl P, Jaeger-Lansky A, Hoeflmayer D, Strommer S, et al, Gastric cancer growth control by BEZ235 in vivo does not correlate with PI3K/mTOR target inhibition but with [18F]FLT uptake, Clinical cancer research : an official journal of the American Association for Cancer Research 2011;17:5322–32.

57. Kinross KM, Brown DV, Kleinschmidt M, Jackson S, Christensen J, Cullinane C, et al, In vivo activity of combined PI3K/mTOR and MEK inhibition in a Kras(G12D);Pten deletion mouse model of ovarian cancer, Molecular cancer therapeutics 2011;10:1440–9.

58. Aide N, Kinross K, Cullinane C, Roselt P, Waldeck K, Neels O, et al, 18F-FLT PET as a surrogate marker of drug efficacy during mTOR inhibition by everolimus in a preclinical cisplatin-resistant ovarian tumor model. Journal of nuclear medicine : official publication, Society of Nuclear Medicine 2010;51:1559–64.

59. Brepoels L, Stroobants S, Verhoef G, De Groot T, Mortelmans L, De Wolf-Peeters C, (18)F-FDG and (18)F-FLT uptake early after cyclophosphamide and mTOR inhibition in an experimental lymphoma model, Journal of nuclear medicine : official publication, Society of Nuclear Medicine 2009;50:1102–9.

60. Wei LH, Su H, Hildebrandt IJ, Phelps ME, Czernin J, Weber WA, Changes in tumor metabolism as readout for Mammalian target of rapamycin kinase inhibition by rapamycin in glioblastoma, Clinical cancer research : an official journal of the American Association for Cancer Research 2008;14:3416–26.

61. Wahl RL, Jacene H, Kasamon Y, Lodge MA, From RECIST to PERCIST: Evolving Considerations for PET response criteria in solid tumors, Journal of nuclear medicine : official publication, Society of Nuclear Medicine 2009;50 Suppl 1:122S–50S.

62. Marco Gerlinger, M.D., Andrew J. Rowan, B.Sc., Stuart Horswell, M.Math., James Larkin, M.D. PD, David Endesfelder, Dip.Math., Eva Gronroos, Ph.D., Pierre Martinez, Ph.D., Nicholas Matthews BS, Aengus Stewart, M.Sc., Patrick Tarpey, Ph.D., Ignacio Varela, Ph.D., Benjamin Phillimore, B.Sc., Sharmin Begum MS, Neil Q. McDonald, Ph.D., Adam Butler, B.Sc., David Jones, M.Sc., Keiran Raine, M.Sc., Calli Latimer BS, Claudio R. Santos, Ph.D., Mahrokh Nohadani, H.N.C., Aron C. Eklund, Ph.D., Bradley Spencer-Dene PD, Graham Clark, B.Sc., Lisa Pickering, M.D., Ph.D., Gordon Stamp, M.D., Martin Gore, M.D., Ph.D., Zoltan Szallasi MD, et al, Intratumor Heterogeneity and Branched Evolution Revealed by Multiregion Sequencing, New England Journal of Medicine 2012;366.

63. Ignatiadis M, Reinholz M, Minimal Residual Disease and Circulating Tumor Cells in Breast Cancer, Breast cancer research BCR 2011;195:222.

64. Schwarzenbach H, Hoon DSB, Pantel K, Cell-free nucleic acids as biomarkers in cancer patients, Nature Reviews Cancer 2011;11:426–437.

65. McBride DJ, Orpana AK, Sotiriou C, Joensuu H, Stephens PJ, Mudie LJ, et al, Use of cancer-specific genomic rearrangements to quantify disease burden in plasma from patients with solid tumors, Genes chromosomes cancer 2010;49:1062–1069.

66. G. Jerusalem, S. Ellard, A. Fasolo, A. Awada, C. Manlius, L. Vittori, C. Massacesi, T. Sahmoud CM and JB, Non-Infectious pneumonitis (NIP) in breast cancer (BC) patients treated with Everolimus (Afinitor TM) containing therapy: Analysis of five studies, Cancer research 2009;69:Suppl 3 Abstract 1115.

67. Bhojani N, Jeldres C, Patard J-J, Perrotte P, Suardi N, Hutterer G, et al, Toxicities associated with the administration of sorafenib, sunitinib, and temsirolimus and their management in patients with metastatic renal cell carcinoma, European Urology 2008;53:917–930.

68. Di Lorenzo G, Porta C, Bellmunt J, Sternberg CN, Kirkali Z, Staehler MD, et al, Toxicities of targeted therapy and their management in kidney cancer, European Urology 2011;59:526–40.

17. APPENDICES

Appendix 1: Screening, Monitoring and management for HBV

Screening HBV

Consider screening for hepatitis B for the following patients:

1. All patients who have lived in Asia, Africa, Central and South America, Eastern Europe, Spain, Portugal and Greece.
2. Patients with any of the following risk factors:
    a. Known or suspected past hepatitis B infection
    b. Blood transfusion(s) prior to 1990
    c. Current or prior IV drug users
    d. Current or prior dialysis
    e. Household contact with hepatitis B infected patient(s)
    f. Current or prior high risk sexual activity
    g. Body piercing or tattoos
    h. Mother known to have hepatitis B
    i. History suggestive of hepatitis B infection e.g. dark urine, jaundice, right upper quadrant pain
3. Additional patients at the discretion of the investigator

Serologic Markers to be evaluated for screening of HBV

1. HBV-DNA: Serum hepatitis B DNA assay
2. HBsAg: Hepatitis B surface antigen
3. HBsAb: Hepatitis B surface antibody
4. HBcAb: Hepatitis B core antibody

All screening evaluations will be performed within 21 days prior to treatment Day 1 except for patients with positive baseline hepatitis B or C results for whom the screening period can be extended to 28 days to allow time for prophylactic treatment (see below).

Monitoring and Prophylactic treatment of Hepatitis B reactivation

Table 1-A provides details of monitoring and prophylactic therapy according to the baseline results of viral load and serologic markers testing

Table 1-A: Action to be taken based on screening HBV results

**Test**	**Result**				
**HBV-DNA**	+	+ or -	-	-	-
**HBsAg**	+ or -	+	-	-	-
**HBs Ab**	+ or -	+ or -	+<br>and no prior HBV vaccination	+ or -	-<br>or + with prior HBV vaccination
**HBcAb**	+ or -	+ or -	+ or -	+	-
**Recommendation**	Prophylaxis treatment should be started 1-2 weeks prior to first dose of study drug<br>Monitor HBV-DNA approximately every 6 weeks		No prophylaxis<br>Monitor HBV-DNA approximately every 4 weeks		No specific action

Antiviral prophylaxis therapy should continue for at least 4 weeks after last dose of study drug.

Management of HBV reactivation

For hepatitis B reactivation, definition and management guidelines see Table 6-9 Guidelines for management of hepatitis B.

Table 1-B: Guidelines for management of hepatitis B

**HBV reactivation (with or without clinical signs and symptoms)***	
For patients with baseline results:<br>Positive HBV-DNA<br>**OR**<br>positive HBsAg<br>-------------------------------------<br>reactivation is defined as:<br>**[**Increase of 1 log in HBVDNA<br>relative to baseline<br>HBV-DNA value **OR** new appearance of measurable HBV-DNA**]**<br>**AND**<br>ALT elevation x 5 ULN	**Treat**: Start a second antiviral<br>AND<br>Interrupt study drug administration until resolution:<br>• ≤ grade 1 ALT (or baseline ALT, if > grade 1) and<br>• ≤ baseline HBV-DNA levels<br>**If resolution occurs within ≤ 28 days** study drug should be restarted at one dose lower, if available. (see Table 6-2Study drug dose reductions) If the patient is already receiving the lowest dose of study drug according to the protocol, the patient should restart at the same dose after resolution. Both antiviral therapies should continue at least 4 weeks after last dose of study drug.<br>**If resolution occurs > 28 days** Patients should discontinue study drug but continue both antiviral therapies at least 4 weeks after last dose of study drug.
For patients with baseline results:<br>Negative HBV-DNA **and** HBsAg<br>**AND**	**Treat** : Start first antiviral medication<br>AND<br>**Interrupt** study drug administration until resolution:<br>• ≤ baseline HBV-DNA levels<br>**If resolution occurs within ≤ 28 days** study drug should be restarted

**[**Positive HBs Ab (with no prior history of vaccination against HBV), **OR** positive HBcAb**]**<br>------------------------------------<br>reactivation is defined as: New appearance of measurable HBV-DNA	at one dose lower, if available. (see Table 6-2Study drug dose reductions) If the patient is already receiving the lowest dose of study drug according to the protocol, the patient should restart at the same dose after resolution. Antiviral therapy should continue at least 4 weeks after last dose of study drug.<br>**If resolution occurs > 28 days** Patients should discontinue study drug but continue antiviral therapy at least 4 weeks after last dose of study drug.

*All reactivations of hepatitis B are to be recorded as grade 3 (CTCAE v 3.0 Metabolic Laboratory/Other: Viral Re-activation), unless considered life threatening by the investigator; in which case they should be recorded as grade 4 (CTCAE v 3.0 Metabolic Laboratory/Other: Viral Re-activation).

Date of viral reactivation is the date on which **both** DNA and ALT criteria were met (e.g. for a patient who was HBV-DNA positive on 01-JAN-10 and whose ALT reached ≥ 5 × ULN on 01-APR-10, the date of viral reactivation is 01-APR-10).

Appendix 2: Screening, Monitoring and management for HCV

Screening for HBC

Consider screening for hepatitis C in the following patients:

1. Known or suspected past hepatitis C infection (including patients with past interferon 'curative' treatment)
2. Blood transfusions prior to 1990
3. Current or prior IV drug users
4. Current or prior dialysis
5. Household contact of hepatitis C infected patient(s)
6. Current or prior high-risk sexual activity
7. Body piercing or tattoos
8. At the discretion of the investigator

Serologic Markers to be evaluated for screening of HCV

1. HCV quantitative RNA-PCR

All screening evaluations will be performed within 21 days prior to treatment Day 1 except for patients with positive baseline hepatitis B or C results for whom the screening period can be extended to 28 days to allow time for prophylactic treatment (see below)

Monitoring for HCV

The following two categories of patients should be monitored every 6 weeks for HCV reactivation:

- Patients with detectable HCV RNA-PCR test or other at baseline.
- Patients known to have a history of HCV infection, despite a negative viral load test at baseline (including those that were treated and are considered 'cured')

For definition of hepatitis C reactivation and the management guidelines, see Table 2-A Guidelines for management of hepatitis C.

Table 2-A: Guidelines for management of hepatitis C

**HCV reactivation***	
For patients with baseline results:	Discontinue study drug

Detectable HCV-RNA,<br>reactivation is defined as:<br>ALT elevation x 5 ULN	
For patients with baseline results:<br>Knowledge of past hepatitis C<br>infection with no detectable HCVRNA,<br>reactivation is defined as:<br>New appearance of detectable<br>HCV-RNA	Discontinue study drug

* All reactivations of hepatitis C are to be recorded as grade 3 (CTCAE v 3.0 Metabolic Laboratory/Other: Viral Re-activation), unless considered life threatening by the investigator; in which case they should be recorded as grade 4 (CTCAE v 3.0 Metabolic Laboratory/Other: Viral Re-activation).

EU Number: 2024-511850-34-00

Sponsor Protocol Number: IJB-SYNERGY-012017

ClinicalTrials.gov Number: NCT03616886

A Phase I/II Study of Paclitaxel plus Carboplatin and Durvalumab (MEDI4736) with or without Oleclumab (MEDI9447) for Previously Untreated Locally Recurrent Inoperable or Metastatic Triple-Negative Breast Cancer

SYNERGY Trial

Study Chair

Laurence Buisseret, MD, PhD

Co-Study Chair

Christian Maurer, MD

Sponsor:

Institut Jules Bordet

Rue Meylemeersch, 90, 1070 Bruxelles/Brussel
Belgique/België

CTSU Trials – Synergy: ctsu.synergy@hubruxelles.be

Protocol version: 9.0

Issue date: 06/01/2025

CONFIDENTIAL

Information and data included in this protocol contain trade secrets and privileged or confidential information which is the property of the Institut Jules Bordet. No person is authorised to make it public without the written permission of the Institut Jules Bordet

PROTOCOL WRITING COMMITTEE

Christian Maurer, MD	Resident<br>Department I of Internal Medecine and Center for Integrated Oncology Aachen Bonn Cologne Duesseldorf; University of Cologne, Cologne, Germany.
Laurence Buisseret, MD, PhD	Medical Oncologist<br>Department of Medical Oncology<br>Institut Jules Bordet<br>Brussels, Belgium
Michail Ignatiadis, MD, PhD	Medical Oncologist<br>Department of Medical Oncology<br>Institut Jules Bordet<br>Brussels, Belgium
Clinical Trials Support Unit	Institut Jules Bordet<br>Brussels, Belgium
Marianne Paesmans, MSc	Study statistician<br>Institut Jules Bordet<br>Brussels, Belgium

PROTOCOL APPROVAL – Signature Page

EudraCT Number: 2017-004651-23

Sponsor Protocol Number: IJB-SYNERGY-012017

ClinicalTrials.gov Number: NCT03616886

The signature below constitutes the approval of this protocol and the attachments, and provides the necessary assurances that this study will be conducted according to all stipulations of the protocol, including all statements regarding confidentiality, and according to national, local legal and regulatory requirements and applicable European regulations and ICH GCP guideline.

Sponsor

Title and Name	Approval date	Signature

Study Chair(s)

Title and Name	Approval date	Signature

National Coordinator

Title and Name	Function	Approval date	Signature
	National Coordinator		

Site Principal Investigator

Title and Name	Function	Approval date	Signature
	Site Principal Investigator		

The name and function of the representatives of the sponsor authorised to sign the protocol or any substantial modification to the protocol are:
•Doctor Martine Piccart, Scientific Director, Institut Jules Bordet
•Doctor Michail Ignatiadis, Leader of the Academic Promoting Team, Institut Jules Bordet

The contact details of the Institut Jules Bordet are:
Dr. Laurence Buisseret, MD, PhD
Phone: +32 (0) 2 541 38 43
E-mail: laurence.buisseret@hubruxelles.be

DOCUMENT HISTORY

Version	Protocol version release date	Short description of modifications	Submission as substantial amendment
1.0	30/06/2018	Initial release.<br>This document is based on the CTSU protocol template v2.	☐ Yes<br>☐ No<br>☒ Not applicable
2.0	10/01/2019	• Study rationale update<br>• Clarification DLT definition<br>• Section deleted as present in the IMP management manual<br>• Clarification regarding the steroids in the allowed and prohibited concomitant treatments<br>• Clarification regarding immunotherapy toxicity and management<br>• Clarification for pregnancy test (urine vs serum)<br>• Modification due to new IB version of durvalumab (v13)<br>• Modification to be consistent within all protocol sections<br>• Wording clarifications	☒ Yes<br>☐ No<br>☐ Not applicable
2.1	05/03/2019	Modification in response to the request for additional information in Belgium (AMD-0049):<br>• Modification of the section about AESIs and potential Hy's law case<br>• Wording clarifications	☐ Yes<br>☐ No<br>☒ Not applicable
3.0	15/04/2019	Modifications due to the analyses of the Phase I data :<br>• Validation of the RP2D at 3000mg (DL0) for oleclumab<br>• Carboplatin dose reduced from AUC 2 to AUC 1.5 due to neutropenia experienced in the 6 patients included in the Phase I part<br>• G-CSF advised since neutropenia grade 2 (< 1500 /µl)<br>Other Modifications unrelated to the analyses of the Phase I data :<br>• Study rationale update<br>• Clarification of a few inclusion criteria<br>• Baseline imaging timeframe widen<br>• Modifications of the Guidance for specific Immune-related Adverse Events<br>• Wording clarifications for translational research<br>• Addition of a central review regarding imaging at baseline, weeks 8, 16 and 24<br>• Information about GDPR requirements	☒ Yes (BELGIUM)<br>☒ No (FRANCE : initial submission)<br>☐ Not applicable

3.1	06/08/2019	Modification in response to the request for additional information in France (INI-0025):<br>• Chemotherapy (Re-)Treatment Criteria modified<br>• Inclusion/exclusion criteria modified (exclusion criteria 9 & 12)<br>• Screening echocardiography added for all patients previously treated with anthracyclines who do not have a recent cardiac work up (< 6 months)<br>• Clarification regarding haematological and coagulation criteria for metastatic lesion biopsy during week 3<br>• Independent Data Monitoring Committee (IDMC) added for the Phase II	☒ Yes (BELGIUM)<br>☐ No<br>☒ Not applicable (FRANCE)
4.0	06/02/2020	• Update of the study rationale (Impassion 130), the ASCO/CAP criteria/guidelines<br>• Clarification regarding the eligibility and screening samples of the de novo patients<br>• Inclusion/exclusion criteria added to be consistent with French requirements<br>• Monitoring of the lipase has been added in assessments<br>• Clarification of the definition of the visit at week 13 and the EOT visit<br>• Clarification of a patient evaluable<br>• Addition of websites where the study can be found<br>• Wording clarification	☒ Yes(BELGIUM & FRANCE)<br>☐ No<br>☐ Not applicable
5.0	05/02/2021	• Update of the study rationale<br>• Inclusion/ exclusion criteria clarified (IC 9, IC 11 & EC1)<br>• Clarification regarding AURORA or similar molecular screening program<br>• Clarification about chemotherapy logistics and accountability<br>• Note added about hormonal contraception<br>• Modification of the allowed & prohibited concomitant treatments<br>• Modifications of the Guidance for specific Immune-related Adverse Events and for chemotherapy hepatic toxicities<br>• Clarification in the schedule of assessment (coagulation evaluation and samples collected)<br>• AESI reporting adapted<br>• Safety events reporting by fax removed<br>• Clarification regarding samples to be collected for the translational research<br>• Clarification for the interim analysis<br>• Wording clarifications	☒ Yes (BELGIUM & FRANCE)<br>☐ No<br>☐ Not applicable
6.0	14/10/2021	• Update of the study rationale<br>• Results of interim analysis and IDMC recommendation added.<br>• Stop of recruitment added	☒ Yes (BELGIUM & FRANCE)<br>☐ No

		• For patients under treatment : possibility to cross-over from Arm A to B was added<br>• AESI definition updated<br>• New appendix 1 – toxicity management guidelines	☐ Not applicable
7.0	10/03/2023	• New appendix 1 – toxicity management guidelines<br>• Update of schedule of assessment after week 104	☒ Yes (BELGIUM & FRANCE)<br>☐ No<br>☐ Not applicable
8.0	23/09/2024	• Update according to the new CTR regulation	☒ Yes (BELGIUM & FRANCE)<br>☐ No<br>☐ Not applicable
9.0	06/01/2025	• New appendix 1 – toxicity management guidelines	☒ Yes (BELGIUM & FRANCE)<br>☐ No<br>☐ Not applicable

TABLE OF CONTENTS

LIST OF ABBREVIATIONS 13
SYNOPSIS 16
1. BACKGROUND AND SCIENTIFIC RATIONALE 30
1.1. Background 30
1.1.1. Triple-negative Breast Cancer 30
1.1.2. Current Treatment Options 30
1.1.2.1. Anthracyclines 30
1.1.2.2. Taxanes 30
1.1.2.3. Platinum Salts 30
1.1.2.4. Combination Treatments 31
1.1.3. Targeted Treatment Approaches 31
1.1.4. Immunotherapy 31
1.1.4.1. Targeting PD-L1 with durvalumab (MEDI4736) 33
1.1.4.2. Targeting CD73 with oleclumab (MEDI9447) 33
1.2. Rationale 34
1.2.1. Choosing TNBC as Target Disease 34
1.2.2. Choosing Paclitaxel and Carboplatin as Chemotherapeutical Backbone 34
1.2.3. Adding oleclumab, an anti-CD73 agent to durvalumab, a PD-L1 inhibitor 35
1.3. Potential Risks and Benefits 35
2. OBJECTIVES AND ENDPOINTS OF THE STUDY 36
2.1. Objectives 36
2.1.1. Primary objectives 36
2.1.2. Secondary objectives 36
2.1.3. Exploratory objectives 37
2.2. Endpoints 37
2.2.1. Primary endpoints 37
2.2.2. Secondary endpoints 37
2.2.3. Exploratory endpoints 38
3. STUDY DESIGN 38
3.1. Phase I 38
3.1.1. Paclitaxel and Carboplatin 38
3.1.2. Durvalumab 38
3.1.3. Oleclumab 39
3.2. Phase II 40
3.2.1. Paclitaxel and Carboplatin 41
3.2.1. Durvalumab 41
3.2.2. Oleclumab 41
3.3. Phase I and Phase II 41

3.3.1. Drugs administration ... 41
3.3.2. Chemotherapy Pre- and Post-medication ... 42
3.3.3. Durvalumab/Oleclumab (MEDI9447) Pre- and Post-medication ... 42
3.3.4. Chemotherapy (Re-)Treatment Criteria ... 42
3.3.5. Discontinuation and duration of therapy ... 43
3.3.6. Pseudo-progression related to immunotherapy ... 43
3.3.7. Imaging assessment ... 43
4. SELECTION OF SUBJECTS ... 44
4.1. Inclusion criteria ... 44
4.2. Exclusion criteria ... 45
4.3. Subject registration/inclusion procedure ... 47
5. CRITERIA FOR SUBJECT WITHDRAWAL ... 48
6. STUDY TREATMENTS ... 49
6.1. Investigational Medicinal Product: **Durvalumab** ... 49
6.1.1. Drug information ... 49
6.1.1.1. Formulation ... 49
6.1.1.2. Preparation ... 49
6.1.1.3. Therapeutic regimen and dose ... 49
6.1.1.4. Stability and storage ... 49
6.1.1.5. Labelling ... 50
6.1.1.6. Drug logistics and accountability ... 50
6.1.2. Toxicity ... 50
6.2. Investigational Medicinal Product: **Oleclumab** ... 50
6.2.1. Drug information ... 50
6.2.1.1. Formulation ... 50
6.2.1.2. Preparation ... 50
6.2.1.3. Therapeutic regimen and dose ... 50
6.2.1.4. Stability and storage ... 51
6.2.1.5. Labelling ... 51
6.2.1.6. Drug logistics and accountability ... 51
6.2.2. Toxicity ... 51
6.3. Investigational Medicinal Product: **Paclitaxel** ... 51
6.3.1. Drug information ... 51
6.3.1.1. Formulation ... 52
6.3.1.2. Preparation ... 52
6.3.1.3. Therapeutic regimen and dose ... 52
6.3.1.4. Stability and storage ... 52
6.3.1.5. Labelling ... 52
6.3.1.6. Drug logistics and accountability ... 52

6.3.2. Toxicity 52
6.4. Investigational Medicinal Product: **Carboplatin** 52
6.4.1. Drug information 52
6.4.1.1. Formulation 53
6.4.1.2. Preparation 53
6.4.1.3. Therapeutic regimen and dose 53
6.4.1.4. Stability and storage 53
6.4.1.5. Labelling 53
6.4.1.6. Drug logistics and accountability 53
6.4.2. Toxicity 53
6.5. Non-Investigational Medicinal Product(s) 54
6.5.1. Chemotherapy pre- and post-medication 54
6.5.2. Gadolinium-based contrast agents 54
6.5.3. Iodinated contrast agent (referred henceforth as iodine) 54
6.6. Contraception 54
6.7. Concomitant treatments 55
6.7.1. Allowed Concomitant treatments 55
6.7.2. Prohibited Concomitant treatments and procedures 55
6.7.3. Incompatibilities/Drug Interactions 56
6.7.3.1. Durvalumab 56
6.7.3.2. Oleclumab 56
6.7.3.3. Paclitaxel 56
6.7.3.4. Carboplatin 56
7. TOXICITY MANAGEMENT AND DOSE ADJUSTMENTS 57
7.1. General Remarks 57
7.2. Dose-limiting Toxicities of oleclumab (phase I part) 58
7.3. Durvalumab and oleclumab (phase I and II) 59
7.3.1. Immune-related Adverse Events 59
7.3.2. Infusion-related Reactions 62
7.4. Chemotherapy 63
7.4.1. Hematologic Toxicities 64
7.4.2. Gastrointestinal Toxicities 64
7.4.3. Neurological Toxicities 65
7.4.4. Hepatic Toxicities 65
7.4.5. Renal Toxicities 65
7.4.6. Infusion-related Reactions (IRRs) 66
8. SCHEDULE OF ASSESSMENTS 66
8.1. Screening period 66
8.2. Study treatment period 68

8.2.1. Chemotherapy in combination with immunotherapy (= during the first 12 weeks) 68
8.2.2. Maintenance treatment with immunotherapy (= after chemotherapy until the end of treatment) ... 69
8.2.2.1. From end of chemotherapy until week 104 ... 69
8.2.2.2. From week 104 until end of treatment visit: ... 70
8.3. End of treatment visit ... 71
8.4. Efficacy Follow-up period ... 71
8.5. Survival Follow-up Period ... 72
8.6. Tables of Assessments ... 72
9. ASSESSMENT OF EFFICACY ... 78
9.1. Efficacy endpoints ... 78
9.2. Methods for assessing efficacy endpoints ... 78
9.2.1. Documentation of Progression ... 78
9.2.2. Documentation of iBOR ... 78
10. ASSESSMENT OF SAFETY ... 79
10.1. Safety parameters and Methods for assessing safety parameters ... 79
10.2. Pharmacovigilance parameters definitions and reporting guidance ... 79
10.2.1. Adverse Events ... 79
10.2.1.1. Definitions ... 79
10.2.1.2. AE intensity ... 80
10.2.1.3. Relationship to study treatments ... 80
10.2.1.4. Reporting of adverse events ... 81
10.2.2. Serious Adverse Events ... 81
10.2.2.1. Definitions ... 81
10.2.2.2. Reporting of serious adverse events by investigators ... 82
10.2.2.3. Reporting of serious adverse events by sponsor ... 83
10.2.3. Exceptions to AEs/SAEs reporting ... 83
10.2.4. Adverse Events of Special Interest for durvalumab: immune-mediated adverse events ... 84
10.2.4.1. Definition ... 84
10.2.4.2. Reporting AESIs for durvalumab ... 85
10.2.5. Adverse events of special interest for oleclumab: cardiovascular adverse events 85
10.2.5.1. Definition ... 85
10.2.5.2. Reporting AESIs for oleclumab ... 85
10.2.6. Potential Hy's law case ... 86
10.2.6.1. Definition ... 86
10.2.6.2. Reporting ... 86
10.2.7. Pregnancy ... 86

10.2.8. Overdose, misuse and abuse 87
10.2.8.1. Definitions 87
10.2.8.2. Reporting of overdose, misuse and abuse 87
10.2.9. Medication Error 87
10.2.9.1. Definition 87
10.2.9.2. Reporting of medication error 87
11. TRANSLATIONAL RESEARCH(ES) 87
11.1. Overview 87
11.1.1. Breast primary tumour tissue sample - mandatory 88
11.1.2. Metastatic/recurrent lesion tissue samples 88
11.1.2.1. Biopsy at screening - mandatory 88
11.1.2.2. Biopsy “on treatment” - mandatory 88
11.1.2.3. Biopsy “at disease progression” - optional 89
11.1.3. Blood samples collection 89
11.2. Objectives 89
11.3. Timelines 90
11.4. Biological material handling 91
11.5. Central biobanking of biological material collected during the trial and Destruction 91
11.6. Future undefined research 92
12. STATISTICAL CONSIDERATIONS 92
12.1. Statistical design 92
12.1.1. Sample size 92
12.1.2. Study Duration 93
12.1.2.1. Expected Accrual Time 93
12.1.2.2. Expected Duration of the Trial 93
12.1.3. Randomization and Stratifications for Phase II 93
12.2. Statistical plan 93
12.3. Interim Analysis 94
12.3.1. Phase I: Safety Interim Analysis 94
12.3.2. Phase II: Efficacy and Safety Interim Analysis 94
12.4. Statistical Methods 95
12.4.1. Efficacy Parameters 95
12.4.1.1. CBR and DCR 95
12.4.1.2. DOR, PFS and OS 95
12.4.1.3. Data Recoding and Display 95
13. FIRST ACT OF RECRUITMENT 96
14. SUSPENSION OR TRIAL TERMINATION 96
15. END OF STUDY 96

16. DIRECT ACCESS TO SOURCE DATA/DOCUMENTS 96
17. QUALITY CONTROL AND QUALITY ASSURANCE 96
17.1. Quality control 96
17.2. Quality assurance 97
18. REGULATORY AND ETHICAL CONSIDERATIONS 97
18.1. Obligations 97
18.1.1. Trial Master File 97
18.1.2. Regulations, laws and guidelines 97
18.1.3. Authorisation 97
18.1.4. Modifications 98
18.1.5. Notifications 98
18.1.6. Summary of results 98
18.2. Informed consent form and procedure for obtaining patient’s consent 98
18.2.1. The informed consent 98
18.2.2. The procedure for obtaining patient’s consent 99
18.3. Subject identification 101
19. DATA HANDLING AND RECORD KEEPING 101
19.1. Investigator’s files and subject’s clinical source documents 101
19.2. Data Quality Assurance 102
19.3. Case Report Forms (CRF) 102
19.4. Source data documentation 103
19.5. Retention of documents 103
19.6. Adequate arrangements to secure data compliant with GDPR 104
19.7. Measures implemented in case of data security breach 105
19.7.1. The procedure if a Data Processor causes a data breach 105
19.7.2. Notification of the data breach 105
19.7.3. Risk assessment 106
19.7.4. Measures to mitigate the adverse effect 106
19.7.5. Record keeping 106
20. STUDY OVERSIGHT: 106
20.1. Scientific Committee 106
20.2. Independent Data Monitoring Committee 107
21. STUDY SPONSORSHIP AND FINANCING 107
22. STUDY INSURANCE 107
23. PUBLICATION POLICY 108
24. REFERENCES 108
Appendix 1: TOXICITY MANAGEMENT GUIDELINES 112
Appendix 2: Privacy Guarantee 112

LIST OF ABBREVIATIONS

Abbreviation	Description
AACR	American Association for Cancer Research
ADCC	Antibody-dependent cell-mediated cytotoxicity
ADL	Activities of Daily Living
ADR	Adverse Drug Reaction
AE	Adverse Event
AESI	Adverse Event of Special Interest
AFMPS	Agence Fédérale des Médicaments et des Produits de Santé
ALT	Alanine Amninotransferase
ALP	Alkaline phosphatase
AMP	Adenosine MonoPhosphate
ANC	Absolute Neutrophil Count
aPTT	activated Partial Thromboplastin Time
AR	Adverse Reaction
ASCO	American Society of Clinical Oncology
AST	Aspartate aminotransferase
AUC	Area Under the Curve
BC	Breast Cancer
BIG	Breast International Group
BOR	Best Overall Response
BRCA	Breast Cancer gene
BUN	Blood Urea Nitrogen
CAP	College of American Pathologists
CB	Clinical Benefit
CBC	Complete Blood Count
CBR	Clinical Benefit Rate
CD	Cluster of Differentiation
CA	Cancer antigen
CEA	Carcinoembryonic antigen
CI	Confidence Interval
CNS	Central Nervous System
CPD	Confirmed Progressive Disease
CR	Complete Response
CRF	Case Report Form
CT	Computed Tomography
CTCAE	Common Terminology Criteria for Adverse Events
CTSU	Clinical Trials Support Unit
DC	Disease Control
DCR	Disease Control Rate
DL	Dose Level
DLT	Dose Limiting Toxicity
DNA	Deoxyribonucleic Acid
DOR	Duration Of Response
DVT	Deep Vein Thrombosis
ECOG	Eastern Cooperative Oncology Group
(e)CRF	(Electronic) Case Report Form
EEA	European Economic Area
eGFR	estimated Glomerular Filtration Rate
EOS	End Of Study
EOT	End Of Treatment

ER	Estogen receptor
EU	European Union
EVCTM	EudraVigilance Clinical Trial Module
FDA	Food and Drug Administration
FFPE	Formalin-Fixed paraffin-embedded
FISH	Fluorescence In Situ Hybridization
FNA	Fine Needle Aspiration
FSCP	Female Subjects of Childbearing Potential
FTIH	First-Time-In-Human
GFR	Glomerular Filtration Rate
GMP	Good Manufacturing Practice
Hb	Haemoglobin
HBV	Hepatitis B Virus
HBsAg	Hepatitis B Surface Antigen
HCV	Hepatitis C Virus
HER2	Human epidermal growth factor receptor 2
HIV	Human Immunodeficiency Virus
HR	Hazard Ratio
IB	Investigator’s Brochure
iBOR	Immune Best Objective Response
IC	Immune Cells
ICB	Immune Checkpoint Blockade
ICD	Immunogenic Cell Death
ICF	Informed Consent Form
ICH-GCP	International Conference on Harmonisation – Good Clinical Practice
iCPD	iRECIST Confirmed Progressive Disease
IDMC	Independent Data Monitoring Committee
IEC	Independent Ethical Committee
IFN	Interferon
IgG	Immunoglobulin G
IgG1κ	Immunoglobulin G1 kappa
IgG1λ	Immunoglobulin G1 lambda
IHC	Immunohistochemistry
IJB	Institut Jules Bordet
IL	Interleukin
ILD	Interstitial lung disease
IMP	Investigational Medicinal Product
INN	International Nonproprietary Names
INR	International Normalized Ratio
irAE	immune-related Adverse Event
IRB	Institutional Review Board
iRECIST	Immune Response Evaluation Criteria In Solid Tumours
IRR	Infusion-Related Reaction
ISF	Investigator’s study file
iUPD	iRECIST Unconfirmed Progressive Disease
IV	Intravenous
LDH	Lactic Dehydrogenase
mAb	Monoclonal antibody
MBC	Metastatic Breast Cancer
MDSC	Myeloid-derived suppression cells
mITT	Modified Intention to treat
MRI	Magnetic Resonance Imaging

NCI-CTCAE	National Cancer Institute - Common Terminology Criteria for Adverse Events
NIMP	Non-Investigational Medicinal Product
NLT	New Lesion Target
NLNT	New Lesion Non-Target
NSAID	Non-Steroidal Anti-Inflammatory Drug
NSCLC	Non-Small Cell Lung Cancer
OR	Overall Response
ORR	Objective Response Rate
OS	Overall Survival
PARP	Poly (ADP-ribose) polymerase
PBMCs	Peripheral Blood Mononuclear Cells
pCR	pathologic Complete Response
PD	Progressive Disease
PD-1	Programmed cell death receptor 1
PD-L1	Programmed cell death receptor ligand 1
PFS	Progression-Free Survival
PK	Pharmacokinetic
PLT	Platelets
PO	Per os
PR	Partial Response
PR	Progesterone receptor
PS	Performance Status
QxW	Every x weeks
RBC	Red Blood Cell Count
RECIST	Response Evaluation Criteria In Solid Tumours
RNA Seq	Ribonucleic Acid Sequencing
RP2D	Recommended Phase II Dose
SABCS	San Antonio Breast Cancer Symposium
SAE	Serious Adverse Event
SAR	Serious Adverse Reaction
SITC	Society for Immunotherapy of Cancer
SD	Stable Disease
SmPC	Summary of Product Characteristics
SPM	Second Primary Malignancy
SUSAR	Suspected Unexpected Serious Adverse Reaction
T1DM	Type 1 Diabetes Mellitus
TB	Tuberculosis
TBL	Total serum bilirubin
T-cells	T-lymphocytes
TCR	T-cells response
TEAE	Treatment Emergent Adverse Event
TILs	Tumour-infiltrating lymphocytes
TNBC	Triple-negative breast cancers
TR	Translational Research
TSH	Thyroid-stimulating Hormone
UAR	Unexpected Adverse Reaction
ULN	Upper Limit of Normal
UPD	Unconfirmed progressive disease
WBC	White Blood Cell Count
WES	Whole Exome Sequencing

SYNOPSIS

<table>
<tr><td>STUDY TITLE</td><td>A Phase I/II Study of Paclitaxel plus Carboplatin and Durvalumab (MEDI4736) with or without Oleclumab (MEDI9447) for Previously Untreated Locally Recurrent Inoperable or Metastatic Triple-negative Breast Cancer</td></tr>
<tr><td>SHORT TITLE</td><td>SYNERGY Trial</td></tr>
<tr><td>SPONSOR</td><td>Institut Jules Bordet (IJB) - Brussels, Belgium</td></tr>
<tr><td>INDICATION(S)</td><td>Previously untreated locally recurrent inoperable or metastatic triple-negative breast cancer (TNBC)</td></tr>
<tr><td>TARGET STUDY POPULATION</td><td>Pre or post-menopausal female subjects with previously untreated, locally recurrent inoperable or metastatic triple-negative breast cancer.</td></tr>
<tr><td>PHASE</td><td>Phase I/II</td></tr>
<tr><td>STUDY DESIGN</td><td>
The trial consists of two parts:
<u>PHASE I</u>
Part 1 is a phase I trial examining the combination of paclitaxel, carboplatin, durvalumab and oleclumab in order to define the recommended phase II dose (RP2D) of oleclumab in this treatment combination.

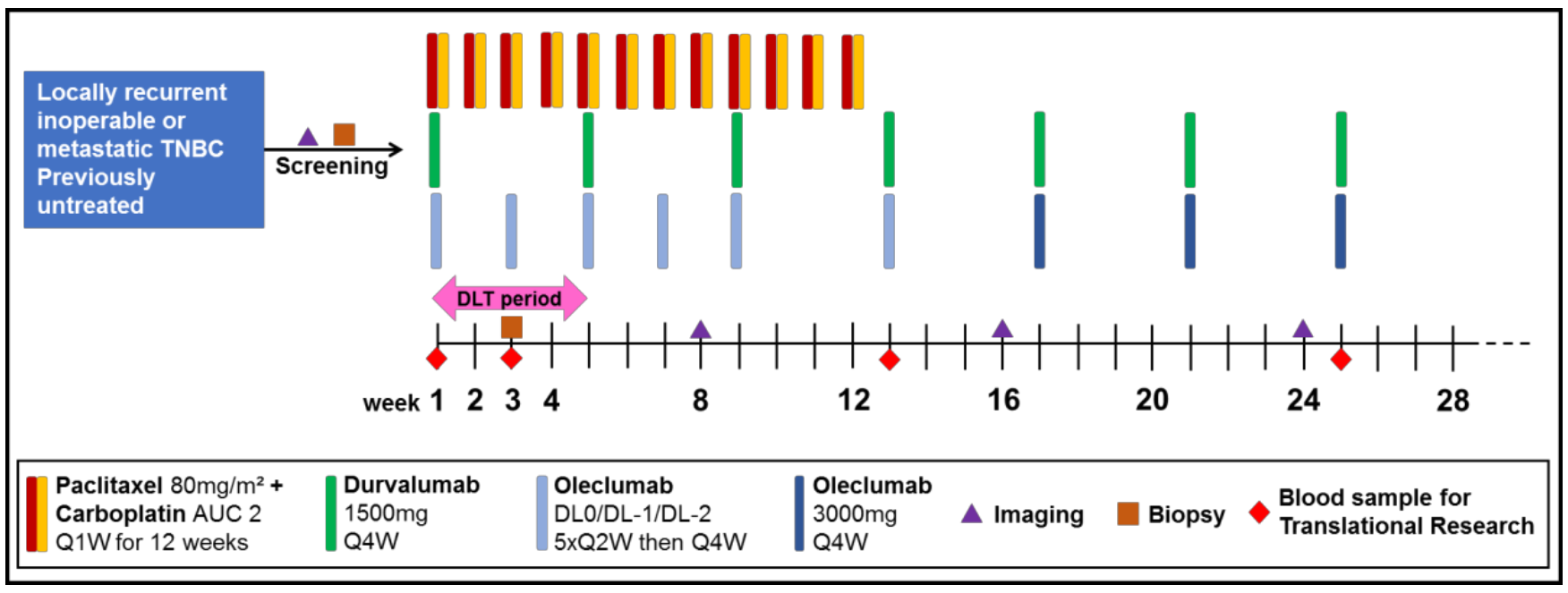

The period for DLT evaluation is defined as the time from receiving the first dose of oleclumab until the planned administration of the third dose; this corresponds to 28 days after receiving the first dose of oleclumab in case no treatment interruption occurred.
Subjects who do not remain in the study for at least 28 days after the first oleclumab administration for reasons other than DLT will be considered non-evaluable for DLT assessment and will be replaced with another subject at the same dose level.
Patients who develop a DLT will stop study treatment permanently.
<u>PHASE II</u>
Part 2 is a multicenter, randomized, open-label trial investigating the role of an anti-CD73 monoclonal antibody (oleclumab) in combination with an anti-PD-L1 antibody (durvalumab) plus chemotherapy (paclitaxel + carboplatin) as first-line treatment for locally recurrent inoperable or metastatic TNBC.
Patients will be randomized between chemotherapy (paclitaxel and carboplatin) with immunotherapy including durvalumab with or without oleclumab (1:1 ratio).
</td></tr>
</table>

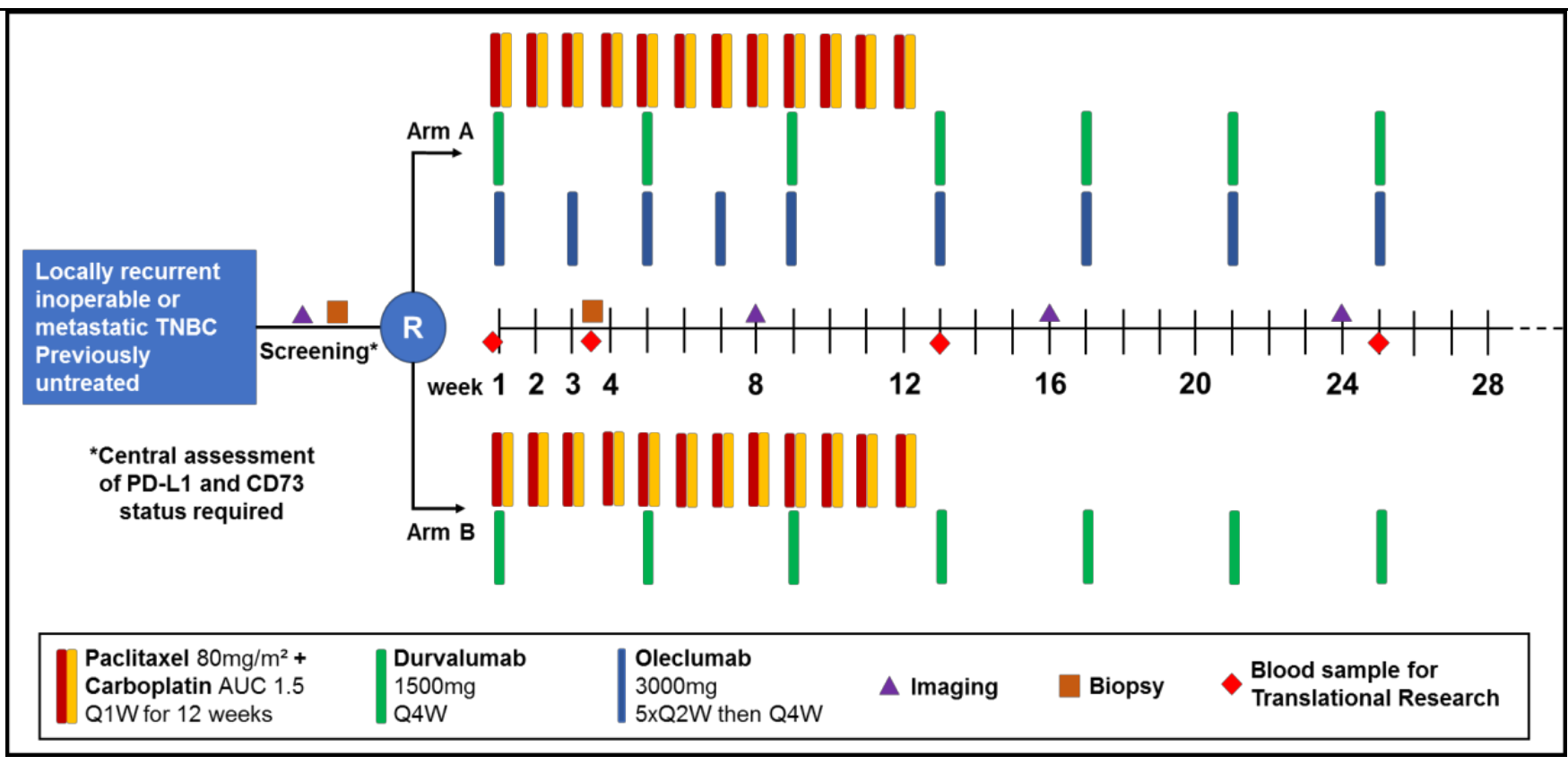

PHASE I and PHASE II

Paclitaxel and carboplatin will be given weekly for a total of 12 injections. Immunotherapy (durvalumab with or without oleclumab) will be given as long as the patient benefits. Premature discontinuation of paclitaxel/carboplatin or discontinuation of durvalumab/oleclumab is indicated in case of:

- Progressive disease (PD) using RECIST v1.1 criteria or iRECIST assessed locally
- Unacceptable toxicity
- Intercurrent illness that necessitates discontinuation of study treatment
- Investigator's decision to withdraw the subject
- Pregnancy
- Subject noncompliance with the study treatment or procedure requirements
- Withdrawal of consent to treatment
- Death
- Administrative reasons requiring cessation of study treatment.

Initial disease status will be evaluated by imaging studies (contrast-enhanced CT scan of the chest, the abdomen and the pelvis or MRI of the chest, the abdomen and the pelvis) during the screening phase. Disease status will be followed by imaging studies at weeks 8 (± 3 days), 16 (± 3 days) and 24 (± 3 days) post start of treatment (allowing efficacy data to be captured as close to 24 weeks post start of study treatment as possible for a more accurate evaluation of DCR). Thereafter imaging will continue every 12 weeks (± 3 days; contrast-enhanced CT scan or MRI) independent of any treatment delays.

Patients who stopped all study treatments for reasons other than PD will continue post-treatment imaging studies for disease status follow-up as described in the schedule of assessment until verified PD, start of a new anti-cancer treatment, withdrawal of consent to study participation, death, or end of the study whichever comes first.

Note: Pseudo-progression related to immunotherapy: Patients experiencing PD as defined by RECIST v1.1 can continue the study treatment in case of good clinical condition assessed by a stable or even improved ECOG performance status. If the next assessment of tumour burden (8 weeks later) confirms PD (as defined by iRECIST) study treatment must be discontinued. Unconfirmed PD as defined by iRECIST (iUPD) can only be assigned for the first 2 imaging assessments (week 8 and week 16) and several times as long as confirmed progression (iCPD) is not confirmed at the next assessment. If PD is not confirmed, reassessments continue as originally planned.

**STUDY RATIONALE**	In metastatic TNBC, single immune checkpoint blockade (ICB) demonstrated clinical activity in a limited subset of patients in early phases trials. Synergistic combinations are warranted in order to improve the response rates and clinical benefit from immune treatments in BC. Several chemotherapeutic agents were demonstrated to induce immunogenic cell death (ICD). This process increases the immunogenicity of neoplastic cells and primes the immune system by stimulating innate immune effectors and by inducing cytotoxic T cell responses. Trials combining ICB with standard chemotherapy are ongoing in advanced TNBC. Phase III trials with chemotherapy +/- ICB as first-line treatments for metastatic TNBC are ongoing. Based on the results of the Impassion 130 trial, the FDA and EMA approved atezolizumab in combination with nab-paclitaxel for adult patients with unresectable locally advanced or metastatic TNBC whose tumours express PD-L1 in 2019. Based on the results of the KEYNOTE-355 trial, the FDA gave an accelerated approval on December 2020 to pembrolizumab for previously untreated locally recurrent unresectable TNBC or metastatic TNBC with combined positive score (CPS)≥10.<br><br>CD73 is an ectoenzyme catalysing the conversion of adenosine monophosphate (AMP) to adenosine and organic phosphate that can be found on the surface of tumour cells, stromal cells such as endothelial cells and certain leukocytes. Extracellular adenosine promotes tumour cell metastasis, angiogenesis and has multiple immunosuppressive functions. In preclinical tumour models, targeted blockade of CD73 reduced tumour growth, metastases and increased the number of tumour-infiltrating $CD8^+$ effector cells and activated macrophages. In BC, CD73 expression is associated with poor prognosis in the TNBC subtype. Patients with high levels of CD73 and low levels of tumour-infiltrating leukocytes in the primary tumour had the worst clinical outcome in a large cohort of TNBC from a phase III clinical trial that compared anthracycline alone to anthracycline-taxane chemotherapy. Furthermore, treatment with anthracyclines, paclitaxel and platinum induced up-regulation of CD73 in human BC cells. Targeting the CD73 pathway to relieve the adenosine-mediated immunosuppression seems a promising strategy in TNBC.<br><br>In mouse models, the potential of adenosine targeting treatment was enhanced when combined with other immunomodulatory treatment as ICB. The combination of ICB targeting PD-1 with an anti-CD73, oleclumab already showed synergistic activity in preclinical models.<br><br>Phase I trials with anti-CD73 compounds or antagonists of the adenosine A2A receptor +/- ICB in advanced solid tumours (including TNBC) are ongoing (NCT02503774; NCT02403193; NCT02655822). Interim safety data on 113 patients and efficacy data for 96 patients (of whom 31 with TNBC) enrolled in a phase I/Ib trial of CPI-444, an A2A antagonist, +/- atezolizumab (NCT02655822) in advanced solid tumours were presented at AACR 2017. Fifty-six percent were resistant or refractory to prior treatment with anti-PD-(L)1 antibodies. Disease control in TNBC was achieved in 41% of those receiving CPI-444 as single agent (n=17) and in 21% of those receiving the combination (n=14) for an overall disease control rate of 32% in 31 patients. CPI-444 was well tolerated with no grade 3 or 4 adverse events with single agent CPI-444.
**OBJECTIVES**	**Primary Objectives**<br><br>PHASE I: To confirm the **safety** of oleclumab in combination with paclitaxel, carboplatin and durvalumab in previously untreated, locally recurrent inoperable or metastatic TNBC patients.<br><br>PHASE II: To evaluate the **clinical benefit** of oleclumab in combination with paclitaxel, carboplatin and durvalumab in previously

	untreated, locally recurrent inoperable or metastatic TNBC patients by the comparing the clinical benefit rate (CBR) at 24 weeks from the 1st dose of study drug administration between patients treated with or without the anti-CD73 antibody oleclumab.<br><br>**Secondary Objectives**<br><br>PHASE I: To determine the **recommended phase II dose** (RP2D) of oleclumab in combination with paclitaxel, carboplatin and durvalumab in previously untreated, locally recurrent inoperable or metastatic TNBC patients.<br><br>PHASE II:<br>1) To evaluate the **efficacy** of oleclumab in combination with paclitaxel, carboplatin and durvalumab in previously untreated, locally recurrent inoperable or metastatic TNBC patients assessed by comparing the objective response rate (ORR; complete response [CR] + partial response [PR]) and the duration of response (DOR) between patients treated with or without the anti-CD73 antibody oleclumab.<br>2) To evaluate the **survival benefit** of oleclumab in combination with paclitaxel, carboplatin and durvalumab in previously untreated locally recurrent inoperable or metastatic TNBC patients by comparing the progression-free survival (PFS) and overall survival (OS) between patients treated with or without the anti-CD73 antibody oleclumab.<br>3) To evaluate the **safety and feasibility** of oleclumab in combination with paclitaxel, carboplatin and durvalumab in previously untreated, locally recurrent inoperable or metastatic TNBC patients.<br>4) To evaluate the efficacy, clinical and survival benefits of oleclumab in combination with paclitaxel, carboplatin and durvalumab in previously untreated, locally recurrent inoperable or metastatic TNBC patients **according to PD-L1 and CD73 expression**.<br><br>**Exploratory Objective**<br><br>PHASE I/II:<br>1) To compare ORR by RECIST v1.1 and iRECIST during the first 24 weeks from the 1st dose of study drug administration.<br>2) To compare PFS by RECIST v1.1 and iRECIST from date of 1st dose of study drug administration
**ENDPOINTS**	**Primary Endpoints**<br><br>PHASE I: Safety and tolerability will be assessed by monitoring frequency, duration and severity of adverse events (AEs).<br><br>PHASE II: Clinical benefit (CB). CB is defined as a patient who achieved CR or PR or demonstrated SD at 24 weeks from the 1st dose of study drug administration based on RECIST v1.1.<br><br>**Secondary Endpoints**<br><br>PHASE I: Safety and tolerability will be assessed by monitoring frequency, duration and severity of adverse events (AEs) including Dose limiting toxicities (DLTs) as defined per protocol.<br><br>PHASE II:<br>1) Objective Response. OR is defined as a patient (in the intent-to treat population) who achieved a CR or PR as best overall response (BOR) based on RECIST v1.1.<br>2) Duration of Response. DOR is defined as the time from documentation of first tumour response to disease progression based on RECIST v1.1.

	3) Progression Free Survival. PFS is defined as the time from 1st study drug administration to the first documented disease progression based on RECIST v1.1 or death due to any cause, whichever occurs first. (Subjects who are alive and progression free at the time of analysis will be censored at the time-point of their last tumour assessment by imaging.)<br>4) Overall Survival. OS is defined as the time from 1st study drug administration to death due to any cause. (Subject without documented death at the time of the analysis will be censored at the date of the last follow-up.)<br>5) Frequency, duration and severity of AEs assessment based on CTCAE 5.0.<br>**Exploratory Endpoint**<br>PHASE I/II:<br>1) Response assessment based on RECIST v1.1 versus iRECIST during the first 24 weeks from the 1st dose of study drug administration.<br>2) PFS assessment based on RECIST v1.1 versus iRECIST
**NUMBER OF SUBJECTS**	**PHASE I**<br>Number of evaluable patients required: minimum of 6, maximum of 18<br>Estimated number of subjects to enrol: minimum of 7, maximum of 21<br>Estimated number of subjects to screen: minimum of 8, maximum of 24<br>**PHASE II**<br>The primary hypothesis is that the combination of chemotherapy + durvalumab + oleclumab increases the clinical benefit rate (CBR) by 20% compared to chemotherapy + durvalumab (i.e. from an expected CBR in the control arm of 40 % to 60% in the experimental arm). The expected CBR in the control arm is based on early results of an ongoing phase Ib/II trial.<br>With a one-sided Type I error (α) 0.1 randomizing 68 patients to each treatment arm will provide 80% power to detect the aforementioned 20% treatment difference in CBR by RECIST (applying Casagrande continuity correction). One interim analysis for futility is planned at an information fraction of 0.50 (68 evaluable patients randomized = 34 patients per arm).<br>Number of evaluable subjects: 136 (68 per treatment arm)<br>Estimated number of subjects to randomize: 150<br>Estimated number of subjects to screen: 180<br>A patient is deemed evaluable if she received at least 1 treatment dose (at least one dose of durvalumab and/or oleclumab).
**INCLUSION CRITERIA**	Patients must meet all the following criteria to be eligible for the study:<br>1. Age of ≥ 18 years<br>2. Female<br>3. Life expectancy of a least 12 weeks<br>4. Body weight above 35kg<br>5. The locally recurrent or metastatic relapse must be histologically confirmed TNBC in patients not previously treated with systemic treatment and which cannot be treated with curative intent. Newly diagnosed patients with de-novo metastatic disease are eligible<br>6. Estrogen receptor (ER) and progesterone receptor (PR) negativity (<1% positive staining cells in the invasive tumour determined locally using IHC per ASCO/CAP criteria)<br>7. Human epidermal growth factor receptor 2 (HER2) negativity (negative IHC staining [score 0 or 1] or negative fluorescence in situ hybridization [FISH] based on the ASCO/CAP guidelines and recommendations from 2013) and determined locally

	Note: patients initially diagnosed with hormone receptor–positive and/or HER2-positive breast cancer OR de novo metastatic patients with a primary tumour hormone receptor-positive (weak positivity or ER negativity and PR positivity) considered as non-clinically relevant are eligible if the tumour biopsy obtained from a local recurrence or distant metastasis site confirms the TNBC disease.<br>8. Confirmed tumour PD-L1 and CD73 IHC assessment as documented through central testing of a representative tumour tissue specimen for stratification purposes (only for phase II)<br>9. Provision of recurrence/metastatic tissue samples from resections, core-needle biopsies or excisional, incisional, punch, or forceps biopsies:<br>- at least 1 FFPE [Formalin-Fixed paraffin-embedded] tumour tissue and 1 frozen core as a priority, if feasible 2 additional fresh tumour tissue cores should be collected too.<br>- Fine-needle aspiration (FNA) (defined as samples that do not preserve tissue architecture and yield cell suspension and/or smears), brushing, and cell pellets from cytology samples are not acceptable.<br>Note 1: If the subject has just performed a metastatic lesion biopsy, she is eligible only if a FFPE tissue sample (or at least 20 unstained slides, freshly cut for the purposes of the study) of the metastatic/recurrent lesion is available. In this situation only, frozen cores are not mandatory.<br>Note 2: In case of a de-novo metastatic disease, if the biopsy of a metastatic lesion is not feasible, the subject is eligible if primary tumour lesion samples (FFPE + frozen core) are available.<br>10. Provision of an archived FFPE diagnostic biopsy or surgical primary tumour sample (or at least 20 unstained slides, freshly cut for the purposes of the study). In case of neoadjuvant treatment (before surgery), the diagnostic biopsy is preferable.<br>11. At least 6 months elapsed between the completion of surgical and/or systemic treatment with curative intent (e.g., the date of primary breast tumour surgery or the date of last adjuvant chemotherapy administration (radiotherapy is not included), whichever occurred last) and first documented local or distant disease recurrence (NOTE: not applicable for de-novo metastatic disease)<br>12. At least one measurable disease based on RECIST v1.1. Tumour lesions in a previously irradiated area are considered measurable, if progression has been demonstrated in such lesions<br>13. Adequate organ function:<br>a) Absolute neutrophil count (ANC) ≥ 1500/µl (without the addition of growth factors)<br>b) Platelets [PLT] ≥ 100000/µl (without the addition of growth factors/prior transfusions)<br>c) Hemoglobin (Hb) ≥ 10 g/dl (without the addition of growth factors/prior transfusions)<br>d) Creatinine ≤ 1.5 x upper limit of normal (ULN) OR estimated glomerular filtration rate (eGFR) ≥ 60 ml/min as calculated using the method standard for the institution. If eGFR is lower than 60 ml/min, a 24-hour urine creatinine clearance can be performed to rule out an underestimation of the eGFR.<br>e) Total serum bilirubin (TBL) ≤ 1.5 x ULN unless the subject has documented Gilbert syndrome in which case up to 3 x ULN is acceptable<br>f) Aspartate and alanine aminotransferase (AST/ALT) ≤ 2.5 x ULN unless liver metastases are present, in which case it must be ≤ 5 x ULN<br>g) International Normalized Ratio (INR) ≤ 1.5 x ULN unless subject is receiving anticoagulant therapy as long as INR and activated partial

	thromboplastin time (aPTT) is within therapeutic range of intended use of anticoagulants<br>14. Performance status (PS) of 0 or 1 on the ECOG Performance scale<br>15. Female subjects of childbearing potential (FSCP) must be willing to use one highly effective method of contraception (detailed at protocol section 6.6.) for the course of the study through 6 months after the last study drug administration. FSCP must have a negative serum pregnancy test done within the 28 days before treatment start. FSCP are those who have not been surgically sterilized or have not been free of menses for at least 1 year.<br>16. Absence of any psychological, familial, sociological or geographical condition potentially hampering compliance with the study protocol and follow-up schedule; those conditions should be discussed with the patient before registration in the trial<br>17. Absence of any concurrent illness that would preclude the evaluation of safety<br>18. Agreement to provide tissue and blood samples for research purposes<br>19. Written informed consent must be given according to ICH/GCP, and national/local regulations before patient enrolment<br><br>Inclusion criterion applicable to FRANCE only<br>20. Affiliated to the French Social Security System<br><br>Note: patients eligible for the Synergy trial are also eligible for the AURORA program (NCT02102165) or any other similar molecular screening program, aiming to understand the molecular aberrations in metastatic BC. Patients can be included in both trials in centres recruiting patients for AURORA or for any similar molecular screening program as long as tumour tissue and blood samples can be collected for both studies. If tissue and blood samples cannot be collected for both, it is the investigator choice (and patient) to decide in which trial the patient will enter.
**EXCLUSION CRITERIA**	Patients who exhibit any of the following conditions at screening are ineligible for admission into the study:<br>1. Active or prior documented autoimmune or inflammatory disorders (including inflammatory bowel disease [e.g., colitis or Crohn's disease], diverticulitis [with the exception of diverticulosis], systemic lupus erythematosus, Sarcoidosis syndrome, or Wegener syndrome [granulomatosis with polyangiitis, Graves' disease, rheumatoid arthritis, hypophysitis, uveitis, etc]). The following are exceptions to this criterion:<br>a) Patients with vitiligo or alopecia<br>b) Patients with hypothyroidism (e.g., following Hashimoto syndrome) stable on hormone replacement<br>c) Any chronic skin condition that does not require systemic therapy<br>d) Patients without active disease in the last 5 years may be included but only after consultation with the sponsor<br>e) Patients with celiac disease controlled by diet alone<br>2. Current or prior treatment with immunosuppressive medication within 14 days prior to enrolment. The following are exceptions to this criterion:<br>a) Intranasal, inhaled, topical steroids, or local steroid injections (e.g., intra articular injection)<br>b) Systemic corticosteroids at physiologic doses not to exceed 10 mg/day of prednisone or its equivalent<br>c) Steroids as premedication for hypersensitivity reactions (e.g., CT scan premedication<br>3. Any live, attenuated vaccine administered within 28 days prior to enrolment or anticipation that such a live attenuated vaccine will be required during the study

	4. Chronic daily treatment with non-steroidal anti-inflammatory drug (NSAID) (occasional use for the symptomatic relief of medical conditions, for example, headache, fever is allowed)<br>5. Active infection including<br>a) Tuberculosis (TB) (clinical evaluation that includes clinical history, physical examination and radiographic findings, and TB testing in line with local practice)<br>b) Hepatitis B (known positive HBV surface antigen (HBsAg) result). Patients with a past or resolved HBV infection (defined as the presence of hepatitis B core antibody [anti-HBc] and absence of HBsAg) are eligible.<br>c) Hepatitis C. Patients positive for hepatitis C (HCV) antibody are eligible only if polymerase chain reaction is negative for HCV RNA.<br>d) Human immunodeficiency virus (positive HIV 1/2 antibodies).<br>6. Treatment with systemic immunostimulatory agents, including but not limited to, interferon (IFN)-alpha, IFN-beta, interleukin (IL)-2, conjugated IL-2 cytokines within 42 days or five half-lives of the drug, whichever is longer, prior to screening<br>7. Previous treatment with immune checkpoint inhibitors (e.g. anti-PD-1, anti-PD-L1 including durvalumab, anti-Cytotoxic T-lymphocyte-associated molecule-4), anti-CD73 antibodies, adenosine A2A receptor antagonists, or prior treatment with CD137 agonists/OX-40 agonists or any other antibody or drug targeting T-cell co-stimulation or other immunomodulatory therapies<br>8. Any unresolved toxicity NCI CTCAE Grade ≥ 2 from previous anticancer therapy with the exception of alopecia, vitiligo and the laboratory values defined in the inclusion criteria<br>9. Known hypersensitivity reactions to the study drugs or to any of the excipients, pre-medications (acetaminophen/paracetamol, diphenhydramine or equivalent anti-histamine and methylprednisolone or equivalent glucocorticoid) and to other platinum containing compounds<br>10. Untreated central nervous system (CNS) metastases and/or carcinomatous meningitis.<br>Subjects with previously treated brain metastases with local treatment (stereotactic radiosurgery or whole brain radiation therapy) may participate provided they have stable brain metastases on a recent brain MRI (performed during the 2 weeks prior inclusion) and have measurable disease outside the CNS.<br>Note: Known brain metastases are considered active (and not eligible for trial), if any of the following criteria are applicable:<br>a) Recent brain imaging demonstrates progression of existing and/or appearance of new lesions<br>b) Neurological symptoms attributed to brain metastases have not returned to baseline<br>c) Steroids were used for management of symptoms related to brain metastases within 14 days of enrolment<br>d) Completion of local therapy for brain metastases within 28 days of enrolment<br>11. Major surgical procedure (as defined by the principal investigator) within 28 days prior to enrolment. Note: Local surgery of isolated lesions for palliative intent is acceptable.<br>12. Uncontrolled intercurrent illness, including but not limited to,<br>a) Symptomatic congestive heart failure, uncontrolled hypertension, unstable angina pectoris, cardiac arrhythmia. Patients previously treated with anthracyclines are eligible if a recent cardiac work up (< 6 months) demonstrated a normal left ventricular ejection fraction (LVEF≥50%).<br>b) Interstitial lung disease

<table>
<tr><td></td><td>
c) Serious chronic gastrointestinal conditions associated with diarrhoea
d) Psychiatric illness/social situations that would limit compliance with study requirement, substantially increase risk of incurring AEs or compromise the ability of the patient to give written informed consent
13. Past medical conditions, including,
a) Class II-IV congestive heart failure
b) Myocardial infarction within 12 months prior enrolment,
c) Deep vein thrombosis (DVT) or thrombo-embolic event within 12 months prior to enrolment
d) History of stroke or transient ischemic attack requiring medical therapy
e) Intra-abdominal inflammatory process within the last 12 months prior to enrolment such as, but not limited to, diverticulitis, peptic ulcer disease, or colitis
f) History of idiopathic pulmonary fibrosis, organizing pneumonia (e.g. bronchiolitis obliterans), drug-induced pneumonitis, idiopathic pneumonitis, or evidence of active pneumonitis
g) History of another primary malignancy except for malignancy treated with curative intent and with no known active disease ≥5 years before the first dose of IP and of low potential risk for recurrence, adequately treated non-melanoma skin cancer or lentigo maligna without evidence of disease, adequately treated carcinoma in situ without evidence of disease
h) Status post allogeneic bone marrow transplantation or solid organ transplantation
14. Pregnant or lactating women.

Exclusion criterion applicable to FRANCE only
15. Vulnerable persons according to the article L.1121-6 of the Public Health Code, adults who are the subjects of a measure of legal protection or unable to express their consent according to article L.1121-8 of the Public Health Code.
</td></tr>
<tr><td>INVESTIGATIONAL MEDICINAL PRODUCT(S) DOSE/ ROUTE/ REGIMEN</td><td>
<u>PHASE I</u>

Patients are treated with paclitaxel, carboplatin, durvalumab and oleclumab.

Paclitaxel and Carboplatin: Paclitaxel 80 mg/m2 IV once a week + carboplatin (AUC 2, calculated according to the Calvert formula) IV once a week (Q1W) for 12 weeks (12 injections of paclitaxel + 12 injections of carboplatin).

Durvalumab is given at a fixed dose of 1500 mg IV every 4 weeks (Q4W)

Oleclumab is given IV every 2 weeks (Q2W). The dose differs according to the respective dose level (DL).

<u>Dose level 0 (DL0):</u>
Oleclumab will be administered at the dose of 3000 mg IV every two weeks (Q2W) for the first 5 administrations then every 4 weeks (Q4W) in a maximum of 6 patients.
First, 3 patients will be enrolled at DL0:
• In case of more than 1 DLT (even prior enrolment of 3 patients), accrual to DL0 stops and accrual starts at the next lower dose level of oleclumab (dose level -1).
• In case of 1 or no DLT out of the 3 patients, 3 additional patients will be enrolled at DL0. Those 3 additional patients will be enrolled at the earliest 28 days after treatment start of the 3rd patient:
</td></tr>
</table>

  - If there is more than 1 DLT (even prior enrolment of 6 patients), accrual to DL0 stops and accrual starts at the next lower dose level of oleclumab (DL -1).
  - If there is 1 or no DLT out of the 6 patients, the RP2D will be 3000 mg IV 5xQ2W then Q4W.

<u>Dose level -1 (DL-1):</u>
Oleclumab will be administered at the dose of 1500 mg IV Q2W for the first 5 administrations then Q4W in a maximum of 6 patients.
First, 3 patients will be enrolled at DL-1:

- In case of more than 1 DLT (even prior enrolment of 3 patients), accrual to DL-1 stops and accrual starts at the next lower dose level of oleclumab (dose level -2).
- In case of 1 or no DLT out of the 3 patients, 3 additional patients will be enrolled at DL-1. Those 3 additional patients will be enrolled at the earliest 28 days after treatment start of the 3rd patient:
  - If there is more than 1 DLT (even prior enrolment of 6 patients), accrual to DL-1 stops and accrual starts at the next lower dose level of oleclumab (DL-2).
  - If there is 1 or no DLT out of the 6 patients, the RP2D will be 1500 mg IV 5xQ2W then Q4W.

<u>Dose level -2 (DL-2):</u>
Oleclumab will be administered at the dose of 750 mg IV Q2W for the first 5 administrations then Q4W in a maximum of 6 patients.
First, 3 patients will be enrolled at DL-2:

- In case of more than 1 DLT (even prior enrolment of 3 patients), accrual to DL-2 stops and the trial will be closed and the phase II part will not open.
- In case of 1 or no DLT out of the 3 patients, 3 additional patients will be enrolled at DL-2. Those 3 additional patients will be enrolled at the earliest 28 days after treatment start of the 3rd patient:
  - If there is more than 1 DLT (even prior enrolment of 6 patients), accrual to DL-2 stops and the trial will be closed and the phase II part will not open.
  - If there is 1 or no DLT out of the 6 patients, the RP2D will be 750 mg IV 5xQ2W then Q4W.

<u>Recruitment of patients at different dose levels of oleclumab:</u>

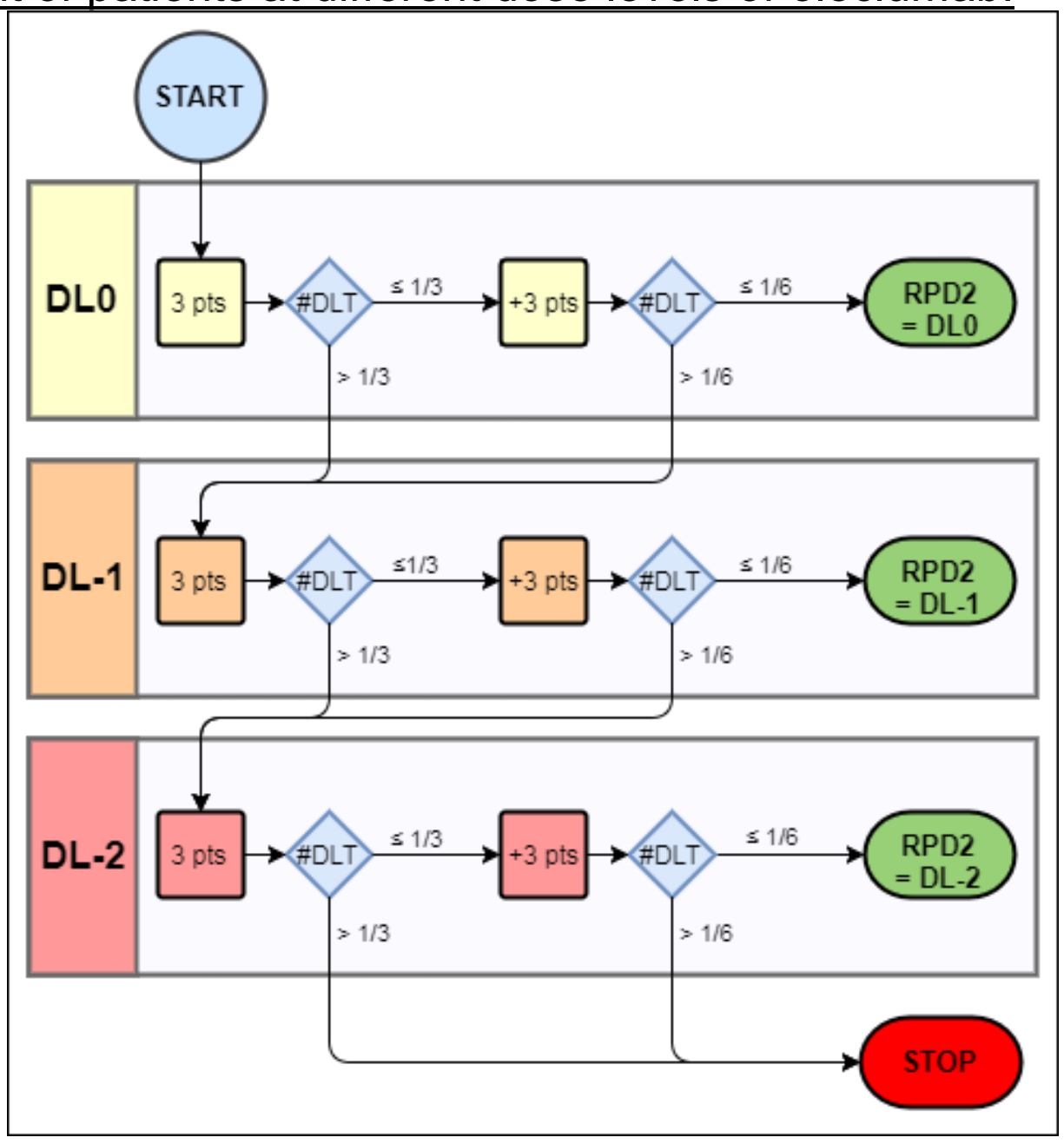

	After the 12 administrations of chemotherapy, the next injection of oleclumab (at week 13 in case of no chemotherapy interruption) will be given according to the current DL. Following administrations of oleclumab after the end of chemotherapy (starting week 17 in case of no chemotherapy interruption), will be given at 3000 mg IV Q4W, independently of the initial DL.<br><br>**PHASE I results**<br>Following the results of the Safety Interim Analysis, the oleclumab dose DL0 = 3000 mg is validated as RP2D for the Phase II part. Additionally, carboplatin dose was reduced from AUC 2 to AUC 1.5.<br><br>**PHASE II**<br>Patients will be treated with paclitaxel, carboplatin, durvalumab ± oleclumab.<br><br>**Paclitaxel and Carboplatin:** Paclitaxel 80 mg/m2 IV once a week + carboplatin (AUC 1.5, calculated according to the Calvert formula) IV once a week (Q1W) for 12 weeks (12 injections of paclitaxel + 12 injections of carboplatin).<br><br>**Durvalumab** will be given at a fixed dose of 1500 mg IV every 4 weeks (Q4W) (same doses than in the phase I part).<br><br>**Oleclumab** will be given (only in arm A) every two weeks for 5 administrations (Q2W) and then every 4 weeks (Q4W) at the fixed dose of 3000 mg IV, as defined in the Phase I part of the trial.<br><br>**PHASE II – Interim analysis results:**<br>No safety concerns were identified for the arm with oleclumab. The pre-specified boundary for futility was crossed and therefore the IDMC recommended stopping the further recruitment for patients in the trial in both arms. Following this recommendation, recruitment stopped on 22nd of June 2021. Patients still under treatment can continue treatment either with oleclumab (arm A) or without (patients in arm B and patients wishing to cross from A into arm B, respectively).
**NON-INVESTIGATIONAL MEDICINAL PRODUCT(S) DOSE/ ROUTE/ REGIMEN**	Chemotherapy pre- and post-medication, gadolinium-based and iodinated contrast agents for imaging.
**ASSESSMENT OF EFFICACY**	The following efficacy endpoints are assessed:<br>• Clinical benefit (CB)<br>• Objective response (OR) assessed as best overall response;<br>• Duration of response (DOR);<br>• Progression-free survival (PFS);<br>• Overall survival (OS);
**ASSESSMENT OF SAFETY**	Clinical and laboratory adverse events (AEs) and serious adverse events (SAEs) will be reported and graded according to the National Cancer Institute Common Terminology Criteria for Adverse Events (NCI-CTCAE) version 5.0.
**STATISTICAL ANALYSES**	Statistical plan: The analysis populations are defined as follows:<br><br>PHASE I: Patients of the phase I belong to the safety population if any amount of study treatment has been administered.<br>PHASE II:<br>• The primary analysis population will be a modified intention to treat (mITT) population, which is defined as all randomized patients, who

	have received at least one dose of treatment (whether or not corresponding to the assigned study treatment).<br>• The safety population is defined as patients who received any amount of the assigned study treatment.<br>• Per protocol, population is defined as all randomized patients who are eligible and have started their allocated treatment. A patient will be considered to be eligible if he/she did not have any major deviations from the patient entry criteria. Eligibility will be assessed by the medical review team.<br>• CBR, ORR, DOR, PFS and OS will be estimated in the mITT population. The rate of patients not evaluable for the primary analysis population will be closely followed. NOTE: CBR, ORR, DOR, PFS and OS will be reported separately for patients of the phase I part.<br>• For safety analyses, only patients in the safety population will be analysed.<br><br>Interim Analysis: The study has at least two planned safety interim analysis for the phase I part and one planned efficacy interim analysis for futility for the phase II part.<br><br>PHASE I: Safety Interim Analysis<br>A safety interim analysis was performed 28 days after the first administration of oleclumab in the 3rd and the 6th patient of the DL0 cohort. Interim safety data was reviewed by the scientific committee. In addition, continuous safety monitoring was performed for the phase I part prior to the safety interim analyses.<br><br>SYNERGY began enrolment on 02/01/2019. As of 04/04/2019, when the last enrolled patient finished the DLT period, 0 out of 6 patients treated experienced a DLT, thus the RPD2 for oleclumab is DL0 – 3000 mg. Outside the DLT period, within a data-freeze of 11/04/2019, the Scientific Committee perceived a high frequency (83.3% - 5 out of 6 patients) of high-grade neutropenia, with 7 events, and one serious AE, corresponding to a grade 3 febrile neutropenia associated with a grade 3 lung infection (16.7% of treated patients) related to the chemotherapy regimen.<br><br>In order to improve the safety of study treatment and also to preserve its dose-density (and consequently its efficacy), the Scientific Committee decided to reduce the carboplatin dose from AUC 2 to AUC 1.5 for the Phase II part of the study, aiming at avoiding delay and dose reductions for both chemotherapy drugs (carboplatin + paclitaxel).<br><br>No other safety signals were perceived by SYNERGY's Scientific Committee so far, therefore no other study treatment modifications are recommended for the purpose of this protocol amendment.<br><br>Long term safety results will be reviewed by an independent data monitoring committee (IDMC) after one year following the beginning of the phase I part. Of note, safety data of ongoing phase II patients will be included in this first IDMC report.<br><br>PHASE II: Efficacy and Safety Interim Analysis<br>An efficacy interim analysis for futility was planned after the enrolment of 68 evaluable patients (34 per treatment arm). Safety and efficacy results were reviewed by the IDMC. The interim analysis was carried out when CBR assessment was possible for all patients, i.e. maximum 24 weeks for the last of the 68 evaluable patients.<br><br>The primary purpose of efficacy interim analysis was to check whether the alternative hypothesis can be rejected prematurely. For planning the interim analysis, a Lan-Demets approach has been used (O'Brien and Fleming spending functions for β error). On the z scale, the stopping boundary was

	0.074 (ie, at interim, in case the z test statistic is below 0.074, the trial will stop early for futility).<br><br>In the interim analysis, the pre-specified boundary for futility was crossed (z-value = -0.2574 (<0.074); p-value=0.6914 (>0.464; one-sided Fisher's exact test) suggesting the futility of oleclumab addition in the treatment. No safety concerns were identified for the arm with oleclumab. The IDMC recommended stopping the further recruitment for patients in the trial in both arms. Following this recommendation, recruitment stopped on 22nd of June 2021. Patients still under the study treatment can continue treatment either with oleclumab (arm A) or without (patients in arm B and patients wishing to cross from A into arm B, respectively).
**STRATIFICATION FACTORS**	**Only for Phase II:** Patients will be randomized 1:1 between chemotherapy/durvalumab/oleclumab and chemotherapy/durvalumab.<br><br>The stratification factors are CD73 expression, PD-L1 status and the trial site.
**TRANSLATIONAL RESEARCH(ES)**	The translational research proposal within this clinical trial aims to understand the host/tumour immune interaction before and after treatment to identify potential predictive biomarker of response to treatment as well as mechanisms underlying resistance to immunotherapy. This project aims also to improve our understanding of mechanisms of action such as apoptosis induction, reduction of proliferation, immune pathways activation and oncogenic pathways inhibition underlying the synergistic effects of the combination of paclitaxel, carboplatin and durvalumab with oleclumab.<br><br>The objectives of the translational research project are:<br>1) To identify potential predictive immune biomarkers by performing a widespread histological and molecular profiling of the pre- and post- study treatment peripheral blood and tumour tissues using IHC and other histological methods as immunofluorescence as well as flow cytometry and chromatography;<br>2) To identify genomic alterations associated with treatment efficacy by characterizing pre- and post- study treatment tissue samples using whole-genome and RNA-sequencing;<br>3) To assess the role of the tumour microenvironment in the treatment efficacy by applying single-cell level spatial transcriptomics;<br>4) To provide a readout of the T-cell receptor repertoire present in tumour-infiltrating T-cells using single-cell RNA sequencing;<br>5) To evaluate the role of plasma ctDNA in monitoring response/resistance to the study treatment.<br><br>Due to the fast evolution within these fields, the actual analytical methodology will only be specified and decided upon at the time these analyses will start.<br><br>Several tissue samples (archived primary tumour, biopsies of metastatic lesions) and blood samples collection will be performed during the study.

	Screening	Week1	During week 3	1st immuno-therapy visit	Week 25	First Immune related toxicities	First Disease Progression
Breast primary tumour tissue (FFPE block or 20 unstained slides freshly cut for the purposes of the study)	X						

<table>
<tr>
<td></td>
<td>
<table>
<tr><td>Metastatic/recurrent lesion biopsy (1 FFPE + 1 frozen [± 2 fresh] tissues cores)</td><td>X</td><td></td><td>X</td><td></td><td></td><td></td><td>X
(optional)</td></tr>
<tr><td>Whole Blood (1X5mL)</td><td></td><td>X</td><td></td><td></td><td></td><td></td><td></td></tr>
<tr><td>Blood for plasma (3X9mL)</td><td></td><td>X</td><td>X</td><td>X</td><td>X</td><td>X</td><td>X</td></tr>
<tr><td>Blood for serum (1x9mL)</td><td></td><td>X</td><td>X</td><td>X</td><td>X</td><td>X</td><td>X</td></tr>
<tr><td>Blood for PBMCs (1x8mL)</td><td></td><td>X</td><td>X</td><td></td><td>X</td><td>X</td><td>X</td></tr>
<tr><td>Blood for mRNA isolation (1x2.5mL)</td><td></td><td>X</td><td>X</td><td>X</td><td>X</td><td>X</td><td>X</td></tr>
</table>
Stratification factors (only for phase II): PD-L1 and CD73 expression will be assessed using immunohistochemistry (IHC) on the screening FFPE metastatic/recurrent tumour tissue lesion sample (or on the archived FFPE primary tumour sample in case the metastatic/recurrent tumour lesion sample is not evaluable for PD-L1 or CD73) by a central laboratory prior to randomization for stratification purposes. For the screening, metastatic and archived primary tissue samples must be sent to the central laboratory. In case of de novo metastatic disease, a sample of the primary tumour lesion is acceptable for stratification.
Patients whose tumour tissue is not evaluable for PD-L1 and CD73 will not be eligible. All sites and study team staffs will be blinded to PD-L1 and CD73 status.
</td>
</tr>
<tr>
<td>LENGTH OF THE STUDY</td>
<td>
<u>Expected Accrual Time</u>
PHASE I: It is estimated that 2 patients per month will be enrolled leading to an accrual time between 3 and 9 months.
PHASE II: It is estimated that 10 patients per month will enter the treatment phase leading to an accrual time of 15 months.
In total, the accrual time will be between 18 and 24 months.
<u>Expected Duration of the Trial</u>
Approximate treatment duration for a patient: 12 months.
Expected duration of the all study: approximately 50 months (taken into account accrual, treatment period and follow-up), assuming that the phase II starts approximately 1 month after the safety interim analysis of the phase I.
</td>
</tr>
<tr>
<td>END OF STUDY</td>
<td>
The end of study is declared when all the following criteria have been met:

Approximately 24 months after the date the last patient has been randomized.
Until every patient reached PD or died or withdrew consent or is lost to follow-up.
The database has been fully cleaned and frozen for all analyses.

</td>
</tr>
</table>

1. BACKGROUND AND SCIENTIFIC RATIONALE

1.1. Background

1.1.1. Triple-negative Breast Cancer

Triple-negative breast cancers (TNBC) are defined as tumours that lack expression of estrogen receptor (ER), progesterone receptor (PR) and human epidermal growth factor receptor 2 (HER2). They account for about 15% of all invasive breast cancers (BC), usually have a high histologic grade and are associated with a worse outcome compared to other malignant breast tumours[1]. Patients with metastatic TNBC have a poor clinical outcome, generally with rapid progression and a median overall survival (OS) of less than 1.5 years[2].

1.1.2. Current Treatment Options

Chemotherapy is currently the mainstay of systemic treatment for TNBC. There is no preferred standard regimen of chemotherapy for TNBC and the chemotherapeutic approach is similar as it is for other subtypes of metastatic breast cancer (MBC)[1]. Neoadjuvant chemotherapy is considered standard of care for patients with early-stage TNBC. A minority of TNBC are highly sensitive to chemotherapy and patients experiencing a pathological complete response (pCR) in early disease setting have an excellent outcome[1]. However, once there is metastatic disease, relapses and disease progression inevitably occur even after initial responses to chemotherapy. Depending on the chemotherapeutic agents that have been used in (neo)adjuvant setting and the time between initial treatment and disease relapse, different treatment options are available in the metastatic setting. Anthracyclines, cyclophosphamide, taxanes and platinum agents are the most commonly used drugs in both early and advanced disease.

1.1.2.1. *Anthracyclines*

Anthracyclines are one of the most effective drugs for BC. A meta-analysis comparing single-agent anthracyclines to single-agent taxanes as first-line therapy of patients with MBC has found the same response rates (33 and 38%, respectively) but a better progression-free survival (PFS) for anthracyclines (hazard ratio [HR] for taxanes compared with anthracyclines 1.19; 95% confidence interval [CI] 1.04-1.36)[3]. However, the use of anthracyclines for metastatic disease is limited by the cumulative dose-dependent risk of cardiac toxicity.

1.1.2.2. *Taxanes*

Especially in patients pre-treated with anthracyclines who have reached the maximum anthracycline cumulative dose, taxane-based regimens are most commonly used as first-line therapy for patients with MBC[4]. The role of paclitaxel in the treatment of BC is well established. Reported response rates for paclitaxel administered as single agent to patients with MBC are between 25 and 40%[3,5–7]. Weekly administration of paclitaxel has been shown to be more effective in terms of response rate than tri-weekly regimen[8].

1.1.2.3. *Platinum Salts*

Platinum salts are highly effective in BC patients harbouring a germline BRCA mutation. In a phase II study, cisplatin response rate in BRCA-related MBC was 80%[9]. The TNT study compared docetaxel to carboplatin in unselected advanced TNBC patients. In the whole cohort, response rates and PFS were the same for docetaxel (35.6% and 4.5 months) and for carboplatin (31.4% and 3.1 months; p-value=0.44 and 0.29, respectively). However, among patients with germline BRCA mutations, the objective response rate with carboplatin was higher (68%) than with docetaxel (33%; p-value=0.03)[10]. Three major trials examined the use

of carboplatin in the neoadjuvant setting of TNBC irrespective of BRCA mutations: GeparSixto (18 weeks of weekly paclitaxel + weekly non-pegylated liposomal doxorubicin + bevacizumab once every three weeks +/- weekly carboplatin AUC 1.5), CALGB/Alliance 40603 (12 weeks of paclitaxel weekly followed by doxorubicin + cyclophosphamide every two weeks for 4 cycles + carboplatin AUC 6 once every 3 weeks for four cycles and/or bevacizumab once every 2 weeks for nine cycles) and Brightness (12 weeks of weekly paclitaxel + carboplatin AUC 6 every 3 weeks + veliparib vs paclitaxel + carboplatin vs paclitaxel followed by doxorubicin and cyclophosphamide)[11–13]. All three trials reported an improvement in the pCR rate with the addition of carboplatin. In GeparSixto, the addition of carboplatin improved pCR rates (breast/axilla) from 36.9% to 53.2% (p=0.005). CALGB/Alliance 40603 reported an increase in pCR rates (breast/axilla) from 41% to 54% (p=0.0029). In Brightness, the pCR rates for the two carboplatin-containing arms were 53.2% and 57.5% (p-value=0.36) as compared to a statistically significant lower rate of 31% in the standard arm without carboplatin and veliparib (p-value<0.001). All trials were not powered to show carboplatin effects on survival. Long-term outcomes data for GeparSixto and CALGB/Alliance 40603 were divergent: GeparSixto demonstrated improved PFS with the addition of carboplatin (HR 0.56; 95% CI 0.33-0.96; median follow-up 35 months) while CALGB/Alliance 40603 did not report improved event-free survival with the addition of carboplatin (HR 0.84; 95% CI 0.58-1.22)[14,15]. Even though the exact role of platinum salts in the treatment for TNBC still needs to be defined, carboplatin is considered as an attractive treatment choice for unselected advanced TNBC[4].

1.1.2.4. *Combination Treatments*

For the treatment of advanced BC, both combination and sequential single agent chemotherapy are reasonable options[4]. Sequential monotherapy is usually the preferred choice. Combination treatment is considered standard of care for patients with rapid disease progression, life threatening visceral metastases or need for rapid symptom and/or disease control[4]. A Cochrane meta-analysis published in 2013 reported the same OS for combination and sequential single agent chemotherapy with higher response but also toxicity rates for the combination treatment[16]. The question, whether single agent chemotherapy or combination chemotherapy should be applied for the treatment of TNBC, is particularly important as these tumours tend to be more aggressive.

1.1.3. Targeted Treatment Approaches

Targeted treatment options are very limited in TNBC. Bevacizumab is approved in some countries for TNBC. The enzyme poly(adenosine diphosphate-ribose) polymerase (PARP) is a successful target for treatment of patients with BRCA mutations. Recently, the OlympiAD and EMBRACA trials, comparing PARP inhibitors (olaparib and talazoparib, respectively) versus single agent chemotherapy of physician's choice in patients with metastatic hormone receptor positive (HR+) or TNBC patients with a germline BRCA mutation, reported an improvement in PFS in favour of PARP inhibitors[17,18]. Based on the results of the OlympiAD and EMBRACA trials, olaparib and talozaparib have recently been approved by the FDA for MBC patients harbouring germline BRCA mutations.

1.1.4. Immunotherapy

Immunotherapy with immune checkpoint blockade (ICB) agents represents one of the most promising advances in oncology in the past few years and demonstrated impressive antitumour activity and durable clinical benefit in diverse advanced malignancies[19–21]. This recent breakthroughs of immunotherapy coupled with the prognostic influence of tumour-infiltrating lymphocytes (TILs) in BC and the expression of PD-L1 in a non-negligible proportion of BC (mainly TNBC) has led to the initiation of several clinical trials testing ICB in BC[22].

The programmed cell death 1 (PD-1) pathway plays a critical role in regulating the immune response. PD-1, an inhibitory immune checkpoint receptor can be found on activated T cells, B cells, natural killer cells, activated monocytes, dendritic cells and a subset of thymocytes[23]. Interaction between PD-1 and its programmed death-ligand 1 (PD-L1, B7-H1) inhibits activated T-cells and downregulates T cell response by inhibiting T cell receptor (TCR)-mediated activation and CD28 co-stimulation[24,25]. PD-L1 is expressed in primary and metastatic breast tumours and more frequently in TNBC[26–28].

In metastatic TNBC, single ICB demonstrated clinical activity in a limited subset of patients in early phases trials. The KEYNOTE-012 trial was a multicenter, phase Ib study of single agent pembrolizumab, an anti-PD-1-antibody, in patients with advanced PD-L1-positive solid tumours. Thirty-two patients with advanced TNBC irrespective of prior therapy were enrolled. The overall response rate (ORR) in this cohort was 18.5%; the median duration of response was not yet reached at the time point of the final publication in May 2016. Overall, toxicities were mild and did not differ from the ones observed in the other tumour cohorts[29]. Atezolizumab, an anti-PD-L1-antibody was tested in a multicenter phase I study in TNBC patients. With 112 evaluable patients, it is so far, the largest immunotherapy trial in TNBC that reported results at AACR 2017. Enrolment was initially limited to patients with PD-L1 ≥ 5% of tumour-infiltrating immune cells but then opened to patients regardless of PD-L1 status. ORRs in first- and second-line were 26% and 11%, respectively. Median overall survival of the responders was not reached with no deaths as data cut-off[30].

Synergistic combinations are warranted to improve the response rates and clinical benefit from immune treatments in BC. Several chemotherapeutic agents were demonstrated to induce immunogenic cell death (ICD). This process increases the immunogenicity of neoplastic cells and primes the immune system, by stimulating innate immune effectors and by inducing cytotoxic T cell responses[31].

Trials combining ICB with standard chemotherapy are ongoing in advanced TNBC:

1) The combination of pembrolizumab + eribulin, a non-taxane microtubule inhibitor, was tested in a phase Ib/II trial in patients with advanced TNBC treated with ≤ 2 prior lines of chemotherapy for metastatic disease. Updated findings of this trial including 106 patients that were evaluable for efficacy were presented at the SABCS 2017. The ORR with the combination for untreated patients with metastatic TNBC (n=65) was 29.2% (95% CI, 18.6%-41.8%). In a cohort of patients pre-treated with 1 to 2 therapies (n=41), the ORR was 22.0% (95% CI, 10.6%-37.6%). Across all treatment arms, there were 3 complete responses (2.8%). The clinical benefit rate (CBR; complete response, partial response, durable stable disease [duration greater than or equal to 24 weeks]) was 36.8% and the median duration of response (DOR) was 8.3 months. Clinical activity was observed regardless of PD-L1 expression. In PD-L1 positive cases (n=49), ORR was 30.6% with 2 CR and 22.4% with 1 CR in PD-L1-negative cases (n=49)[32]. The overall survival data were presented at ASCO 2020. The median OS was 21 months (95% CI 8.3-29.0 months), 15.2 (95% CI 12.8-19.4 months), 14.0 (95% CI 11-19.4 months), and 15.5 months (95% CI 12.4-18.7 months) in the naive treatment population (n=29) with PDL1≥1%, naïve treatment (n=31) with PDL1<1%, in pre-treated with 1 to 2 therapies PDL1≥1% (n=45), in pre-treated with PDL1<1% (n=44) respectively[33].
2) Results of the phase Ib trial of atezolizumab + nab-paclitaxel in advanced TNBC with ≤ 3 prior lines of therapy for the metastatic setting were presented at ASCO 2016. The ORR in the whole cohort (n=32) was 38%, in the first-line cohort (n=13) 46%, in the second-line cohort (n=9) 22% and in the third-line (n=10) 40%. No dose limiting toxicities (DLTs) or related deaths occurred[34].

Phase III trials with chemotherapy ± ICB as first-line treatment for metastatic TNBC are ongoing (KEYNOTE-355 (NCT02819518) and IMpassion130 (NCT02425891)).

The IMpassion130 evaluates the combination of atezolizumab with nab-paclitaxel compared to nab-paclitaxel plus placebo. It is the first positive phase III trial showing encouraging survival

benefit in patients with PD-L1+ advanced TNBC and thus positions ICB as a valid option for this BC subtype[35,36]. Based on these results, the FDA and EMA approved atezolizumab in combination with nab-paclitaxel for adult patients with unresectable locally advanced or metastatic TNBC whose tumours express PD-L1 in 2019.

KEYNOTE-355 evaluates the combination of the chemotherapy (nab-paclitaxel or paclitaxel or gemcitabine-carboplatin) with pembrolizumab versus placebo. The patients were stratified according to the combined positive score (CPS). The study has met its primary endpoint in the cohort CPS≥10, with mPFS=9.7 months in the pembrolizumab arm and 5.6 months in placebo-chemotherapy (HR 0.65 95% CI 0.49–0.86, one-sided p=0·0012). Based on these results, the FDA gave an accelerated approval on December 2020 to pembrolizumab for previously untreated locally recurrent unresectable TNBC or metastatic TNBC with CPS≥10[37].

1.1.4.1. *Targeting PD-L1 with durvalumab (MEDI4736)*

Durvalumab is a human immunoglobulin G1 kappa (IgG1κ) monoclonal antibody that targets PD-L1. Durvalumab blocks the interaction of PD-L1 (but not PD-L2) with PD-1 on T cells and CD80+ immune cells and is engineered to reduce antibody-dependent cell-mediated cytotoxicity (ADCC).

Safety data are available from several trials evaluating durvalumab in monotherapy in solid tumours and in combination with the anti-CTLA-4, tremelimumab[38] or with gefitinib, or osimertinib and also with the anti-CD73 MEDI9447 (oleclumab). For the combination with MEDI9447, no DLTs were reported[39].

Preliminary safety data are available from a placebo-controlled phase II trial examining the combination of durvalumab plus nab-paclitaxel followed by durvalumab plus epirubicin and cyclophosphamide as neoadjuvant treatment in TNBC (NCT02685059). Initial results of the 31 first treated patients have been reported at ASCO 2017. So far, the addition of durvalumab did not result in increased toxicity[40]. Preliminary efficacy data were presented at ASCO 2018 and demonstrated that durvalumab in combination with chemotherapy increased pCR rate to 53%, compared with 44% for chemotherapy alone[41]. Although not statistically significant, these early results are encouraging and suggest that ICB could improve the benefit of neoadjuvant therapy in TNBC.

In a phase II trial, SAFIR02-BREAST IMMUNO trial, the durvalumab was evaluated in maintenance after induction chemotherapy in metastatic breast cancer without targetable molecular alteration. The exploratory analysis for TNBC (n=82) has shown the improvement of the OS HR=0.54, 95% CI 0.30-0.97, p=0.0377[42]. Despite the small number of subjects, these data support the idea of applying maintenance immunotherapy after the induction treatment in patients suffering from metastatic TNBC.

Durvalumab has been granted accelerated FDA approval for the treatment of patients with locally advanced or metastatic urothelial carcinoma who have disease progression during or following platinum-containing chemotherapy or who have disease progression within 12 months of neoadjuvant or adjuvant treatment with platinum-containing chemotherapy. Approval is based on one single arm trial of 191 patients with locally advanced or metastatic urothelial carcinoma whose disease progressed after prior platinum-containing chemotherapy (NCT01693562). The confirmed ORR in 191 patients was 17.8% (95% CI 12.7-24.0)[43]. To date durvalumab has been given to more than 6000 patients along ongoing studies either as monotherapy or in combination with other anti-cancer agents.

1.1.4.2. *Targeting CD73 with oleclumab (MEDI9447)*

CD73 is an ectoenzyme catalysing the conversion of adenosine monophosphate (AMP) to adenosine and organic phosphate that can be found on the surface of tumour cells, stromal cells such as endothelial cells and certain leukocytes[44]. Extracellular adenosine promotes

tumour cell metastasis, angiogenesis and has multiple immunosuppressive functions[45]. In preclinical tumour models, targeted blockade of CD73 reduced tumour growth, metastases and increased the number of tumour-infiltrating $CD8^+$ effector cells and activated macrophages[46]. In BC, CD73 expression is associated with poor prognosis in the TNBC subtype[47,48]. Patients with high levels of CD73 and low levels of tumour-infiltrating leukocytes in the primary tumour had the worst clinical outcome in a large cohort of TNBC from a phase III clinical trial that compared anthracycline alone to anthracycline-taxane chemotherapy[47]. Furthermore, treatment with anthracyclines, paclitaxel and platinum induced up-regulation of CD73 in human BC cells[48,49]. Targeting the CD73 pathway to relieve the adenosine-mediated immunosuppression seems a promising therapeutic strategy in TNBC.

In mouse models, the potential of adenosine targeting treatment was enhanced when combined with other immunomodulatory treatment as ICB[50]. The combination of ICB targeting PD-1 with an anti-CD73, MEDI9447(oleclumab) already showed synergistic activity in preclinical models[51].

Phase I trials with anti-CD73 compounds or antagonists of the adenosine A2A receptor +/- ICB in advanced solid tumours (including TNBC) are ongoing (NCT02503774; NCT02403193; NCT02655822). Interim safety data on 113 patients and efficacy data for 96 patients (of whom 31 with TNBC) enrolled in a phase I/Ib trial of CPI-444, an A2A antagonist, +/- atezolizumab (NCT02655822) in advanced solid tumours were presented at AACR 2017. Fifty-six percent were resistant or refractory to prior treatment with anti-PD-L1 antibodies. Disease control in TNBC was achieved in 41% of those receiving CPI-444 as single agent (n=17) and in 21% of those receiving the combination (n=14) for an overall disease control rate of 32% in 31 patients. CPI-444 was well tolerated with no grade 3 or 4 adverse events with single agent CPI-444[52].

In this trial, oleclumab (MEDI9447) a human immunoglobulin G1 lambda (IgG1λ) monoclonal antibody (mAb) that selectively binds to and inhibits the ectonucleotidase activity of CD73 will be evaluated to inhibit the production of adenosine and organic phosphate from AMP by CD73.

1.2. Rationale

1.2.1. Choosing TNBC as Target Disease

TNBC is the most aggressive BC subtype with a poor prognosis for metastatic patients (median OS between 12-15 months) and very limited treatment options. Early phases immunotherapy trials in BC already demonstrated encouraging results with ICB in monotherapy and in combination with chemotherapy particularly in this TNBC subtype in the neoadjuvant and metastatic setting[22]. Indeed, triple-negative tumours are considered more immunogenic as expression of immune-related genes and the presence of TIL in primary tumours were associated with a better clinical outcome in TNBC[53,54]. Higher levels of PD-L1 were also observed in this BC subtype[26–28].

However, the efficacy of immunotherapy in TNBC is limited with lower ORR than in other cancer types. This trial aims to improve the response and the clinical benefit of an immunotherapeutical approach in patients with advanced TNBC.

1.2.2. Choosing Paclitaxel and Carboplatin as Chemotherapeutical Backbone

Taxane-based regimens are considered a standard of care in first-line therapy for patients with MBC. However, the median PFS is relatively short. Platinum-salts are at-least as active as taxanes in metastatic TNBC. In the neoadjuvant setting of TNBC the addition of platinum salts to standard therapies increased pCR rates. Furthermore, pre-clinical work demonstrated that treatment with carboplatin and paclitaxel increased the expression of CD73 and PD-L1 by TNBC cells[49]. A platinum backbone is increasingly used in the standard of care arm of phase III trials enrolling metastatic patients with TNBC. Chemotherapeutic agents have also been

demonstrated to promote immune responses by inducing ICD and releasing tumour antigens[55]. In this trial, we evaluate the combination of paclitaxel + carboplatin together with an immunotherapeutic approach including ICB.

1.2.3. Adding oleclumab, an anti-CD73 agent to durvalumab, a PD-L1 inhibitor

As CD73 expression is associated with a poor prognosis and a reduced anti-tumour immunity in TNBC, targeting CD73 could be a promising strategy to reprogram the tumour microenvironment in this BC subtype[47]. By decreasing the adenosine-mediated immunosuppression in the tumour microenvironment, anti-CD73 treatment enhanced the potential of other immunomodulatory treatments such as ICB. This synergistic activity has already been demonstrated in preclinical models with the combination of either anti-CD73 or adenosine receptors antagonists with anti-PD1 or anti-CTLA-4 antibodies[50,56–58]. In this trial, we investigate the role of the addition of oleclumab, an anti-CD73 to the combination of chemotherapy and ICB with durvalumab.

1.3. Potential Risks and Benefits

The outcome of patients with metastatic TNBC is poor with the majority dying within 2 years after the diagnosis. A minor subset of patients with metastatic TNBC derives long-term benefit from ICB. By adding CD73-targeting oleclumab, we aim to decrease adenosine immunosuppressive effects in the tumour microenvironment and to increase the proportion of patients who might respond to the combination of chemotherapy with PD-1/PD-L1 blockade.

Durvalumab, alone or in combination with different agents including oleclumab and nab-paclitaxel, epirubicin and cyclophosphamide, has been evaluated in different phase I/II trials. The safety profile of durvalumab in monotherapy and in combination with other anticancer agents is consistent with the pharmacology of the target and other agents in the immune checkpoint inhibitor class. Most adverse drug reactions (ADRs) seen with ICB agents are caused by non-specific activation of the immune system and can affect almost any organ. These immune-related adverse events (irAEs) observed with ICB include gastrointestinal AEs such as colitis and diarrhoea, pancreatitis, pneumonitis/interstitial lung disease (ILD), renal AEs such as nephritis and increases in serum creatinine, hepatic AEs such as hepatitis and liver enzyme elevations, dermatitis/rash, myocarditis, myositis/polymyositis, endocrinopathies such as hypo- and hyper-thyroidism, hypophysitis, adrenal insufficiency and type I diabetes mellitus, other rare or less frequent inflammatory events include neurotoxicities, infusion-related reactions and infections/serious infections. The majority of these irAEs are mild to moderate and do not require treatment discontinuation but severe to occasionally life-threatening irAEs have been reported.

In monotherapy clinical studies AEs (all grades) reported very commonly with durvalumab (≥ 10% of patients) are fatigue, nausea, decreased appetite, dyspnoea, cough, constipation, diarrhoea, vomiting, back pain, pyrexia, asthenia, anaemia, arthralgia, peripheral oedema, headache, rash, and pruritus. Approximately 10% of patients experienced an AE that resulted in permanent discontinuation of durvalumab and approximately 6% of patients experienced a serious adverse event (SAE) that was considered to be related to durvalumab by the study investigator. A detailed summary of durvalumab monotherapy AE data can be found in the current version of the durvalumab Investigator's Brochure (IB).

The combination of durvalumab with chemotherapy (nab-paclitaxel, epirubicin and cyclophosphamide) in the neoadjuvant treatment of TNBC patients is being explored in a randomized, placebo controlled phase II trial. So far, the addition of durvalumab did not result in an increased toxicity. The combination of durvalumab with oleclumab has also been evaluated and generally well tolerated with no reported DLTs.

A total of 667 subjects have been treated with oleclumab across 12 clinical studies as of 09Jun2021. Oleclumab was administered as a single agent in 42 adult subjects with selected advanced solid tumours. In this monotherapy group, the most frequently reported treatment related treatment emergent adverse events were fatigue (16.7%), nausea (9.5%), and anaemia (9.5%). The combination of oleclumab and durvalumab has been administered in 295 subjects with the most common treatment related treatment emergent adverse events being fatigue (11.9%), pruritus (7.5%), diarrhoea (6.4%), rash (6.1%), and asthenia (5.4%).. In combination with durvalumab, TEAE of special interest for oleclumab was microvascular capillary permeability (16.9%) with patients presenting peripheral oedema or pleural effusion; followed by thromboembolic event (7.5%) including pulmonary embolism (3.7%). There are no identified risks for oleclumab. The important potential risks include infusion-related reactions, anaphylaxis and serious allergic reactions, immune complex disease, increased microvascular permeability, and thrombosis.

A detailed summary of oleclumab monotherapy and combination AE data can be found in the current version of the oleclumab IB.

The risk of overlapping toxicity between durvalumab/oleclumab and paclitaxel/carboplatin is thought to be minimal. Part 1 of the study consists of a phase I to evaluate the safety and feasibility of this double treatment combination and to define the recommended phase II dose (RP2D) of oleclumab in combination with paclitaxel, carboplatin and durvalumab.

Well-known serious risks associated with paclitaxel include bone marrow suppression (neutropenia, anaemia, and thrombocytopenia), elevation of liver enzymes, alopecia, peripheral neuropathy, infection, and pneumonitis. Well-known serious risks of carboplatin include bone marrow suppression (neutropenia, anaemia, and thrombocytopenia), nausea and vomiting, and hypersensitivity.

Given the poor prognosis for metastatic TNBC patients and the lack of targeted agents, this population is considered appropriate for trials of novel therapeutic candidates. The benefit-risk ratio for durvalumab ± oleclumab in combination with paclitaxel + carboplatin is expected to be acceptable in this setting.

2. OBJECTIVES AND ENDPOINTS OF THE STUDY

2.1. Objectives

2.1.1. Primary objectives

PHASE I: To confirm the **safety** of oleclumab in combination with paclitaxel, carboplatin and durvalumab in previously untreated, locally recurrent inoperable or metastatic TNBC patients.

PHASE II: To evaluate the **clinical benefit** of oleclumab in combination with paclitaxel, carboplatin and durvalumab in previously untreated, locally recurrent inoperable or metastatic TNBC patients by comparing the clinical benefit rate (CBR) at 24 weeks from the 1st dose of study drug administration between patients treated with or without the anti-CD73 antibody oleclumab.

2.1.2. Secondary objectives

PHASE I: To determine the **recommended phase II dose** (RP2D) of oleclumab in combination with paclitaxel, carboplatin and durvalumab in previously untreated, locally recurrent inoperable or metastatic TNBC patients.

PHASE II:

1) To evaluate the **efficacy** of oleclumab in combination with paclitaxel, carboplatin and durvalumab in previously untreated, locally recurrent inoperable or metastatic TNBC patients assessed by comparing the objective response rate (ORR; complete response [CR] + partial response [PR]) and the duration of response (DOR) between patients treated with or without the anti-CD73 antibody oleclumab.
2) To evaluate the **survival benefit** of oleclumab in combination with paclitaxel, carboplatin and durvalumab in previously untreated, locally recurrent inoperable or metastatic TNBC patients by comparing the progression-free survival (PFS) and overall survival (OS) between patients treated with or without the anti-CD73 antibody oleclumab.
3) To evaluate the **safety and feasibility** of oleclumab in combination with paclitaxel, carboplatin and durvalumab in previously untreated, locally recurrent inoperable or metastatic TNBC patients.
4) To evaluate the efficacy, clinical and survival benefits of oleclumab in combination with paclitaxel, carboplatin and durvalumab in previously untreated locally recurrent inoperable or metastatic TNBC patients **according to PD-L1 and CD73 expression**.

2.1.3. Exploratory objectives

PHASE I/II:

1) To compare ORR by RECIST v1.1 and iRECIST during the first 24 weeks from the 1st dose of study drug administration.
2) To compare PFS by RECIST v1.1 and iRECIST from date of 1st dose of study drug administration

2.2. Endpoints

2.2.1. Primary endpoints

PHASE I: Safety and tolerability will be assessed by monitoring frequency, duration and severity of adverse events (AEs).

PHASE II: Clinical benefit (CB). CB is defined as a patient who achieved CR or PR or demonstrated SD at 24 weeks from the 1st dose of study drug administration based on RECIST v1.1.

2.2.2. Secondary endpoints

PHASE I: Safety and tolerability will be assessed by monitoring frequency, duration and severity of adverse events (AEs) including Dose limiting toxicities (DLTs) as defined per protocol (*see section 7.2. Dose-limiting Toxicities of oleclumab*).

PHASE II:

1) Objective Response. OR is defined as a patient (in the intent-to treat population) who achieved a CR or PR as best overall response (BOR) based on RECIST v1.1.
2) Duration of Response. DOR is defined as the time from documentation of first tumour response to disease progression based on RECIST v1.1.
3) Progression Free Survival. PFS is defined as the time from 1st study drug administration to the first documented disease progression based on RECIST v1.1 or death due to any cause, whichever occurs first. (Subjects who are alive and progression free at the time of analysis will be censored at the time-point of their last tumour assessment by imaging.)

4) Overall Survival. OS is defined as the time from 1st study drug administration to death due to any cause. (Subject without documented death at the time of the analysis will be censored at the date of the last follow-up.)
5) Frequency, duration and severity of AEs based on CTCAE 5.0.

2.2.3. Exploratory endpoints

PHASE I/II:

1) Response assessment based on RECIST v1.1 versus iRECIST during the first 24 weeks from the 1st dose of study drug administration.
2) PFS assessment based on RECIST v1.1 versus iRECIST

3. STUDY DESIGN

The trial consists of two parts.

3.1. Phase I

Part 1 is a phase I trial examining the combination of paclitaxel, carboplatin, durvalumab and oleclumab in order to define the recommended phase II dose (RP2D) of oleclumab in this treatment combination.

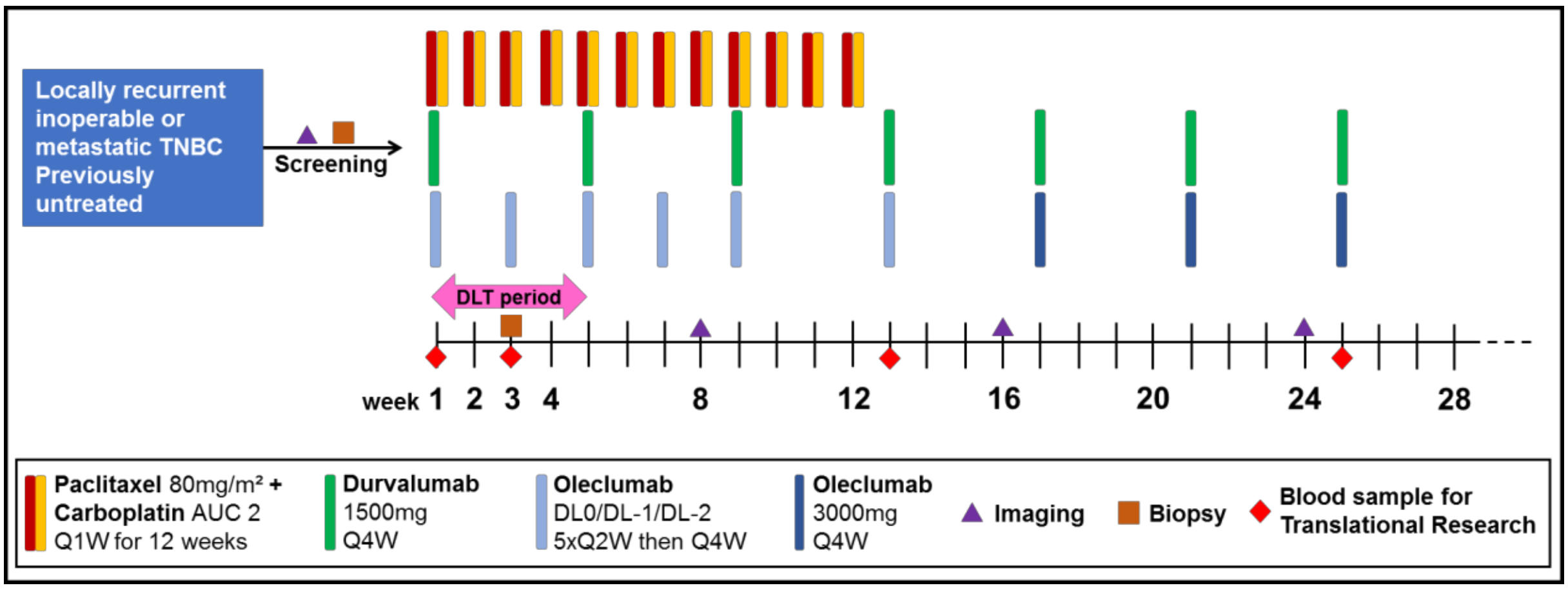

Figure 1 Phase I schema

Patients are treated with paclitaxel, carboplatin, durvalumab and oleclumab.

3.1.1. Paclitaxel and Carboplatin

Paclitaxel 80 mg/m^2 IV once a week + carboplatin AUC 2 (Area under the curve 2, calculated according to the Calvert formula)[59] IV once a week (Q1W) for 12 weeks (= 12 administrations of paclitaxel + 12 administrations of carboplatin).

3.1.2. Durvalumab

Durvalumab dose was determined by a population PK model developed using monotherapy data from a phase I study (study 1108; N=292; doses= 0.1 to 10 mg/kg Q2W or 15 mg/kg Q3W; solid tumours). Population PK analysis indicated only minor impact of body weight on the PK of durvalumab (coefficient of ≤0.5) as observed with other monoclonal antibodies[60,61]. As fixed and body size-based dosing perform similarly, a fixed dose of 1500 mg every 4 weeks (Q4W) (equivalent to 20 mg/kg Q4W based on average body weight of 75 kg) is used in the current study.

3.1.3. Oleclumab

A previous phase I study determined a recommended dose of oleclumab in combination with durvalumab for a phase II study at 3000 mg. The phase I part of this trial will evaluate the safety and feasibility of this recommended dose of oleclumab in combination with durvalumab and chemotherapy. PK models demonstrated that body weight-based and fixed dosing regimens perform similarly. However, due to the ease of use and reduced potential dosing errors, a fixed dosing approach is preferable and is used in the current study.

Oleclumab is given IV every 2 weeks (Q2W) for the first 5 administrations then every 4 weeks (Q4W). The dose of oleclumab in combination with chemotherapy differs according to the respective dose level (DL). After the 12 administrations of chemotherapy, oleclumab will be given in combination with durvalumab at a maintenance dose of 3000 mg every 4 weeks (Q4W).

The period for DLT evaluation is defined as the time from receiving the first dose of oleclumab until the planned administration of the third dose; this corresponds to 28 days after receiving the first dose of oleclumab in case no treatment interruption occurred (*for DLT definition, please see section 7.2. Dose-limiting Toxicities of oleclumab*).

Subjects who do not remain in the study for at least 28 days after the first oleclumab administration for reasons other than DLT will be considered non-evaluable for DLT assessment and will be replaced with another subject at the same dose level.

Patients who develop a DLT will stop study treatment permanently.

<u>Dose level 0 (DL0):</u>
Oleclumab will be administered at the dose of 3000 mg IV every two weeks (Q2W) for the first 5 administrations then every 4 weeks (Q4W) in a maximum of 6 patients.
First, 3 patients will be enrolled at DL0:

- In case of more than 1 DLT (even prior enrolment of 3 patients), accrual to DL0 stops and accrual starts at the next lower dose level of oleclumab (dose level -1).
- In case of 1 or no DLT out of the 3 patients, 3 additional patients will be enrolled at DL0. Those 3 additional patients will be enrolled at the earliest 28 days after treatment start of the 3rd patient:
    - If there is more than 1 DLT (even prior enrolment of 6 patients), accrual to DL0 stops and accrual starts at the next lower dose level of oleclumab (DL -1).
    - If there is 1 or no DLT out of the 6 patients, the RP2D will be 3000 mg IV 5xQ2W then Q4W.

<u>Dose level -1 (DL-1):</u>
Oleclumab will be administered at the dose of 1500 mg IV Q2W for the first 5 administrations then every 4 weeks (Q4W) in a maximum of 6 patients.
First, 3 patients will be enrolled at DL-1:

- In case of more than 1 DLT (even prior enrolment of 3 patients), accrual to DL-1 stops and accrual starts at the next lower dose level of oleclumab (dose level -2).
- In case of 1 or no DLT out of the 3 patients, 3 additional patients will be enrolled at DL-1. Those 3 additional patients will be enrolled at the earliest 28 days after treatment start of the 3rd patient:
    - If there is more than 1 DLT (even prior enrolment of 6 patients), accrual to DL-1 stops and accrual starts at the next lower dose level of oleclumab (DL-2).
    - If there is 1 or no DLT out of the 6 patients, the RP2D will be 1500 mg IV 5xQ2W then Q4W.

<u>Dose level -2 (DL-2):</u>
Oleclumab will be administered at the dose of 750 mg IV Q2W for the first 5 administrations then every 4 weeks (Q4W) in a maximum of 6 patients.
First, 3 patients will be enrolled at DL-2:

- In case of more than 1 DLT (even prior enrolment of 3 patients), accrual to DL-2 stops and the trial will be closed and the phase II part will not open.
- In case of 1 or no DLT out of the 3 patients, 3 additional patients will be enrolled at DL-2. Those 3 additional patients will be enrolled at the earliest 28 days after treatment start of the 3rd patient:
  - If there is more than 1 DLT (even prior enrolment of 6 patients), accrual to DL-2 stops and the trial will be closed and the phase II part will not open.
  - If there is 1 or no DLT out of the 6 patients, the RP2D will be 750 mg IV 5xQ2W then Q4W.

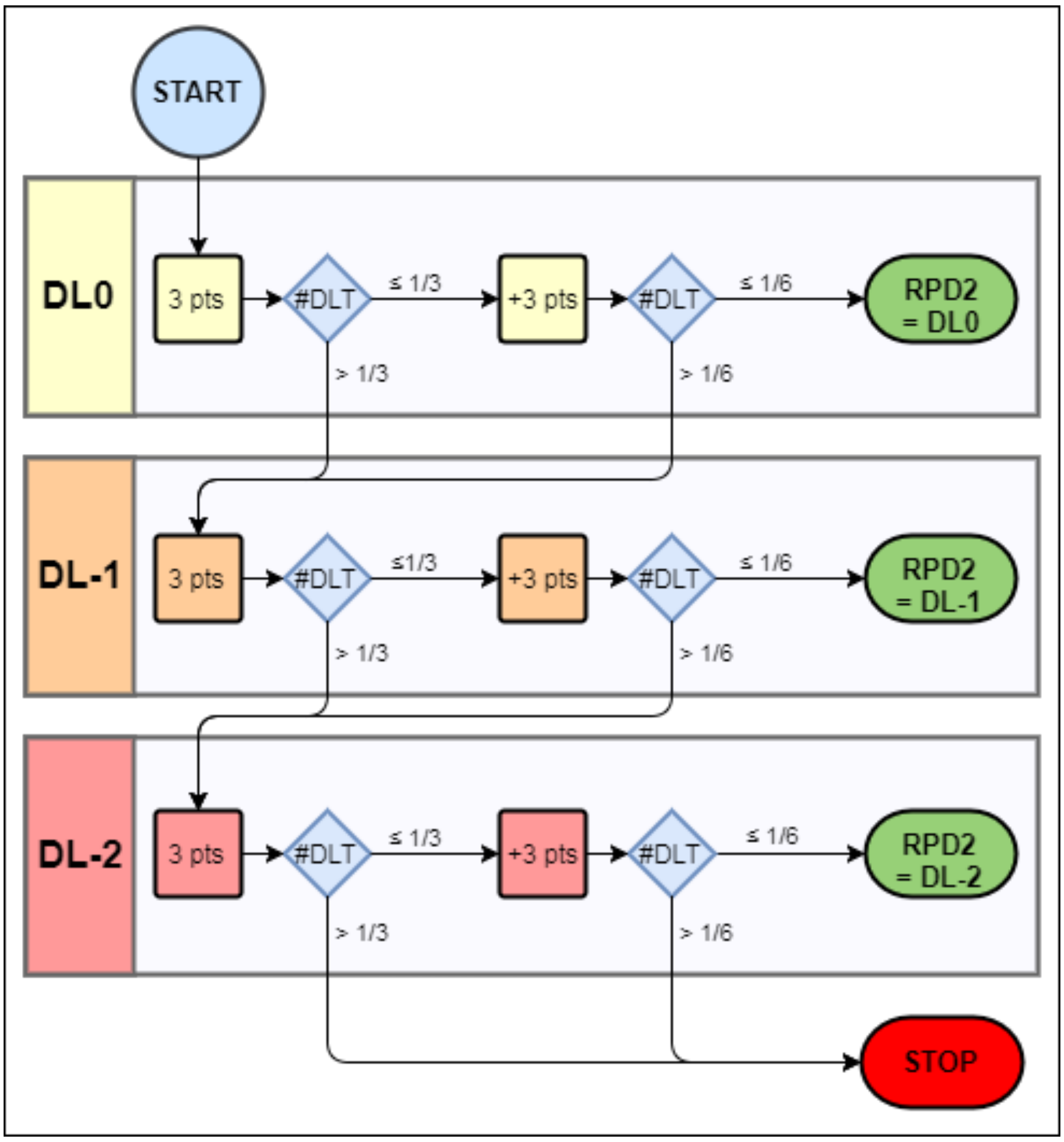

Figure 2 Recruitment of patients at different dose levels of oleclumab (MEDI9447)

After the 12 administrations of chemotherapy, the next injection of oleclumab (at week 13 in case of no chemotherapy interruption) will be given according to the current DL. Following administrations of oleclumab after the end of chemotherapy (starting week 17 in case of no chemotherapy interruption), will be given at 3000 mg IV Q4W, independently of the initial DL.

3.2. Phase II

This multicenter, randomized, open-label trial investigates the role of an anti-CD73 monoclonal antibody (MEDI9447 or oleclumab) in combination with an anti-PD-L1 antibody (durvalumab) plus chemotherapy (paclitaxel + carboplatin) as first-line treatment for locally recurrent inoperable or metastatic TNBC.

Patients will be randomized between chemotherapy (paclitaxel and carboplatin) with immunotherapy including durvalumab with or without oleclumab (1:1 ratio).

Following the results of the Phase I Safety Interim Analysis described in section 12.3., the oleclumab dose DL0 = 3000 mg is validated as RP2D for the Phase II part. Additionally, carboplatin dose was reduced from AUC 2 to AUC 1.5 due to high grade neutropenia in the majority of the subjects.

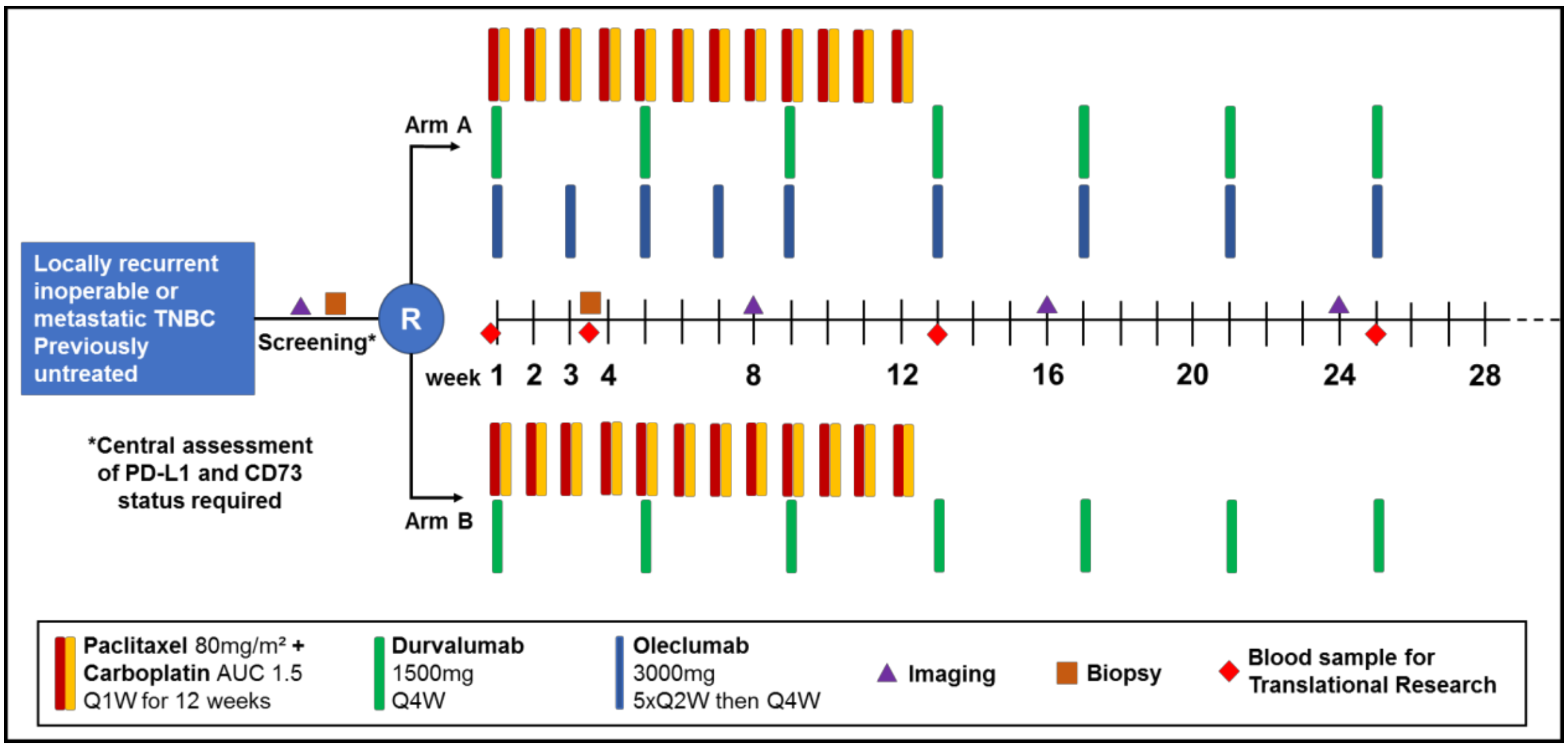

Figure 3 Phase II schema

3.2.1. Paclitaxel and Carboplatin

Paclitaxel 80 mg/m^2 IV once a week + carboplatin AUC 1.5 (Area under the curve 1.5, calculated according to the Calvert formula) IV once a week (Q1W) for 12 weeks (12 administrations of paclitaxel + 12 administrations of carboplatin).

3.2.1. Durvalumab

Durvalumab will be given at the fixed dose of 1500 mg IV Q4W.

3.2.2. Oleclumab

Oleclumab will be given (only in arm A) every two weeks for 5 administrations (Q2W) and then every 4 weeks (Q4W) at the fixed dose of 3000 mg IV, as defined in the Phase I part of the trial.

Following the results of the Phase II Safety and futility Interim Analysis (see section 12.3.2) the pre-specified boundary for futility was crossed. No safety concerns were identified for the arm with oleclumab. The IDMC recommended stopping the further recruitment for patients in the trial in both arms. Following this recommendation, recruitment stopped on 22 June 2021. Patients still under treatment can continue treatment either with oleclumab (arm A) or without (patients in arm B and patients wishing to cross from A into arm B, respectively).

3.3. Phase I and Phase II

3.3.1. Drugs administration

In case the patient is in the phase I or is randomized into arm A of the phase II, the 4 drugs will be administered the same day, the order of the administration will be as follows: oleclumab, durvalumab, paclitaxel and carboplatin.

In case the patient is randomized in arm B of the phase II, durvalumab will be administered followed by paclitaxel and followed by carboplatin.

Within 60 minutes before starting infusion (and during and after infusion, if clinically indicated), patient's vital signs (heart rate, blood pressure and temperature) should be recorded.

Oleclumab will be infused over one hour, however if there are interruptions during infusion, the total infusion time should not exceed 4 hours. For more details regarding oleclumab administration, *please see to section 6.2.1.3. Therapeutic regimen and dose.*

Durvalumab IV infusion will start no less than 15 minutes after the end of oleclumab infusion (for patients enrolled in the phase I or in arm A of the phase II) or will be the first study drug administration (for patients enrolled in arm B of the phase II). It will be administered over approximately 60 minutes. If there are interruptions during infusion, the total allowed time should not exceed 8 hours at room temperature. For more details regarding durvalumab administration, *please see to section 6.1.1.3. Therapeutic regimen and dose.*

Paclitaxel and carboplatin will be administered successively intravenously (IV) over 60 minutes each (5 minutes between the 2 infusions).

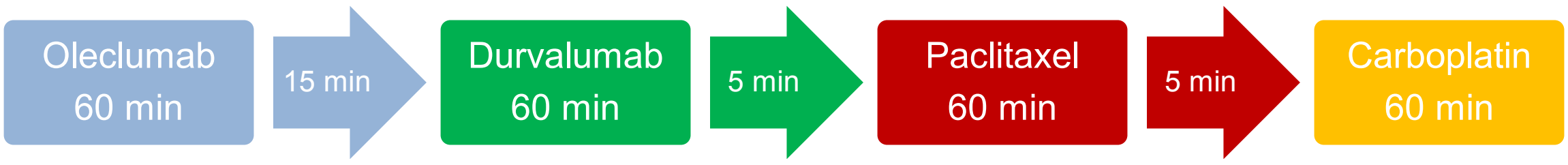

Figure 4 Study Drug Administration Schema (Phase I and Arm A Phase II)

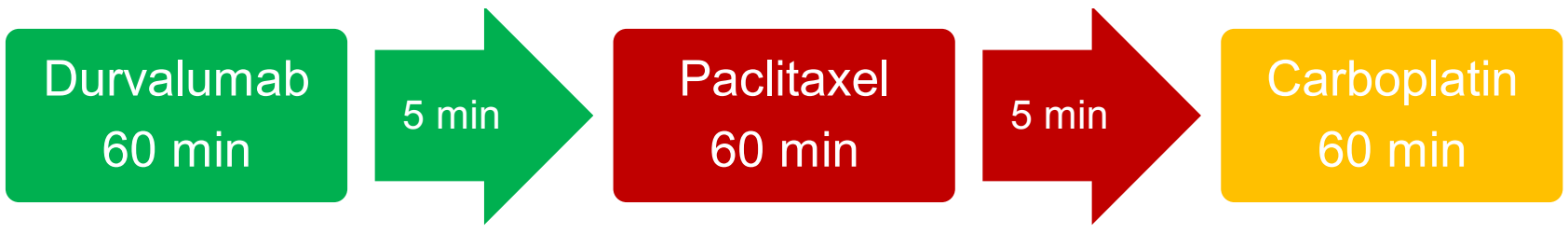

Figure 5 Study Drug Administration Schema (Arm B Phase II)

Patients will be informed about the possibility of delayed symptoms following infusion and instructed to contact their study physician if they develop such symptoms.

3.3.2. Chemotherapy Pre- and Post-medication

Chemotherapy pre-and post-medication including antiemetics, antihistamines and steroids are administered according to local standard of care.

3.3.3. Durvalumab/Oleclumab (MEDI9447) Pre- and Post-medication

No further pre- or post-medication are mandatorily required regarding durvalumab and/or oleclumab. However, further pre-medication might be required in case of infusion-related reactions (IRR). *Please see section 7.4.6. Infusion-related Reactions*

3.3.4. Chemotherapy (Re-)Treatment Criteria

Table 1 Chemotherapy (Re-) Treatment Criteria

	**First administration**	**Subsequent administrations**
Absolute neutrophil count (ANC)	1500/µl	1000/µl
Platelet count	100000/µl	75000/µl
AST/ALT	≤ 3 x ULN	≤ 3 x ULN
Bilirubin	≤ 1.5 x ULN	≤ 1.5 x ULN
eGFR	> 60 mL/min	> 60 mL/min

3.3.5. Discontinuation and duration of therapy

Paclitaxel and carboplatin will be given weekly for a total of 12 administrations. Immunotherapy (durvalumab with or without oleclumab) will be given as long as the patient benefits. Premature discontinuation of paclitaxel/carboplatin or discontinuation of durvalumab/oleclumab is indicated in case of:

- Progressive disease (PD) using RECIST v1.1 criteria or iRECIST assessed locally (please see the following section for pseudoprogression)
- Unacceptable toxicity
- Intercurrent illness that necessitates discontinuation of study treatment
- Investigator's decision to withdraw the subject
- Pregnancy
- Subject noncompliance with the study treatment or procedure requirements
- Withdrawal of consent to treatment
- Death
- Administrative reasons requiring cessation of study treatment.

Patients who stopped all study treatments for reasons other than PD will continue post-treatment imaging studies for disease status follow-up as described in the schedule of assessment until verified PD, start of a new anti-cancer treatment, withdrawal of consent to study participation, death, or end of the study whichever comes first.

Specific procedures to be performed during the trial, as well as their prescribed times and associated visit windows, are outlined in *section 8. Schedule of Assessments.*

3.3.6. Pseudo-progression related to immunotherapy

Patients experiencing PD as defined by RECIST v1.1 can continue the study treatment in case of good clinical condition assessed by a stable or even improved ECOG performance status. If the next assessment of tumour burden (8 weeks later) confirms PD (as defined by iRECIST) study treatment must be discontinued. Unconfirmed PD as defined by iRECIST (iUPD) can only be assigned for the first **2 imaging assessments** (week 8 and week 16) and several times as long as confirmed progression (iCPD) is not confirmed at the next assessment. If PD is not confirmed, reassessments continue as originally planned.

3.3.7. Imaging assessment

Initial disease status will be evaluated by imaging studies (contrast-enhanced CT scan of the chest, the abdomen and the pelvis or MRI of the chest, the abdomen and the pelvis) during the screening phase. Disease status will be followed by imaging studies at weeks 8 (± 3 days; contrast-enhanced CT scan or MRI), 16 (± 3 days; contrast-enhanced CT scan or MRI) and 24 (± 3 days; contrast-enhanced CT scan or MRI) post start of treatment (allowing efficacy data to be captured as close to 24 weeks post start of study treatment as possible for a more accurate evaluation of DCR). Thereafter imaging will continue every 12 weeks until week 104 (± 3 days; contrast-enhanced CT scan or MRI), independent of any treatment delays. After week 104, the imaging will be performed as per standard local practice. Note: Tumour assessment should be performed at the end of treatment (EOT) visit if last tumour assessment was performed more than 28 days before treatment stop date. If last tumour assessment was performed within 28 days of treatment stop date, no need to repeat this exam at the EOT visit.

The same radiographic procedures used to assess measurable disease sites at screening should be used throughout the study (e.g., the same contrast protocol for CT scans and/or MRI). All known sites of disease (including brain in case of known central nervous system (CNS) metastases) must be documented at screening and re-assessed at each subsequent tumour evaluation. Assessments should be performed by the same evaluator, if possible, to ensure internal consistency across visits.

4. SELECTION OF SUBJECTS

4.1. Inclusion criteria

Patients must meet all the following criteria to be eligible for the study:

1. Age of ≥ 18 years
2. Female
3. Life expectancy of a least 12 weeks
4. Body weight above 35kg
5. The locally recurrent or metastatic relapse must be histologically confirmed TNBC in patients not previously treated with systemic treatment and which cannot be treated with curative intent. Newly diagnosed patients with de-novo metastatic disease are eligible
6. Estrogen receptor (ER) and progesterone receptor (PR) negativity (< 1% positive staining cells in the invasive tumour) determined locally using IHC per ASCO/CAP criteria)[62]
7. Human epidermal growth factor receptor 2 (HER2) negativity (negative IHC staining [score 0 or 1] or negative fluorescence in situ hybridization [FISH] based on the ASCO/CAP guidelines and recommendations) and determined locally[63]

   Note: patients initially diagnosed with hormone receptor–positive and/or HER2-positive breast cancer OR de novo metastatic patients with a primary tumour hormone receptor-positive (weak positivity or ER negativity and PR positivity) considered as non-clinically relevant are eligible if the tumour biopsy obtained from a local recurrence or distant metastasis site confirms the TNBC disease.
8. Confirmed tumour PD-L1 and CD73 IHC assessment as documented through central testing of a representative tumour tissue specimen for stratification purposes (**only for phase II**).
9. Provision of recurrence/metastatic tissue samples from resections, core-needle biopsies or excisional, incisional, punch, or forceps biopsies:
   - At least 1 FFPE [Formalin-Fixed paraffin-embedded] tumour tissue and 1 frozen core as a priority, if feasible 2 additional fresh tumour tissue cores should be collected too.
   - Fine-needle aspiration (FNA) (defined as samples that do not preserve tissue architecture and yield cell suspension and/or smears), brushing, and cell pellets from cytology samples are not acceptable.

   Note 1: If the subject has just performed a metastatic lesion biopsy, she is eligible only if a FFPE tissue sample (or at least 20 unstained slides, freshly cut for the purposes of the study) of the metastatic/recurrent lesion is available. In this situation only, frozen cores are not mandatory.

   Note 2: In case of a de-novo metastatic disease, if the biopsy of a metastatic lesion is not feasible, the patient is eligible if primary tumour lesion samples (FFPE + frozen cores) are available.
10. Provision of an archived FFPE diagnostic biopsy or surgical primary breast tumour sample (or at least 20 unstained slides, freshly cut for the purposes of the study).

    Note: In case of neoadjuvant treatment (before surgery), the diagnostic biopsy is preferable.
11. At least 6 months elapsed between the completion of surgical and/or systemic treatment with curative intent (e.g., the date of primary breast tumour surgery or the date of last adjuvant chemotherapy administration (radiotherapy is not included), whichever occurred last) and first documented local or distant disease recurrence (NOTE: not applicable for de-novo metastatic disease)

12. At least one measurable disease based on RECIST v1.1. Tumour lesions in a previously irradiated area are considered measurable, if progression has been demonstrated in such lesions
13. Adequate organ function:
    a) Absolute neutrophil count (ANC) ≥ 1500/µl (without the addition of growth factors)
    b) Platelets [PLT] ≥ 100000/µl (without the addition of growth factors/prior transfusions)
    c) Hemoglobin (Hb) ≥ 10 g/dl (without the addition of growth factors/prior transfusions)
    d) Creatinine ≤ 1.5 x upper limit of normal (ULN) OR estimated glomerular filtration rate (eGFR) ≥ 60 ml/min as calculated using the method standard for the institution. If eGFR is lower than 60 ml/min, a 24-hour urine creatinine clearance can be performed to rule out an underestimation of the eGFR.
    e) Total serum bilirubin (TBL) ≤ 1.5 x ULN unless the subject has documented Gilbert syndrome in which case up to 3 x ULN is acceptable
    f) Aspartate and alanine aminotransferase (AST/ALT) ≤ 2.5 x ULN unless liver metastases are present, in which case it must be ≤ 5 x ULN
    g) International Normalized Ratio (INR) ≤ 1.5 x ULN unless subject is receiving anticoagulant therapy as long as INR and activated partial thromboplastin time (aPTT) is within therapeutic range of intended use of anticoagulants
14. Performance status (PS) of 0 or 1 on the ECOG Performance scale
15. Female subjects of childbearing potential (FSCP) must be willing to use one highly effective method of contraception (detailed at protocol section 6.6.) for the course of the study through 6 months after the last study drug administration. FSCP must have a negative serum pregnancy test done within the 28 days before treatment start. FSCP are those who have not been surgically sterilized or have not been free of menses for at least 1 year.
16. Absence of any psychological, familial, sociological or geographical condition potentially hampering compliance with the study protocol and follow-up schedule; those conditions should be discussed with the patient before registration in the trial
17. Absence of any concurrent illness that would preclude the evaluation of safety
18. Agreement to provide tissue and blood samples for research purposes
19. Written informed consent must be given according to ICH/GCP, and national/local regulations before patient enrolment

Inclusion criterion applicable to FRANCE only

20. Affiliated to the French Social Security System

Note: patients eligible for the Synergy trial are also eligible for the AURORA program (NCT02102165) or any other similar molecular screening program, aiming to understand the molecular aberrations in metastatic BC. Patients can be included in both trials in centres recruiting patients for AURORA or for any similar molecular screening program as long as tumour tissue and blood samples can be collected for both studies. If tissue and blood samples cannot be collected for both, it is the investigator choice (and patient) to decide in which trial the patient will enter.

4.2. Exclusion criteria

Patients who exhibit any of the following conditions at screening are ineligible for admission into the study:

1. Active or prior documented autoimmune or inflammatory disorders (including inflammatory bowel disease [e.g., colitis or Crohn's disease], diverticulitis [with the exception of diverticulosis], systemic lupus erythematosus, Sarcoidosis syndrome, or Wegener syndrome [granulomatosis with polyangiitis, Graves' disease, rheumatoid arthritis, hypophysitis, uveitis, etc]). The following are exceptions to this criterion:

a) Patients with vitiligo or alopecia
b) Patients with hypothyroidism (e.g., following Hashimoto syndrome) stable on hormone replacement
c) Any chronic skin condition that does not require systemic therapy
d) Patients without active disease in the last 5 years may be included but only after consultation with the sponsor
e) Patients with celiac disease controlled by diet alone

2. Current or prior treatment with immunosuppressive medication within 14 days prior to enrolment. The following are exceptions to this criterion:
   a) Intranasal, inhaled, topical steroids, or local steroid injections (e.g., intra articular injection)
   b) Systemic corticosteroids at physiologic doses not to exceed 10 mg/day of prednisone or its equivalent
   c) Steroids as premedication for hypersensitivity reactions (e.g., CT scan premedication
3. Any live, attenuated vaccine administered within 28 days prior to enrolment or anticipation that such a live attenuated vaccine will be required during the study
4. Chronic daily treatment with non-steroidal anti-inflammatory drug (NSAID) (occasional use for the symptomatic relief of medical conditions, for example, headache, fever is allowed)
5. Active infection including
   a) Tuberculosis (TB) (clinical evaluation that includes clinical history, physical examination and radiographic findings, and TB testing in line with local practice)
   b) Hepatitis B (known positive HBV surface antigen (HBsAg) result). Patients with a past or resolved HBV infection (defined as the presence of hepatitis B core antibody [anti-HBc] and absence of HBsAg) are eligible.
   c) Hepatitis C. Patients positive for hepatitis C (HCV) antibody are eligible only if polymerase chain reaction is negative for HCV RNA.
   d) Human immunodeficiency virus (positive HIV 1/2 antibodies).
6. Treatment with systemic immunostimulatory agents, including but not limited to, interferon (IFN)-alpha, IFN-beta, interleukin (IL)-2, conjugated IL-2 cytokines within 42 days or five half-lives of the drug, whichever is longer, prior to screening
7. Previous treatment with immune checkpoint inhibitors (e.g. anti-PD-1, anti-PD-L1 including durvalumab, anti-Cytotoxic T-lymphocyte-associated molecule-4), anti-CD73 antibodies, adenosine A2A receptor antagonists, or prior treatment with CD137 agonists/OX-40 agonists or any other antibody or drug targeting T-cell co-stimulation or other immunomodulatory therapies
8. Any unresolved toxicity NCI CTCAE Grade ≥ 2 from previous anticancer therapy with the exception of alopecia, vitiligo and the laboratory values defined in the inclusion criteria
9. Known hypersensitivity reactions to the study drugs or to any of the excipients, pre-medications (acetaminophen/paracetamol, diphenhydramine or equivalent anti-histamine and methylprednisolone or equivalent glucocorticoid) and to other platinum containing compounds
10. Untreated central nervous system (CNS) metastases and/or carcinomatous meningitis.

    Subjects with previously treated brain metastases with local treatment (stereotactic radiosurgery or whole brain radiation therapy) may participate provided they have stable brain metastases on a recent brain MRI (performed during the 2 weeks prior inclusion) and have measurable disease outside the CNS.

    Note: Known brain metastases are considered active (and not eligible for trial), if any of the following criteria are applicable:

   a) Recent brain imaging demonstrates progression of existing and/or appearance of new lesions
   b) Neurological symptoms attributed to brain metastases have not returned to baseline
   c) Steroids were used for management of symptoms related to brain metastases within 14 days of enrolment
   d) Completion of local therapy for brain metastases within 28 days of enrolment

11. Major surgical procedure (as defined by the principal investigator) within 28 days prior to enrolment. Note: Local surgery of isolated lesions for palliative intent is acceptable.
12. Uncontrolled intercurrent illness, including but not limited to,
    a) Symptomatic congestive heart failure, uncontrolled hypertension, unstable angina pectoris, cardiac arrhythmia. Patients previously treated with anthracyclines are eligible if a recent cardiac work up (< 6 months) demonstrated a normal left ventricular ejection fraction (LVEF≥50%).
    b) Interstitial lung disease
    c) Serious chronic gastrointestinal conditions associated with diarrhoea
    d) Psychiatric illness/social situations that would limit compliance with study requirement, substantially increase risk of incurring AEs or compromise the ability of the patient to give written informed consent
13. Past medical conditions, including,
    a) Class II-IV congestive heart failure
    b) Myocardial infarction within 12 months prior enrolment,
    c) Deep vein thrombosis (DVT) or thrombo-embolic event within 12 months prior to enrolment
    d) History of stroke or transient ischemic attack requiring medical therapy
    e) Intra-abdominal inflammatory process within the last 12 months prior to enrolment such as, but not limited to, diverticulitis, peptic ulcer disease, or colitis
    f) History of idiopathic pulmonary fibrosis, organizing pneumonia (e.g. bronchiolitis obliterans), drug-induced pneumonitis, idiopathic pneumonitis, or evidence of active pneumonitis
    g) History of another primary malignancy except for malignancy treated with curative intent and with no known active disease ≥5 years before the first dose of IP and of low potential risk for recurrence, adequately treated non-melanoma skin cancer or lentigo maligna without evidence of disease, adequately treated carcinoma in situ without evidence of disease
    h) Status post allogeneic bone marrow transplantation or solid organ transplantation
14. Pregnant or lactating women.

Exclusion criterion applicable to FRANCE only

15. Vulnerable persons according to the article L.1121-6 of the Public Health Code, adults who are the subjects of a measure of legal protection or unable to express their consent according to article L.1121-8 of the Public Health Code.

4.3. Subject registration/inclusion procedure

The procedure for a subject participation in this study is a two steps procedure: a registration step and an inclusion step.

As soon as the subject has signed the informed consent form, she should be registered in the web based “Subject Registration Tool” (= step 1 registration). The system assigns the study subject number.

If the screened subject meets all eligibility criteria, she is enrolled via the web based “Subject Registration Tool” (= step 2 inclusion). For the phase II part, this step will be a randomisation

process. A maximum of 3 days is allowed between subject's enrolment and first study drug administration.

If the subject does not meet all eligibility criteria, she is considered as a screen failure. Screen failures have also to be reported via the web based "Subject Registration Tool" as not enrolled.

Screen failure is defined as any subject that signed the ICF and is finally not enrolled in the study. Any screen failure must be documented by the participating sites and reported via the Subject Registration Tool.

Screening and inclusion guidelines are provided to the participating sites. Those guidelines are described in the Subject Registration Manual.

5. CRITERIA FOR SUBJECT WITHDRAWAL

The investigator has the right to discontinue a subject from the study treatment or withdraw a subject from the study at any time. The subject has also the right to discontinue the study treatment or withdraw from the study at any time without giving its reasons. Although it is not mandatory for a patient to give her reason for withdrawing prematurely from the study treatment or the complete study, the investigator should make reasonable effort to ascertain the reason(s), while fully respecting the subject's rights.

Reasons of premature discontinuation from the study treatment or from the entire study may include, but are not limited to, the following:

- Subject 's request/ consent withdrawal
- Any medical condition that the investigator or the sponsor considers may jeopardise the subjects' safety if she continues the study treatment or the study
- Investigator or sponsor determines it is in the best interest of the subject
- Subject non-compliance
- Pregnancy

The reason should be documented in the medical notes of the subject and in eCRF.

In case, the subject decides to stop prematurely the study treatment, the investigator will ask her if she agrees to continue attending the study visits as per schedule assessments and/or to provide survival data.

The outcome of the discussion between the investigator and the patient should be documented in the medical notes of the subject and in the eCRF.

Any subject who discontinues the study will be encouraged to return to the site for a study completion visit and to allow appropriate safety evaluations of ongoing AEs.

In the case of subject who fails to attend scheduled visits, several attempts should be made by the investigator to contact the subject or one responsible relative or the subject's general practitioner for follow up information. It is only after sufficient unsuccessful attempts to contact the subject, that a subject may be declared "lost to follow-up". An excessive rate of withdrawals and/or subjects lost to follow-up can make the study uninterpretable; therefore, unnecessary subject attrition should be avoided. The attempts and their outcome should be documented in the medical notes of the subject.

In case the sponsor recommends discontinuation of treatment, the subject should continue attending the study visits as per the schedule of assessments.

For safety reasons, during the study, no other treatment for cancerous disease, whether experimental or not, can be administered.

However, the patient may participate in non-interventional studies that do not directly affect her therapeutic management throughout the duration of this study.

6. STUDY TREATMENTS

6.1. Investigational Medicinal Product: **Durvalumab**

6.1.1. Drug information

INN: Durvalumab (MEDI4736)

Description: Durvalumab is a human immunoglobulin G1 kappa (IgG1κ) monoclonal antibody that targets PD-L1.

Mechanism of action: Durvalumab blocks the interaction of PD-L1 (but not PD-L2) with PD-1 on T cells and CD80+ immune cells and is engineered to reduce ADCC.

6.1.1.1. *Formulation*

Liquid drug product: Durvalumab is formulated at 50 mg/mL in 26 mM histidine/histidine-HCl, 275 mM trehalose dihydrate, 0.02% (w/v) polysorbate 80, pH 6.0. The investigational product is supplied as a liquid solution in clear 10R glass vials closed with an elastomeric stopper and a flip off cap overseal. Each vial contains 500 mg (nominal) of active investigational product at a concentration of 50 mg/mL. The solution will be diluted with 0.9% (w/v) saline or 5% (w/v) dextrose for IV infusion.

6.1.1.2. *Preparation*

The instructions for the preparation of durvalumab solution are found in the IMP management manual.

The durvalumab doses must be prepared by the investigator's or site's designated investigational product manager or other qualified professional using aseptic technique.

Total time from needle puncture of the durvalumab (MEDI4736) vial prior to the start time of the infusion must not exceed:

- 24 hours at 2°C to 8°C (36°F to 46°F)
- 4 hours at room temperature

In the event dose preparation time exceeds the specified time limits, the preparation must be re-prepared from new vials.

6.1.1.3. *Therapeutic regimen and dose*

Durvalumab will be given at the fixed dose of 1500 mg IV every 4 weeks (Q4W).

Durvalumab IV infusion will start no less than 15 minutes after the end of oleclumab infusion (for patients enrolled in the phase I or in arm A in the phase II) or will be the first study drug administration (for patients enrolled in arm B in the phase II)., and will be administered over approximately 1 hour through a 0.2- or 0.22-μm in-line filter.

If there are interruptions during infusion, the total allowed time should not exceed 8 hours at room temperature. In the event infusion time exceeds the specified time limits, the infusion must be re-prepared from new vials.

Durvalumab solution should not be infused together with other solutions or medications.

6.1.1.4. *Stability and storage*

Durvalumab must be stored at the participating sites in a secure area in accordance with Good Clinical Practice (GCP) and Good Manufacturing Practice (GMP) requirements and must be accessible to authorised personnel only.

Storage conditions are found in the IMP management manual.

6.1.1.5. *Labelling*

Durvalumab is labelled according to the regulatory requirements of each country, as well as the Rules Governing Medicinal Products in the European Union, Volume 4, EU Guidelines to Good Manufacturing Practice, Medicinal Products for Human and Veterinary Use, Annex 13, Investigational Medicinal Products.

6.1.1.6. *Drug logistics and accountability*

Durvalumab is provided to all participating sites labelled for investigational use only. The site acknowledges receipt of the IMP according to defined study procedure set up at study start. Any damaged shipment is replaced (if applicable). Accurate records of the IMP reception, dispensing, return from the subject (if applicable) and destruction or return to the sponsor (if applicable) must be recorded on the Drug Accountability Form.

6.1.2. Toxicity

The adverse reactions observed for durvalumab can be found in the Investigator's brochure. Please see section 7. Toxicity management and doses adjustments.

6.2. Investigational Medicinal Product: **Oleclumab**

6.2.1. Drug information

INN: Oleclumab (MEDI9447)

Description: Oleclumab is a human immunoglobulin G1 lambda (IgG1λ) monoclonal antibody (mAb) with a triple mutation in the heavy chain constant region for reduced effector function that selectively binds to and inhibits the ectonucleotidase activity of CD73.

Mechanism of action: Oleclumab inhibits the production of adenosine from AMP by CD73. Oleclumab relieves adenosine-mediated immunosuppression in the tumour microenvironment.

6.2.1.1. *Formulation*

Oleclumab (MEDI9447) is supplied in 10R vials as a sterile clear to opalescent, colorless to slightly yellow liquid, free from or practically free from visible particles containing 50 mg/mL oleclumab, 25 mM histidine/histidine hydrochloride (HCl), 240 mM sucrose, 0.03% (weight/volume [w/v]) polysorbate 80, at pH 6.0. Oleclumab will be diluted into an IV bag containing 0.9% (w/v) saline and administered via IV infusion.

6.2.1.2. *Preparation*

The instructions for the preparation of oleclumab solution are found in the IMP management manual.

The oleclumab dose must be prepared by the site-designated pharmacist using aseptic technique.

6.2.1.3. *Therapeutic regimen and dose*

Oleclumab is given IV every 2 weeks (Q2W) for the first 5 administrations then every 4 weeks (Q4W).

The RP2D was defined during the phase I part of the trial and DL0 (3000 mg) will be used thereafter during the phase II part of the trial.

Do not co-administer other drugs through the same infusion line.

Following dose preparation, the entire content of the IV bag should be administered. The IV line will be flushed with a volume of IV diluent equal to the priming volume of the infusion set used after the content of the IV bag is fully administered, or complete the infusion according to institutional policy to ensure the full dose is administered and document if the line was not flushed.

Oleclumab will be administered at room temperature (approximately 20 to 25°C) by controlled infusion into a peripheral or central vein. Standard infusion time for oleclumab is one hour, however if there are interruptions during infusion, the total allowed time should not exceed 4 hours at room temperature. If this duration is met, then the remaining dose should be abandoned and should not be completed with a second prepared dose.

6.2.1.4. *Stability and storage*

Oleclumab must be stored at the participating sites in a secure area in accordance with GCP and GMP requirements and must be accessible to authorised personnel only.

Storage conditions are found in the IMP management manual.

6.2.1.5. *Labelling*

Oleclumab is labelled according to the regulatory requirements of each country, as well as to the Rules Governing Medicinal Products in the European Union, Volume 4, EU Guidelines to Good Manufacturing Practice, Medicinal Products for Human and Veterinary Use, Annex 13, Investigational Medicinal Products.

6.2.1.6. *Drug logistics and accountability*

Oleclumab is provided to all participating sites labelled for investigational use only. The site acknowledges receipt of the IMP defined study procedure set up at study start. Any damaged shipment is replaced (if applicable). Accurate records of the IMP reception, dispensing, return from the subject (if applicable) and destruction or return to the sponsor (if applicable) must be recorded on the Drug Accountability Form.

6.2.2. Toxicity

The adverse reactions observed for oleclumab can be found in the Investigator’s brochure. *Please see section 7. Toxicity management and doses adjustments*.

6.3. Investigational Medicinal Product: **Paclitaxel**

6.3.1. Drug information

INN: Paclitaxel

Description: Paclitaxel is an antimicrotubule agent.

Mechanism of action: Paclitaxel promotes the assembly of microtubules from tubulin dimers and stabilises microtubules by preventing depolymerisation. This stability inhibits the normal dynamic reorganisation of the microtubule network, which is essential for vital interphase and mitotic cellular functions. In addition, paclitaxel induces abnormal arrays or bundles of microtubules throughout the cell cycle and multiple asters of microtubules during mitosis.

6.3.1.1. *Formulation*

The formulation of paclitaxel drug product to use is the one commonly available at the site pharmacy.

6.3.1.2. *Preparation*

The instructions for the preparation of paclitaxel solution are found in the package insert or in the Summary of Product Characteristics.

6.3.1.3. *Therapeutic regimen and dose*

Paclitaxel is given at a dose of 80 mg/m² IV once a week for 12 weeks.

6.3.1.4. *Stability and storage*

It must be stored at the participating sites in a secure area in accordance to GCP and GMP requirements and must be accessible to authorised personnel only.

Storage conditions are found in the package insert or in the Summary of Product Characteristics.

6.3.1.5. *Labelling*

Paclitaxel is labelled according to the regulatory requirements of each country, as well as to the Rules Governing Medicinal Products in the European Union, Volume 4, EU Guidelines to Good Manufacturing Practice, Medicinal Products for Human and Veterinary Use, Annex 13, Investigational Medicinal Products.

6.3.1.6. *Drug logistics and accountability*

The study drug is taken from the commercial stocks of the hospital pharmacies and will be re-labelled by the pharmacists prior to providing them to the subjects.

Accurate records of the IMP dispensing, batch/lot number and expiry date must be recorded on the Drug Accountability Form if the local pharmacy does not have any available system in place. If due to an internal site process, the completion of this drug accountability log for this standard chemotherapy is not feasible, the completion of this log is not mandatory in case the site is able to provide the batch/lot number administered to a specific subject under request (e.g. in case of audit, inspection or safety issue).

6.3.2. Toxicity

The adverse reactions observed for paclitaxel can be found in the Summary of Product Characteristics.
Please see section 7. Toxicity management and doses adjustments.

6.4. Investigational Medicinal Product: **Carboplatin**

6.4.1. Drug information

INN: Carboplatin

Description: Carboplatin is an antineoplastic agent.

Mechanism of action: Carboplatin is a second-generation platinum compound with a broad spectrum of antineoplastic properties. Carboplatin contains a platinum atom complexed with

two ammonia groups and a cyclobutane-dicarboxyl residue. This agent is activated intracellularly to form reactive platinum complexes that bind to nucleophilic groups such as GC-rich sites in DNA, thereby inducing intrastrand and interstrand DNA cross-links, as well as DNA-protein cross-links. These carboplatin-induced DNA and protein effects result in apoptosis and cell growth inhibition.

6.4.1.1. *Formulation*

The formulation of carboplatin drug product to use is the one commonly available at the site pharmacy.

6.4.1.2. *Preparation*

The instructions for the preparation of carboplatin solution are found in the package insert or in the Summary of Product Characteristics.

6.4.1.3. *Therapeutic regimen and dose*

Carboplatin is given at a dose of AUC 2 IV (in phase I) and at a dose of AUC 1.5 IV (in phase II), once a week for 12 weeks.

AUC stands for Area under the curve, calculated according to the Calvert formula: Carboplatin dose = Target AUC * (GFR +25).

6.4.1.4. *Stability and storage*

It must be stored at the participating sites in a secure area in accordance with GCP and GMP requirements and must be accessible to authorised personnel only.

Storage conditions are found in the package insert or in the Summary of Product Characteristics.

6.4.1.5. *Labelling*

Carboplatin is labelled according to the regulatory requirements of each country, as well as to the Rules Governing Medicinal Products in the European Union, Volume 4, EU Guidelines to Good Manufacturing Practice, Medicinal Products for Human and Veterinary Use, Annex 13, Investigational Medicinal Products.

6.4.1.6. *Drug logistics and accountability*

The study drug is taken from the commercial stocks of the hospital pharmacies and will be re-labelled by the pharmacists prior to providing them to the subjects.

Accurate records of the IMP dispensing, batch/lot number and expiry date must be recorded on the Drug Accountability Form if the local pharmacy does not have any available system in place. If due to an internal site process, the completion of this drug accountability log for this standard chemotherapy is not feasible, the completion of this log is not mandatory in case the site is able to provide the batch/lot number administered to a specific subject under request (e.g. in case of audit, inspection or safety issue).

6.4.2. Toxicity

The adverse reactions observed for carboplatin can be found in the Summary of Product Characteristics.
Please see section 7. Toxicity management and doses adjustments.

6.5. Non-Investigational Medicinal Product(s)

6.5.1. Chemotherapy pre- and post-medication

Chemotherapy pre- and post-medication including antiemetics, antihistamines and steroids are administered according to local standard of care.

6.5.2. Gadolinium-based contrast agents

Gadolinium is a rare-earth based contrast that is injected intravenously before a patient undergoes MRI in order to improve the discernibility of lesions. Examples of gadolinium-based contrast agents: gadoterate meglumine, gadobutrol, gadobenate dimeglumine.

About specific Gadolinium-based contrast agents it is important to note that the participating site should follow its own usual practices of agent dose and choice, as long as they are in accordance with the regulatory authorities rulings and guidelines. In accordance with the final opinion of the European Medicines Agency (EMA/625317/2017 date 23 Nov 2017) on use of linear gadolinium agents in body scan, we recommend, however that:

- Macrocyclic Gadolinium-based contrast agents should be favoured over linear agents;
- The use of Magnevist (gadopentetic acid), Omniscan (gadodiamide) and Optimark (gadoversetamide) should be avoided;
- The same Gadolinium-based contrast agent is used for each patient for all MRI scans.
- Gadolinium-based contrast agents should be used in the lowest doses that enhance images sufficiently.

6.5.3. Iodinated contrast agent (referred henceforth as iodine)

Iodine is an iodinated-containing contrast agent used during the performance of CT scans. It is injected intravenously into patients before exam performance and serves the purpose of increasing the capacity to discern lesions. Example of iodinated contrast agent: iomeprol.

Information on formulation, preparation, stability, storage conditions and toxicity of NIMPs can be found in the package insert or in the Summary of Product Characteristics.

Treatment management, dosing and compliance are left at the discretion of the participating site, according to local guidelines for use of contrast agents during radiological exams.

6.6. Contraception

Female subjects of childbearing potential must agree to always use **one highly effective** method of contraception during the course of this study and for at least 6 months after the last administration of the study treatments.

The highly effective methods of contraception accepted in this study are the following:

1) Intrauterine device
2) Intrauterine hormone-releasing system
3) Bilateral tubal occlusion
4) Vasectomised partner
5) Sexual abstinence

Note: hormonal contraceptives (oestrogen and/or progestogen containing) that inhibit ovulation are highly effective methods of contraception, but they should *be used with caution* during the treatment of breast cancer (including TNBC). The investigator should discuss with the subject the benefits and risks of using hormonal contraceptives in subjects with TNBC and other forms of contraception (as listed above) should be encouraged.

6.7. Concomitant treatments

Concomitant therapy includes any medication (e.g., prescription drugs, over-the-counter drugs, herbal or homeopathic remedies, nutritional supplements) used by a patient from 28 days prior to the enrolment to discontinuation visit (end of treatment visit). All such medications should be reported to the investigator (including start/stop dates, dose, indication and any other details as per CRF completion guideline) and recorded on the Concomitant Medications Case Report Form (CRF).

6.7.1. Allowed Concomitant treatments

The following therapies are permitted on study:

- Prophylactic or therapeutic anticoagulation therapy (such as low−molecular weight heparin or warfarin at a stable dose level)
- Inhaled corticosteroids for chronic obstructive pulmonary disease
- Topical steroids, or local steroid injections (e.g. intrarticular)
- Mineralocorticoids (e.g., fludrocortisone)
- Systemic corticosteroids at physiologic doses that do not exceed 10 mg/day of prednisone or its equivalent
- If clinically indicated, pre-medication with corticosteroids as per local practice prior to chemotherapy, durvalumab, oleclumab
- Bisphosphonates or denosumab for the prevention of skeletal events
- Radiation therapy for patients whose extracranial tumour burden is stable or responding to study treatment and who subsequently develop 1 to 3 brain metastases. These patients may receive brain radiation therapy (either stereotactic radiosurgery or whole brain radiation therapy) and remain on study treatment provided they meet the following criteria:
  - No evidence of progression or haemorrhage after the completion of CNS directed therapy
  - No ongoing requirement for corticosteroid therapy for CNS disease (anticonvulsants at a stable dose are allowed)
  - Patients who require corticosteroids for more than 21 days after the completion of radiation must be discontinued from study treatment
  - Note: Treatment with chemotherapy and durvalumab/ oleclumab should be withheld during CNS-directed radiation therapy.
- Symptomatic treatment including acetaminophen, ibuprofen, promethazine, steroids and/or H2 receptor antagonists indicated for infusion-associated symptoms.
- Radiotherapy for palliative reasons on non-target lesion (e.g., treatment of known bone metastases to prevent fractures and/or reduce pain). It is not required to hold durvalumab/ oleclumab during palliative radiotherapy; chemotherapy should be interrupted per institutional standard of care.
- Inactive influenza vaccinations during influenza season ONLY (approximately October to March)

Loco-regional ablative procedures are conceivable **upon agreement of the Sponsor** for patients with excellent response after week 24 (in order to guarantee evaluation of the primary endpoint of the study).

6.7.2. Prohibited Concomitant treatments and procedures

The following treatments and procedures are not allowed during the treatment period of the study and for at least 28 days prior to the enrolment, unless otherwise specified below:

- Traditional herbal medicines; these therapies are not fully studied and their use may result in unanticipated drug-drug interactions that may cause or confound the assessment of toxicity
- Treatment with systemic steroids of more than prednisone 10 mg/day or its equivalent or any other form of immunosuppressive agents (including but not limited to cyclophosphamide, azathioprine, methotrexate, and thalidomide). These treatments need to have been stopped at least 14 days prior to the first dose of study medication
- Oral hormone replacement therapy
- Chronic daily treatment with non-steroidal anti-inflammatory drug (NSAID) (occasional use for the symptomatic relief of medical conditions, for example, headache, fever is allowed)
- Treatment with systemic immunostimulatory agents, including but not limited to, interferon (IFN)-alpha, IFN-beta, interleukin (IL)-2, conjugated IL-2 cytokines within 42 days or five half-lives of the drug, whichever is longer, prior to screening
- Any live, attenuated vaccine during treatment, or within 28 days following the last dose of durvalumab/ oleclumab
- Treatment with anti−TNF-α agents.
- Patients should not donate blood while participating in this study or for at least 90 days following the last infusion of durvalumab.

6.7.3. Incompatibilities/Drug Interactions

6.7.3.1. *Durvalumab*

No formal pharmacokinetics (PK) drug-drug interaction studies have been conducted with durvalumab.

6.7.3.2. *Oleclumab*

No formal PK drug-drug interactions studies have been conducted with oleclumab.

6.7.3.3. *Paclitaxel*

The metabolism of paclitaxel is catalysed, in part, by cytochrome P450 isoenzymes CYP2C8 and CYP3A4. Therefore, in the absence of a PK drug-drug interaction study, caution should be exercised when administering paclitaxel concomitantly with medicines known to inhibit either CYP2C8 or CYP3A4 (e.g. ketoconazole and other imidazole antifungals, erythromycin, fluoxetine, gemfibrozil, clopidogrel, cimetidine, ritonavir, saquinavir, indinavir, and nelfinavir) because toxicity of paclitaxel may be increased due to higher paclitaxel exposure. Administration of paclitaxel concomitantly with medicines known to induce either CYP2C8 or CYP3A4 (e.g. rifampicin, carbamazepine, phenytoin, efavirenz, nevirapine) is not recommended because it could result in lower paclitaxel exposures and efficacy may be compromised.

6.7.3.4. *Carboplatin*

When combining carboplatin with other myelosuppressive compounds or radiation therapy, the myelosuppressive effect of carboplatin and/or the other compounds may be more pronounced. Patients receiving concomitant therapy with other nephrotoxic agents are likely to experience more severe and prolonged myelotoxicity due to decreased renal clearance of carboplatin.

<u>Concomitant use contraindicated</u>

- Yellow fever vaccine: risk of generalized vaccinal disease mortality.

Concomitant use not recommended

- Live attenuated vaccines (except yellow fever): risk of systemic, possible fatal disease. This risk is increased in subjects who are already immunosuppressed by their underlying disease. Use an inactivated vaccine where this exist (poliomyelitis).
- Phenytoin, fosphenytoin- risk of exacerbation of convulsions resulting from the decrease of phenytoin digestive absorption by the cytotoxic drug or risk of toxicity enhancement or loss of efficacy of the cytotoxic drug due to increased hepatic metabolism by phenytoin.

Concomitant use to be taken into consideration

- Chelating agents - decreasing effect of carboplatin
- Cyclosporin (and by extrapolation tacrolimus and sirolimus): Excessive immunosuppression with risk of lymphoproliferation.
- Aminoglycosides: The concomitant use of carboplatin with aminoglycosides antibiotics should be taken into account due to the cumulative nephrotoxicity and ear toxicity, particularly in patients with severe renal impairment.
- Loop diuretics: The concomitant use of carboplatin with loop diuretic should be taken into account due to the cumulative nephrotoxicity and ear toxicity.
- Due to the increase of thrombotic risk in case of tumoural diseases, the use of anticoagulative treatment is frequent. The high intra-individual variability of the coagulability during diseases, and the eventuality of interaction between oral anticoagulants and anticancer chemotherapy, require, if it is decided to treat the patient with vitamin K antagonists, to increase frequency of the control of the INR monitoring. Caution and more frequent INR monitoring are recommended with concomitant treatment of warfarin with Carboplatin, as increased INR has been reported.

Carboplatin may interact with aluminium to form a black precipitate. Needles, syringes, catheters or IV administration sets that contain aluminium parts which may come into contact with carboplatin, should not be used for the preparation or administration of the drug.

7. TOXICITY MANAGEMENT AND DOSE ADJUSTMENTS

The severity of adverse events will be graded according to the NCI CTCAE v5.0 grading system and will decide on dose modifications and/or treatment delays.

The same dose adjustment rules apply for the phase I and phase II part of the trial.

7.1. General Remarks

- For any concomitant conditions already apparent at baseline, the dose modifications will apply according to the corresponding shift in toxicity grade, if the investigator feels it is appropriate. For example, if a patient has grade 1 asthenia at baseline that increases to grade 2 during treatment, this will be considered a shift of one grade and treated as grade 1 toxicity for dose-modification purposes.
- When several toxicities with different grades of severity occur at the same time, the dose modifications should be according to the highest grade observed.
- If, in the opinion of the investigator, a toxicity is due solely to one chemotherapeutic agent of the study treatment (e.g., carboplatin) and the dose of that drug is delayed or modified in accordance with the guidelines below, the other chemotherapeutic agent may be administered if there is no contraindication. If the toxicity is attributed to immunotherapy and the patient is treated with durvalumab and oleclumab, both drugs should be interrupted or discontinued. The only exception is for infusion-related reaction (IRR) clearly attributed to one of the two immunotherapeutic drugs, patient should continue the other drug (durvalumab or oleclumab) as scheduled.

- If the dose of one drug in the regimen is delayed, treatment with the other drug(s) may continue as scheduled.
- If the toxicity may be attributed to both immunotherapy and chemotherapy, durvalumab/oleclumab should be interrupted or discontinued and chemotherapy should be reduced (if applicable), interrupted, or discontinued.
- Definite discontinuation of one chemotherapeutical agent due to AEs while the other part will be kept is allowed.
- Dose reductions of durvalumab and oleclumab are not permitted.

7.2. Dose-limiting Toxicities of oleclumab (phase I part)

Grading of DLTs will be according to the National Cancer Institute Common Terminology Criteria for Adverse Events (NCI CTCAE) V5.0.

A DLT is defined as any Grade 3 or higher treatment-related toxicity that occurs during the DLT-evaluation period (defined as the time from receiving the first dose of oleclumab until the planned administration of the third dose, *see section 3.1. Phase I*). All DLTs must be documented as AEs. Toxicity that is clearly and directly related to the primary disease or to aetiology is excluded from this definition.

The following will be DLTs, regardless of relatedness:

- Any Grade 4 immune-related AE
- Any ≥ Grade 3 colitis
- Any ≥ Grade 3 renal failure/nephritis
- Any ≥ Grade 3 non-infectious pneumonitis irrespective of duration
- Any Grade 3 immune-related AE, excluding colitis, renal failure/nephritis and pneumonitis, that does not downgrade to ≤ Grade 2 within 3 days after onset of the event despite maximal medical supportive care including systemic corticosteroids or does not downgrade to ≤ Grade 1 or baseline within 14 days
- Liver transaminase elevation ≥ 5 ULN or total bilirubin > 3 × ULN will be considered a DLT regardless of duration or reversibility
- Any increase in AST or ALT > 3 × ULN and concurrent increase in total bilirubin > 2 × ULN

The definition of DLT excludes the following conditions:

- Grade 4 asymptomatic lipase/amylase elevation with no radiographic evidence of pancreatitis
- Grade 3 fatigue that improves by at least 1 grade within 14 days
- Grade 3 endocrine disorder (thyroid, pituitary, and/or adrenal insufficiency) that can be managed with or without systemic corticosteroid therapy and/or hormone replacement therapy
- Grade 3 inflammatory reaction attributed to a local antitumour response (e.g., inflammatory reaction at sites of metastatic disease, lymph nodes, etc.) that resolved to ≤ Grade 1 within 30 days
- Concurrent vitiligo or alopecia of any AE grade
- Grade 3 IRR (first occurrence and in the absence of steroid prophylaxis) that resolves within 6 hours with appropriate clinical management.
- Grade 3 or Grade 4 neutropenia ≤ 7 days
- Grade 3 or Grade 4 lymphopenia
- Grade 3 or Grade 4 anemia
- Grade 3 or Grade 4 thrombocytopenia ≤ 7 days that is not associated with clinically significant bleeding that requires medical intervention
- Isolated Grade 3 electrolyte abnormalities that are not associated with clinical signs or symptoms and are reversed with appropriate maximal medical intervention within 3 days
- Grade 3 fever lasting ≤ 24 hours with or without medical therapy

Immune-related AEs (irAEs) are defined as AEs of immune nature (i.e. inflammatory) in the absence of a clear alternative etiology that is not related to the investigational product. In the absence of clinical abnormality, repeated laboratory testing will be conducted to confirm significant laboratory findings prior to designation as a DLT. An AE not listed above may be defined as a DLT after consultation with the sponsor and investigators, based on the emerging safety profile.

Subjects who develop a DLT will stop study treatment permanently.

7.3. Durvalumab and oleclumab (phase I and II)

7.3.1. Immune-related Adverse Events

Immune-mediated or related AEs (im or irAEs) may occur after the first or several months after the last dose of durvalumab/oleclumab. Table hereunder describes the management of specific immune-related adverse events and when to withhold, discontinue and re-initiate durvalumab or durvalumab and oleclumab if the patient is treated with both immunotherapeutic drugs. In general, withhold immunotherapy for severe (Grade 3) imAEs. Permanently discontinue immunotherapy for life-threatening (Grade 4) irAEs, recurrent severe (Grade 3) irAEs that require systemic immunosuppressive treatment, or an inability to reduce corticosteroid dose to 10 mg or less of prednisone or equivalent per day within 12 weeks of initiating corticosteroids. After 12 weeks of treatment interruption for irAE, rechallenge with durvalumab +/- oleclumab should be discussed with the sponsor. **For more detailed guidance, please refer to the management guidelines for irAEs in the Appendix 1.**

Note: Consider calcium and vitamin D supplementation with prolonged steroid use. All patients receiving steroids should also be on proton pump inhibitor therapy for gastrointestinal prophylaxis. With long-term steroid and other immunosuppressive use, consider need for Pneumocystis jirovecii pneumonia (PJP) prophylaxis.

Table 2 Guidance for specific Immune-related Adverse Events

TOXICITY	GRADE	DESCRIPTION	MANAGEMENT
**Colitis**	1	Asymptomatic; clinical or diagnostic observations only; intervention not indicated [Grade 1 diarrhea frequency ≤ 4/day]	**Continue durvalumab/oleclumab**<br>Consider symptomatic treatment.
	2	Abdominal pain; mucus or blood in stool [Grade 2 diarrhea frequency 4–6/day]	**Hold durvalumab/oleclumab**<br>- Promptly start corticosteroids. Infliximab may be needed.<br>- If patient improves: taper corticosteroid and resume ICI.
	3-4	Grade 3: Severe abdominal pain; change in bowel habits; medical intervention indicated; peritoneal signs [Grade 3 diarrhea frequency ≥ 7×/day]<br>Grade 4: Life-threatening consequences; urgent intervention indicated	**Permanently discontinue durvalumab/oleclumab**<br>Start IV methylprednisolone. Infliximab may be needed.
**Hepatitis**	1	AST, ALT < ULN -3xULN and/or total bilirubin < ULN-1.5xULN	**Continue durvalumab/oleclumab**
	2	AST, ALT >3- ≤ 5xULN and/or total bilirubin > 1.5 - ≤ 3xULN	**Hold durvalumab/oleclumab**<br>- If event is persistent or worsens, promptly start corticosteroids.

TOXICITY	GRADE	DESCRIPTION	MANAGEMENT
			Mycophenolate mofetil may be needed. Infliximab should not be used.<br>- If patient improves: Taper corticosteroid and resume ICI.
	3-4	AST, ALT >5xULN and/or total bilirubin >3xULN	Start IV methylprednisolone. Mycophenolate mofetil may be needed.<br>Infliximab should not be used<br>For elevations in transaminases ≤8×ULN, and/or elevations in total bilirubin ≤5×ULN:<br>- Hold durvalumab/oleclumab until resolution to Grade≤1 or baseline<br>- Resume durvalumab/oleclumab if elevations downgrade to Grade≤1 or baseline within 14 days and after completion of steroid taper.<br>- Permanently discontinue durvalumab/oleclumab if the elevations do not downgrade to Grade≤1 or baseline within 14 days.<br>For elevations in transaminases >8×ULN or elevations in bilirubin >5×ULN, permanently discontinue durvalumab/oleclumab
	Hy's law	AST's and/or ALT >3 × ULN **AND** total bilirubin >2 × ULN without initial findings of cholestasis and in the absence of any alternative cause	Permanently discontinue durvalumab/oleclumab<br>Start IV methylprednisolone. Mycophenolate mofetil may be needed.<br>Infliximab should not be used
**Hypophysitis**	1	Asymptomatic or mild symptoms; clinical or diagnostic observations only; intervention not indicated	**Continue durvalumab/oleclumab**
	2	Moderate; minimal, local or noninvasive intervention indicated; limiting age-appropriate instrumental ADL	**Hold durvalumab/oleclumab**<br>- Consider corticosteroids.<br>- Consider drug re-challenge if the event is stabilized and controlled.
	3	Severe or medically significant but not immediately life-threatening; hospitalization or prolongation of existing hospitalization indicated; disabling; limiting self-care ADL	**Hold durvalumab/oleclumab**<br>- Promptly initiate empiric IV methylprednisolone as well as relevant hormone replacement.<br>- Consider drug re-challenge if the event is stabilized and controlled.
	4	Life-threatening consequences; urgent intervention indicated	**Permanently discontinue durvalumab/oleclumab**<br>Promptly initiate empiric IV methylprednisolone, as well as relevant hormone replacement.
**Hypothyroidism**	1	Asymptomatic; clinical or diagnostic observations only; intervention not indicated	**Continue durvalumab/oleclumab**

TOXICITY	GRADE	DESCRIPTION	MANAGEMENT
	2	Symptomatic; thyroid replacement indicated; limiting instrumental ADL	**Continue durvalumab/oleclumab**<br>Start standard thyroid replacement therapy
	3	Severe symptoms; limiting self-care ADL; hospitalization indicated	**Hold durvalumab/oleclumab**<br>- Start standard thyroid replacement therapy.<br>- Consider drug re-challenge after resolution of symptoms to grade ≤2.
	4	Life-threatening consequences; urgent intervention indicated	**Permanently discontinue durvalumab/oleclumab**<br>Start standard thyroid replacement therapy.
**Hyperthyroidism**	1	Asymptomatic; clinical or diagnostic observations only; intervention not indicated	**Continue durvalumab/oleclumab**
	2	Symptomatic; thyroid suppression therapy indicated; limiting instrumental ADL	**Hold durvalumab/oleclumab**<br>- Consider corticosteroids.<br>- Standard therapy for hyperthyroidism should be followed.<br>- Consider drug re-challenge if the event is stabilized and controlled.
	3	Severe symptoms; limiting self-care ADL; hospitalization indicated	**Hold durvalumab/oleclumab**<br>- Promptly initiate empiric IV methylprednisolone.<br>- Standard therapy for hyperthyroidism should be followed<br>- Consider drug re-challenge if the event is stabilized and controlled.
	4	Life-threatening consequences; urgent intervention indicated	**Permanently discontinue durvalumab/oleclumab**<br>- Promptly initiate empiric IV methylprednisolone.<br>- Standard therapy for hyperthyroidism should be followed.
**Pneumonitis**	1	Asymptomatic; clinical or diagnostic observations only	Consider holding durvalumab/oleclumab<br>Reimage (chest CT) prior next cycle
	2	Symptomatic; limiting instrumental ADL; medical intervention indicated	**Hold durvalumab/oleclumab**<br>- Promptly start corticosteroids. Infliximab may be needed.<br>- Chest CT with contrast (if possible) and repeat in 3-4 weeks<br>- Consider drug re-challenge if symptoms resolve to grade ≤ 1 and after steroid taper.
	3-4	Grade 3: Severe symptoms; limiting self-care ADL; oxygen indicated<br><br>Grade 4: Life-threatening respiratory compromise; urgent intervention indicated (e.g., intubation)	**Permanently discontinue durvalumab/oleclumab**<br>Promptly initiate IV methylprednisolone. Infliximab may be needed.

TOXICITY	GRADE	DESCRIPTION	MANAGEMENT
**Nephritis or renal dysfunction**	1	Creatinine level increase of >0.3 mg/dL; serum creatinine > ULN to 1.5 × ULN	**Continue durvalumab/oleclumab**
	2	Serum creatinine >1.5 to 3.0 × baseline; >1.5 to 3.0 × ULN	**Hold durvalumab/oleclumab**<br>- Monitor creatinine.<br>- If event is persistent or worsens, promptly start corticosteroids PO or IV.<br>- Resume when creatinine decreased to ≤ grade 1<br>- Discontinue durvalumab/oleclumab for persistent or recurrent elevation
	3-4	Grade 3: serum creatinine >3.0 × baseline; >3.0 to 6.0 × ULN<br><br>Grade 4: Life-threatening consequences; serum creatinine >6.0 × ULN	**Permanently discontinue durvalumab/oleclumab**<br>- Promptly start corticosteroids.- Consider immunosuppressive treatment after consultation with a nephrologist
**Rash (no bullous skin formations)**	1	Depending on the rash, please see CTCAE v5.0	**Continue durvalumab/oleclumab**<br>- Oral antihistamines<br>- Topical corticosteroids
	2	Depending on the rash, please see CTCAE v5.0	**Continue durvalumab/oleclumab**<br>- Oral antihistamines<br>- Topical corticosteroids<br>- ICIs may need to be held and systemic corticosteroids initiated.<br>- If event is persistent or worsens hold durvalumab/oleclumab and start corticosteroids
	3	Depending on the rash, please see CTCAE v5.0	**Hold durvalumab/oleclumab**<br>- Oral antihistamines<br>- Promptly initiate empiric IV methylprednisolone.<br>- Consider resuming durvalumab/oleclumab when corticosteroid is tapered and rash is grade ≤ 1.
	4	Depending on the rash, please see CTCAE v5.0	**Permanently discontinue durvalumab/oleclumab**<br>- Oral antihistamines<br>- Initiate IV methylprednisolone

7.3.2. Infusion-related Reactions

Infusion-related reactions (IRRs) can be observed with both durvalumab and oleclumab.

In case of a Grade 2 IRR that resolves within one hour of stopping the drug infusion and adequate treatment according to local standard of care, the infusion may be restarted at 50% of the original infusion rate. In case of Grade 3-4 IRR, the study drug has to be discontinued permanently.

Table 3 Management of IRRs to durvalumab/oleclumab

NCI CTCAE Grade	Management	Premedication at subsequent dosing
Grade 1	The infusion rate may be decreased by 50% or temporarily interrupted.<br>Increase monitoring of vital signs until the subject is deemed medically stable in the opinion of the investigator.	None
Grade 2	Stop infusion and monitor symptoms<br>Additional medical therapy may include but is not limited to<br>• IV fluids<br>• Antihistamines<br>• Hydrocortisone/ other corticosteroids<br>• NSAIDS<br>• Paracetamol<br>• Narcotics<br>Increase monitoring of vital signs until the subject is deemed medically stable in the opinion of the investigator.<br>If symptoms resolve within 1 hour of stopping drug infusion, the infusion may be restarted at 50% of the original infusion rate. Otherwise, dosing will be held until symptoms resolve and the subject should be premedicated for the next scheduled visit.	Premedication may include<br>• Antihistamines<br>• Promethazine/ diphenhydramine<br>• Paracetamol<br>• NSAIDS<br>Steroids should not be used for routine premedication of Grade ≤ 2 infusion reactions<br><br>Subjects who develop Grade 2 toxicity despite adequate premedication should be permanently discontinued from further study treatment administration (durvalumab or oleclumab).
Grade 3-4	Stop infusion<br>Additional appropriate medical therapy may include but is not limited to:<br>• IV fluids<br>• Antihistamines<br>• Hydrocortisone/ other corticosteroids<br>• NSAIDS<br>• Paracetamol<br>• Narcotics<br>• Oxygen<br>• Pressors (eg: epinephrine)<br>• Increase monitoring of vital signs until the subject is deemed medically stable in the opinion of the investigator.<br>Hospitalization may be indicated.	Permanently discontinue durvalumab or oleclumab

7.4. Chemotherapy

Chemotherapy administration (paclitaxel and carboplatin) may be interrupted due to AEs for a maximum of 4 weeks. If the patient does not meet the re-treatment criteria within this time period, chemotherapy has to be discontinued permanently. In case of treatment interruption ≤ 4 weeks, treatment with chemotherapy might go on for a maximum of 12 administrations of paclitaxel and carboplatin (i.e. in this context chemotherapy will be administered even after week 12).

Suggested dose modifications for chemotherapy are detailed in the table hereunder.

Table 4 Suggested Dose Modifications for Chemotherapy

	Start Dose	Dose Reduction 1	Dose Reduction 2
**Paclitaxel**	80 mg/m^2	65 mg/m^2	50 mg/m^2
**Carboplatin**	AUC 1.5	AUC 1.0	Discontinue

7.4.1. Hematologic Toxicities

Prior to initial treatment, the following must apply:

- Absolute neutrophil count (ANC) ≥ 1500/µl but ≥ 1000/µl prior to subsequent cycles and
- Platelet count > 100000/µl (prior to first cycle) and ≥ 75000/µl prior to subsequent cycles

If the counts are below, chemotherapy should not be administered and will be postponed for at least 7 days. When counts recover, resume dosing with applicable reductions according to criteria in the table hereunder.

Table 5 Chemotherapy Dose Suggested Adjustments due to Hematologic Toxicity

**Toxicity**	**Occurrence**	**Subsequent Paclitaxel Dose**	**Subsequent Carboplatin Dose**
Neutropenic fever<br>(ANC < 1000/µl and fever > 38°C)<br>OR<br>Delay of the next chemotherapy administration by > 7 days for nadir ANC < 1000/µl	First	80 mg/m^2	Discontinue
	Second	65 mg/m^2	Keep discontinued
	Third	Discontinue	Keep discontinued
Delay of the next chemotherapy administration by </= 7 days for nadir ANC < 1000/µl<br>-------------------------------------<br>*Treatment interruption of paclitaxel and carboplatin until ANC ≥ 1000/µl*	First	80 mg/m^2	AUC 1.0
	Second	65 mg/m^2	Discontinue
	Third	Discontinue	Keep discontinued
Nadir platelet count<br><75000/µl but ≥ 50000/µl<br>-------------------------------------<br>*Treatment interruption of paclitaxel and carboplatin until platelets ≥75000/µl*	First	80mg/m^2	AUC 1.0
	Second	65 mg/m^2	Discontinue
	Third	50 mg/m^2	Keep discontinued
Platelet Count < 50000/µl<br>-------------------------------------<br>*Treatment interruption of paclitaxel and carboplatin until platelets ≥75000/µl*	First	65 mg/m^2	Discontinue
	Second	50 mg/m^2	Keep discontinued
	Third	Discontinue	Keep discontinued

In case of ANC < 1500/µl, consider G-CSF to avoid delay of the next chemotherapy.

In case of ANC < 1000/µl, patients must be closely followed for signs of infections and encouraged to seek immediate medical help if they present fever, worsening of their general condition, sore throat, cough, dysuria or other signs of infection.

7.4.2. Gastrointestinal Toxicities

Gastrointestinal toxicities in terms of colitis/diarrhoea have been reported with taxane-based chemotherapy and are less-common with carboplatin.

For Grade 3 or 4 gastrointestinal toxicities, chemotherapy treatment should be delayed until resolution to less than or equal to the patient's baseline value. Dose reductions at the start of the subsequent cycle will be based on gastrointestinal toxicities from the dose administered in the preceding cycle.

Table 6 Chemotherapy Dose Reduction due to Gastrointestinal Toxicities

**Diarrhea**	**Occurrence**	**Subsequent Paclitaxel Dose**	**Subsequent Carboplatin Dose**
Grade 3<br>-------------------------------------<br>*Treatment interruption of paclitaxel and carboplatin until resolution to less than or equal to the patient's baseline value.*	First	65 mg/m^2	AUC 1.0
	Second	50 mg/m^2	Discontinue
	Third	Discontinue	Keep discontinued

Diarrhea	Occurrence	Subsequent Paclitaxel Dose	Subsequent Carboplatin Dose
Grade 4<br>--------------------------------------<br>*Treatment discontinuation of paclitaxel and interruption of carboplatin until resolution to less than or equal to the patient's baseline value.*	First	Discontinue	AUC 1.0
	Second	Keep discontinued	Discontinue

7.4.3. Neurological Toxicities

Paclitaxel should be withheld for Grade 3 peripheral neuropathy and may be resumed at reduced doses when peripheral neuropathy recovers to less than or equal to the patient's baseline value. In case of Grade 4 neuropathy, paclitaxel needs to be stopped permanently.

Table 7 Chemotherapy Dose Reduction due to Neurological Toxicities

Peripheral Neuropathy	Occurrence	Subsequent Paclitaxel Dose	Subsequent Carboplatin Dose
Grade 2<br>--------------------------------------<br>*No treatment interruption*		65 mg/m^2; if persistent reduce to 50 mg/m^2	Keep AUC 1.5, if persistent consider dose reduction
Grade 3<br>--------------------------------------<br>*Treatment interruption of paclitaxel and carboplatin until resolution to less than or equal to the patient's baseline value.*	First	65 mg/m^2	AUC 1.5, if persistent consider dose reduction
	Second	50 mg/m^2	AUC 1.0
	Third	Discontinue	Discontinue
Grade 4<br>--------------------------------------<br>*Treatment discontinuation*	First	Discontinue	Discontinue

7.4.4. Hepatic Toxicities

Table 8 Chemotherapy Dose Reduction due to Hepatic Toxicities

Toxicity	Occurrence	Subsequent Paclitaxel Dose	Subsequent Carboplatin Dose
ALT/AST > 3 x ULN<br>and<br>Bilirubin > ULN to ≤ 1.5 x ULN<br>--------------------------------------<br>*Treatment interruption of paclitaxel and carboplatin until ALT/AST ≤ 3 x ULN*	First	65 mg/m^2	AUC 1.5
	Second	50 mg/m^2	AUC 1.5
	Third	Discontinue	Discontinue
Independent of ALT/AST<br>Bilirubin > 1.5 x ULN to ≤ 3 x ULN<br>--------------------------------------<br>*Treatment interruption of paclitaxel and carboplatin until ALT/AST ≤ 3 x ULN and Bilirubin ≤ 1.5 x ULN*	First	50 mg/m^2	AUC 1.5
	Second	Discontinue	Discontinue
Independent of ALT/AST<br>Bilirubin > 3 x ULN<br>--------------------------------------<br>*Treatment discontinuation*	First	Discontinue	Discontinue

7.4.5. Renal Toxicities

The targeted dose of carboplatin (AUC 2 for phase I and AUC 1.5 for phase II) should always be calculated based on the current (i.e. determined prior to the administration of

chemotherapy) estimated glomerular filtration rate (eGFR). If there is a significant increase in creatinine (> 120% of the baseline value), an exact method (e.g. nuclear renogram, if available) to determine the GFR is advisable. Carboplatin and paclitaxel should be interrupted in case of GFR < 60 ml/min.

7.4.6. Infusion-related Reactions (IRRs)

Hypersensitivity to paclitaxel and/ or carboplatin is common. While symptoms usually develop within the first 10-15 minutes of paclitaxel infusion and occur in nearly 90% of the cases during the first or second drug infusion, the incidence of allergic reactions with carboplatin increases with repeated drug exposure. To avoid allergic reactions, premedication with antihistamines and corticosteroids is mandatory and should be followed as per local standard of care. The table hereunder outlines the management of allergic reactions with paclitaxel and/or carboplatin.

Table 9 Management of IRRs to Paclitaxel/ Carboplatin

**NCI CTCAE Grade**	**Treatment**	**Premedication at subsequent dosing**
Grade 1	Infusion interruption not indicated<br>Increase monitoring of vital signs until the subject is deemed medically stable in the opinion of the investigator.	According to local standard of care.
Grade 2	Stop infusion and monitor symptoms<br>Additional medical therapy may include but is not limited to<br>• IV fluids<br>• Antihistamines<br>• Hydrocortisone<br>• NSAIDS<br>• Paracetamol<br>• Narcotics<br>Increase monitoring of vital signs until the subject is deemed medically stable in the opinion of the investigator.<br>If symptoms resolve within 1 hour of stopping drug infusion, the infusion may be restarted at 50% of the original infusion rate. Otherwise, dosing will be stopped.	According to local standard of care.
Grade 3-4	Stop infusion<br>Additional appropriate medical therapy may include but is not limited to<br>• IV fluids<br>• Antihistamines<br>• Hydrocortisone/ other corticosteroids<br>• NSAIDS<br>• Paracetamol<br>• Narcotics<br>• Oxygen<br>• Pressors (e.g; : Epinephrine)<br>Increase monitoring of vital signs until the subject is deemed medically stable in the opinion of the investigator.<br>Hospitalization may be indicated.<br>Subject is permanently discontinued from further administration of the respective drug.	No subsequent dosing of the respective drug.

8. SCHEDULE OF ASSESSMENTS

8.1. Screening period

During the screening period (28 days prior enrolment), the principal investigator confirms the subject’s eligibility for the study by conducting the assessments described below.

All screening evaluations are to be performed within 28 days before enrolment except if described differently in the text.

Following evaluations and procedures have to be conducted in the screening period:

- ICF signature to be obtained prior any study specific procedures (patient to be registered in Subject Registration Tool = step 1)
- Demographic data: gender, date of birth
- Eligibility check: review of inclusion and exclusion criteria (eligible patient to be enrolled in Subject Registration Tool = step 2)
- Medical history: prior and/or clinically significant current medical conditions and surgical procedures, assessed inherited mutations in breast cancer related genes (*BRCA1*, *BRCA2*).
- Cancer history and prior cancer therapies specifying name of therapy/procedure, total cumulative dose, date of treatment and number of cycles administered when available for each past treatment
- Concomitant medications, therapies and procedures have to be documented from 28 days prior to the enrolment
- Medical consultation including:
  - Physical examination
  - Vital signs (body temperature, blood pressure, pulse (heart rate))
  - Body weight, height, body surface area (DuBois method)
  - ECOG performance status
- Serum pregnancy test for all female subjects of childbearing potential
- Laboratory tests including:
  - Complete blood count (CBC) and analysis should be performed: red blood cell count (RBC), haemoglobin (Hb), platelet count and white blood cell count (WBC). WBC must include differential count
  - Chemistry panel: 25-OH vitamin D, glucose, urea, creatinine, eGFR (according to institutional standard of care), sodium, potassium, magnesium, chloride, bicarbonate, calcium, phosphorus, total serum bilirubin, ALT, AST, ALP, LDH, total protein, and albumin
- Other laboratory tests:
  - Coagulation: aPTT and INR
  - Blood tumour markers: CA 15-3 and carcinoembryonic antigen (CEA)
  - Thyroid function testing (thyroid-stimulating hormone [TSH], T4)
  - HBV serology (HBsAg, antibody to HBsAg [anti-HBs], anti-HBc). HBV DNA testing is required if the patient has negative serology for HBsAg and positive serology for anti-HBc
  - HCV serology (Qualitative HCV-RNA testing is required if the patient has positive serology)
  - Lipase. In case of lipase increase or suspicion of pancreatitis, amylase should be evaluated
- Echocardiography for all patients previously treated with anthracyclines who do not have a recent cardiac work up (< 6 months)
- SAEs related to protocol-mandated intervention
- Baseline tumour burden assessment: Contrast-enhanced CT scan of the chest, the abdomen and the pelvis. MRI of the chest, the abdomen and the pelvis is an alternative to the contrast-enhanced CT scan and can be chosen per the investigator’s choice. Baseline imaging must be performed within 14 days before starting treatment

Imaging performed before IC signature can be used as baseline if it was performed within 14 days before starting study drug. If no contrast-enhanced CT scan was performed, this exam (or an MRI) must be done before start of study treatment

- Provision of archived FFPE diagnostic biopsy or surgical primary tumour sample (or at least 20 slides unstained, freshly cut for the purposes of the study). In case of neoadjuvant treatment (before surgery), the diagnostic biopsy is preferable. *See section 11. Translational Research(es)*
- Metastatic lesion biopsy, *see section 11. Translational Research(es)*
- **Only for phase II part**: **central assessment of PD-L1 and CD73 status required prior randomization for stratification purposes.** PD-L1 and CD73 status will be assessed using IHC on the screening FFPE tumour lesion biopsy (or on the archived FFPE primary tumour sample in case the tumour lesion biopsy tissue sample is not evaluable for PD-L1 or CD73) by a central laboratory prior to randomization for stratification purposes. Samples should be sent **minimum 10 days** before planned date of first study drug administration. Patients whose tumour tissue is not evaluable for PD-L1 and CD73 will not be eligible. All sites and study team staff will be blinded to PD-L1 and CD73 status

Results of all screening evaluations and procedures must be reviewed by the principal investigator to ensure that all eligibility criteria have been satisfied prior to subject enrolment.

8.2. Study treatment period

8.2.1. Chemotherapy in combination with immunotherapy (= during the first 12 weeks)

A maximum of 3 days is allowed between subject's enrolment and first study drug administration.
*Evaluations with * do not need to be repeated at week 1 if done within 3 days prior first study drug administration at screening.*

- Treatment administration
- Concomitant medications, therapies and procedures
- Medical consultation (within 3 days prior study drug administration) including:
  - Physical examination* every 2 weeks
  - Vital signs* (body temperature, blood pressure, pulse (heart rate)) every 2 weeks
  - Body weight*, body surface area* (DuBois method) every week
  - ECOG performance status* every 2 weeks
- Urine pregnancy test (if positive confirm by serum test) for all female subjects of childbearing potential every 4 weeks (± 7 days) starting at week 5. Note: A serum pregnancy test is accepted as well.
- Laboratory tests* (within 3 days prior study drug administration) every week including:
  - CBC including RBC, Hb, platelet count, WBC (including differential count)
  - Chemistry panel: glucose, urea, creatinine, eGFR (according to institutional standard of care), sodium, potassium, magnesium, chloride, bicarbonate, calcium, phosphorus, total serum bilirubin, ALT, AST, ALP, LDH, total protein, and albumin.
- Other laboratory tests:
  - Coagulation*: aPTT and INR every 8 weeks (within 3 days prior study drug administration) starting at week 9
  - Blood tumour markers (CA 15-3 and CEA) every 8 weeks starting at week 9 (within 3 days prior study drug administration) is only indicated in patients who had elevated markers at baseline (as defined by local laboratory guidelines)
  - Thyroid function testing (thyroid-stimulating hormone [TSH], T4) every 4 weeks (± 7 days) starting at week 5

  - Lipase every 4 weeks (± 7 days) starting at week 5. In case of lipase increase or suspicion of pancreatitis, amylase should be evaluated
- All AEs including SAEs documented according to NCI-CTCAE v5.0 and AESIs, pregnancies, adverse reactions due to overdose, abuse, misuse and medication error, *as described in section 10.2. Pharmacovigilance parameters definitions and reporting guidance*
- Tumour assessment: Contrast-enhanced CT scan or MRI of all known sites with metastatic disease at week 8 (± 3 days). Tumour imaging assessments recommendations are described in *section 3.3.7 Imaging assessment*
- Metastatic lesion biopsy during week 3 before the second administration of durvalumab (applicable in both arms), for arm A it must be performed after the second administration of oleclumab (*see section 11. Translational Research(es)*). If feasible the preferred option is to biopsy the same metastatic lesion biopsied at screening. Haematological and coagulation criteria should meet local clinical standards according to the procedure.
- Blood samples at week 1 and during week 3 before the second administration of durvalumab (applicable in both arms), for arm A it must be performed after the second administration of oleclumab (*see section 11. Translational Research(es)*). If feasible the blood sample for TR research should be performed at the same time as the biopsy.

8.2.2. Maintenance treatment with immunotherapy (= after chemotherapy until the end of treatment)

8.2.2.1. *From end of chemotherapy until week 104*

- Treatment administration
- Concomitant medications, therapies and procedures
- Medical consultation (within 3 days prior to study drug administration) at the first immunotherapy visit (end of chemotherapy), then every 4 weeks until week 25, every 8 weeks until week 52 and every 12 weeks thereafter, including:
  - Physical examination
  - Vital signs (body temperature, blood pressure, pulse [heart rate])
  - Body weight
  - ECOG performance status
- Urine pregnancy test (if positive confirm by serum test) for all female subjects of childbearing potential every 4 weeks (+/- 7 days). Note: Serum pregnancy test is accepted as well.
- Laboratory tests (within 3 days prior to study drug administration) at the first immunotherapy visit (end of chemotherapy), every 4 weeks until week 52 and every 8 weeks thereafter, including:
  - CBC including RBC, Hb, platelet count, WBC (including differential count)
  - Chemistry panel: glucose, urea, creatinine, sodium, potassium, magnesium, chloride, bicarbonate, calcium, phosphorus, total serum bilirubin, ALT, AST, ALP, LDH, total protein, and albumin
- Other laboratory tests:
  - Coagulation: aPTT and INR every 8 weeks until week 52 and every 12 weeks thereafter (within 3 days prior to study drug administration)
  - Blood tumour markers (CA 15-3 and CEA) every 8 weeks until week 52 and every 12 weeks thereafter (within 3 days prior to study drug administration) is only indicated in patients who had elevated markers at baseline (as defined by local laboratory guidelines)

  - Thyroid function testing (thyroid-stimulating hormone [TSH], T4) at the first immunotherapy visit (end of chemotherapy), then every 4 weeks until week 25 and every 8 weeks thereafter (+/- 7 days)
  - Lipase at the first immunotherapy visit (end of chemotherapy), then every 4 weeks until week 25 and every 8 weeks thereafter (+/- 7 days). In case of lipase increase or suspicion of pancreatitis, amylase should be evaluated.
- All AEs including SAEs documented according to NCI-CTCAE v 5.0 and AESIs, pregnancies, adverse reactions due to overdose, abuse, misuse, and medication error, *as described in section 10.2. Pharmacovigilance parameters definitions and reporting guidance*
- Tumour assessment: Contrast-enhanced CT scan or MRI of all known sites with metastatic disease at weeks 16 (± 3 days) and 24 (± 3 days). Thereafter imaging studies will be done every 12 weeks (± 3 days) during the first year. Tumour imaging assessments recommendations are described *section 3.3.7 Imaging assessment*.

  After week 24, an additional medical visit with the patient physician should be planned after each tumour assessment
- Optional metastatic lesion biopsy if progression disease based on RECIST v1.1 (*see section 11. Translational Research(es)*)
- Blood samples at the first immunotherapy visit (end of chemotherapy treatment, if no delay week 13), at week 25, at first progression based on RECIST v1.1 and at first occurrence of possible immune related toxicity (*see section 11. Translational Research(es)*)

8.2.2.2. *From week 104 until end of treatment visit:*

- Treatment administration
- Concomitant medications, therapies and procedures
- Medical consultation (within 3 days prior to study drug administration) every 12 weeks including:
  - Physical examination
  - Vital signs (body temperature, blood pressure, pulse [heart rate])
  - Body weight
  - ECOG performance status
- Urine pregnancy test (if positive confirm by serum test) for all female subjects of childbearing potential every 4 weeks (+/- 7 days). Note: Serum pregnancy test is accepted as well.
- Laboratory tests as per local standard of care
- Other laboratory tests:
  - Thyroid function testing (thyroid-stimulating hormone [TSH], T4) every 8 weeks thereafter (+/- 7 days)
- All AEs including SAEs documented according to NCI-CTCAE v 5.0 and AESIs, pregnancies, adverse reactions due to overdose, abuse, misuse, and medication error, *as described in section 10.2. Pharmacovigilance parameters definitions and reporting guidance*
- Tumour assessment: Contrast-enhanced CT scan or MRI of known sites with metastatic disease will be performed in accordance with local standard of care for assessment of response or disease progression.
- Optional metastatic lesion biopsy if progression disease based on RECIST v1.1 (*see section 11. Translational Research(es)*)

- Blood samples at first progression and at first occurrence of possible immune related toxicity (*see section 11. Translational Research(es)*)

8.3. End of treatment visit

When a subject discontinues study treatment, an end of treatment (EOT) visit should be carried out **28 days (± 7 days) after the last study treatment administration or before starting any new anticancer treatment, whatever comes first.** The following evaluations and procedures need to be performed:

- Concomitant medications, therapies and procedures
- Medical consultation including:
  - Physical examination
  - Vital signs (body temperature, blood pressure, pulse (heart rate))
  - Body weight
  - ECOG performance status
- Urine pregnancy test (if positive confirm by serum test) for all female subjects of childbearing potential. Note: A serum pregnancy test is allowed as well.
- Laboratory tests including:
  - CBC including RBC, Hb, platelet count, WBC (including differential count)
  - Chemistry panel: glucose, urea, creatinine, sodium, potassium, magnesium, chloride, bicarbonate, calcium, phosphorus, total serum bilirubin, ALT, AST, ALP, LDH, total protein, and albumin
- Other laboratory tests:
  - Coagulation: aPTT and INR
  - Blood tumour markers (CA 15-3 and CEA) only indicated in patients who had elevated markers at baseline (as defined by local laboratory guidelines)
  - Thyroid function testing (thyroid-stimulating hormone [TSH], T4)
  - Lipase. In case of lipase increase or suspicion of pancreatitis, amylase should be evaluated.
- All AEs including SAEs documented according to NCI-CTCAE v5.0 and AESIs, pregnancies, adverse reactions due to overdose, abuse, misuse and medication error, *as described in section 10.2. Pharmacovigilance parameters definitions and reporting guidance.*

  Note: (S)AE(s)/AESI(s) should be collected and reported until 90 days after the last administration of the study drug. This should be obtained during any patient visit occurring after EOT visit.
- Tumour assessment: to be performed if last tumour assessment was performed more than 28 days before treatment stop date. If last tumour assessment was performed within 28 days of treatment stop date, no need to repeat this exam. Tumour imaging assessments recommendations are described in *section 3.3.7 Imaging assessment*.
- Optional metastatic lesion biopsy if progression disease based on RECIST v1.1 (*see section 11. Translational Research(es)*)
- Blood samples at progression disease based on RECIST v1.1 (*see section 11. Translational Research(es)*)

8.4. Efficacy Follow-up period

This section is only applicable for patients who stopped the study treatment for reasons other than PD.

The Efficacy Follow-up Period starts after the end of study treatment visit (EOT) and ends at disease progression, start of a new anti-cancer treatment, withdrawal of consent to study participation, death or end of the study whichever comes first. Only patients who interrupt study treatment due to intolerable toxicity or voluntary withdrawal from study treatment go into the efficacy follow-up period – other patients go directly to the survival follow-up period (see the following section).

The following assessments are planned every 12 weeks (± 7 days):

- Medical consultation including:
  - Physical examination
  - Vital signs (body temperature, blood pressure, pulse (heart rate))
  - ECOG performance status
- Urine pregnancy test (if positive confirm by serum test) for all female subjects of childbearing potential at 3 months and 7 months after end of study treatment
- Laboratory tests including:
  - CBC including RBC, Hb, platelet count, WBC (including differential count)
  - Chemistry panel: glucose, urea, creatinine, sodium, potassium, magnesium, chloride, bicarbonate, calcium, phosphorus, total serum bilirubin, ALT, AST, ALP, LDH, total protein, and albumin
- All AEs including SAEs documented according to NCI-CTCAE v 5.0 and AESIs, pregnancies, adverse reactions due to overdose, abuse, misuse, and medication error, *as described in section 10.2. Pharmacovigilance parameters definitions and reporting guidance.*
- Tumour assessment: Patients who stopped all study treatments for reasons other than PD will continue post-treatment imaging studies (Contrast-enhanced CT scan or MRI of all known sites with metastatic disease) for disease status follow-up every 8 weeks (± 7 days) until week 24 and every 12 weeks (± 7 days) until week 104 and per standard local practice after week 104 until verified PD, start of a new anti-cancer treatment, withdrawal of consent to study participation, death or end of the study whichever comes first. Tumour imaging assessments recommendations are described in *section 3.3.7 Imaging assessment*.

8.5. Survival Follow-up Period

The Survival Follow-up Period starts:

- after the EOT visit for patients who have interrupted study treatment due to disease progression,
- at disease progression for patients that are coming from efficacy follow-up.

This period ends at withdrawal of consent to study participation, death (or inability to obtain further information on patient), or end of the study whichever comes first.

During this period there are no further visit but data on overall survival should be obtained every 6 months. Data on overall survival can be obtained during regular out of study clinical follow-up visits, via phone call or patient hospital records.

Data on AEs including SAEs documented according to NCI-CTCAE v 5.0 and AESIs, pregnancies, adverse reactions due to overdose, abuse, misuse, and medication error, *as must be reported to the sponsor as described in section 10.2. Pharmacovigilance parameters definitions and reporting guidance.*

8.6. Tables of Assessments

Table 10 Study Procedures during Screening and Treatment periods

Evaluations	Screening 28 days before enrolment	Study treatment period																
		Chemotherapy + immunotherapy												Immunotherapy (maintenance)				
		1[a]	2	3	4	5	6	7	8	9	10	11	12	First immunotherapy visit (week 13)	Weeks 14 to 25	Weeks 26 to 52	Week 53 to week 104	From week 104
															(starting from the last evaluation performed)			
ICF signature	X																	
Eligibility Criteria	X																	
Past medical and cancer history	X																	
Concomitant medication, therapies & procedures	X	X (from 28 days prior to the enrolment until EOT visit)																
Physical Examination [b] & Vital Signs & ECOG PS [c]	X	X*		X		X		X		X		X		X	Every 4 w	Every 8 w	Every 12 w	
Body Weight, Height, Body Surface Area [d]	X	X*	X	X	X	X	X	X	X	X	X	X	X	X	Every 4 w	Every 8 w	Every 12 w	
**Laboratory tests**																		
Pregnancy Test [e]	X					X				X				X	Every 4 w			
Complete Blood Count (CBC) [f]	X	X*	X	X	X	X	X	X	X	X	X	X	X	X	Every 4 w		Every 8 w	t
Chemistry [g]	X	X*	X	X	X	X	X	X	X	X	X	X	X	X	Every 4 w		Every 8 w	t
Coagulation [h]	X	X*								X					Every 8 w		Every 12 w	t
Blood Tumour markers [i]	X									X					Every 8 w		Every 12 w	
Thyroid Function Testing [j]	X					X				X				X	Every 4 w	Every 8 w		
Lipase	X					X				X				X	Every 4 w	Every 8 w		t
HBV/HCV Testing [k]	X																	
**Treatment administration [l]**																		
• Paclitaxel/Carboplatin		X	X	X	X	X	X	X	X	X	X	X	X					
• Durvalumab		X				X				X				Every 4 w				
• Oleclumab		X		X		X		X		X				Every 4 w				
Adverse Events [m]		X continuously (from 1st administration of study treatment until 90 days after last administration)																
SAEs / AESIs [m]	X	X continuously (from 1st administration of study treatment until 90 days after last administration)																

**Imaging**									
Echocardiography [n]	X								
Tumour assessment [o]	X	At weeks 8, 16 & 24						Every 12 w	[t]
**Samples collection**									
Primary tumour sample [p]	X								
Metastatic lesion biopsy [q]	X			X		Optional at progression (PD)			
**PD-L1 & CD73 status** [r]	X								
Blood Samples for TR [s]		X		X		X	At week 25	X (at PD)	

a. **Evaluations with * do not need to be repeated at week 1 if done within 3 days prior to first study drug administration at screening.**
b. Physical examination has to be done within 3 days prior to study drug administration.
c. Vital signs (body temperature, blood pressure, pulse (heart rate)) and ECOG PS have to be assessed within 3 days prior to study drug administration.
d. Height should be assessed only at screening. After the first 12 weeks, only body weight is asked.
e. A pregnancy test has to be done in all female subjects of childbearing potential at screening (by serum test) and starting at week 5, every 4 weeks (± 7 days) thereafter (by urine test and if positive to be confirmed by serum test).
f. Complete Blood Count (CBC) includes RBC, Hb, platelet count, WBC (including differential count) and has to be done within 3 days prior to study drug administration.
g. Chemistry has to be done within 3 days prior to study drug administration. It includes 25-OH vitamin D (only at screening), glucose, urea, creatinine, eGFR (according to institutional standard of care and only at screening and during the chemotherapy), sodium, potassium, magnesium, chloride, bicarbonate, calcium, phosphorus, total serum bilirubin, ALT, AST, ALP, LDH, total protein and albumin.
h. Coagulation includes aPTT and INR and has to be done within 3 days prior to study drug administration.
i. Blood Tumour markers (CA 15-3 and CEA) have to be done during screening. Re-assessment after the screening (within 3 days prior study drug) is only indicated in patients who had elevated markers at baseline (as defined by local laboratory guidelines).
j. Thyroid function: TSH and T4.
k. For HBV testing: HBsAg, anti-HBs, anti-HBc. HBV DNA testing is required if the patient has negative serology for HBsAg and positive serology for anti-HBc.
For HCV testing: qualitative HCV-RNA testing.
l. A maximum of 3 days are allowed between subject's enrolment and first study drug administration. Paclitaxel: 80 mg/m² IV once a week for 12 administrations; Carboplatin: AUC 2 IV (in phase I) and at a dose of AUC 1.5 IV (in phase II) once a week for 12 administrations; Durvalumab: 1500mg IV Q4W; Oleclumab: Only administered to patients of the phase I (3000mg/1500mg/750mg IV 5xQ2W then 3000mg/1500mg/750mg IV 1xQ4W followed by 3000mg Q4W) and to patients enrolled into arm A of the phase II (RP2D IV (3000 mg) 5xQ2W then RP2D IV Q4W)
m. From IC signature up to 1st study drugs administration, only SAEs related to protocol-mandated intervention have to be reported. From 1st study drugs administration up to 90 days after last administration of study drugs, all AEs & SAE/AESIs have to be reported. From 91 days after last administration of study drugs, only SAEs/AESIs related to study drugs must be reported.
n. Echocardiography for all subjects previously treated with anthracyclines who do not have a recent cardiac work up (< 6 months)
o. Contrast-enhanced CT scan or MRI of the chest, the abdomen and the pelvis must be performed within 14 days before starting treatment (for baseline imaging). Afterwards, contrast-enhanced CT scan or MRI of all known sites with metastatic disease are to be performed at week 8 (± 3 days), week 16 (± 3 days) and week 24 (± 3 days) and every 12 weeks (± 3 days) thereafter.

Remark for imaging procedures: The same radiographic procedures used to assess measurable disease sites at screening should be used throughout the study. All known sites of disease must be documented at screening and re-assessed at each subsequent tumour evaluation. Assessments should be performed by the same evaluator, if possible, to ensure internal consistency across visits.

After week 24, an additional medical visit with the patient physician should be planned after each tumour assessment.

Note 1: Patients who stopped all study treatments for reasons other than PD will continue post-treatment imaging studies for disease status follow-up every 8 weeks (± 7 days) until week 24 and every 12 weeks (± 7 days) thereafter until verified PD, start of a new anti-cancer treatment, withdrawal of consent to study participation, death, or end of the study whichever comes first.

Note 2: Pseudo progression related to immunotherapy: Patients experiencing PD as defined by RECIST v1.1 can continue the study treatment in case of good clinical condition assessed by a stable or even improved ECOG performance status. If the next assessment of tumour burden (8 weeks later) confirms PD (as defined by iRECIST) study treatment must be discontinued. Unconfirmed PD as defined by iRECIST (iUPD) can only be assigned for the 2 first imaging assessments (week 8 and week 16) and several times as long as confirmed progression (iCPD) is not confirmed at the next assessment. If PD is not confirmed, reassessments continue as originally planned.

p. Provision of an archived FFPE diagnostic biopsy or surgical primary tumour sample (or at least 20 unstained slides, freshly cut for the purposes of the study). In case of neoadjuvant treatment (before surgery), the diagnostic biopsy is preferable. (*see section 11. Translational Research(es)*).

q. Mandatory metastatic lesion biopsy (at least 1 FFPE and 1 frozen core as a priority. If feasible, 2 additional fresh tissue cores) at screening (prior to inclusion) and during week 3 before the second administration of durvalumab (applicable in both arms), for arm A, it must be performed after the second administration of oleclumab) (if feasible the preferred option is to biopsy the same metastatic lesion biopsied at screening. Haematological and coagulation criteria should meet local clinical standards according to the procedure) and optional biopsy at progression (based on RECIST v1.1). Acceptable tissues samples include those from resections, core-needle biopsies or excisional, incisional, punch, or forceps biopsies. Not acceptable tissue: FNA, brushing and cell pellets from cytology samples (*see section 11. Translational Research(es)*).

Note 1: At screening, if a metastatic/recurrent lesion biopsy has just been performed, the patient is eligible only if a FFPE tissue sample (or at least 20 slides unstained, freshly cut for the purposes of the study) of the metastatic/recurrent lesion is available. In this specific situation frozen cores are not mandatory.

Note 2: in case of a de novo metastatic disease, if the metastatic lesion biopsy is not feasible, samples of the primary tumour lesion (FFPE + frozen cores) are acceptable.

r. **Only for phase II part: central revision of PD-L1 and CD73 status required prior randomization.** PD-L1 and CD73 status will be assessed using IHC on the screening FFPE tumour lesion biopsy (or on the archived FFPE primary tumour sample in case the tumour lesion biopsy tissue sample is not evaluable for PD-L1 or CD73) by a central laboratory prior to randomization for stratification purposes. Samples should be sent minimum 10 days before planned date of first study drug administration. Patients whose tumour tissue is not evaluable for PD-L1 and CD73 will not be eligible. All site and study team staff will be blinded to PD-L1 and CD73 status.

s. Whole blood sample (1x5mL) is collected at week 1 (before treatment start); Blood samples (3x9mL, 1X 9mL 1x8mL and 1X2.5mL for plasma, serum, PBMCs isolation and mRNA isolation respectively) are collected at week 1 (before treatment start), during week 3 before the second administration of durvalumab (applicable in both arms), for arm A, it must be performed after the second administration of oleclumab), at the first immunotherapy visit (end of chemotherapy = week 13 if no delay) except for PBMCs, at week 25, at progression and at first occurrence of possible immune related toxicity (*see section 11. Translational Research(es)*). If feasible the blood sample for TR research should be performed at the same time as the biopsy.

t. These procedures will be performed as local standard practices

Table 11 Study Procedures at Treatment Discontinuation and after End of treatment (=Follow-up period)

Evaluations	EOT visit [l]<br>28 days after treatment discontinuation	Efficacy Follow-up period [m]<br>Every 12 weeks	Survival Follow-up period [n]<br>Every 6 months
Concomitant medication, therapies & procedures	X		
Physical Examination & Vital Signs & ECOG PS [a]	X	X	
Body Weight	X		
**Laboratory tests**			
Pregnancy test [b]	X	X	
Complete Blood Count (CBC) [c]	X	X[o]	
Chemistry [d]	X	X[o]	
Coagulation [e]	X		
Blood Tumour Marker [f]	X		
Thyroid Function Testing [g]	X		
Lipase	X		
Adverse Events [h]	X		
SAEs/AESIs [h]	X	X	
**Samples collection**			
Tumour assessment [i]	X	X[o]	
Optional Metastatic lesion biopsy [j]	At progression		
Blood samples for TR [k]	At progression		
Data on overall survival			X

a. Vital signs include body temperature, blood pressure, pulse (heart rate).

b. A urine pregnancy test has to be done in all female subjects of childbearing potential (if positive confirm by serum test) at the EOT visit and at 3 and 7 months after EOT visit.

c. CBC includes RBC, Hb, platelet count, WBC (including differential count).

d. Chemistry includes glucose, urea, creatinine, sodium, potassium, magnesium, chloride, bicarbonate, calcium, phosphorus, total serum bilirubin, ALT, AST, ALP, LDH, total protein and albumin.

e. Coagulation includes aPTT and INR.

f. Re-assessment of tumour markers (CA 15-3 and CEA) is only indicated in patients who had elevated markers at baseline (as defined by local laboratory guidelines).

g. Thyroid function: TSH and T4.

h. Continuously from 1st administration of study treatment until 90 days after last administration. From 91 days after last administration of study drugs, only SAEs/AESIs related to study drugs must be reported.

i. Contrast-enhanced CT scan or MRI of the chest, the abdomen and the pelvis must be performed at EOT visit if last tumour assessment was performed more than 28 days before treatment stop date. If last tumour assessment was performed within 28 days of treatment stop date, no need to repeat this exam at EOT visit.

Remark for imaging procedures: The same radiographic procedures used to assess measurable disease sites at screening should be used throughout the study. All known sites of disease must be documented at screening and re-assessed at each subsequent tumour evaluation. Assessments should be performed by the same evaluator, if possible, to ensure internal consistency across visits.

Note 1: Patients who stopped all study treatments for reasons other than PD will continue post-treatment imaging studies for disease status follow-up every 8 weeks (± 7 days) until week 24 and every 12 weeks (± 7 days) thereafter until verified PD, start of a new anti-cancer treatment, withdrawal of consent to study participation, death, or end of the study whichever comes first.

j. Optional metastatic lesion biopsy (*see section 11. Translational Research(es)*).

k. Blood samples (3x9mL, 1X 9mL 1x8mL and 2.5mL for plasma, serum, PBMCs isolation and mRNA isolation respectively) are collected at progression and at first occurrence of possible immune related toxicity (*see section 11. Translational Research(es)*).

l. An end of treatment (EOT) visit should be carried out 28 days (± 7 days) after the study treatment discontinuation or before starting any new anticancer treatment, whatever comes first.

m. The Efficay Follow-Up Period is only applicable for patients who stopped study treatment for reasons other than PD. This period, with assessments planned every 12 weeks (± 7 days), starts after the end of study treatment visit (EOT) and ends at disease progression, start of a new anti-cancer treatment, withdrawal of consent to study participation, death or end of the study whichever comes first.

n. The Survival Follow-up Period starts after the EOT visit for patients who have interrupted study treatment due to disease progression OR at disease progression for patients that are coming from efficacy follow-up period. This period ends at withdrawal of consent to study participation, death, or inability to obtain further information on patient, or end of the study whichever comes first.

During this period there are no further visits but data on overall survival should be obtained every 6 months. Data on overall survival can be obtained during regular out of study clinical follow-up visits, via phone call or patient hospital records.

o. After week 104, these procedures will be performed as local standard practices.

9. ASSESSMENT OF EFFICACY

9.1. Efficacy endpoints

The following efficacy endpoints are assessed:

- Clinical benefit (CB)
- Objective response (OR) assessed as best overall response;
- Duration of response (DOR);
- Progression-free survival (PFS);
- Overall survival (OS);

9.2. Methods for assessing efficacy endpoints

Objective tumour response and PFS will be evaluated through imaging assessment (*see section 3.3.7. Imaging assessment*) and measured according to RECIST v 1.1[64].

As an exploratory endpoint, objective tumour response and PFS will be measured according to immune (i)RECIST[63]. Immunotherapeutics may result in infiltration of immune cells leading to transient increase in the size of malignant lesions, or undetectable lesions becoming detectable. The iRECIST guidelines describe a standard approach to solid tumour measurements and definitions for objective change in tumour size for use in trials evaluating immunotherapeutic agents[66]. iRECIST are identical to those of RECIST 1.1 in many respects but have been adapted to account for instances where an increase in tumour burden, or the appearance of new lesions, does not reflect true tumour progression.

Efficacy assessment will be done locally.

In order to ensure high quality of the local review, imaging data from the screening phase, as well as from week 8, week 16 and week 24 post start of study treatment will be collected at ORILaB and reviewed centrally.

The same method of assessment and the same technique should be used to characterize each identified and reported lesion at baseline and during follow-up.

9.2.1. Documentation of Progression

Progression is documented following RECIST version 1.1 during the whole duration of the trial. During the first 24 weeks, documentation of progression is also performed using iRECIST. Therefore, immune unconfirmed PD as defined by iRECIST (iUPD) can only be assigned for the first 2 imaging assessments (week 8 and week 16) and several times as long as confirmed progression (iCPD) is not confirmed at the next assessment. If PD is not confirmed, reassessment continues as originally planned.

If an iUPD is detected at week 8 or 16 and the next confirmation imaging assessment 8 weeks later has been missed, the next imaging assessment has to be taken into consideration for confirmation of iCPD, provided the clinical condition of the patient allows it. Any scan performed outside the schedule for confirmation is at the investigator’s discretion. In such cases, the schedule of imaging assessment should remain unchanged.

9.2.2. Documentation of iBOR

Any imaging assessment performed after the end of treatment visit are accepted for the assessment of iBOR (immune best objective response), provided the patient has not stopped the treatment due to PD.

10. ASSESSMENT OF SAFETY

10.1. Safety parameters and Methods for assessing safety parameters

The following safety parameters are assessed:

- Demographic data (for details please refer to the paragraph "Schedule of Assessments")
- Medical history (for details please refer to the paragraph "Schedule of Assessments")
- Cancer history (for details please refer to the paragraph "Schedule of Assessments")
- Prior cancer treatment (for details please refer to the paragraph "Schedule of Assessments")
- Concomitant medications (for details please refer to the paragraph "Schedule of Assessments")
- Physical examination and vital signs (for details please refer to the paragraph "Schedule of Assessments")
- ECOG performance status score (for details please refer to the paragraph "Schedule of Assessments")
- Laboratory tests (for details please refer to the paragraph "Schedule of Assessments")
- Test procedures (for details please refer to the paragraph "Schedule of Assessments")
- Adverse Events (AEs) (for details please refer to the paragraph "Pharmacovigilance parameters definitions and reporting guidance")
- Adverse Event of Special Interests (AESIs) (for details please refer to the paragraph "Pharmacovigilance parameters definitions and reporting guidance")
- Serious Adverse Events (SAEs) (for details please refer to the paragraph "Pharmacovigilance parameters definitions and reporting guidance")
- Pregnancy (for details please refer to the paragraph "Pharmacovigilance parameters definitions and reporting guidance")
- Adverse reactions due to overdose, abuse, misuse, medication error (for details please refer to the paragraph "Pharmacovigilance parameters definitions and reporting guidance")

10.2. Pharmacovigilance parameters definitions and reporting guidance

10.2.1. Adverse Events

10.2.1.1. *Definitions*

An ***adverse event (AE)*** is any untoward medical occurrence in a patient or clinical investigation patient administered a pharmaceutical product and which does not necessarily have a causal relationship with this treatment.

An AE can therefore be any unfavourable and unintended sign (including an abnormal laboratory finding, for example), symptom or disease temporally associated with the use of a medicinal product, whether or not considered related to the medicinal product.

An ***adverse reaction (AR)*** is any untoward and unintended response to an investigational medicinal product related to any dose administered.

This definition covers also medication errors and uses outside what is foreseen in the protocol, including misuse and abuse of the product.

This definition implies a reasonable possibility of a causal relationship between the event and the investigational medicinal products (IMPs).

An ***unexpected adverse reaction (UAR)*** is an adverse reaction, the nature or severity (intensity) of which is not consistent with the applicable product information (e.g. investigator's

brochure for an unauthorised investigational product or summary of product characteristics for an authorised product, protocol for radiotherapy/surgery).

Remarks:
- Pre-existing conditions which worsen during a study have to be reported as AEs.
- Abnormal laboratory findings (e.g. haematology, haemostatic function, blood chemistry and serology) or other abnormal assessments (e.g. vital signs) that are judged by the principal investigator as clinically significant have to be recorded as AEs.

10.2.1.2. *AE intensity*

Intensity of all AEs will be graded according to the NCI-CTCAE version 5.0 on a five-point scale (Grade 1 to 5) and reported in details on the CRF. Adverse events not listed in CTCAE should be graded as listed in the table hereunder.

Table 12 Grading for Adverse Events not listed in NCI-CTCAE

CTC Grade	Equivalent To:	Definition
Grade 1	Mild	Asymptomatic or mild symptoms; clinical or diagnostic observations only; intervention not indicated
Grade 2	Moderate	Minimal, local or non-invasive intervention indicated; limiting age-appropriate instrumental activities of daily living (ADL)*
Grade 3	Severe	Severe or medically significant but not immediately life threatening; hospitalization or prolongation of hospitalization indicated; disabling; limiting self-care ADL**
Grade 4	Life-threatening/ disabling	Life-threatening consequences; urgent intervention indicated
Grade 5	Death	Death related to AE

*Instrumental ADL refer to preparing meals, shopping for groceries or clothes, using the telephone, managing money etc.
**Self-care ADL refer to bathing, dressing and undressing, feeding self, using the toilet, taking medications, and not bedridden.

10.2.1.3. *Relationship to study treatments*

The causality relationship of the AE to the study treatment(s) is assessed by the principal investigator as either related or unrelated.

If there is a reasonable suspected causal relationship to the study treatment(s), i.e., there are facts (evidences) or arguments to suggest a causal relationship, event relationship should be assessed as **Related**.

The following criteria should be considered to assess the relationship as **Related (Reasonable possibility):**
- Reasonable temporal association with study treatment.
- It may or may not have been produced by the subject's clinical state, environmental or toxic factors, or other modes of therapy administered to the subject.
- Known response pattern to suspected study treatment.
- Disappears or decreases on cessation or reduction in dose.
- Reappears on re-challenge.

The following criteria should be considered to assess the relationship as **Unrelated (No reasonable possibility)**:
- It does not follow a reasonable temporal sequence from administration of study treatment.
- It may readily have been produced by the subject's clinical state, environmental or toxic factors, or other modes of therapy administered to the subject.

- It does not follow a known pattern of response to the suspected study treatment.
- It does not reappear or worsen when the study treatment is re-administered.

10.2.1.4. *Reporting of adverse events*

From the first administration of study treatment until 90 days after the last administration of the study drugs, all AEs should be reported on the AE page of the CRF.

All AEs will be followed until one of the following occurs:
- Complete resolution
- Return to baseline condition
- Event is considered by the principal investigator to be unlikely to resolve
- Death
- Subject end of study

The final outcome of each AE must be recorded in the CRF.

The principal investigator has to follow-up any AEs that are not resolved after the subject end of study as long as medically indicated but without further recording in the CRF.

Note: Any abnormal laboratory test result and any abnormal assessment (e.g. vital signs) result reported as AE should be followed until it has returned to the normal range, baseline value (laboratory results obtained during the screening) and/or an adequate explanation of the abnormality is found.

10.2.2. Serious Adverse Events

10.2.2.1. *Definitions*

A ***serious adverse event (SAE)*** or ***reaction (SAR)*** is any untoward medical occurrence that at any dose results in any of the following outcomes:
- Death;
- Life-threatening;
- In-patient hospitalisation or prolongation of existing hospitalisation;
- Persistent or significant disability/incapacity;
- Congenital anomaly/birth defect;

Medical and scientific judgement should be exercised in deciding whether expedited reporting is appropriate in other situations, such as important medical events that may not be immediately life-threatening or result in death or hospitalisation but may jeopardise the patient or may require intervention to prevent one of the other outcomes listed in the definition above. These should also usually be considered serious (medically significant condition).

The term “serious” is not synonymous with the term “severe”. The term "severe" is often used to describe the intensity (severity) of a specific event (as in mild, moderate, or severe myocardial infarction); the event itself, however, may be of relatively minor medical significance (such as severe headache). While the term "serious” is based on patient or event outcome or action criteria usually associated with events that pose a threat to a patient's life or functioning. Seriousness (not severity) serves as a guide for defining regulatory reporting obligations.

A ***suspected unexpected serious adverse reaction (SUSAR)*** is a suspected adverse reaction that is both unexpected and serious and is subject to expedited reporting. The expectedness assessment of a serious adverse reaction is done by the sponsor by using the reference safety information within the investigator’s brochure (IB) or the summary of product characteristics (SmPC).

In this study, the expectedness assessment will be performed by using the Reference Safety Information within:

- Investigator's brochure for durvalumab
- Investigator's brochure for oleclumab (MEDI9447)
- Summary of product characteristics for paclitaxel
- Summary of product characteristics for carboplatin

Remarks:

- Death is an outcome and not an event therefore the CTCAE event(s) that caused the death should be reported as SAE(s) except in case of sudden death (An unexpected cessation of life that cannot be attributed to a CTCAE term associated with Grade 5) or death of unknown origin.
- The term "Life-threatening" refers to an event in which the patient was at risk of death at the time of the event; it does not refer to an event which hypothetically might have caused death if it were more severe.
- An in-patient hospitalisation is a hospital stay equal or greater than 24 hours.
- Related second primary malignancies (SPM) [i.e. a cancer judged by the investigator to be related to study treatment(s). It is not considered a metastasis of the initial malignancy] are to be reported as SAEs at any time regardless of the time elapsed since the last dose of study drugs.

10.2.2.2. *Reporting of serious adverse events by investigators*

From Informed Consent Form signature until initiation of the first study treatment(s), all SAEs related to a protocol-mandated intervention (e.g., SAEs related to invasive procedures such as biopsies) have to be reported on SAE form.

All SAEs from the first administration of study treatment(s) and up to 90 days after the last administration of study drugs must be collected and reported on SAE form and on AE page of the CRF.

After this period, only SAEs which have a reasonable possibility to be related to the study treatment(s) (even if the study has been closed) must be reported on SAE form.

All SAEs will be followed until one of the following occurs:

- Complete resolution
- Return to baseline condition
- Event is considered by the principal investigator to be unlikely to resolve
- Death
- End of study

Note: Any abnormal laboratory test result and any abnormal assessment (e.g. vital signs) result reported as SAE should be followed until it has returned to the normal range, baseline value (laboratory results obtained during the screening) and/or an adequate explanation of the abnormality is found.

Table 13 SAE reporting

From ICF signature up to 1st study drugs administration	SAEs related to protocol-mandated intervention
From 1st study drugs administration up to 90 days after last administration of study drugs	All SAEs
From day 91 after last administration study drugs	Only SAEs related to study drugs

All SAEs must be reported to the sponsor immediately and in any case no later than 24 hours from the time the principal investigator or study staff member became aware of

the event. The SAE must be submitted to the sponsor via the study-specific SAE form completed in English.

The SAE report has to be sent to the email address mentioned on the study-specific SAE form.

Any additional documents in local language have to be provided with an English translation of the relevant information.

To be compliant with regulatory reporting requirements, all initial SAE forms must contain the following mandatory information: one identifiable subject, one identifiable reporter, one adverse event, one seriousness criteria, the relationship to study medication, outcome at last observation. Within 7 calendars days, the principal investigator must provide complete information requested on the SAE form of any reported serious adverse event. If the completed form is not received within this deadline, the sponsor will issue queries to the principal investigator.

Any queries on the SAEs sent out by the sponsor need to be answered within 7 days.

All SAE forms need to be reviewed, dated and signed by the principal investigator or medical physician as specified in the study task delegation log.

The principal investigator should report SAEs that occur at their institution to their respective ethics committee according to the local committee's policies.

10.2.2.3. *Reporting of serious adverse events by sponsor*

The SAE reporting and the SUSAR reporting will be in accordance with EU Directive 2001/20/EC and the CT-3 guidance on the collection, verification and presentation of adverse event/reaction reports arising from clinical trials on medicinal products for human use (2011/C 172/01). The sponsor will be responsible of the reporting of the SUSARs to the EudraVigilance Clinical Trial Module (EVCTM), the Competent Authorities, the Ethics Committees and the participating investigators.

10.2.3. Exceptions to AEs/SAEs reporting

Some hospitalisation scenarios do not require reporting as an SAE such as:

- Elective hospitalisation for pre-existing conditions that have not been exacerbated by study medication.
- Hospitalisation planned before the subject consented for study participation and where admission did not take longer than anticipated.
- Hospitalisations for reasons described in the protocol (e.g. hospitalisation for study medication administration, hospitalisation for study related procedures). However, event requiring hospitalisation or prolongation of hospitalisation as a result of a complication of study medication administration or study related procedures is reported as SAE.
- Hospitalisation or prolonged hospitalisation in absence of an AE (social, technical, practical reason and/or convenience admission to a hospital, palliative care, rehabilitation).

Progression of underlying malignancy should not be reported as an AE or SAE if it is clearly consistent with the suspected progression of the underlying cancer but will be reported on the CRF.

Unrelated SPM [i.e. a cancer that is unrelated to the study treatment(s) and is not a metastasis from the initial malignancy] should not be reported as an AE or SAE but is to be reported on the CRF on the appropriate form.

Hospitalisation due solely to the progression of underlying malignancy should NOT be reported as an SAE.

Clinical symptoms of confirmed progression of underlying malignancy should not be reported as AEs or SAEs unless the symptoms cannot be determined as exclusively due to the confirmed progression of the underlying malignancy, or does not fit the expected pattern of confirmed progression for the disease under study.

Deaths related to progression of the underlying disease during the course of the study will not be reported as a SAE, but should be reported on the Death CRF page section (unless the subject has withdrawn consent).

10.2.4. Adverse Events of Special Interest for durvalumab: immune-mediated adverse events

10.2.4.1. *Definition*

The adverse events of special interest (AESIs) for durvalumab are defined as AEs that include, but are not limited to, events with a potential inflammatory or immune-mediated mechanism that may require more frequent monitoring and/or interventions such as corticosteroids, immunosuppressants, and/or endocrine therapy.

Early recognition of signs and symptoms potentially related to an inflammatory or immune-mediated mechanism is important for proper management of toxicities. For guidance on identifying, evaluating, and treating AESI/imAEs, see the Appendix 1 to the protocol.

AESI/imAEs observed include:

- Diarrhoea/colitis.
- Intestinal perforation.
- Pneumonitis events (e.g. pneumonitis, interstitial lung disease (ILD), acute interstitial pneumonitis and pulmonary fibrosis).
- Hepatitis events (e.g. autoimmune hepatitis, hepatitis toxic, hepatocellular injury, hepatotoxicity and hepatitis). Other AESIs such as hepatic failure, jaundice, hyperbilirubinaemia and laboratory abnormality AESIs have been observed.
- Endocrinopathies (i.e. events of hypophysitis, hypopituitarism adrenal insufficiency, diabetes insipidus, hyper- and hypothyroidism,type I diabetes mellitus and diabetes insipidus).
- Rash/dermatitis (including maculo-papular rash, erythematous rash, pruritus, alopecia aerata and vitiligo, eczema and erythema, Steven-Johnsons syndrome, toxic epidermal necrolysis, dermatitis bullous, dermatitis exfoliative or dermatitis psoriasiform, pemphigoid).
- Nephritis events (blood creatinine increases, nephrotic syndrome, rapid progressive glomerulonephritis and acute renal failure).
- Pancreatitis (or laboratory findings suggestive of pancreatitis - increased serum lipase, increased serum amylase)
- Myocarditis
- Myositis/polymyositis
- Events with an inflammatory or immune mediated mechanism could occur in nearly all organs.

Other inflammatory responses that are rare/less frequent with a potential immune-mediated aetiology are also considered as AESIs and include, but are not limited to pericarditis, sarcoidosis, uveitis and other events involving the eye (e.g. keratitis and optic neuritis), skin (e.g. scleroderma and vitiligo), haematological (e.g. haemolytic anaemia and immune thrombocytopenic purpura and immune-mediated neutropenia), rheumatological events (polymyalgia rheumatic and autoimmune arthritis), vasculitis, non-infectious meningitis and non-infectious encephalitis, cholangitis sclerosing, immune-mediated cystitis.

Neuropathy/neuromuscular toxicities such as Guillain-Barré syndrome, and myasthenia gravis are also considered as AESIs.

In addition, infusion-related reactions and hypersensitivity/anaphylactic reactions with a different underlying pharmacological aetiology are also considered AESIs.

More details can be found in the IB.

10.2.4.2. *Reporting AESIs for durvalumab*

Any AESIs for durvalumab ≥ grade 2 (irrespective of the relatedness to durvalumab) occurring during the study treatment period and up to 90 days after the last administration of study treatment must be reported to the sponsor.

After this period, only AESIs ≥ grade 2 which have a reasonable possibility to be related to study drug (even if the study has been closed) must be reported on the SAE form.

All AESIs for durvalumab ≥ grade 2 must be reported within 1 week by the principal investigator or an authorised staff member (i.e. as specified in the study task delegation log) to the sponsor on the study-specific SAE form in English. The report has to be sent to the email address mentioned on the Serious Adverse Event form and on CRF.

All AESIs for durvalumab (< grade 2 and ≥ grade 2) must be reported in the CRF.

Notes:
1. If the above-mentioned AEs ≥ grade 2 occur during the chemotherapy administration and if the events are assessed as definitely related to the chemotherapy by the investigator, they should not be reported on the study specific SAE form.
2. If the above AESIs are also observed for oleclumab, they should be reported to the sponsor as specified above.

10.2.5. Adverse events of special interest for oleclumab: cardiovascular adverse events

10.2.5.1. *Definition*

The adverse events: cardiac chest pain, transient ischemic attack and thromboembolic events are of special interest due to oleclumab potential risk of arterial calcifications, arterial ischemic disorder, and thrombosis.

Edema (e.g, pulmonary or peripheral) is regarded as AESI due to oleclumab potential risks of increased microvascular permeability.

10.2.5.2. *Reporting AESIs for oleclumab*

Any AESIs for oleclumab (irrespective of grade and relatedness to oleclumab) occurring during the study treatment period and up to 90 days after the last administration of study treatment must be reported to the sponsor.

After this period, only AESIs which have a reasonable possibility to be related to study drugs (even if the study has been closed) must be reported on the SAE form.

All AESIs for oleclumab must be reported within 1 week by the principal investigator or an authorised staff member (i.e. as specified in the study task delegation log) to the sponsor on the study-specific SAE form in English. The report has to be sent to the email address mentioned on the Serious Adverse Event form and on CRF.

10.2.6. Potential Hy's law case

10.2.6.1. *Definition*

Hepatic function abnormality that fulfils the biochemical criteria of a potential Hy's Law case in a study subject, with or without associated clinical manifestations, is required to be reported as AESI, unless a definitive underlying diagnosis for the abnormality (e.g., cholelithiasis or bile duct obstruction) that is unrelated to investigational product has been confirmed.

The criteria for a potential Hy's Law case is AST or ALT ≥3x ULN together with TBL ≥2xULN at any point during the study following the start of study medication irrespective of an increase in ALP.

- If the definitive underlying diagnosis for the abnormality has been established and is unrelated to investigational product, the decision to continue dosing of the study subject will be based on the clinical judgment of the investigator.
- If no definitive underlying diagnosis for the abnormality is established, dosing of the study subject must be interrupted immediately. Follow-up investigations and inquiries must be initiated by the investigational site without delay.

10.2.6.2. *Reporting*

Any potential Hy's law case occurring during the study treatment period and up to 90 days after the last administration of study treatment must be reported to the sponsor.

All potential Hy's law case must be reported immediately and no later than 24 hours from the time the principal investigator or an authorised staff member (i.e. as specified in the study task delegation log) became aware of the event to the sponsor on the study-specific SAE form in English. The report has to be sent to the email address mentioned on the Serious Adverse Event form and on CRF.

10.2.7. Pregnancy

A female subject who becomes pregnant during the study must be instructed to stop taking study treatment and to immediately inform the principal investigator.

The principal investigator or an authorised staff member (i.e. as specified in the study task delegation log) should report any pregnancy diagnosed in a female subject during the study treatment period and within 6 months after last study treatment administration. This reporting is done immediately and no later than 24 hours from the time of awareness to the sponsor by using the pregnancy notification form. The pregnancy report has to be sent to the email address mentioned on the Pregnancy form.

The principal investigator should counsel the female subject, and discuss the risks of continuing with the pregnancy and the possible effects on the foetus. The principal investigator should follow-up with the female subject until conclusion of the pregnancy.

If the involved parties sign the specific Informed Consent Form for data collection in case of pregnancy, the principal investigator should report the outcome of any pregnancy and report any abnormality in the newborn within 1 month after the delivery as a follow-up to the initial pregnancy notification to the sponsor. While the pregnancy itself is not considered to be an AE or SAE, any pregnancy complication, any spontaneous abortion, any abnormality in newborn and any child death (intrauterine death, stillbirth, post-delivery death) will be recorded as an AE or SAE (if any of the criteria for seriousness is met).

10.2.8. Overdose, misuse and abuse

10.2.8.1. *Definitions*

Overdose: this refers to the administration of a quantity of a medicinal product given per administration or cumulatively which is above the maximum recommended dose according to the protocol. Clinical judgement should always be applied.

Misuse: this refers to situations where the medicinal product is intentionally and inappropriately used not in accordance with the protocol.

Abuse: This corresponds to the persistent or sporadic, intentional excessive use of a medicinal product, which is accompanied by harmful physical or psychological effects.

10.2.8.2. *Reporting of overdose, misuse and abuse*

The principal investigator or an authorised staff member (i.e. as specified in the study task delegation log) should report any adverse reaction due to overdose, misuse and abuse occurring in a subject during the study medication administration period immediately and no later than 24 hours from the time of awareness to the sponsor by using the appropriate notification form. The report has to be sent to the email address mentioned on the notification form.

10.2.9. Medication Error

10.2.9.1. *Definition*

Medication error: this refers to any unintentional error in the prescribing, dispensing, or administration of a medicinal product while in the control of the healthcare professional or subject.

10.2.9.2. *Reporting of medication error*

The principal investigator or an authorised staff member (i.e. as specified in the study task delegation log) should report any adverse reaction due to medication error, occurring in a subject during the study medication administration period immediately and no later than 24 hours from the time of awareness to the sponsor by using the appropriate notification form. The report has to be sent to the email address mentioned on the notification form.

11. TRANSLATIONAL RESEARCH(ES)

11.1. Overview

The translational research proposal within this clinical trial aims to understand the host/tumour immune interaction before and after treatment to identify potential predictive biomarker of response to treatment as well as mechanisms underlying resistance to immunotherapy. This project aims also to improve our understanding of mechanisms of action such as apoptosis induction, reduction of proliferation, immune pathways activation and oncogenic pathways inhibition underlying the synergistic effects of the combination of paclitaxel, carboplatin and durvalumab with oleclumab.

For this purpose, several tissue samples (archived primary tumour, biopsies of metastatic/recurrent lesions) and blood samples collection will be performed during the study.

Stratification factors (only for phase II): PD-L1 and CD73 expression will be assessed using immunohistochemistry (IHC) on the screening FFPE metastatic/recurrent tumour tissue lesion

sample (or on the archived FFPE primary tumour sample in case the metastatic/recurrent tumour lesion sample is not evaluable for PD-L1 or CD73) by a central laboratory prior to randomization for stratification purposes. For the screening, metastatic and archived primary tissue samples must be sent to the central laboratory. In case of *de novo* metastatic disease, a sample of the primary tumour lesion is acceptable for stratification.

Patients whose tumour tissue is not evaluable for PD-L1 and CD73 will not be eligible.

All sites and study team staffs will be blinded to PD-L1 and CD73 status.

11.1.1. Breast primary tumour tissue sample - mandatory

To be collected at screening:

- Patient experiencing a relapse: archived FFPE tissue from the surgical specimen or the diagnostic/pre-therapeutic biopsy in case the patient has been treated with neo-adjuvant chemotherapy.
  Patient experiencing their first diagnosis (De novo diagnosis): FFPE biopsy of the breast primary tumour.
- A FFPE tissue block is preferred but tissues slides are accepted (at least 20 tissue (5 µm) Superfrost Plus (+) slides, freshly cut for the purpose of the study).

11.1.2. Metastatic/recurrent lesion tissue samples

For all biopsies performed for the translational research part of the SYNERGY trial, one FFPE block and one frozen tissue core will be collected as a priority. In specific cases, when it is feasible based on the size and the accessibility of the metastatic lesion, two additional fresh tissue cores should also be collected (in selected participating sites). All tissue cores (FFPE, frozen and fresh) must be collected from the same lesion at the same time. For fresh tissue cores only, some specific sites will participate in this collection. Not all sites are mandated to collect fresh tissue cores.

Acceptable tissue samples include those from resections, core-needle biopsies or excisional, incisional, punch, or forceps biopsies. Fine-needle aspiration (defined as samples that do not preserve tissue architecture and yield cell suspension and/or smears), brushing, and cell pellets from cytology samples are not acceptable.

11.1.2.1. *Biopsy at screening - mandatory*

A biopsy of a metastatic/recurrent tumour lesion must be performed prior to inclusion, during the screening period.

Note 1: if a metastatic lesion biopsy has just been performed, the patient is eligible only if one archived FFPE tissue sample (or at least 20 unstained slides, freshly cut for the purposes of the study) of the metastatic/recurrent lesion is available. In this specific situation only, frozen/fresh tissue cores are not mandatory.
Note 2: in case of a de novo metastatic disease, a biopsy of the primary lesion is acceptable (at least 1 FFPE tumour tissue and 1 frozen core as a priority, if feasible 2 additional fresh tumour tissue cores should be collected too).

11.1.2.2. *Biopsy "on treatment" - mandatory*

The second "on treatment" biopsy must be performed during week 3 before the second administration of durvalumab (applicable in both arms). Important note, for arm A it must be performed after the second administration of oleclumab. If feasible the preferred option is to

biopsy the same tumoral (metastatic/recurrent or primary in case of *de novo* disease) lesion biopsied at screening.

11.1.2.3. *Biopsy "at disease progression" - optional*

A biopsy "at progression" of progressive lesions (if feasible the preferred option is to biopsy the same metastatic lesion biopsied at screening), if deemed clinically feasible by the investigator, at the time of first evidence of clinical or radiographic disease progression can be performed to evaluate mechanisms of treatment resistance.

<u>Note 1</u>: biopsies "at progression" should be performed within 40 days after PD has been reported and prior to the next anti-cancer therapy.

11.1.3. Blood samples collection

Several blood samples collection (whole blood, PBMCs, PaxGene RNA, plasma and serum) will be performed, for the purpose of the translational research part, at several time points:

- Baseline blood collection at week 1 before treatment start (meaning within 3 days before study drug administration);
- "On treatment" blood samples collection: during week 3 before the second administration of durvalumab (applicable in both arms), for arm A it must be performed after the second administration of oleclumab; at the first immunotherapy visit (end of chemotherapy = at week 13 in case no chemotherapy interruptions did occur) except for PBMCs; at week 25;
- "At first disease progression" (based on RECIST v1.1) blood samples collection within 40 days after PD has been reported and prior to the next anti-cancer therapy;
- At first occurrence of possible immune related toxicity (development of an iAE determined by the investigator to be unacceptable given the individual patient's potential response to therapy and severity of the event).

If feasible, blood samples for TR research should be collected at the same time as the biopsy.

The blood samples must be collected as follow:

- Whole blood for isolation of normal DNA to exclude germline variants during our next-generation sequencing analyses (1x5mL, at baseline only);
- Blood for plasma isolation (3x9 mL per time point);
- Blood for serum isolation (1x9mL per time point);
- Blood for PBMCs isolation (1x8 mL per time point, except at end of chemotherapy);
- Blood for mRNA isolation (1X2.5mL per time point).

11.2. <u>Objectives</u>

The objectives of the translational research project are:

1) To identify potential predictive immune biomarkers by performing a widespread histological and molecular profiling of the pre- and post- study treatment peripheral blood and tumour tissues using IHC and other histological methods as immunofluorescence as well as flow cytometry and chromatography;
2) To identify genomic alterations associated with treatment efficacy by characterizing pre- and post- study treatment tissue samples using whole-genome and RNA-sequencing;
3) To assess the role of the tumour microenvironment in the treatment efficacy by applying single-cell level spatial transcriptomics;
4) To provide a readout of the T-cell receptor repertoire present in tumour-infiltrating T-cells using single-cell RNA sequencing;
5) To evaluate the role of plasma ctDNA in monitoring response/resistance to the study treatment.

6) To evaluate blood-based gene signatures predictive of response and evaluate on-treatment effects.

Due to the fast evolution within these fields, the actual analytical methodology will only be specified and decided at the time these analyses will start.

11.3. Timelines

Table 14 Samples collection timelines

	**Screening**	**Week 1[c]**	**During Week 3[d]**	**first immunotherapy visit**	**Week 25**	**First Immune related toxicities**	**First PD**
Breast primary tumour tissue (FFPE block or 20 unstained slides freshly cut for the purposes of the study)	X[a]						
Metastatic/recurrent lesion biopsy (1 FFPE + 1 frozen tissues and 2 fresh (if feasible and in some selected sites) tissue cores)	X[b]		X[e]				X (optional)
Whole Blood (1X5mL)		X					
Blood for plasma (3X9mL)		X	X	X	X	X	X
Blood for serum (1X9mL)		X	X	X	X	X	X
Blood for PBMCs (1x8mL)		X	X		X	X	X
Blood for mRNA isolation (1X2.5mL)		X	X	X	X	X	X

a) Patient experiencing a relapse: archived FFPE tissue from the surgical specimen or the diagnostic/pre-therapeutic biopsy in case the patient has been treated with neo-adjuvant chemotherapy. / Patient experiencing their first diagnosis (De novo diagnosis): FFPE biopsy of the breast primary tumour.
b) 1. If the subject has just performed a metastatic lesion biopsy, the subject is eligible only if an archived FFPE tissue sample (or at least 20 slides unstained, freshly cut for the purposes of the study) of the metastatic/recurrent lesion is available. In this situation frozen/fresh tissue cores are not mandatory.
2. In case of a de novo metastatic disease, a sample of the primary tumour lesion is acceptable (at least 1 FFPE tumour tissue and 1 frozen core as a priority, if feasible 2 additional fresh tumour tissue cores should be collected too in specific sites).
c) Before treatment start (meaning within 3 days prior study drug administration)
d) Before the second administration of durvalumab (applicable in both arms), for arm A it must be performed after the second administration of oleclumab.
e) If feasible the preferred option is to biopsy the same lesion as biopsied at screening.

Important notes:

1/ besides these timelines, if the patient is operated to the primary breast tumour (in case of a *de novo* metastatic disease) and/or to a metastasi(e)s after week 24 of the study, provision of 1 FFPE and 1 frozen core of the surgical specimen is optional, but encouraged, in order to better understand mechanisms of response to treatment.

2/ in case the patient also participates to a molecular screening program like the AURORA program, the site should ensure that the required tumour biopsies can be provided separately in the SYNERGY study and in this program.

11.4. Biological material handling

Standardised protocols are applied consistently in preparing and storing biological samples to ensure quality and to avoid introducing confounders into research studies. See also the National Cancer Institute (NCI) best practice for biospecimens resource (http://biospecimens.cancer.gov/bestpractices/), the Recommendations for Collection and Handling of Specimens from Breast International Group (BIG) (reference) and the BIG movie on improving translational research samples collection in international trials (available on http://www.youtube.com/user/BreastIntGroup).

Kits are provided to all participating sites in order to perform biological samples collection. The kits are provided by a central service provider.

Further details on collection, handling, local storage and shipping are provided in a separate laboratory instruction manual.

11.5. Central biobanking of biological material collected during the trial and Destruction

Biological samples, material left over and/or derivatives (after molecular analyses embedded in the current trial have been performed) are stored securely, for a minimum of 20 years or until exhaustion or for the maximum time allowed by local regulations or until a request is made for the biological samples to be returned or destroyed, under the responsibility of TR Manager located at Institut Jules Bordet. Some of this biological material may be shipped for further analyses if necessary to other academic institutions or for-profit organisations.

To protect the subjects' identity and privacy, the biological samples (tissue and blood) are double-coded. First they are labelled with a unique study number or 'code' and this code does not carry any personal identifiers. Then the biological samples are relabelled with a second code, which is linked to the first code via a second coding key. The use of the second code provides additional confidentiality and privacy protection over the use of a single code. Access to both coding keys is needed to link any data or biological samples back to a subject identifier. In general, the principal investigator is responsible for maintaining the first coding key and does not have access to the second coding key. As the biological samples and associated data can be traced back indirectly via the use of both coding keys, it may be possible to undertake actions such as sample withdrawal. The principal investigator, his/her staff or those working with them (for example, other researchers) only use the biological samples for the purpose stated in the current trial in accordance with the terms outlined in the Informed Consent Form. The principal investigator and his/her staff require anyone who works with the biological samples to agree to hold the research information and any individual results in confidence.

Subjects may request that their biological samples as well as their material left over and/or derivatives be removed from storage and destroyed or returned to their institution in accordance with the terms outlined in the Informed Consent Form. Requests for destruction or return of the biological samples should be made in writing to the principal investigator. In turn, the principal investigator must promptly inform the sponsor so that appropriate follow-up can be initiated. The principal investigator will be informed when the biological material is destroyed or returned and should notify the subject that biological material destruction is complete or that biological material is stored back in the institution.

Please note any molecular data that is already generated at time of biological material destruction or return request will be used for translational research objectives listed in the protocol. Please also note that specific molecular and clinical data (generated within the study or future research (see below)) could be put on secured database accessible via internet. This can be done for the purpose of data sharing or for publication needs. Data will be double

coded and no reference to subject name will be made. Accession to this data will be made available to any individuals making a specific request and following data access policy rules (specific per database) previously agreed by the sponsor.

Further details on the legal entity acting as data controller, the legal entity acting as data processor and the medical doctor responsible for the sponsor biobank will be provided in a separate laboratory instruction manual.

11.6. Future undefined research

Data and biological samples as well as material left over and/or derivatives (after molecular analyses embedded in the current trial have been performed), may be used for undefined future research. Proposals for access to such samples and data will have to be approved by the study team as well as the appropriate Ethics Committee(s). Access to any biological samples as well as material left over and/or derivatives thereof for future research is only possible if subject consented to the use of the biological material for future undefined research. Electronic documents to keep track of tumour and blood samples will be kept in participating sites and at study sponsor. These documents will contain the subject ID code with no reference to subject name, initials and address. Some of this biological material may be shipped for further analyses if necessary to other academic institutions or for-profit organizations.

12. STATISTICAL CONSIDERATIONS

12.1. Statistical design

12.1.1. Sample size

PHASE I:

- Number of evaluable patients required: minimum 6 patients - maximum 18 patients.
- Estimated number of patients to enrol: minimum 7 patients - maximum 21 patients.
- Estimated number of patients to screen: minimum 8 patients – maximum 24 patients

PHASE II:

The primary hypothesis is that the combination of chemotherapy + durvalumab + oleclumab increases the clinical benefit rate (CBR) by 20% compared to chemotherapy + durvalumab (i.e. from an expected CBR in the control arm 40 % to 60% in the experimental arm). The expected CBR in the control arm is based on early results of an ongoing phase Ib/II trial [64]. For definition of CB and response assessment, *please see section 2.2. Endpoints.*

With a one-sided Type I error (α) 0.1 randomizing 68 patients to each treatment arm will provide 80% power to detect the aforementioned 20% treatment difference in CBR by RECIST (applying Casagrande continuity correction). One interim analysis for futility is planned at an information fraction of 0.50 (68 evaluable patients randomized = 34 patients per arm).

- Number of evaluable patients required: 136 patients (68 patients per arm).
- Estimated number of patients to randomize: 150 patients
- Estimated number of patients to screen: 180 patients

A patient is deemed evaluable if she received at least 1 treatment dose (at least one dose of durvalumab and/or oleclumab).

12.1.2. Study Duration

12.1.2.1. *Expected Accrual Time*

PHASE I: It is estimated that 2 patients per month will be enrolled leading to an accrual time between 3 and 9 months.

PHASE II: It is estimated that 10 patients per month will enter the treatment phase leading to an accrual time of 15 months.

In total, the accrual time will be between 18 and 24 months.

12.1.2.2. *Expected Duration of the Trial*

Approximate treatment duration for a patient: 12 months

Expected duration of the all study: approximately 50 months (taken into account accrual, treatment period and follow-up), assuming that the phase II starts approximately 1 month after the safety interim analysis of the phase I.

As defined in the End of Study definition (*section 15. End of study*), the study will end approximately 24 months after the date the last patient has been randomized.

12.1.3. Randomization and Stratifications for Phase II

Patients will be randomized 1:1 between chemotherapy + durvalumab + oleclumab and chemotherapy + durvalumab.

The stratification factors are CD73 expression, PD-L1 status and the trial site.

Note: Unless not evaluable, stratification is based on CD73 expression/PD-L1 status of a metastatic lesion. If not evaluable, CD73 expression and PD-L1 status of the primary breast lesion will be analysed and patients stratified accordingly. Patients whose tumour tissue is not evaluable for PD-L1 and CD73 will not be eligible. All site and study team staff will be blinded to PD-L1 and CD73 status.

12.2. Statistical plan

The analysis populations are defined as follows:

PHASE I: Patients of the phase I belong to the safety population if any amount of study treatment has been administered.

PHASE II:

- The primary analysis population will be a modified intention to treat (mITT) population, which is defined as all randomized patients, who have received at least one dose of treatment (whether or not corresponding to the assigned study treatment).
- The safety population is defined as patients who received any amount of the assigned study treatment.
- Per protocol, population is defined as all randomized patients who are eligible and have started their allocated treatment. A patient will be considered to be eligible if he/she did not have any major deviations from the patient entry criteria. Eligibility will be assessed by the medical review team.
- CBR, ORR, DOR, PFS and OS will be estimated in the mITT population. The rate of patients not evaluable for the primary analysis population will be closely followed. NOTE: CBR, ORR, DOR, PFS and OS will be reported separately for patients of the phase I part.

- For safety analyses, only patients in the safety population will be analysed.

12.3. Interim Analysis

The study has at least two planned safety interim analysis for the phase I part and one planned efficacy interim analysis for futility for the phase II part.

12.3.1. Phase I: Safety Interim Analysis

A safety interim analysis was performed 28 days after the first administration of oleclumab in the 3rd and the 6th patient of the DL0 cohort. Interim safety data was reviewed by the scientific committee. In addition, continuous safety monitoring was performed for the phase I part prior to the safety interim analyses.

SYNERGY began enrolment on 02/01/2019. As of 04/04/2019, when the last enrolled patient finished the DLT period, 0 out of 6 patients treated experienced a DLT, thus the RPD2 for oleclumab is DL0 – 3000 mg. Outside the DLT period, within a data-freeze of 11/04/2019, however, the Scientific Committee perceived a high frequency (83.3% - 5 out of 6 patients) of high-grade neutropenia, with 7 events, and one serious AE, corresponding to a grade 3 febrile neutropenia associated with a grade 3 lung infection (16.7% of treated patients) related to the chemotherapy regimen.

In order to improve the safety of study treatment and also to preserve its dose-density (and consequently its efficacy), the Scientific Committee decided to reduce the carboplatin dose from AUC 2 to AUC 1.5 for the Phase II part of the study, aiming at avoiding delay and dose reductions for both chemotherapy drugs (carboplatin + paclitaxel).

No other safety signals were perceived by SYNERGY`s Scientific Committee so far, therefore no other study treatment modifications are recommended for the purpose of this protocol amendment.

Long term safety results will be reviewed by an independent data monitoring committee (IDMC) after one year following the beginning of the phase I part. Of note, safety data of ongoing phase II patients will be included in this first IDMC report.

12.3.2. Phase II: Efficacy and Safety Interim Analysis

An interim analysis for futility was planned after the enrolment of 68 evaluable patients (34 per treatment arm). Safety and efficacy results were reviewed by the IDMC. The interim analysis was carried out when CBR assessment was possible for all patients, i.e. 24 weeks after the first treatment dose of the last of the 68 evaluable patients.

The primary purpose of efficacy interim analysis was to check whether the alternative hypothesis could be rejected prematurely. For planning the interim analysis, a Lan-Demets approach has been used (O'Brien and Fleming spending functions for β error). On the z scale, the stopping boundary is 0.074 (i.e., at interim, in case the z test statistic is below 0.074, the trial will stop early for futility).

The IDMC meeting took place on 28 May 2021. In the interim analysis the pre-specified boundary for futility was crossed (z-value = -0.2574 (<0.074); p-value=0.6914 (>0.464; one-sided Fisher's exact test) suggesting the futility of oleclumab addition to the treatment. No safety concerns were identified for the arm with oleclumab. The IDMC recommended stopping the further recruitment for patients in the trial in both arms. Following this recommendation, recruitment stopped on 22 June 2021. Furthermore, the IDMC stimulated the sponsor to consider offering patients still on therapy to continue treatment either without oleclumab or with oleclumab after extensive informed consent. The most important endpoint in clinical trials for advanced breast cancer patients is overall survival. Long-term benefit with immune

therapeutics have been observed. After internal discussion and discussion with the principal investigators the sponsor agrees to offer the possibility to continue treatment with oleclumab (arm A) or without (patients in arm B and patients wishing to cross from A into arm B, respectively).

12.4. Statistical Methods

12.4.1. Efficacy Parameters

12.4.1.1. *CBR and DCR*

The primary treatment effect CBR will be summarized by treatment arms with the 95% CI for the difference between the two proportions.

The comparison for the binary endpoints (the primary endpoint ORR, but also DCR) will be performed on the modified intention-to-treat population using a one-sided Fisher exact test.

12.4.1.2. *DOR, PFS and OS*

The time to event parameters DOR, PFS and OS will be described using Kaplan-Meier curves. Median estimates as well as estimates at 24 weeks and 1 year will be provided as well as an estimation of the hazard ratio between arms together with its 95% CI. Comparison between the treatment arms will be done using a logrank test using a 0.1 significance level (1-sided).

12.4.1.3. *Data Recoding and Display*

Frequency tables will be tabulated (by treatment group or otherwise specified) for all categorical variables by the levels of the variables as they appear on the CRF (with %). Categories with a text field specification will be tabulated as categories and then supplemented by a listing with the following information for the patients fulfilling the condition for the specification (patient id, institution, treatment group, value of the item and text field contents).

Dates relating to events prior to entry will be presented as the delay in days (or weeks, months, or years) between the past event and the date of entry (date of randomization – date of past event + 1) and presented using the median and range. For example, the date of last administration of prior treatment (or the date of first diagnosis of the cancer) will be presented as the time elapsed (in days, weeks, months or years, as appropriate) since the day of the last administration and the date of entry on study (date of randomization – last administration/diagnosis +1).

Other delays (e.g., re-treatment delays) are presented as continuous variables using the median and range.

Continuous variables for which a coding system exists (such as for laboratory data) will be recoded into categories (for adverse events, the grading scale specified in the protocol will be used). Whenever no specific scale exists, lab data will be categorized based on the normal range: for example, below the lower normal limit (when appropriate), within the normal range, above the upper normal limit (ULN) and the degree to which it is above the ULN (for example > 2.5 x ULN, > 5 x ULN, > 10 x ULN). For laboratory data, the nadir is generally displayed. The nadir in a given cycle is the lowest laboratory value in that cycle; the overall nadir for a patient is the lowest laboratory value among all cycles.

Other continuous variables (for example age, dose …) will be presented using the median and range (minimum, maximum).

If appropriate, continuous data may also be presented in categories (e.g. age may also be grouped in decades).

13. FIRST ACT OF RECRUITMENT

The start of the study is the first act of recruitment. This is the date of signature of the informed consent form by the first patient in the study.

14. SUSPENSION OR TRIAL TERMINATION

The sponsor must suspend or terminate the trial prematurely in certain circumstances, including:

- if the positive opinion by the Ethics Committee in charge of the trial is not granted or irrevocably revoked;
- if the authorisation by the Competent Authority of the Member State concerned in charge of the trial is not granted or irrevocably revoked;
- if patient recruitment is insufficient;
- the safety or health of participants is at risk, particularly as a result of inadequate product safety or manufacturing defects;
- alterations in accepted clinical practice that make the continuation of the trial unwise or harmful to participants;
- the Sponsor and/or the Institution and/or the Principal Investigator become or are declared insolvent or a petition in bankruptcy has been filed against it or if one of them is dissolved.

All the investigators will be immediately informed of the suspension or termination of the trial using expedited means (e.g. e-mail, investigator letters, Ethics Committee information).

The patients should be contacted by the investigators to tell them the suspension or the termination of the trial and to inform them of the actions they need to take. A follow-up appointment should be made for patients so that their condition can be reviewed and any concerns addressed.

15. END OF STUDY

The end of study is declared when all the following criteria have been met:

- Approximately 24 months after the date the last patient has been randomized.
- Until every patient reached PD or died or withdrew consent or is lost to follow-up
- The database has been fully cleaned and frozen for all analyses

16. DIRECT ACCESS TO SOURCE DATA/DOCUMENTS

The participating site and the principal investigator shall allow the monitors, the employees of the sponsor (provided that subject confidentiality is protected) or persons subcontracted by the sponsor responsible for auditing, the representatives of the Ethics Committees, and of the Competent Authorities/ Regulatory agencies to have direct access to source data / documents/ complete study records.

17. QUALITY CONTROL AND QUALITY ASSURANCE

17.1. Quality control

Throughout the study the dedicated sponsor study team members verify the data to ensure that:

- the rights and well-being of subjects are protected
- the reported trial data are accurate, complete and verifiable from source documents

- the conduct of the trial is compliant with the ICH-GCP, the applicable regulatory requirements, the study protocol and the study guidelines.

Quality control activities combine central monitoring and clinical site monitoring. Monitoring activities may be performed remotely and/or on site according to the study needs.

17.2. Quality assurance

To ensure compliance with the protocol, CRF completion guidelines, study documentation, SOPs, GCP and all applicable regulatory requirements, the sponsor may conduct a quality assurance audit on participating site. Regulatory agencies may also conduct a regulatory inspection of the study. The principal investigator must inform the sponsor in case an inspection from Regulatory agencies has been scheduled at his/her site.

Such audits/inspections can occur at any time during or after completion of the study. If an audit or inspection occurs, the investigator and institution must agree to allow the auditor/inspector direct access to all relevant documents and to allocate his/her time and the time of his/her staff to the auditor/inspector to discuss findings and any relevant issues in the presence of the sponsor, as needed or required.

18. REGULATORY AND ETHICAL CONSIDERATIONS

18.1. Obligations

18.1.1. Trial Master File

The sponsor shall set-up and maintain a e-Trial Master File (e-TMF) containing the essential documents and written communications relating to that Study which allow verification of the conduct of a Study and the quality of the data generated. The e-TMF must be readily available, and directly accessible upon request, to the Member States Concerned. The e-TMF must be kept in a secure location for the duration of the Study and archived after completion or premature termination of the Study in a secure fireproof facility for at least 25 years.

The sponsor shall provide all the participating sites with a copy of the final protocol, subject information sheets, consent forms and all other relevant study documentation; and any approved modification thereof when applicable.

18.1.2. Regulations, laws and guidelines

The sponsor (or its legal representative) and the principal investigators shall ensure compliance with the Regulation EU No 536/2014, the Guideline for Good Clinical Practice" ICH-E6 Tripartite Guideline, the principles of the "Declaration of Helsinki" 1964, as revised from time to time, and the EU General Data Protection Regulation (GDPR).

18.1.3. Authorisation

The sponsor (or its legal representative) shall submit an application dossier to the intended Member States Concerned in accordance with the Regulation EU No 536/2014, taking into account any local regulations/requirements. Each Member State concerned will consider the Reporting Member State's conclusion on Part I, and the ethics committee's opinion on Part II, and issue their single national decision via the Clinical Trials Information System (CTIS). A Study cannot be commenced in a Member States Concerned until the single national decision has been issued. In case of a staggered application, starting with only a part I application, the part II application may be submitted after the conclusion part I, but not later than two years after the part I conclusion.

18.1.4. Modifications

The sponsor shall be entitled to modify the Study. It is up to the sponsor to decide whether a modification is to be regarded as substantial or not, based on the definitions given in the Regulation. Any substantial modification of the Study, including the addition of a clinical trial site or the change of a principal investigator in the clinical trial site, can only be implemented in a Member State Concerned if it has been approved in accordance with the procedure set out in this Regulation.

Information on any changes to the clinical trials which are not substantial modifications but are relevant for the supervision of the clinical trials by the Member States Concerned shall be permanently updated in CTIS by the sponsor. A non-substantial modification is a modification without substantial impact on the safety or rights of the subjects and/or the reliability and robustness of the data, and the information is not necessary for oversight. This modification should not be reported as such.

18.1.5. Notifications

The sponsor (or its legal representative) shall notify through the CTIS the start of the Study, the end of recruitment and eventually the restart of recruitment, temporary halt and eventually restart, early termination, all unexpected events, urgent safety measures, end of the study, serious breaches.

18.1.6. Summary of results

When intermediate data analysis is provided for in the protocol (related to an adaptive or integrated design), a summary of these results has to be submitted in CTIS within one year after the completion of the intermediate data analysis. It is restricted to the endpoints of the intermediate analysis as defined in the Study protocol. The summary might be expanded if justified, e.g. dose selection in integrated protocols based on safety or/and pharmacological data.
Irrespective of the outcome of the Study, within one year from the end of the Study in all Member States concerned, the sponsor shall submit to CTIS both a summary of the results of the clinical trial and a summary for laypersons.

18.2. Informed consent form and procedure for obtaining patient's consent

18.2.1. The informed consent

The informed consent form (ICF) is based on the Belgian national template which is available on the web site of competent authority (AFMPS-FAGG). It is identified by a version number and a date, and it makes reference to the associated protocol (title of the clinical trial, EudraCT number, protocol number).

The ICF describes in details the following items: the investigator and the sponsor, objectives and description of the study protocol (procedures, methods, IMP/interventions), course of the study, drugs received as treatment, risks and discomforts (possible adverse events and potential hazards of the study including risks related to a pregnancy), benefits, costs, notification of new information, interest to participate in the study, alternative treatment,

withdrawal of the study, treatment after stopping or at the end of the study, publication of study results, contact details at the hospital, translational research, data protection clause insurance and liability, signatures.

The ICF is translated into the national language(s) of the participating country(ies) under the responsibility of the sponsor or its authorized delegate. Then the translations must be approved by an appropriate independent ethics committee/independent review board (IEC/IRB). Once approved, the ICF and the IRB/IEC's written approval/favourable opinion of the ICF are provided by the sponsor to the principal investigators in the national language(s) of their countries.

The principal investigators are not allowed to make any changes in the approved version of the ICF unless these changes are non-substantial for the sponsor, such as but not limited to adding the hospital logo or crest, completing a contact person’s phone number.

The ICF to be provided to patients shall be revised whenever important new information becomes available that may be relevant to the patient’s consent. Any revised ICF shall receive the IRB/IEC's approval/favourable opinion in advance of use.

18.2.2. The procedure for obtaining patient’s consent

SYNERGY is an international, multicenter clinical trial. The terms and conditions described below are subject to certain variations depending on the country and its national laws, but also on the hospitals where the participants will be recruited.

There will be no direct advertising for research subjects, i.e., advertising that is intended to be seen or heard by prospective subjects to solicit their participation in a study. Direct advertising includes, but is not necessarily limited to: newspaper, radio, TV, bulletin boards, posters, and flyers.

However, for the sake of transparency, a description of this clinical trial will be available at http://www.ClinicalTrials.gov (USA). The information will be also available in a similar European website, https://euclinicaltrials.eu/. Such an information may be available on official national websites such as but not limited to the competent authorities websites (for example BELGIUM : https://banquededonneesessaiscliniques.be/fr), the website of the National Cancer Institutes (for example, FRANCE : http://www.e-cancer.fr), and some participating sites may also publish some information about the trial on their own websites.

The informed consent process can only start once the study has been given a written positive approval/favourable opinion by the IRB/IEC's and has been approved by the National Competent Authorities if required by national laws of the participating country(-ies). Also the Investigator’s site initiation process must have been completed by the Sponsor or its authorized subcontractor(s) (including a signed contract between the site and the Sponsor and/or CRO). Patients should be identified as potentially eligible by a healthcare professional that has clinical responsibility for the patient. This is to ensure medical confidentiality is adhered to. Patients will be also assessed for their potential eligibility by a healthcare professional that is authorised to recruit to the study.

Once a patient has been assessed and identified as potentially eligible for the study, the patient should be approached and informed to the study.

The person informing the patient on the study must be familiar with all aspects of the study as described in the latest IEC/IRB approved version of the protocol.

The investigator, or another member of the investigating team, should discuss the study with the patient whereby the patient is given the opportunity to understand the objectives, risks and inconveniences of the trial and the conditions under which it is to be conducted. The information should be provided to patients in both oral and written forms. The language used

to inform the patient, both oral and written, should be concise, described in layman's terms and should be understandable to the patient and impartial witness/interpreter, when applicable. The person obtaining the informed consent must have the patient's medical notes and the current IEC/IRB approved versions of the ICF available during the discussion. Other study documents may also be used during the interview (e.g. patient diaries, study schedule sheets).

All patients receive the appropriate version of the written information and are asked to read and review it.

Usually, if the patient is considering participating in a clinical trial, she may take the consent document home to discuss with family, friend or advocate.

All prospective patients must have the cognitive ability to provide legally effective informed consent. For patients not qualified to give or incapable of giving consent, written consent must be obtained from the legal representative. The legal representative is an individual authorized under applicable law to consent on behalf of a prospective patient, to the patient's participation in the clinical study. This person should be designated by judicial decision or by a mandate (signed and dated by the two parties). In default, the authorized person who may consent on behalf of a patient is, in decreasing order: (i) the spouse, legal cohabitant and (ii) child (adult), parent, brother or sister (adult). In the case where both the patient and her legal representative are unable to read, an impartial witness should be present during the entire informed consent discussion. An impartial witness is a person, who is independent of the study, who cannot be unfairly influenced by people involved in the study, who attends the informed consent process if the patient cannot read the ICF. After the patient and/or legal representative has/have orally consented to participation in the trial, the witness's signature on the form will attest that the information in the consent form was accurately explained and understood.

Neither the investigator nor any member of the study team shall coerce or unduly influence a patient to participate or to continue to participate in a study. The principal investigator or designee also explains to the patient that the participation to the study is voluntary and that the patient is free to refuse to enter the study or to withdraw from it at any time, for any reason without any impact on the patient's subsequent care. No claims shall be made, either explicitly or implicitly, that the drug, biologic or device is safe or effective for the purposes under investigation, or that the drug, biologic or device is known to be equivalent or superior to any other drug, biologic or device. It is the responsibility of the principal investigator or a person designated by the principal investigator (if acceptable by local regulations) to obtain a signed written informed consent from each potential patient prior any study related procedure being carried out. The informed consent is applicable throughout the patient's participation to the study. It is commonly accepted that patient should have a minimum of 24 hours between the date the ICF is provided to the patient and the actual date when the patient (and/or the patient's legally designated representative) signs the informed consent. The date and time the trial was discussed and the date the ICF was given to the patient must be documented in the medical notes.

The consent form must be signed prior to any study related pre-screening or screening procedures being performed. The person obtaining the patient's consent should also inform relevant team members of the treatment decision for the patient.

The written ICF must be dated and personally signed by the principal investigator or authorised sub-investigator and the patient giving consent (or patient legal representative or impartial witness).

The original copy of the signed ICF will be retained in the investigator's study file (ISF) and must be made available for monitoring, audit or inspection. A copy of the signed ICF is given to the patient.

In case of new information that might affect the patient's willingness to continue participating in the study or results in significant changes in the risk/benefit assessment, the ICF should be reviewed and updated if necessary. All patients, including those already being treated, should be informed of the new information, given a copy of the revised form, and give their consent to continue in the study.

18.3. Subject identification

The principal investigator or any authorised study member staff must assure that subjects' identity is maintained confidential and that their identities are protected from unauthorised parties. Personal medical information is always treated as confidential.

On CRFs or any other study document, subjects shall not be identified by their names or initials or social security numbers or patient chart numbers, but always by the assigned subject study identification codes and their dates of birth (either complete or partial as allowed by national laws) in order to avoid identification errors. If this information is asked on any study document which cannot be edited by the sponsor (e.g. in case of copyright), the investigator shall ensure that this information is neither reported on the CRF nor communicated by any mean to the sponsor.

The principal investigator should create and maintain up-to-date in the Investigator's Study File (ISF) the Subject screening/enrolment/identification log. This ICH-GCP log is required for documenting chronically any screened subject, their name, their assigned study number in case of study enrolment or the reason in case of non-enrolment. A note is made in the hospital medical records that the subject is participating in the study.

The name of a subject is neither asked for nor recorded by the sponsor. If subjects' names or any other confidential subject information are included by error on copies of documents submitted to the sponsor, they are obliterated and the assigned subject study identification code added to the document by the sponsor. Moreover, the sponsor asks to the principal investigator or any authorised study member staff to also obliterate this information on the documents that she has submitted.

Documents (e.g. subjects' written Informed Consent Forms) not for submission to sponsor should be maintained by the principal investigator in strict confidence.

19. DATA HANDLING AND RECORD KEEPING

19.1. Investigator's files and subject's clinical source documents

The principal investigator must maintain adequate and accurate records to enable the conduct of the study to be fully documented and the study data to be subsequently verified. These documents should be classified into two separate categories: (1) investigator's study file (ISF); and (2) subject clinical source documents.

The ISF should be established according to the ICH-GCP E6 and should include, among other documents, the site staff delegated study task log established by the principal investigator, the subject screening/enrolment and identification log and signed ICF.

Subject clinical source documents (paper or electronic) are the documents where the data are recorded for the first time and include, but are not limited to, subject hospital/clinic records; physician's and nurse's notes; appointment book; original laboratory reports; consultant letters and pharmacy dispensing records.

19.2. Data Quality Assurance

The sponsor is responsible for all data management activities of this study. This includes set-up and build of the electronic data capture system, quality checking of the database throughout study conduct, and study closeout.

The data related to the medical care of the subjects are recorded in the medical notes (source documents) by site staff. Thereafter the site staff enters in the eCRF only the data required to perform the statistical analyses.

The data related to the quality of life are collected on paper questionnaires (source documents). The data from the questionnaires are afterwards entered manually into the electronic data capture system by the site staff.

Site staff is responsible for manual data entry into the electronic data capture system. In the event of discrepant data, the sponsor issues queries to site staff, which site staff resolves electronically in the electronic data capture system in a timely manner.

The sponsor writes a Data Management Plan that describes the quality checks to be performed on the data.

The study data are transferred to the statistical team prior to any study committees data reviews or prior to analyses. The electronic transfer of data is processed by using a tool designed by the sponsor which ensures a secured transfer of the data in Statistical Analysis System (SAS) format.

19.3. Case Report Forms (CRF)

Data for this study are captured by using an electronic CRF (eCRF) via a sponsor designated electronic data capture system for each subject enrolled. The site receives training, an eCRF completion guideline for appropriate eCRF handling and completion and personal access code by the sponsor.

The eCRF should only be completed by designated and trained site staff and submitted electronically to sponsor.

The principal investigator is responsible for creating and maintaining up to date the site staff delegated study task log (named Signature Log in ICH GCP) mentioning the responsible persons completing, updating and/or signing the eCRF.

Once completed, the eCRF must be reviewed and electronically signed by the principal investigator or an authorised delegate from the study staff. This also applies to records for those subjects who fail to complete the study. If a subject withdraws from the study treatment or does not complete the follow-up, the reason must be noted in the eCRF. If a subject withdraws from the entire study, the withdrawal of consent should be noted in the eCRF.

The principal investigator should ensure the accuracy, completeness, legibility and timeliness of the data reported in the eCRF and in all required reports. Data reported in the eCRF must be derived from source documents and should be consistent with the source documents.

An audit trail will maintain a record of the initial entries and changes made, reasons for change (if necessary), time and date of entry, and user name of the person adding or changing an entry.

At the end of the study, the investigator will receive patient data entered in the eCRF for his or her site in a readable format that must be kept with the study records. Acknowledgement of receipt of these documents is required.

19.4. Source data documentation

The site staff should maintain adequate and accurate source documents, which should be attributable, legible, contemporaneous, original, accurate, and complete, as referenced in ICH E6 (R2) in Section 8.

Source documents (paper or electronic) are those in which patient data are recorded and documented for the first time. They include, but are not limited to, hospital records, clinical and office charts, laboratory notes, memoranda, quality of life questionnaires, evaluation checklists, pharmacy dispensing records, recorded data from automated instruments, copies of transcriptions that are certified after verification as being accurate and complete, microfiche, photographic negatives, microfilm or magnetic media, X-rays, patient files, and records kept at pharmacies, laboratories, and medico-technical departments involved in the study.

Before study initiation, the types of source documents that are to be generated is defined. This includes any protocol data to be entered directly into the eCRFs (i.e., no prior written or electronic record of the data) and considered source data.

Source documents that are required to verify the validity and completeness of data entered into the eCRFs must not be obliterated or destroyed and must be retained per the policy for retention of records described in Section 18.5.

When clinical observations are entered directly into a study site's computerized medical record system (i.e., in lieu of original hardcopy records), the electronic record can serve as the source document if the system has been validated in accordance with health authority requirements pertaining to computerized systems used in clinical research. An acceptable computerized data collection system allows preservation of the original entry of data. If original data are modified, the system should maintain a viewable audit trail that shows the original data as well as the reason for the change, name of the person making the change, and date of the change.

19.5. Retention of documents

Following closure of the study, the principal investigator must maintain all site study records, except for those required by local regulations to be maintained by someone else, in a safe and secure location. The records must be maintained to allow easy and timely retrieval, when needed (e.g., audit or inspection), and, whenever feasible, to allow any subsequent review of data in conjunction with assessment of the facility, supporting systems, and staff. Where permitted by local laws/regulations or institutional policy, some or all of these records can be maintained in a format other than hard copy (e.g., microfiche, scanned, electronic); however, caution needs to be exercised before such action is taken. The principal investigator must ensure that all reproductions are legible and are a true and accurate copy of the original and meet accessibility and retrieval standards, including re-generating a hard copy, if required. Furthermore, the principal investigator must ensure there is an acceptable back-up of these reproductions and that an acceptable quality control process exists for making these reproductions.

The sponsor informs the principal investigator of the time period for retaining these records to comply with all applicable regulatory requirements. The minimum retention time meets the strictest standard applicable to that participating site for the study, as dictated by any

institutional requirements or national laws or regulations; otherwise, the retention period will default to 20 years after the completion of the study and/or after approval by relevant Health Authorities, whichever is longer.

Should the principal investigator wish to assign the study records to another party or move them to another location, the sponsor must be notified in advance. If the principal investigator cannot guarantee this archiving requirement at the participating site for any or all of the documents, special arrangements must be made between the principal investigator and the sponsor to store these in a sealed container(s) outside of the participating site so that they can be returned sealed to the principal investigator, in case of a regulatory audit. Where source documents are required for the continued care of the subject; appropriate copies should be made before storing outside of the participating site.

19.6. Adequate arrangements to secure data compliant with GDPR

All “personal data” are treated in accordance with data protection laws, including the General Data Protection Regulation (GDPR).

Personal and clinical data related to our studies are stored on secured servers. Those servers protect data against loss, destruction, access, modification or dissemination by unauthorized persons. Only a limited and controlled number of persons are authorized to access the data. The Audit Trail function integrated in the OpenClinica 3.14 EDC system allows verifying that non authorized data modification didn’t occur.

All the personal data transferred over internet are submitted in coded form. We use SSL (Secure Socket Layer) encryption for the transmission of data.

To ensure the compliance with GDPR, our IT Department has implemented the following set of measures:

- A rigorous policy of password management
- A procedure of creation and deletion of user accounts. Access to the workstations and applications must be done using registered user accounts, and not “generic” (account1, compta2...), in order to be able to trace the actions done on a file and empower all stakeholders.
- This rule is also applying to systems and networks administrators of our organisation.
- For OpenClinica: a user listing is sent twice a year to Project Managers to verify that all user accesses are up to date.
- Secured workstations. Workstations are locked automatically after a period of inactivity (10 minutes). This measure is in place to restrict the risks of fraudulent use of an application in case of temporary absence of the user.
- A precise identification of persons who can access the files. Access to personal data processed in a file is limited to persons who can legitimately access them for the execution of their assigned tasks. A periodic check of application profiles and access rights on the servers is done to ensure the adequacy of the offered rights and the reality of the functions occupied by each user.
- A secured Local Area network. Institut Jules Bordet network is secured against external attacks. Logical safety devices such as filter routers (ACLs), firewalls, intrusion probes, etc. ensure a first level of protection. Reliable protection against viruses and spyware are constantly updated, both on the server and on the user’s workstations.
- Remote accesses to the information system are authenticated by the user and the post. Internet access to our tools needs to pass by strong security measures, including the use of IPsec, SSL/TLS, or HTTPS protocols.

- Secured physical access to facilities. Access to sensitive premises, such as rooms hosting computer servers and network components, is limited to authorized personnel. These premises are subject to special security: locked doors, digicode, and access controlled by name badges.
- Anticipating the risk of loss or disclosure of data. All of our servers are subjects to regular backup. The backup media are stored in a fireproof safe room separated from the one that hosts the servers. An "emergency-rescue" procedure describes how to quickly reassemble these servers in the event of a major crash or disaster.
- Information secure policy. All the rules related to computer security are formalized in a document (SOP) accessible to all users of our organisation.

19.7. Measures implemented in case of data security breach

19.7.1. The procedure if a Data Processor causes a data breach

All agreements with Data Processors stipulate that the Data Processor must notify every data breach to the Data Controller (the Sponsor). In the event a data breach occurs and that breach was caused by a Data Processor, the Sponsor shall assess with the Data Processor if the breach poses a risk to an individual's rights and freedoms. The risk assessment shall be performed with the assistance of the Data Processor and shall evaluate if a notification to the supervisory authority is necessary pursuant to articles 33 and 34 of the GDPR and the Guidelines 01/2021 on Examples regarding Personal Data Breach Notification (Adopted on 14 December 2021, version 2.0). The risk assessment shall be carried out using the methodology developed by the European Union Agency for Network and Information Security (ENISA) for personal data breach severity assessment.

The Sponsor shall contact the Data Processor to request a corrective and preventive actions plan. The Sponsor shall request the Data Processor to provide without undue delay the following information:

- The up-to-date name and contact details of the data protection officer (DPO) or other contact point where more information can be obtained;
- A description of the likely consequences of the personal data breach;
- A description of the measures taken or proposed to address the personal data breach, including, where appropriate, measures to mitigate its possible adverse effects.

19.7.2. Notification of the data breach

The Sponsor shall notify the supervisory authority without undue delay, and at the latest within 72 hours after having become aware of the breach, if the breach poses a risk to the individual's rights and freedoms. If the data breach only has minor consequences and no significant effect on the data subjects, the Sponsor shall not notify the supervisory authority but only document the breach by keeping record of the risk assessment, and filling the notification form for information.

If the data breach poses a high risk to those individuals affected, the Sponsor shall inform those individuals in clear, plain language without undue delay, unless there are effective technical and organizational protection measures that have been put in place, or other measures that ensure that risk is no longer likely to materialize. The individuals shall also be informed upon request from the supervisory authority.

The information to individuals include the DPO's details, a description of the likely consequences of the breach and the measures taken (including mitigating actions and any possible adverse effects). This information shall be given by the Data Processor where the confidentiality of the individuals should be preserved.

19.7.3. Risk assessment

The risk shall be assessed in accordance with the Guidelines 01/2021 and the methodology developed by ENISA. In particular, the risk assessment shall take into considerations:

If the breach derives from an intentional action or from an unintentional human error caused by inattentiveness;

- The quantity of data affected by the breach and the type of personal data
- The low or high amount of individuals affected
- The immediate detection of the breach
- If the receiving party is bound by professional secrecy, reported the breach himself and deleted the file and personal data upon request
- The relationship between the Sponsor and the receiving party

19.7.4. Measures to mitigate the adverse effect

Depending on the type of data breach, the measures taken to mitigate the risks may include the following:

- Limit the access to the most sensible data
- enforcing training, education and awareness programs
- avoiding data exchange through unsecure channels
- implementing measures to force user authentication when accessing sensitive personal data checking unusual dataflow between the file server and employee workstations
- Enforcing GDPR compliance program with security standards such as ISO 27000 series, COSO, CSA, CCM, HIPAA, NIST.

19.7.5. Record keeping

The Sponsor shall document any personal data breaches, comprising the facts relating to the personal data breach, its effects and the remedial action taken. That documentation shall enable the supervisory authority to verify compliance.

20. STUDY OVERSIGHT:

20.1. Scientific Committee

A scientific committee including the study chair, co-chair, Institut Jules Bordet study team, principal investigators and study team of participating sites will be constituted to monitor data

and overall safety to ensure that the study meets the highest standards of ethics and patient safety.

During the Phase I, the scientific committee will evaluate the safety, tolerability and all other data concerning the treatment with paclitaxel, carboplatin, durvalumab and oleclumab.

In Phase II, the same scientific committee is planned to review safety and events (relapse, deaths, premature treatment stops). For this purpose, teleconferences and or meetings as well as written reports are planned every 6 months. Additional teleconferences can be organized if necessary for safety concerns.

20.2. Independent Data Monitoring Committee

An Independent Data Monitoring Committee (IDMC) will be formed for this study to review data related to SYNERGY study. Sponsor employees, as well as other individuals involved in the design, setup, or conduct of the study, will be excluded from IDMC membership. The IDMC members shall be familiar with the methodology of oncology trials.

The outcome of this data review will be communicated in a timely manner to the sponsor. When necessary, the sponsor shall communicate the relevant information to investigators for notification of their respective Ethics Committees and/or for notification of the Regulatory Authority if required by the national laws of the countries where the study is conducted.

The IDMC will follow a charter that outlines the IDMC roles, responsibilities, operating procedures for the meetings and the flow of communication.

21. STUDY SPONSORSHIP AND FINANCING

Within the European Union, Institut Jules Bordet will be the legal sponsor of the Study, as the term is defined under the Regulation EU No 536/2014

Institut Jules Bordet is solely responsible for any and all safety reporting associated with the conduct of the Study, as well as any and all regulatory obligations associated with the conduct of the Study, except for those obligations expressly identified in specific Agreements made with third parties

Institut Jules Bordet
Rue Meylemeersch, 90, 1070 Bruxelles/Brussel
Belgique/België

This study is supported by a grant from AstraZeneca. AstraZeneca will also provide the durvalumab and oleclumab free of charge to all study subjects.

22. STUDY INSURANCE

The sponsor is responsible for taking out the insurance for the study according to Regulation EU No 536/2014 and also the laws of the countries where the study is conducted. An insurance certificate must be available to the participating sites at the time of study initiation. Clinical study insurance is only valid in participating sites authorised by the sponsor.

The insurance of the sponsor does not relieve the participating site and the principal investigator of any obligation to maintain their own liability insurance policy.

23. PUBLICATION POLICY

Publication and presentations of any results from this study shall be in accordance with accepted scientific practice, academic standards and customs and in accordance with the specific policy developed for this study if any.

The final publication of the study results is written by the study chairs or designated active principal investigators of the study on the basis of the final analysis performed at the sponsor headquarters (Institut Jules Bordet, Brussels, Belgium).

The sponsor recognises right of utilising data derived from the study for teaching purposes, communication at congresses and scientific publications.

Nevertheless, in order to ensure the accuracy and scientific value of the information, while preserving the independence and accountability of the study chair and the confidentiality of the information, only clear, checked and validated data shall be used. To that effect, it is essential that the study chair and the sponsor exchange and discuss, prior any publication or communication, any draft publication or communication made by the study chair.

All manuscripts shall include an appropriate acknowledgment section, mentioning all principal investigators who have contributed to the study, the staff of the Clinical Trials Support Unit involved in the study, as well as the supporting bodies. The number of acknowledgments per participating entity shall depend on the journal's rules and be based on fair and practical considerations.

24. REFERENCES

1. Foulkes, W. D., Smith, I. E. & Reis-Filho, J. S. Triple-Negative Breast Cancer. *New England Journal of Medicine* **363**, 1938–1948 (2010).
2. Dent, R. *et al.* Triple-Negative Breast Cancer: Clinical Features and Patterns of Recurrence. *Clinical Cancer Research* **13**, (2007).
3. Piccart-Gebhart, M. J. *et al.* Taxanes Alone or in Combination With Anthracyclines As First-Line Therapy of Patients With Metastatic Breast Cancer. *Journal of Clinical Oncology* **26**, 1980–1986 (2008).
4. Cardoso, F. *et al.* 3rd ESO–ESMO International Consensus Guidelines for Advanced Breast Cancer (ABC 3). *Annals of Oncology* **28**, mdw544 (2016).
5. Seidman, A. D. *et al.* Phase II trial of paclitaxel by 3-hour infusion as initial and salvage chemotherapy for metastatic breast cancer. *Journal of Clinical Oncology* **13**, 2575–2581 (1995).
6. Nabholtz, J. M. *et al.* Multicenter, randomized comparative study of two doses of paclitaxel in patients with metastatic breast cancer. *Journal of clinical oncology : official journal of the American Society of Clinical Oncology* **14**, 1858–67 (1996).
7. Harris, L. N. *et al.* Molecular subtypes of breast cancer in relation to paclitaxel response and outcomes in women with metastatic disease: results from CALGB 9342. *Breast cancer research : BCR* **8**, R66 (2006).
8. Seidman, A. D. *et al.* Randomized Phase III Trial of Weekly Compared With Every-3-Weeks Paclitaxel for Metastatic Breast Cancer, With Trastuzumab for all HER-2 Overexpressors and Random Assignment to Trastuzumab or Not in HER-2 Nonoverexpressors: Final Results of Cancer and Leukemia Group B Protocol 9840. *Journal of Clinical Oncology* **26**, 1642–1649 (2008).
9. Byrski, T. *et al.* Results of a phase II open-label, non-randomized trial of cisplatin chemotherapy in patients with BRCA1-positive metastatic breast cancer. *Breast Cancer Research* **14**, R110 (2012).
10. Tutt, A. *et al.* Carboplatin in BRCA1/2-mutated and triple-negative breast cancer BRCAness subgroups: the TNT Trial. *Nature Medicine* **24**, 628–637 (2018).

11. von Minckwitz, G. *et al.* Neoadjuvant carboplatin in patients with triple-negative and HER2-positive early breast cancer (GeparSixto; GBG 66): a randomised phase 2 trial. *The Lancet Oncology* **15**, 747–756 (2014).
12. Sikov, W. M. *et al.* Impact of the Addition of Carboplatin and/or Bevacizumab to Neoadjuvant Once-per-Week Paclitaxel Followed by Dose-Dense Doxorubicin and Cyclophosphamide on Pathologic Complete Response Rates in Stage II to III Triple-Negative Breast Cancer: CALGB 40603 (Alliance). *Journal of Clinical Oncology* **33**, 13–21 (2015).
13. Geyer, C. E. *et al.* Phase 3 study evaluating efficacy and safety of veliparib (V) plus carboplatin (Cb) or Cb in combination with standard neoadjuvant chemotherapy (NAC) in patients (pts) with early stage triple-negative breast cancer (TNBC). *Journal of Clinical Oncology* **35**, 520 (2017).
14. von Minckwitz, G. *et al.* Abstract S2-04: Early survival analysis of the randomized phase II trial investigating the addition of carboplatin to neoadjuvant therapy for triple-negative and HER2-positive early breast cancer (GeparSixto). *Cancer Research* **76**, S2-04-S2-04 (2016).
15. Sikov, W. *et al.* Abstract S2-05: Event-free and overall survival following neoadjuvant weekly paclitaxel and dose-dense AC +/- carboplatin and/or bevacizumab in triple-negative breast cancer: Outcomes from CALGB 40603 (Alliance). *Cancer Research* **76**, S2-05-S2-05 (2016).
16. Dear, R. F. *et al.* Combination versus sequential single agent chemotherapy for metastatic breast cancer. *Cochrane Database of Systematic Reviews* CD008792 (2013) doi:10.1002/14651858.CD008792.pub2.
17. Robson, M. *et al.* Olaparib for Metastatic Breast Cancer in Patients with a Germline *BRCA* Mutation. *New England Journal of Medicine* **377**, 523–533 (2017).
18. Litton, J. K. *et al.* Talazoparib in Patients with Advanced Breast Cancer and a Germline *BRCA* Mutation. *New England Journal of Medicine* **379**, 753–763 (2018).
19. Topalian, S. L. *et al.* Safety, activity, and immune correlates of anti-PD-1 antibody in cancer. *The New England journal of medicine* **366**, 2443–54 (2012).
20. Brahmer, J. R. *et al.* Safety and Activity of Anti–PD-L1 Antibody in Patients with Advanced Cancer. *New England Journal of Medicine* **366**, 2455–2465 (2012).
21. Hodi, F. S. *et al.* Improved survival with ipilimumab in patients with metastatic melanoma. *The New England journal of medicine* **363**, 711–23 (2010).
22. Solinas, C. *et al.* Targeting immune checkpoints in breast cancer: an update of early results. *ESMO Open* **2**, e000255 (2017).
23. Keir, M. E., Butte, M. J., Freeman, G. J. & Sharpe, A. H. PD-1 and Its Ligands in Tolerance and Immunity. *Annual Review of Immunology* **26**, 677–704 (2008).
24. Hui, E. *et al.* T cell costimulatory receptor CD28 is a primary target for PD-1–mediated inhibition. *Science* **355**, 1428–1433 (2017).
25. Dong, H. *et al.* Tumor-associated B7-H1 promotes T-cell apoptosis: a potential mechanism of immune evasion. *Nature medicine* **8**, 793–800 (2002).
26. Mittendorf, E. A. *et al.* PD-L1 expression in triple-negative breast cancer. *Cancer immunology research* **2**, 361–70 (2014).
27. Schalper, K. A. *et al.* In situ tumor PD-L1 mRNA expression is associated with increased TILs and better outcome in breast carcinomas. *Clin Cancer Res* **20**, 2773–2782 (2014).
28. Wimberly, H. *et al.* PD-L1 Expression Correlates with Tumor-Infiltrating Lymphocytes and Response to Neoadjuvant Chemotherapy in Breast Cancer. *Cancer Immunol Res* **3**, 326–332 (2015).
29. Nanda, R. *et al.* Pembrolizumab in Patients With Advanced Triple-Negative Breast Cancer: Phase Ib KEYNOTE-012 Study. *Journal of Clinical Oncology* **34**, 2460–2467 (2016).
30. Schmid, P., Cruz, C. & Braiteh, F. S. Atezolizumab in metastatic TNBC (mTNBC): Long-term clinical outcomes and biomarker analyses. (2017).

Confidential

31. Zitvogel, L., Galluzzi, L., Smyth, M. & Kroemer, G. Mechanism of Action of Conventional and Targeted Anticancer Therapies: Reinstating Immunosurveillance. *Immunity* **39**, 74–88 (2013).
32. Tolaney, S. *et al.* Phase 1b/2 study to evaluate eribulin mesylate in combination with pembrolizumab in patients with metastatic triple-negative breast cancer. in *San Antonio Breast Cancer Conference* Abstract PD6-13 (2017).
33. Tolaney, S. M. *et al.* A phase Ib/II study of eribulin (ERI) plus pembrolizumab (PEMBRO) in metastatic triple-negative breast cancer (mTNBC) (ENHANCE 1). *JCO* **38**, 1015–1015 (2020).
34. Adams, S. *et al.* Phase Ib trial of atezolizumab in combination with nab-paclitaxel in patients with metastatic triple-negative breast cancer (mTNBC). *Journal of Clinical Oncology* **34**, (2016).
35. Schmid, P. *et al.* Atezolizumab and Nab-Paclitaxel in Advanced Triple-Negative Breast Cancer. *New England Journal of Medicine* NEJMoa1809615 (2018) doi:10.1056/NEJMoa1809615.
36. Schmid, P. *et al.* Atezolizumab plus nab-paclitaxel as first-line treatment for unresectable, locally advanced or metastatic triple-negative breast cancer (IMpassion130): updated efficacy results from a randomised, double-blind, placebo-controlled, phase 3 trial. *The Lancet. Oncology* **21**, 44–59 (2020).
37. Cortes, J. *et al.* Pembrolizumab plus chemotherapy versus placebo plus chemotherapy for previously untreated locally recurrent inoperable or metastatic triple-negative breast cancer (KEYNOTE-355): a randomised, placebo-controlled, double-blind, phase 3 clinical trial. *Lancet* **396**, 1817–1828 (2020).
38. Hao, D. *et al.* A Canadian Cancer Trials Group phase IB study of durvalumab with or without tremelimumab + standard platinum-doublet chemotherapy in patients with advanced, incurable solid malignancies (IND.226). *European Journal of Cancer* **69**, S95 (2016).
39. Bendell, J. C. *et al.* A phase 1 study to evaluate the safety, tolerability, pharmacokinetics, immunogenicity, and antitumor activity of MEDI9447 alone and in combination with durvalumab (MEDI4736) in patients with advanced solid tumors. *Journal of Clinical Oncology* **34**, TPS3096–TPS3096 (2016).
40. Loibl, S. *et al.* A randomized phase II neoadjuvant study (GeparNuevo) to investigate the addition of durvalumab, a PD-L1 antibody, to a taxane-anthracycline containing chemotherapy in triple negative breast cancer (TNBC). *Journal of Clinical Oncology* **35**, 3062 (2017).
41. Loibl, S. *et al.* Randomized phase II neoadjuvant study (GeparNuevo) to investigate the addition of durvalumab to a taxane-anthracycline containing chemotherapy in triple negative breast cancer (TNBC). *Journal of Clinical Oncology* **36**, 104 (2018).
42. Bachelot, T. *et al.* Durvalumab compared to maintenance chemotherapy in metastatic breast cancer: the randomized phase II SAFIR02-BREAST IMMUNO trial. *Nat Med* (2021) doi:10.1038/s41591-020-01189-2.
43. Powles, T. *et al.* Efficacy and Safety of Durvalumab in Locally Advanced or Metastatic Urothelial Carcinoma. *JAMA Oncology* **3**, e172411 (2017).
44. Stagg, J. & Smyth, M. J. Extracellular adenosine triphosphate and adenosine in cancer. *Oncogene* **29**, 5346–5358 (2010).
45. Allard, B. *et al.* Anti-CD73 therapy impairs tumor angiogenesis. *International journal of cancer. Journal international du cancer* **134**, 1466–73 (2014).
46. Stagg, J. *et al.* CD73-deficient mice have increased antitumor immunity and are resistant to experimental metastasis. *Cancer research* **71**, 2892–900 (2011).
47. Buisseret, L. *et al.* Clinical significance of CD73 in triple-negative breast cancer: multiplex analysis of a phase III clinical trial. *Annals of Oncology* (2017) doi:10.1093/annonc/mdx730.

48. Loi, S. *et al.* CD73 promotes anthracycline resistance and poor prognosis in triple negative breast cancer. *Proceedings of the National Academy of Sciences of the United States of America* **110**, 11091–6 (2013).
49. Samanta, D. *et al.* Chemotherapy induces enrichment of CD47 + /CD73 + /PDL1 + immune evasive triple-negative breast cancer cells. *Proceedings of the National Academy of Sciences* **115**, E1239–E1248 (2018).
50. Allard, B., Pommey, S., Smyth, M. J. & Stagg, J. Targeting CD73 enhances the antitumor activity of anti-PD-1 and anti-CTLA-4 mAbs. *Clinical cancer research : an official journal of the American Association for Cancer Research* **19**, 5626–35 (2013).
51. Hay, C. M. *et al.* Targeting CD73 in the tumor microenvironment with MEDI9447. *Oncoimmunology* **5**, e1208875 (2016).
52. Emens, L. *et al.* CPI-444, an oral adenosine A2a receptor (A2AR) antagonist, demonstrates clinical activity in patients with advanced solid tumors. *Abstract CT119, AACR 2017*.
53. Desmedt, C. *et al.* Biological processes associated with breast cancer clinical outcome depend on the molecular subtypes. *Clinical cancer research : an official journal of the American Association for Cancer Research* **14**, 5158–65 (2008).
54. Loi, S. *et al.* Prognostic and predictive value of tumor-infiltrating lymphocytes in a phase III randomized adjuvant breast cancer trial in node-positive breast cancer comparing the addition of docetaxel to doxorubicin with doxorubicin-based chemotherapy: BIG 02-98. *Journal of clinical oncology : official journal of the American Society of Clinical Oncology* **31**, 860–7 (2013).
55. Galluzzi, L., Zitvogel, L. & Kroemer, G. Immunological Mechanisms Underneath the Efficacy of Cancer Therapy. *Cancer Immunology Research* **4**, 895–902 (2016).
56. Beavis, P. A. *et al.* Adenosine Receptor 2A Blockade Increases the Efficacy of Anti-PD-1 through Enhanced Antitumor T-cell Responses. *Cancer Immunology Research* **3**, 506–517 (2015).
57. Mittal, D. *et al.* Antimetastatic Effects of Blocking PD-1 and the Adenosine A2A Receptor. *Cancer Research* **74**, 3652–3658 (2014).
58. Iannone, R., Miele, L., Maiolino, P., Pinto, A. & Morello, S. Adenosine limits the therapeutic effectiveness of anti-CTLA4 mAb in a mouse melanoma model. *American journal of cancer research* **4**, 172–81 (2014).
59. van Warmerdam, L. J., Rodenhuis, S., ten Bokkel Huinink, W. W., Maes, R. A. & Beijnen, J. H. The use of the Calvert formula to determine the optimal carboplatin dosage. *Journal of cancer research and clinical oncology* **121**, 478–86 (1995).
60. Wang, D. D., Zhang, S., Zhao, H., Men, A. Y. & Parivar, K. Fixed dosing versus body size-based dosing of monoclonal antibodies in adult clinical trials. *Journal of clinical pharmacology* **49**, 1012–24 (2009).
61. Ng, C. M., Lum, B. L., Gimenez, V., Kelsey, S. & Allison, D. Rationale for Fixed Dosing of Pertuzumab in Cancer Patients Based on Population Pharmacokinetic Analysis. *Pharmaceutical Research* **23**, 1275–1284 (2006).
62. Allison, K. H. *et al.* Estrogen and Progesterone Receptor Testing in Breast Cancer: ASCO/CAP Guideline Update. *Journal of clinical oncology : official journal of the American Society of Clinical Oncology* JCO1902309 (2020) doi:10.1200/JCO.19.02309.
63. Wolff, A. C. *et al.* Human Epidermal Growth Factor Receptor 2 Testing in Breast Cancer: American Society of Clinical Oncology/College of American Pathologists Clinical Practice Guideline Focused Update. *Journal of clinical oncology : official journal of the American Society of Clinical Oncology* **36**, 2105–2122 (2018).
64. Eisenhauer, E. A. *et al.* New response evaluation criteria in solid tumours: revised RECIST guideline (version 1.1). *European Journal of Cancer (Oxford, England: 1990)* **45**, 228–247 (2009).
65. Seymour, L. *et al.* iRECIST: guidelines for response criteria for use in trials testing immunotherapeutics. *The Lancet Oncology* **18**, e143–e152 (2017).

66. Seymour, L. *et al.* iRECIST: guidelines for response criteria for use in trials testing immunotherapeutics. *The Lancet Oncology* **18**, e143–e152 (2017).
67. Tolaney, S. *et al.* Abstract P5-15-02: Phase 1b/2 study to evaluate eribulin mesylate in combination with pembrolizumab in patients with metastatic triple-negative breast cancer. *Cancer Research* **77**, P5-15-02-P5-15–02 (2017).

APPENDIX 1: TOXICITY MANAGEMENT GUIDELINES

This document is provided by AstraZeneca and is available in a separate document. This is a general guidelines and some information present in this appendix is not applicable for the SYNERGY study.

APPENDIX 2: PRIVACY GUARANTEE

Personal data

If a patient participates in the study, his/her personal data will be processed; "processing" includes the collection, storage, structuring, transmission and any other use or provision. Personal data includes the following information:
- information that identifies the patient directly (such as his/her name, address, telephone number, or health insurance number);
- the age and sex of the patient;
- information relating to the patient's state of health, including medical history;
- the patient's medical treatments and his/her response to medical treatments;
- for women who become pregnant during the study: data about the pregnancy and childbirth;
- information about the patient's biological samples and the results of analyses performed on these;
- information about the patient's medical images and the results of the evaluation of these.

There will be different groups of data:
- The data group containing all data about the patient collected at the study hospital, including information that identifies him/her directly. This data group is called "**unencoded data**" and will be kept only in the study hospital's medical records.
- There will also be groups of "**encoded data**". The principle of data encoding entails that if a patient participates in the study, a code will be assigned to him/her. It will be unique, sequential and composed of 4 digits and it will replace all directly (surname, first name) or indirectly (telephone number or other number) identifying data on documents used for data collection or biological samples collected for the study. This encoding must be applied before any transmission to a third party (the manager of the database collected here is the sponsor, i.e. Institut Jules Bordet, Brussels, Belgium).

Legal basis for the processing of patient data

The protection of patient data and the related rights are guaranteed by the General Data Protection Regulation (European Regulation 2016/679), by the law of 22 August 2002 concerning patient rights in Belgium, by the law of 30 July 2018 concerning the data privacy in Belgium as well as any (new) applicable legislation in the participating countries.

Data controller of patient data

The sponsor (Institut Jules Bordet, Brussels, Belgium) is the data controller of the patient's encoded data. The investigator and the hospital are, on the one hand, subcontractors of the sponsor with regard to the processing of the patient's encoded data and, on the other hand,

data controllers of the patient's unencoded data collected both as part of the study and outside it.

Consultation and use of unencoded data

Unencoded data can be consulted by the investigator and other individuals from the hospital who are working on the study or providing care to the patient. Individuals accessing the data are subject to professional secrecy. In addition, a limited number of the sponsor's employees, or its contractual partners, ethics committees and regulatory authorities may consult the unencoded data, but solely for the purpose of verifying that the study is being conducted properly. These persons are bound by an obligation of confidentiality.

Consultation and use of encoded data

Encoded data will be transmitted to the sponsor. The encoded data can also be transmitted to service providers on the basis of contractual agreements. In addition, the encoded data may be shared with one or more partners collaborating with the sponsor with respect to the investigational drug and the diagnostic tests associated with this study's field of research.

The aforementioned recipients may use the encoded data for the following purposes:

- to answer scientific questions;
- to learn more about the investigational drug and how this group of drugs (i.e. drugs that may act in a similar way in the body) acts;
- to learn more about cancer and related health issues, and to develop corresponding diagnostic tests;
- to contribute to the correct planning of future studies;
- to develop scientific analysis methods;
- to publish the anonymised results of the study in scientific articles or presentations and to use them for educational purposes;
- to provide the sponsor of the study with informational materials on the study, the investigational drug, cancer and related health issues and diagnostic tests.

The patient's encoded data may be transmitted to regulatory bodies (including ethics committees) for regulatory purposes, for example for reporting relevant safety information, in order to receive marketing authorisation or for discussions on the reimbursement and marketing of the drug being tested and diagnostic tests. Regulatory bodies may also use the encoded data to learn more about cancer and related health issues.

Transfer of personal data to other countries

Some recipients of the data may be located outside your country and outside the European Economic Area (United States, for example). These may be countries whose level of data protection has not been confirmed by the European Commission as adequate. In this case, security measures equivalent to the security measures required by Belgian and European regulations will be taken in order to protect patient rights in terms of data confidentiality, by entering into specific contractual agreements.

Storage of encoded data

Encoded data will be kept for at least 25 years after the end of the study. Subsequently, they may be kept for an additional period of time, for the above-mentioned scientific purposes or for any legal reason (change of obligations with regard to storage, for example).

Patient rights related to personal data

The patient may exercise the following rights related to his/her personal data:

- Request information about the processing of data about him/her; however, she may not be able to access some data before the end of the study, without the risk of being excluded from the study. This measure may be necessary to protect the scientific integrity of the study.
- Request the correction of the data about him/her if they are incorrect or incomplete. The patient has the right to restrict the processing of data about him/her.
- Request the transfer of his/her personal data to himself/herself or to someone else in a commonly usable format.
- Withdraw his/her consent at any time without giving a reason. The patient also has the right to end his/her participation in the study by no longer coming, without having to justify himself/herself. However, this is not withdrawal in this case. Withdrawal means that the patient is actively withdrawing from the study and withdraws consent for data processing. This shall not affect the legality of processing data about him/her based on his/her consent given before withdrawal. However, after his/her withdrawal, data about him/her will no longer be collected.
- At the same time as his/her withdrawal, the patient has the right to request the erasure of data about him/her if it is no longer necessary for the purposes of processing or if there is no other legal basis for their further processing.
- The patient also has the right to lodge a complaint concerning the way in which his/her data is processed with the supervisory authority of his country responsible for ensuring compliance with data protection legislation.

If the patient wishes to exercise one of his/her rights relating to data about him/her or to know more about the measures to protect his/her personal data, she can send a request to the investigator or to the data protection officer of the study hospital.

Fragmentation patterns of plasma cell-free DNA for ctDNA detection and quantification. Healthy subject cohort.

Study chair: Michail Ignatiadis, MD, PhD
Co-chair: Andrea Gombos, MD

Investigators:
Jean-Benoit Burrion, MD Cancer Prevention and Screening Clinic, Institute Jules Bordet
Claire Lefere, MD Cancer Prevention and Screening Clinic, Institute Jules Bordet
Andreas Papagiannis, PhD student (Promoter Michail Ignatiadis)

1. Background:

1.1 <u>Liquid biopsy in advanced breast cancer:</u>

Circulating tumor DNA (ctDNA) is found in the plasma of a substantial majority of patients with advanced cancer and represents a source of cancer DNA for analysis of tumor somatic genetic features. In contrast with tumor biopsies which are invasive and potentially limited by tumour heterogeneity and temporal tumour evolution, liquide biopsies could offer a less invasive and more contemporary monitoring of evolution of somatic alterations during treatment. (1)
Recent prospective data shows evidence that ctDNA testing in metastatic breast cancer is highly accurate, with high agreement between different ctDNA testing techniques, and high sensitivity for mutations identified in advanced breast cancer tissue biopsies.
The recently published plasmaMATCH trial which used droplet digital PCR based ctDNA assessment of four genes (PIK3CA, HER2, AKT1 and ESR1) concluded to a high concordance (98%) with contemporaneous tissue sequencing when this was available. A cohort of patients underwent retrospective TGS sequencing in plasma (Guardant360°- 73 gene panel), showing again a 95% sensitivity to detect tissue mutations. Authors conclude that ctDNA testing offers accurate, rapid genotyping that enables the selection of mutation-directed therapies for patients with breast cancer. Based on the degree of accuracy observed ctDNA assessment could replace tissue-based mutation analysis. (2) The same trial demonstrated that testing for multiple individually rare pathogenic mutations can generate useful therapeutic 'hits' for a substantial percentage of patients (35%)(2)
Another large prospective trial, the AURORA molecular screening program uses a 40 gene TGS panel to identify somatic mutations in sequential plasma samples collected from metastatic breast cancer patients. Results are compared with TGS (oncoDEEP panel - OncoDNA®) of contemporary metastatic tumor biopsies and primary tumors. According to the first results published (ctDNA sequencing available in 90 patients), 60% of patients had at least one mutation detected in ctDNA at baseline. Out of 77 genomic alterations detected in one or both tissue samples (primary or metastatic), 46 (60%) were found in cfDNA. The variants not detected in cfDNA had a statistically significant lower variant allele frequency (VAF) in the tissue samples. Tier I or II mutations as per the ESMO Scale of Clinical Actionability for molecular Targets (ESCAT) (PIK3CA, ESR1, AKT1, HER2) were detected in ctDNA in majority of cases (89%). (3)

Furthermore, the use of ctDNA can be a valuable tool to identify resistance alterations with serial blood sampling. However, there are limited data so far on this approach in breast cancer. The most of the evidence so far is in the setting of HR+ MBC where endocrine +/- targeted therapy resistance has led to the development of clones harbouring ESR1, RB1, PIK3CA, ERBB2 and FGFR alterations. (4)(5)(6)(7)(8) Further prospective data is needed to establish the clinical utility of serial ctDNA as a tool for monitoring resistance molecular alterations in MBC and the development of more sensitive techniques is warranted developed for quantitative assessment of ctDNA during disease evolution.

1.2 Liquid biopsy in early breast cancer (EBC):

In EBC, patient specific assays (so called personalized ctDNA assays) have been assessed to track minimal residual disease during follow-up after completion of surgery and systemic (neo)adjuvant chemotherapy. Using these assays, ctDNA has been detected about 1 year before imaging diagnosis of relapse. (9)(10) Currently there are three available personalized ctDNA assays (the Signatera assay, Natera Inc; the Radar assay, Inivata Inc and ArcherDX) which rely on the analysis of the primary tumor and normal DNA using exome sequencing to detect a set of clonal, tumor specific, Single Nucleotide Variants (SNVs) that will be tracked using high coverage next generation sequencing (NGS) of plasma cell free DNA in serial blood samples. This provides a window for treating patients who present with what we have called 'ctDNA relapse': patients that become ctDNA-positive during follow-up but have no detectable metastatic disease using standard imaging. (1) Several clinical trials are currently being developed to investigate if starting early systemic treatment in patients showing molecular relapse can improve outcome.

1.3 Fragmentation patterns of plasma cell-free DNA as an emerging tool for ctDNA detection and quantification

Recently, Cristiano et al reported that genome wide fragmentation patterns of plasma cell-free DNA are different between healthy individuals and patients with cancer. (11) The same investigators developed a tool for early cancer detection using low-coverage WGS of plasma cell-free DNA followed by machine learning approaches and were able to classify an individual as having cancer or not based on the fragmentation patterns of plasma cell-free DNA with high specificity (98%) and sensitivity that ranged from 57-100% depending on the cancer type. (11) In another study by Mouliere et al. the authors having collected 344 plasma samples from 200 patients with multiple cancer types and 65 healthy controls, showed that by utilizing supervised machine learning algorithms trained on features such as the proportion of fragments in different sizes as well as the ratios of proportions of fragments in different sizes, they were able to detect the presence or absence of ctDNA. More specifically, their random forests (RF) model trained on 153 samples on classifying cancerous vs healthy samples displayed an AUC = 0.908. In their work, they also examined the proportion of fragments below 150 base pairs comparing healthy and cancerous samples from multiple cancer types. The proportion of fragments below 150 base pairs was significantly higher in breast cancer compared to healthy samples (12).

The substantial advantage of the above methods for estimating the ctDNA is that they are independent of primary tumor availability, are considerably less costly than personalized

ctDNA assays (described in 1.2) and can all be performed by analysing data from low-coverage WGS of plasma cfDNA. However, the above assays are only providing information on the presence or not of ctDNA but not on the fraction of circulating tumor DNA in total cell free DNA.
We already performed estimation of ctDNA fraction from low-coverage (0.1x) WGS using the fragmentation ratio in a cohort of 48 metastatic ER+/HER2- BC patients treated with exemestane and everolimus in the Pearl study (ClinicalTrials.gov Identifier: NCT02028364). The fragmentation ratio of each sample is defined as the ratio of the number of fragments of length less than 150 base pairs to the number of fragments with length equal or greater than 150 base pairs. Figure 1 displays the correlation of ctDNA fraction using the fragmentation ratio as a proxy to progression-free survival (PFS). Indeed, higher fragmentation ratio is associated with shorter PFS.

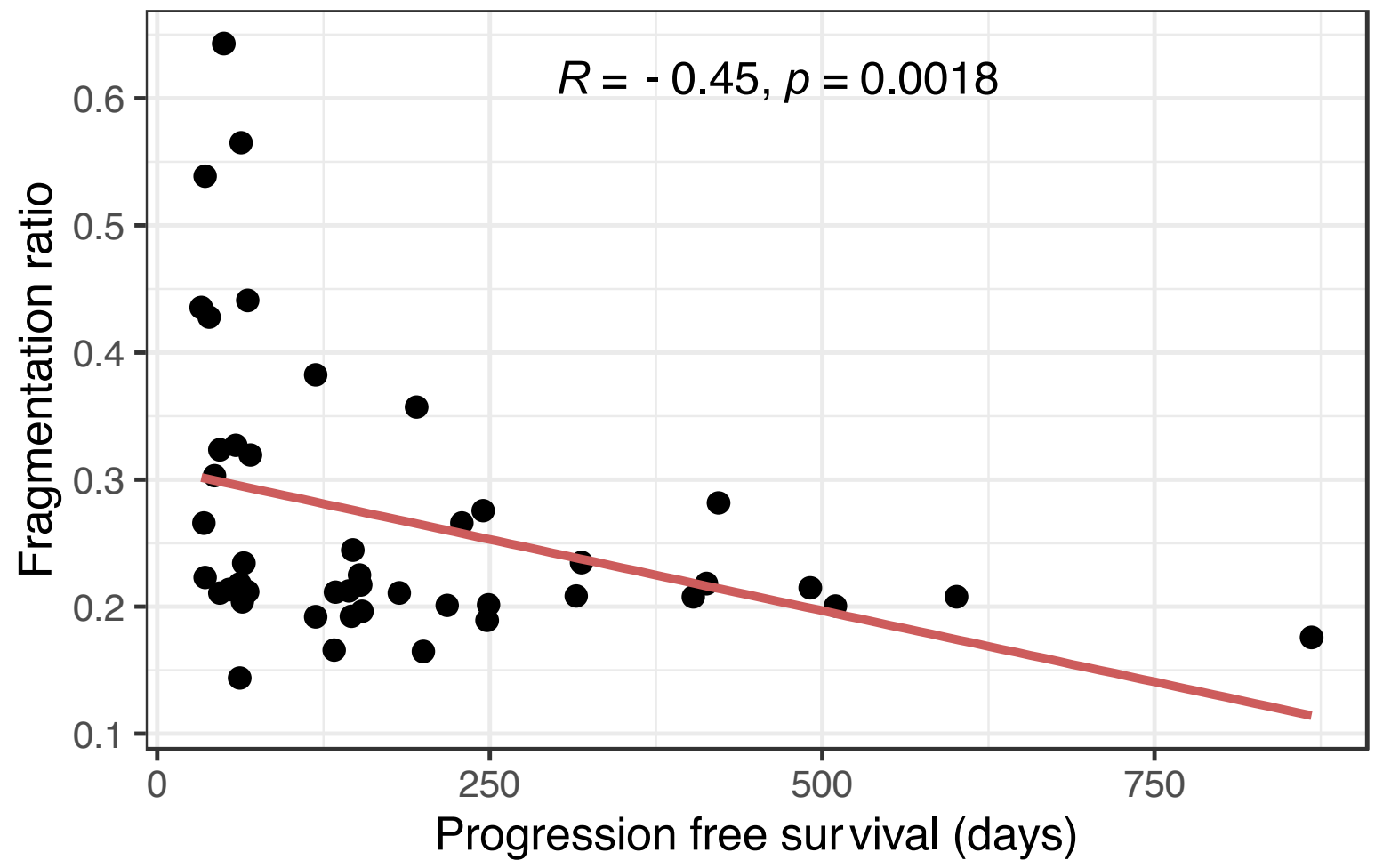

Figure 1. Correlation of fragmentation ratio as a proxy of ctDNA fraction and progression free survival within the context of the Pearl study. On the y-axis the fragmentation ratio is displayed while the x-axis depicts the number of days without tumour progression. Each dot represents a Pearl baseline sample (n = 48). The Spearman correlation coefficient is displayed along with the p-value.

2. Endpoint:

To reliably quantify the circulating tumor DNA fraction and develop a ctDNA detection and quantification ALgorithm that captures Fragmentation patterns of plasma cell-free DNA using mAchine learning Approaches (ctDNA ALFA assay)

3. Objectives:

Primary:
To develop an algorithm for ctDNA detection (yes vs no) and for the estimation of ctDNA fraction (percentage,%) by comparing fragmentation patterns of plasma cell-free DNA between healthy women and women with EBC enrolled in the NeoRHEA (NCT03065621) neoadjuvant trial (ctDNA ALFA assay).

Secondary:

a. To evaluate associations between ctDNA detection and ctDNA fraction estimation using the ctDNA ALFA assay and event free survival (EFS) in patients with EBC that have been enrolled in 2 neoadjuvant studies (NeoRHEA, NCT03065621 and a blood circulating biomarkers neoadjuvant study, Institute Jules Bordet – CE2557)

b. To evaluate associations between ctDNA detection and fraction estimation using the ctDNA ALFA assay and progression free survival (PFS) in patients with MBC that have been enrolled in the Pearl study (NCT02028364).

c. To evaluate whether the ctDNA ALFA assay adds prognostic information to personalised ctDNA assays such as Signatera (Natera Inc), Radar (Inivata Inc) in EBC and the OncoFOLLOW (OncoDNA Inc) in MBC in the above studies, respectively.

4. Steps for the development and validation of the ctDNA ALFA assay

4.1 <u>Development of the ctDNA ALFA assay:</u>

We will develop the ctDNA ALFA assay by comparing the fragmentation patterns of plasma cell-free DNA (cfDNA) from 100 healthy women with 100 women with early breast cancer enrolled in the NeoRHEA study.

Healthy women (matched for their age and menopausal status with the patient population of NeoRHEA) will be the controls. These women will be asked to donate one baseline plasma sample.

Eligible healthy women will be identified via Institut Jules Bordet's cancer screening clinic.

Inclusion criteria:

- having negative results of screening mammography realised within maximum 2 years before recruitment
- pre- and postmenopausal women
- written informed consent

Exclusion criteria:

- prior history of cancer
- known genetic predisposition of cancer
- current active infectious or autoimmune disease.

Women will be followed on a yearly basis during 3 years to exclude cancer diagnosis up to 3 years from blood collection. All blood samples will be analysed at the end of the study when all healthy women will be enrolled. In case of abnormal cfDNA findings, they will be promptly contacted for a consultation and an imaging check-up will be proposed.

Women with EBC enrolled in the completed NeoRHEA study will serve as the cases.

NeoRHEA is a phase 2 trial that have enrolled patients with ER+/HER2- breast cancer who received 4 months of neoadjuvant endocrine treatment in combination with the CDK4/6 inhibitor palbociclib. For each woman enrolled in the NeoRHEA study, we collected blood samples for ctDNA detection at 4 time points: baseline (pre-treatment), after 1 and 4 months of neoadjuvant treatment and post-surgery. Only the baseline plasma sample from the women enrolled in the NeoRHEA study will be used for the development of the ctDNA ALFA assay.

4.2 Assessement of the prognostic performance of ctDNA ALFA assay and comparison with commercial ctDNA assays in 3 clinical studies:

Once, we will develop the ctDNA ALFA assay, we will evaluate its prognostic performance in 3 clinical studies. First, we will evaluate associations between ctDNA detection/quantification by the ctDNA ALFA assay at each of the 4 time-points of blood sample collection in the NeoRHEA study and EFS (400 plasma samples from 100 patients).

Then, we will evaluate associations between ctDNA monitoring by the ctDNA ALFA assay at and EFS for patients enrolled in a blood circulating biomarkers neoadjuvant study, Institute Jules Bordet – CE2557. In this translational study, 44 patients with EBC, candidates for neoadjuvant chemotherapy have been enrolled. All patients received anthracyline and taxane based neoadjuvant chemotherapy. We collected blood samples for circulating biomarkers at 3 timepoints: before the initiation of neoadjuvant chemotherapy (baseline), after neoadjuvant chemotherapy, and before surgery (pre-surgery), and post-surgery (132 plasma samples from 44 patients).

Finally, we will evaluate associations between between ctDNA monitoring by the ctDNA ALFA assay and PFS for patients enrolled in the Pearl study. This a phase 2 study that enrolled 48 patients diagnosed with ER+ metastatic breast cancer who have been treated with exemestane – everolimus (mTOR inhibitor). Plasma samples were collected at 2 timepoints: baseline (pre-treatment) and after 2 weeks of treatment with exemestane and everolimus (96 plasma samples from 48 patients).

As a next step, we will compare the prognostic performance of ctDNA monitoring using the ctDNA ALFA assay with the performance of three commercially available assays in the same plasma samples:

- The Radar assay (Inivata Inc) will be performed in the 400 plasma samples from the 100 patients [6] in the NeoRHEA study.
- The Signatera assay (Natera Inc) [7] that has been already performed in 132 plasma samples from 44 patients enrolled in the circulating biomarkers IJB CE2557 study
- The OncoFOLLOW assay (OncoDNA, Inc), a 40-gene breast cancer specific gene panel that has been already performed in 96 plasma samples from 48 patients enrolled in the Pearl study.

We will also explore whether ctDNA monitoring using the ctDNA ALFA assay can improve the prognostic performance of the above commercially available assays in the 3 respective studies.

4.3 Prospective Independent validation

If the objectives of our project are met, we plan to protect the intellectual property associated with the development of our ctDNA ALFA assay with the help of the technology transfer office of the Université Libre de Bruxelles. Then, we will perform a prospective and independent validation of the ctDNA ALFA assay as a tool for early detection of disease relapse and development of 2nd cancers in patients with previous diagnosis of breast cancer and genetic predisposition to develop breast or other cancers.

5. Material and methods

5.1 Patients and samples

Healthy women (n=100), matched for their age and menopausal status with the patient population of NeoRHEA will be identified in a single center via the Institut Jules Bordet's cancer screening clinic (see I/E criteria above). Eligible women will be informed by one of the investigators of the current project. After a written informed consent, they be asked to donate one baseline plasma sample. For each healthy subject a total of 40 ml of blood will be collected in EDTA tubes for plasma isolation.
We estimate that the enrolment of the 100 healthy subjects will be completed in 6 month. Women will be followed annually during 3 years via a phone call in order to exclude cancer diagnosis.
There are no immediate safety concerns for the women enrolled.

All plasma samples will be analysed at the end of the study when enrolment is completed. We will perform whole genome sequencing (WGS) of plasma cell-free DNA that will be used for the evaluation of fragmentation patterns using machine-learning techniques. In the case of abnormal cfDNA findings, women will be promptly contacted for a consultation with a medical oncologist and an imaging check-up will be proposed.
Cell-free DNA from plasma samples will be sequenced in collaboration with the BRIGHTcore facilities.

6. Statistical considerations

This case control study (1:1) will compare fragmentation patterns of plasma cell-free DNA between healthy women and women with EBC enrolled in the NeoRHEA neoadjuvant trial using machine learning techniques.

Associations between the ctDNA ALFA assay and clinical outcome will be performed using Cox proportional-hazards regression analyses without (univariate) and with (multivariate) adjustment for baseline patient clinicopathological characteristics. The value of the ctDNA ALFA assay to predict clinical outcome will be evaluated using c-Index analyses. C-index (also known as the concordance probability) is the frequency of concordant pairs among all pairs of subjects. A pair of patients is called concordant if the risk of the event predicted by a model is lower for the patient who experiences the event at a later timepoint. As such, greater c-Index values, indicate a better model fit when comparing distinct Cox

proportional-hazards models. C-Index will also be used in order to evaluate the added prognostic value of the ctDNA ALFA assay compared to commercial ctDNA assays.

References:

1. Ignatiadis M, Sledge GW, Jeffrey SS. Liquid biopsy enters the clinic - implementation issues and future challenges. Nat Rev Clin Oncol. 2021;18:297–312.

2. Turner NC, Kingston B, Kilburn LS, Kernaghan S, Wardley AM, Macpherson IR, et al. Circulating tumour DNA analysis to direct therapy in advanced breast cancer (plasmaMATCH): a multicentre, multicohort, phase 2a, platform trial. The Lancet Oncology. Elsevier; 2020;21:1296–308.

3. Aftimos P, Oliveira M, Irrthum A, Fumagalli D, Sotiriou C, Gal-Yam EN, et al. Genomic and transcriptomic analyses of breast cancer primaries and matched metastases in AURORA, the Breast International Group (BIG) molecular screening initiative. Cancer Discov [Internet]. American Association for Cancer Research; 2021 [cited 2021 Aug 22]; Available from: https://cancerdiscovery.aacrjournals.org/content/early/2021/06/29/2159-8290.CD-20-1647

4. Fribbens C, Garcia Murillas I, Beaney M, Hrebien S, O'Leary B, Kilburn L, et al. Tracking evolution of aromatase inhibitor resistance with circulating tumour DNA analysis in metastatic breast cancer. Ann Oncol. 2018;29:145–53.

5. O'Leary B, Cutts RJ, Liu Y, Hrebien S, Huang X, Fenwick K, et al. The Genetic Landscape and Clonal Evolution of Breast Cancer Resistance to Palbociclib plus Fulvestrant in the PALOMA-3 Trial. Cancer Discov. 2018;8:1390–403.

6. Razavi P, Chang MT, Xu G, Bandlamudi C, Ross DS, Vasan N, et al. The Genomic Landscape of Endocrine-Resistant Advanced Breast Cancers. Cancer Cell. 2018;34:427-438.e6.

7. Razavi P, dos Anjos CH, Brown DN, Qing L, Ping C, Herbert J, et al. Molecular profiling of ER+ metastatic breast cancers to reveal association of genomic alterations with acquired resistance to CDK4/6 inhibitors. JCO. Wolters Kluwer; 2019;37:1009–1009.

8. Jacob S, Davis AA, Gerratana L, Velimirovic M, Shah AN, Wehbe F, et al. The Use of Serial Circulating Tumor DNA to Detect Resistance Alterations in Progressive Metastatic Breast Cancer. Clin Cancer Res. 2021;27:1361–70.

9. Coombes RC, Page K, Salari R, Hastings RK, Armstrong A, Ahmed S, et al. Personalized Detection of Circulating Tumor DNA Antedates Breast Cancer Metastatic Recurrence. Clin Cancer Res. 2019;25:4255–63.

10. Garcia-Murillas I, Schiavon G, Weigelt B, Ng C, Hrebien S, Cutts RJ, et al. Mutation tracking in circulating tumor DNA predicts relapse in early breast cancer. Science Translational Medicine. American Association for the Advancement of Science; 2015;7:302ra133-302ra133.

11. Cristiano S, Leal A, Phallen J, Fiksel J, Adleff V, Bruhm DC, et al. Genome-wide cell-free DNA fragmentation in patients with cancer. Nature. 2019;570:385–9.

12. Mouliere F, Chandrananda D, Piskorz AM, Moore EK, Morris J, Ahlborn LB, et al. Enhanced detection of circulating tumor DNA by fragment size analysis. Sci Transl Med. 2018;10:eaat4921.
