# Supplementary material for: ALFAssay: A feed‑forward neural network for quantitative fragmentomics‑based ctDNA profiling in breast cancer
Source: PLoS Comput Biol. 2026 Jul 27;22(7):e1014505. doi: 10.1371/journal.pcbi.1014505 (PMC13450836; doi:10.1371/journal.pcbi.1014505)
Supplement: Supplementary file 2 [file pcbi.1014505.s001.docx]

**Supplementary Information**

**Figure A**. **Training Progression of Feed-Forward Neural Network (FFNN).** The root mean squared error (RMSE) loss over epochs during the training of a fully connected neural network with three hidden layers. The input dimension is 204 features, expanded to a hidden dimension of 304 neurons for the first hidden layer, followed by two hidden layers each maintaining this size (304 neurons). Each hidden layer is activated with a Rectified Linear Unit (ReLU) and normalized using Layer Normalization. The final output is a single neuron predicting tumor fraction. The progressive decrease in RMSE indicates the model’s convergence and learning efficacy.

**Figure B.** **Receiver Operating Characteristic** (**ROC) comparison of ALFAssay, Regression, Fragle and their composite (ALFAssay+Fragle) models on the validation dataset.** (A) ichorCNA detection (ctDNA detected defined as ichorCNA tumor fraction ≥ 0.03) as reference. Area Under the Curve (AUC) values are shown in the legend; the text box reports PPV and NPV at the indicated thresholds. For all models, thresholds were determined on the validation set by tracing the ROC of the predicted tumor fraction or score against ichorCNA-based ground-truth labels and selecting the cut-off that maximized Youden’s J. (B) OncoFollow detection (samples considered ctDNA-positive if any tested gene was mutated) as reference. AUCs are reported in the legend, and PPV/NPV values at the Youden’s J–optimal operating points are shown in the text box. (C) Progression-free survival (PFS) classification as reference (patients with PFS ≤ 6 months versus > 6 months). ROC curves compare ALFAssay, Regression, and Fragle performance as well as their composite (ALFAssay + Fragle). AUCs are given in the legend.

**Figure C**. **Correlations between ALFAssay tumor-fraction estimates and alternative ctDNA measures in the training dataset.** A–C (NeoRhea, early breast cancer): ALFAssay vs (A) ichorCNA tumor fraction, and (B) Fragle tumor fraction; cross-model correlations among alternatives: (C) –F (Synergy, metastatic TNBC): ALFAssay vs (G) ichorCNA and (H) Fragle; cross-model (I) Fragle vs ichorCNA. Points represent individual samples; color encodes ichorCNA detection (blue, ctDNA-; orange, ctDNA+ defined as ichorCNA tumor fraction >= 3%), and shape denotes cohort. Spearman correlation coefficients (R) and p-values are shown in each panel.

**Figure D**. **Kaplan–Meier estimates of progression-free survival (PFS) according to ctDNA levels and detection status at Baseline.** (A–C) PFS curves stratified by ctDNA levels derived from ALFAssay (A), ichorCNA (B), and Fragle (C). For ALFAssay, ctDNA levels were defined as *Undetected* (< 0.03), *Low* (0.03 – 0.2), and *High* (> 0.2). For ichorCNA, ctDNA levels were defined as *Undetected* (< 0.03), *Low* (0.03 – 0.2), and *High* (> 0.2). For Fragle, thresholds were chosen to match the number of patients per category in ALFAssay: *Undetected* (< 0.08), *Low* (0.08–0.37), and *High* (> 0.37). (D–F) PFS curves stratified by pairwise ctDNA detection based on Day 14 ctDNA positivity (ALFAssay was considered positive if tumor fraction was ≥ 0.015, ichorCNA was considered positive if tumor fraction was ≥ 0.03, Fragile was considered positive if tumor fraction was ≥ 0.08, OncoFollow positive if 1 tested gene is mutated) across two models: (D) ALFAssay + OncoFollow, (E) ALFAssay + Fragle, and (F) ALFAssay + ichorCNA. Curves show the proportion without progression over time (months) . Log-rank p-values indicate between-group differences.

**Figure E**. **Progression free survival** (**PFS) curves stratified by pairwise ctDNA detection based on Baseline and Day 14 ctDNA positivity**. (A-B) OncoFollow + Fragle, (C-D) ichorCNA + Fragle, and (E-F) ichorCNA + OncoFollow. ALFAssay was considered positive if tumor fraction was ≥ 0.03, ichorCNA was considered positive if tumor fraction was ≥ 0.03, Fragle was considered positive if tumor fraction was ≥ 0.89, OncoFollow positive if 1 tested gene is mutated. Curves show the proportion without progression over. Log-rank p-values indicate between-group differences.

**Figure F.** **Univariate Cox proportional-hazards models relating progression free survival(PFS) to binarized ctDNA biomarkers** at baseline (A) and Day 14 after treatment initiation (B). Forest plots show hazard ratios (HRs) with 95% confidence intervals (CIs); the dashed vertical line marks the null (HR = 1). Numeric CI limits and two- sided p-values are printed to the right. HR > 1 indicates increased risk of progression; HR < 1 indicates a protective association. Biomarkers include single-model outputs (ALFAssay, OncoFollow, ichorCNA, Fragle) and pairwise composites (a composite is positive if either model is positive; negative only if both are negative). Binarization thresholds: ichorCNA ctDNA-positive if tumor fraction≥ 0.03; Fragle ≥0.08 (for Fragle, thresholds were chosen to match the number of patients per category in ALFAssay); ALFAssay ≥0.03 (see Methods); OncoFollow positive if 1 tested gene is mutated.

# **CONSORT 2025 Checklist**

# **NeoRHEA Study**

This study is a prospective, open-label, single-arm phase II trial (NeoRHEA). CONSORT items related to randomisation and blinding are not applicable (N/A). Checklist completed according to the CONSORT 2025 Statement (Hopewell et al., BMJ 2025;388:e081123).

# **Synergy Study**

SYNERGY (NCT03616886) is a phase I/II trial testing if targeting the immunosuppressive adenosine pathway with the anti-CD73 antibody oleclumab, plus the anti-PD-L1 durvalumab and chemotherapy, enhances antitumor activity in untreated advanced triple-negative breast cancer. Checklist completed according to the CONSORT 2025 Statement (Hopewell et al., BMJ 2025;388:e081123).

**Pearl Study**

This is a prospective, single arm trial in which patients with locally advanced or metastatic endocrine receptor positive and HER2 negative breast cancer refractory to non-steroidal aromatase inhibitors (NSAI) will receive Everolimus 10mg orally daily given in conjunction with exemestane 25mg orally daily until disease progression or treatment discontinuation for any other reasons.

**Healthy Women**

98 healthy women were recruited and approved by the Institut Bordet Ethical committee (CE3369, 15/11/21)**.**

| Section / Topic | No | CONSORT item description | Reported on page / figure |
| --- | --- | --- | --- |
| Title and abstract | 1a | Identification as a clinical trial | Abstract (p.2) |
|  | 1b | Structured summary of trial design, methods, results, and conclusions | Methods (p.12) |
| Open science | 2 | Trial registration (registry name, ID, date) | Abstract (p.2); Methods (p.12) |
|  | 3 | Where the trial protocol and SAP can be accessed | Methods (p.12); Data Availability (p.20) |
|  | 4 | Data sharing statement | Data Availability (p.20) |
|  | 5a | Sources of funding and role of funders | Acknowledgements (p.20) |
|  | 5b | Financial and other conflicts of interest | Competing Interests (p.1) |
| Introduction | 6 | Scientific background and rationale | Introduction (p.2) |
|  | 7 | Specific objectives related to benefits and harms | Introduction (p.2) |
| Methods | 8 | Patient/public involvement | N/A – Patients not involved |
|  | 9 | Description of trial design (type, allocation, framework) | Methods (p.12) |
|  | 10 | Important protocol changes | N/A |
|  | 11 | Trial setting and locations | Methods (p.12) |
|  | 12a | Eligibility criteria for participants | Methods (p.12) |
|  | 12b | Eligibility for sites/personnel | N/A |
|  | 13 | Intervention details (allowing replication) | Methods (p.12) |
|  | 14 | Prespecified outcomes (primary & secondary) | Methods (p.12) |
|  | 15 | Definition and assessment of harms | N/A – no adverse events collected |
|  | 16a | Sample size determination | N/A |
|  | 16b | Interim analyses/stopping rules | N/A |
| Randomisation | 17a | Generation of random sequence | N/A |
|  | 17b | Type and restriction | N/A |
|  | 18 | Allocation concealment mechanism | N/A |
|  | 19 | Implementation | N/A |
| Blinding | 20a | Who was blinded | Open-label; N/A |
|  | 20b | How blinding was achieved | N/A |
| Statistical methods | 21a | Methods to compare groups | Methods (p.18) |
|  | 21b | Who is included in each analysis | Figure 1 |
|  | 21c | Handling missing data | N/A |
|  | 21d | Additional analyses (subgroups, sensitivity) | Methods (p.17-18) |
| Results | 22a | Participant flow (diagram) | Figure 1 |
|  | 22b | Losses/exclusions after enrolment | Figure 1 |
|  | 23a | Recruitment and follow-up dates | Methods (p.12) |
|  | 23b | Why trial ended or stopped | Completed as planned; N/A |
|  | 24a | Intervention actually administered (adherence, fidelity) | Methods (p.12) |
|  | 24b | Concomitant care | N/A |
|  | 25 | Baseline demographic/clinical characteristics | N/A |
|  | 26 | Numbers analysed and effect sizes | Methods (p.12); Tables S1, S2 |
|  | 27 | Harms or unintended events | N/A |
|  | 28 | Ancillary/subgroup analyses | Results p.5 |
| Discussion | 29 | Interpretation consistent with results | Discussion (p.9-12) |
|  | 30 | Limitations, bias, generalisability | Discussion (p.59-12) |
