# Supplementary material for: ALFAssay: A feed‑forward neural network for quantitative fragmentomics‑based ctDNA profiling in breast cancer
Source: PLoS Comput Biol. 2026 Jul 27;22(7):e1014505. doi: 10.1371/journal.pcbi.1014505 (PMC13450836; doi:10.1371/journal.pcbi.1014505)
Supplement: Supplementary file 3 [file pcbi.1014505.s009.docx]

**Reviewer #1**:

Summary of the paper

 The work presented by Stanciu and colleagues introduces ALFAssay, a neural network-based method to estimate the fraction of circulating tumor DNA from cell-free DNA, using fragmentation profiles from shallow whole-genome sequencing: it is specifically aimed to address the limitations of ctDNA estimation in low copy number tumors such as HR-/HER2+ breast cancer.

 The authors correctly report and describe other existing tools that make use of fragmentomics, such as illMAC and Fragle. The novelty of their approach lies in its breast cancer-specific training and the normalization pipeline applied to raw fragment profiles to remove coverage-dependent bias.

The results seem to support the validity of their approach in terms of ctDNA differences between healthy controls and breast cancers, concordance with other methods, and the ability to identify patients with worse prognoses. Additionally, they showed the complementary aspects of the work, highlighting the strong but incomplete correlation with other models and the pairwise combination of tools to explore progression-free survival differences.

 The authors recognize and address the limits of their work, specifically that the ground truth for training (ichorCNA) is not a gold standard and carries inherent uncertainty, in particular below the lower limit of detection (0.03), suggesting the direction for future studies using spike-in control methods to provide better validation.

The raw data is accessible by requests and the processed data is deposited on Zenodo.
The code is available on github, although it lacks a proper “packaging” and any guide on how to use it.

Recommendation by the reviewer

 Overall, the paper is well written and the results clearly presented and discussed.

Despite the limitations and the not completely novelty of ALFAssay (Fragle being the most similar), different approaches to detect and/or quantify ctDNA from liquid biopsies are worth being explored aiming to the integration of multiple methods with the purpose of reducing biases carried by single-method approaches. To achieve this, new bioinformatics approaches must be easy to use so that other researchers can readily test them.

**I recommend the publication of the paper, provided that the code is integrated with scripts to run the pipeline, possibly with testing examples, and a well documented guide.**

***Reply:***

*Thank you for this valuable suggestion. In response, we have added a comprehensive README file to the project’s GitHub repository that provides clear instructions on how to run the full pipeline, along with a practical example to facilitate reproducibility and ease of use.*

*The documentation is available here:*
[*https://github.com/mariaalexandrastanciu/ALFAssay/blob/main/README.md*](https://github.com/mariaalexandrastanciu/ALFAssay/blob/main/README.md)

*This addition is intended to ensure that users can readily execute the pipeline and understand its workflow.*

Minor issues/requests:

 - the authors could display the distribution of the fragment length in the four cohorts for a better characterization of the dataset: showing tumor subtype differences may be useful to the research community, in addition to the expected differences between healthy controls and tumor samples;

**Reply:**

*We thank the reviewer for this suggestion. We explored fragment length distribution plots across the four cohorts; however, the observed differences were very subtle and did not reveal clearly distinguishable patterns. In particular, even the differences between healthy controls and metastatic breast cancer samples were only marginally visible, while differences between tumor subtypes were even less pronounced. We therefore considered that these plots would provide limited additional biological or methodological insight in the context of the present study. Nevertheless, we agree that such information may still be of interest to the research community and we can include these plots as supplementary figures if the reviewer and editor consider this necessary.*

The figure shows the fragment size distributions for Pearl(metastatic breast cancer) and healthy women - even looking at this plot the difference is quite small, by looking at the difference between Neorhea(early breast cancer ) and healthy women the difference is just not noticeable:

- in the paragraph “Risk Stratification Analysis Based on ctDNA Fraction Prediction” the authors wrote “For ichorCNA samples with ctDNA levels < 0.03 were defined as Undetected, between 0.03 and 0.3 as Low and > 0.2 as High”: I assume 0.2 is a typo;

***Reply:***

*We thank the reviewer for pointing this out. This was indeed a typographical error. In addition, since ichorCNA is used as the ground truth in our study and its limit of detection is 0.03, we consistently applied this threshold to define the “Undetected” category in order to ensure comparability across analyses between ALFAssay and ichorCNA. The manuscript has been corrected accordingly.*

 - related to the previous point, the authors also report that for ALFAssay they used 0.015 and 0.2 as thresholds between Undetected, Low and High categories: the first one is attributable to the decision thresholds based on the training data, however the second one is not discussed anywhere; unless 0.2 is also a typo the authors should discuss their decision, and in any case, briefly mention the origin of the lower threshold (0.015).

**Reply:**

*We thank the reviewer for this comment. The lower threshold (0.015) corresponds to the decision cut-off derived on the training data using Youden’s J criterion and was retained to ensure consistency in comparisons with models for which thresholds can be defined in the same way.*

*For the survival analysis though, we also considered the ichorCNA limit of detection (0.03), which is used as the ground truth in this study, and applied the same threshold to ALFAssay to ensure comparability between methods.*

*The upper threshold (0.2) was not derived through a formal optimization procedure but was introduced as a cut-off to distinguish samples with clearly elevated ctDNA levels from those with lower burden. We have clarified this point in the manuscript to avoid ambiguity in the first paragraph of the Results section/* *Risk Stratification Analysis Based on ctDNA Fraction Prediction.*

**Reviewer #2:**

In this manuscript, the authors report on the development of ALFAssay, an computational approach to use fragmentation profiles to estimate circulation tumor DNA fraction from shallow whole genome sequencing of plasma from patients with breast cancer.

 Major Concern:
 In the introduction, the authors emphasize the difficulty of estimating ctDNA fraction in cancer types, such as breast cancer, that have low copy number alterations using existing CN-based tools such as ichorCNA. They propose that ALFAssay, by using fragmentomics, may be an improvement on this approach. However, the ALFAssay model used ichorCNA results as the reference for model training. At best, a model can only ever perform as well as the gold standard against which it was trained. Thus, it is inherently flawed to think that a new model will perform better than the gold standard against which it was trained. The data shown here is convincing that ALFAssay can replicate the results of ichorCNA, but not that it is an improvement. This failure to improve is clearly demonstrated in the early breast cancer cohort where no/very little ctDNA was detected by either ichorCNA or ALFAssay, even though it has been well demonstrated that ctDNA is present in early breast cancer patients using ultrasensitive orthogonal methods such as tissue-informed MRD (for example iSPY, PREDICT-DNA, etc). In order to claim that ALFAssay is an improvement on ichorCNA, it cannot be trained using ichorCNA as the reference - it needs to be trained on results from an orthogonal method**. If authors are unable to re-train the model using results from an orthogonal method, then they should reframe the development of ALFAssay as an alternative, but not an improvement, to ichorCNA. The authors also need to be mindful of circularity when comparing and/or combining ALFAssay with the reference it was trained upon (ichorCNA).**

***Reply***:

*We thank the reviewer for this important and thoughtful comment. We agree that training ALFAssay using ichorCNA-derived ctDNA estimates introduces important limitations regarding interpretation and benchmarking. In particular, without an orthogonal ground-truth measurement (e.g., tissue-informed MRD assays), we cannot conclusively demonstrate that ALFAssay outperforms ichorCNA, and comparisons between the two approaches must be interpreted with caution due to potential circularity.*

*We also acknowledge, however, that a model trained on an imperfect reference may still learn biologically meaningful signals that extend beyond the noise or limitations of the training labels, particularly when the input features capture complementary information. In our case, fragmentation patterns may contain ctDNA-related information that is only partially reflected by copy-number-based estimates. Nevertheless, because the present study does not include sufficiently large cohorts with independent orthogonal ctDNA measurements, we agree that claims of superiority over ichorCNA would not be adequately supported.*

*In light of this, we have revised the manuscript to more accurately position ALFAssay as an alternative fragmentation-based approach for ctDNA estimation, rather than as a demonstrated improvement over ichorCNA. We now emphasize its complementary nature and explicitly acknowledge the limitations associated with using ichorCNA-derived labels during training.*

*We have also clarified the potential for circularity when comparing or combining ALFAssay with ichorCNA and adjusted the language throughout the manuscript accordingly.*

*The following sections were revised to reflect these points:*

- *Introduction: final paragraph*
- *Results: “Risk Stratification Analysis Based on ctDNA Fraction Prediction”*
- *Discussion:*
  1. *Paragraph beginning “Several limitations of our study warrant discussion...”*
  2. *Paragraph beginning “ALFAssay demonstrates performance...”*

Minor:
 The text along the axis, legends, etc within the supplemental figures needs to be enlarged - it is very difficult to read.

**Reply:**

*We thank the reviewer for this comment. The text size of the axes, legends, and labels in the supplementary figures has been increased to improve readability.*

I don't think it is necessary to provide the equations for accuracy, sens, spec, PPV, and NPV - these are standard definitions.

 **Reply:**

*We thank the reviewer for this suggestion. We agree that these are standard metric definitions and have removed the corresponding equations from the Methods section to improve readability and streamline the manuscript.*

Even in the word document, several of the characters within the equations are not rendering correctly. Consider using an alternative program such as Latex to better display equations.

**Reply:**

*We thank the reviewer for pointing this out. We have revised the equations and corrected the rendering issues identified in the Word document. We hope that the formatting problems have now been resolved. However, if any equation rendering issues persist in the revised version, we would greatly appreciate the reviewer letting us know so that we can address them accordingly.*

**Reviewer #3:**

The manuscript describes ALFAssay, a new computational method based on a feed-forward neural network to estimate ctDNA fraction from cfDNA fragmentation features derived from low-pass WGS, that was applied to multiple breast cancer cohorts. The topic is timely and the datasets are relevant; however, there are some methodological aspects that may limit generalizability and the potential added value over existing approaches. My main comments are below.

 1) The preprocessing step normalizes bin-level fragmentation ratios via LOESS as a function of sequencing depth “across samples”. In Figure 2, it appears that LOESS models are fitted within each dataset. This would imply dataset-specific normalization and may limit real-world implementation. More generally, a normalization that requires a cohort to fit depth effects is not directly applicable in prospective settings where samples are processed one-by-one. What happens if the fit is derived from the training set and the fitted function is applied to the validation cohort without refitting? Please discuss this limitation and/or provide an alternative strategy (e.g., a fixed reference fitted on controls/ctDNA-negative samples).

**Reply:**

*We thank the reviewer for this important comment. We agree that the original LOESS-based normalization strategy constitutes a limitation, as it relied on fitting depth-dependent effects across samples within each dataset, making the preprocessing cohort-dependent and less suitable for prospective real-world applications where samples are processed individually.*

*In response to this concern, we revised the preprocessing strategy and removed the LOESS normalization step entirely. Instead, we now provide the total fragment count for each genomic bin directly as an additional input feature to the neural network alongside the short-to-total fragmentation ratios. As a result, the model input dimensionality increased from 204 to 408 features, with each fragmentation ratio paired with its corresponding bin-level coverage value.*

*This modification eliminates the need for cohort-level normalization or refitting procedures during preprocessing, while allowing the neural network to learn and account for coverage-related biases directly during training. We believe this updated approach improves the generalizability and practical applicability of the model in prospective settings and simplifies the overall preprocessing pipeline by removing a dataset-dependent normalization step.*

2) In shallow WGS, depth and coverage variability can reflect technical effects but also underlying CNV levels. It is unclear how LOESS normalization operates on biologically meaningful signal, especially in samples with higher CNA burden. Are the ~200 informative bins overlapping with CN-informative regions? Please provide analyses to support that the normalization step is robust to CNA effects, including what happens when restricting to regions not involved in CNA (e.g., segments with CN=2 as provided by ichorCNA). This would support complementarity with CNA-based tools.

**Reply:**

*We thank the reviewer for this important observation. We agree that, in shallow WGS data, sequencing depth and coverage variability may reflect not only technical effects but also biologically meaningful copy-number alterations, particularly in samples with higher CNA burden. As a result, applying LOESS normalization across samples could potentially attenuate or distort biologically relevant signals.*

*In response to this concern, we revised the preprocessing strategy and retrained the model without the LOESS normalization step. Instead, the total fragment count for each genomic bin was included directly as an additional input feature to the neural network together with the short-to-total fragmentation ratios. This approach allows the model to learn and account for coverage-related effects during training, rather than explicitly correcting them through cohort-level preprocessing.*

*By removing the LOESS normalization step, we aimed to reduce the risk of inadvertently modifying biologically relevant CNA-associated signals while simplifying the preprocessing pipeline. The updated framework therefore relies on the neural network to jointly model fragmentation and coverage information directly from the input features.*

3) The comparison to a single median short-fragment ratio regression baseline is useful but not sufficient. Given the feature vector comprises ~200 bins, a regularized linear model (ridge/lasso/elastic net) represents a natural and strong baseline. Please add this baseline to demonstrate that the non-linear FFNN provides an improved performance.

**Reply**:

*We thank the reviewer for this suggestion. In response, we implemented and evaluated a regularized LASSO regression model as an additional baseline comparison. We selected LASSO because it is well suited for high-dimensional input spaces and sparse feature selection, which better matches our feature representation than the previously used median short-fragment ratio approach.*

*This became particularly relevant after revising the preprocessing pipeline and removing the LOESS normalization step. In the updated framework, each genomic bin contributes two input features—the short-to-total fragmentation ratio and the corresponding total fragment count—resulting in a 408-dimensional feature space. Under this representation, reducing the data to a single median fragmentation ratio would no longer appropriately capture the structure of the input data.*

*The LASSO model was therefore trained using the same feature space as ALFAssay, allowing a more appropriate comparison between a regularized linear model and the non-linear feed-forward neural network architecture. The corresponding analyses and results have now been added to the manuscript.*

4) The key value proposition is improved ctDNA quantification when CNA-based / other fragmentation tools underperform (e.g., low-CNA tumors). Please discuss performance and/or clinical stratification in the subset of samples where ichorCNA is undetected/low (or otherwise challenging), and where ALFAssay should provide the clearest benefit.

**Reply:**

*We thank the reviewer for highlighting this important point. To address it, we performed an exploratory analysis of PEARL samples with undetected ichorCNA signal, defined as ichorTF <= 0.03. Among 58 such samples, ALFAssay was elevated (PredictedValue >= 0.03) in 3 samples. Two of these 3 samples also had detectable mutation by OncoFOLLOW, and all 3 had high tumor fraction calculated by Fragle, suggesting that ALFAssay may detect ctDNA-related fragmentation signal in a small subset of samples where CNA-based tumor fraction is low. However, given the very small number of ALFAssay-high/ichor-low cases, formal clinical stratification or survival analysis would be underpowered and potentially misleading. We have therefore added this as a descriptive analysis and clarified in the Discussion that larger cohorts, ideally enriched for low-CNA tumors, are needed to assess the clinical value of ALFAssay in this setting.*

5) The manuscript reports complementarity between fragmentation-based tools and mutation/CNA-based assays. This would be strengthened by a focused analysis of discordant samples (e.g., ALFAssay high but mutation VAF low, or vice versa) to assess whether discordance is associated with clinical outcome, sample quality, or biological features.

***Reply:***

*We thank the reviewer for this valuable suggestion. To explore the complementarity and potential discordance between fragmentation-based and CNA/mutation-based approaches, we performed an exploratory analysis of PEARL samples with undetected ichorCNA signal (ichorTF <= 0.03). Among 58 such samples, ALFAssay was elevated (PredictedValue >= 0.03) in 3 cases. Notably, 2 of these 3 samples also had detectable mutations identified by OncoFOLLOW, and all 3 showed elevated tumor fraction estimates by Fragle, suggesting that ALFAssay may capture ctDNA-related fragmentation signal in a subset of samples with limited CNA signal.*

*We additionally considered whether such discordance could reflect technical or sample-quality effects. However, the fragmentomics pipeline incorporated stringent quality-control and filtering procedures, including restriction to properly paired reads, exclusion of low-confidence genomic regions, filtering on mappability (>70%), removal of regions overlapping ENCODE blacklist regions, and selection of bins recommended for downstream analysis using the QDNAseq framework. These steps were intended to minimize technical variability and ensure that fragmentation features were derived from high-confidence genomic regions.*

*Nevertheless, the number of discordant ALFAssay-high/ichor-low samples was very small, limiting the ability to perform meaningful statistical association analyses with clinical outcome or biological subgroups without substantial risk of overinterpretation. We therefore present these findings as descriptive observations and have clarified this point in the Discussion. Larger cohorts, particularly those enriched for low-CNA tumors and accompanied by orthogonal ctDNA measurements, will be required to determine whether such discordant cases reflect biologically relevant ctDNA signal beyond CNA-based detection.*
