# Supplementary material for: ALFAssay: A feed‑forward neural network for quantitative fragmentomics‑based ctDNA profiling in breast cancer
Source: PLoS Comput Biol. 2026 Jul 27;22(7):e1014505. doi: 10.1371/journal.pcbi.1014505 (PMC13450836; doi:10.1371/journal.pcbi.1014505)
Supplement: Peer review history

# Peer review: Decision Letter 0

**DOI:** 10.1371/journal.pcbi.1014505.r001

**Authors:** Can Yang, Ruslan Kalendar

18 Mar 2026

PCOMPBIOL-D-26-00112

ALFAssay: a feed-forward neural network for quantitative fragmentomics-based ctDNA profiling in breast cancer

PLOS Computational Biology

Dear Dr. Stanciu,

Thank you for submitting your manuscript to PLOS Computational Biology. After careful consideration, we feel that it has merit but does not fully meet PLOS Computational Biology's publication criteria as it currently stands. Therefore, we invite you to submit a revised version of the manuscript that addresses the points raised during the review process.

Please submit your revised manuscript by May 18 2026 11:59PM. If you will need more time than this to complete your revisions, please reply to this message or contact the journal office at ploscompbiol@plos.org. When you're ready to submit your revision, log on to https://www.editorialmanager.com/pcompbiol/ and select the 'Submissions Needing Revision' folder to locate your manuscript file.

Please include the following items when submitting your revised manuscript:

* A letter that responds to each point raised by the editor and reviewer(s). You should upload this letter as a separate file labeled 'Response to Reviewers'. This file does not need to include responses to formatting updates and technical items listed in the 'Journal Requirements' section below.

* A marked-up copy of your manuscript that highlights changes made to the original version. You should upload this as a separate file labeled 'Revised Manuscript with Track Changes'.

* An unmarked version of your revised paper without tracked changes. You should upload this as a separate file labeled 'Manuscript'.

If you would like to make changes to your financial disclosure, competing interests statement, or data availability statement, please make these updates within the submission form at the time of resubmission. Guidelines for resubmitting your figure files are available below the reviewer comments at the end of this letter

We look forward to receiving your revised manuscript.

Kind regards,

Ruslan Kalendar, PhD

Academic Editor

PLOS Computational Biology

Can Yang

Section Editor

PLOS Computational Biology

**Additional Editor Comments :**

The reviewers’ comments are set out below. Authors are requested to take these comments into account when revising their manuscript accordingly.

**Journal Requirements:**

1) Please ensure that the CRediT author contributions listed for every co-author are completed accurately and in full.

At this stage, the following Authors/Authors require contributions: Alexandra Stanciu, Andrea Gombos, Elisa Agostinetto, Delphine Vincent, Laurence Buisseret, Andreas Papagiannis, Francoise Rothe, Nicola Occelli, Christos Sotiriou, David Venet, and Michail Ignatiadis. Please ensure that the full contributions of each author are acknowledged in the "Add/Edit/Remove Authors" section of our submission form.

The list of CRediT author contributions may be found here: https://journals.plos.org/ploscompbiol/s/authorship#loc-author-contributions

2) Thank you for stating that "The trials were approved by the institutional review boards and ethical committees of each participating center."  Please include:

i) The full name(s) of the Institutional Review Board(s) or Ethics Committee(s).

3) Please upload all main figures as separate Figure files in .tif or .eps format. For more information about how to convert and format your figure files please see our guidelines:

https://journals.plos.org/ploscompbiol/s/figures

4) Please ensure that all Figure files have corresponding citations and legends within the manuscript. Currently, Figure 1 in your submission file inventory does not have an in-text citation. If the figure is no longer to be included as part of the submission, please remove it from the file inventory.

5) We have noticed that you have uploaded Supporting Information files, but you have not included a list of legends. Please add a full list of legends for your Supporting Information files after the references list.

6) Some material included in your submission may be copyrighted. According to PLOSu2019s copyright policy, authors who use figures or other material (e.g., graphics, clipart, maps) from another author or copyright holder must demonstrate or obtain permission to publish this material under the Creative Commons Attribution 4.0 International (CC BY 4.0) License used by PLOS journals. Please closely review the details of PLOSu2019s copyright requirements here: PLOS Licenses and Copyright. If you need to request permissions from a copyright holder, you may use PLOS's Copyright Content Permission form.

Please respond directly to this email and provide any known details concerning your material's license terms and permissions required for reuse, even if you have not yet obtained copyright permissions or are unsure of your material's copyright compatibility. Once you have responded and addressed all other outstanding technical requirements, you may resubmit your manuscript within Editorial Manager.

Potential Copyright Issues:

i) Figure 2. Please confirm whether you drew the images / clip-art within the figure panels by hand. If you did not draw the images, please provide (a) a link to the source of the images or icons and their license / terms of use; or (b) written permission from the copyright holder to publish the images or icons under our CC BY 4.0 license. Alternatively, you may replace the images with open source alternatives. See these open source resources you may use to replace images / clip-art:

- https://commons.wikimedia.org

- https://openclipart.org/.

ii) The following Figures contain a logo or branding: Supplementary files (3369 2021 11 04-1.pdf, CE-PEARL.pdf, PEARL-Protocol-version 3.0 clean.pdf, , Protocole-pourCE-corrected.pdf, SYNERGY_protocol_v9.0.pdf, neorhea.pdf, 2016-000879-24-Opinion_Final.pdf, and 2016-000879-24-Approval-letter.pdf). We are not permitted to publish this under our CC-BY 4.0 license, even with permission. We ask that you please remove or replace it.

7) In the online submission form you indicate that your data is not available for proprietary reasons and have provided a contact point for accessing this data. Please note that your current contact point is a co-author on this manuscript. According to our Data Policy, the contact point must not be an author on the manuscript and must be an institutional contact, ideally not an individual. Please revise your data statement to a non-author institutional point of contact, such as a data access or ethics committee, and send this to us via return email. Please also include contact information for the third party organization, and please include the full citation of where the data can be found.

8) Your current Financial Disclosure states, "The author(s) received no specific funding for this work.".

However, your funding information on the submission form indicates receiving funds.. Please ensure that the funders and grant numbers match between the Financial Disclosure field and the Funding Information tab in your submission form. Note that the funders must be provided in the same order in both places as well.

Please amend your detailed Financial Disclosure statement. This is published with the article. It must therefore be completed in full sentences and contain the exact wording you wish to be published.

1) Please clarify all sources of financial support for your study. List the grants, grant numbers, and organizations that funded your study, including funding received from your institution. Please note that suppliers of material support, including research materials, should be recognized in the Acknowledgements section rather than in the Financial Disclosure

2) State the initials, alongside each funding source, of each author to receive each grant. For example: "This work was supported by the National Institutes of Health (####### to AM; ###### to CJ) and the National Science Foundation (###### to AM)."

3) State what role the funders took in the study. If the funders had no role in your study, please state: "The funders had no role in study design, data collection and analysis, decision to publish, or preparation of the manuscript."

4) If any authors received a salary from any of your funders, please state which authors and which funders..

Note: If the reviewer comments include a recommendation to cite specific previously published works, please review and evaluate these publications to determine whether they are relevant and should be cited. There is no requirement to cite these works unless the editor has indicated otherwise.

**Reviewers' comments:**

Reviewer's Responses to Questions

Reviewer #1: Summary of the paper

The work presented by Stanciu and colleagues introduces ALFAssay, a neural network-based method to estimate the fraction of circulating tumor DNA from cell-free DNA, using fragmentation profiles from shallow whole-genome sequencing: it is specifically aimed to address the limitations of ctDNA estimation in low copy number tumors such as HR-/HER2+ breast cancer.

The authors correctly report and describe other existing tools that make use of fragmentomics, such as illMAC and Fragle. The novelty of their approach lies in its breast cancer-specific training and the normalization pipeline applied to raw fragment profiles to remove coverage-dependent bias.

The results seem to support the validity of their approach in terms of ctDNA differences between healthy controls and breast cancers, concordance with other methods, and the ability to identify patients with worse prognoses. Additionally, they showed the complementary aspects of the work, highlighting the strong but incomplete correlation with other models and the pairwise combination of tools to explore progression free survival differences.

The authors recognize and address the limits of their work, specifically that the ground truth for training (ichorCNA) is not a gold standard and carries inherent uncertainty, in particular below the lower limit of detection (0.03), suggesting the direction for future studies using spike-in control methods to provide better validation.

The raw data is accessible by requests and the processed data is deposited on Zenodo.

The code is available on github, although it lacks a proper “packaging” and any guide on how to use it.

Recommendation by the reviewer

Overall, the paper is well written and the results clearly presented and discussed.

Despite the limitations and the not completely novelty of ALFAssay (Fragle being the most similar), different approaches to detect and/or quantify ctDNA from liquid biopsies are worth being explored aiming to the integration of multiple methods with the purpose of reducing biases carried by single-method approaches. To achieve this, new bioinformatics approaches must be easy to use so that other researchers can readily test them.

I recommend the publication of the paper, provided that the code is integrated with scripts to run the pipeline, possibly with testing examples, and a well documented guide.

Minor issues/requests:

- the authors could display the distribution of the fragment length in the four cohorts for a better characterization of the dataset: showing tumor subtype differences may be useful to the research community, in addition to the expected differences between healthy controls and tumor samples;

- in the paragraph “Risk Stratification Analysis Based on ctDNA Fraction Prediction” the authors wrote “For ichorCNA samples with ctDNA levels < 0.03 were defined as Undetected, between 0.03 and 0.3 as Low and > 0.2 as High”: I assume 0.2 is a typo;

- related to the previous point, the authors also report that for ALFAssay they used 0.015 and 0.2 as thresholds between Undected, Low and High categories: the first one is attributable to the decision thresholds based on the training data, however the second one is not discussed anywhere; unless 0.2 is also a typo the authors should discuss their decision, and in any case, briefly mention the origin of the lower threshold (0.015).

Reviewer #2: In this manuscript, the authors report on the development of ALFAssay, an computational approach to use fragmentation profiles to estimate circulation tumor DNA fraction from shallow whole genome sequencing of plasma from patients with breast cancer.

Major Concern:

In the introduction, the authors emphasize the difficulty of estimating ctDNA fraction in cancer types, such as breast cancer, that have low copy number alterations using existing CN-based tools such as ichorCNA. They propose that ALFAssay, by using fragmentomics, may be an improvement on this approach. However, the ALFAssay model used ichorCNA results as the reference for model training. At best, a model can only ever perform as well as the gold standard against which it was trained. Thus, it is inherently flawed to think that a new model will perform better than the gold standard against which it was trained. The data shown here is convincing that ALFAssay can replicate the results of ichorCNA, but not that it is an improvement. This failure to improve is clearly demonstrated in the early breast cancer cohort where no/very little ctDNA was detected by either ichorCNA or ALFAssay, even though it has been well demonstrated that ctDNA is present in early breast cancer patients using ultrasensitive orthogonal methods such as tissue-informed MRD (for example iSPY, PREDICT-DNA, etc). In order to claim that ALFAssay is an improvement on ichorCNA, it cannot be trained using ichorCNA as the reference - it needs to be trained on results from an orthogonal method. If authors are unable to re-train the model using results from an orthogonal method, then they should reframe the development of ALFAssay as an alternative, but not an improvement, to ichorCNA. The authors also need to be mindful of circularity when comparing and/or combining ALFAssay with the reference it was trained upon (ichorCNA).

Minor:

The text along the axis, legends, etc within the supplemental figures needs to be enlarged - it is very difficult to read.

I don't think it is necessary to provide the equations for accuracy, sens, spec, PPV, and NPV - these are standard definitions.

Even in the word document, several of the characters within the equations are not rendering correctly. Consider using an alternative program such as Latex to better display equations.

Reviewer #3: The manuscript describes ALFAssay, a new computational method based on a feed-forward neural network to estimate ctDNA fraction from cfDNA fragmentation features derived from low-pass WGS, that was applied to multiple breast cancer cohorts. The topic is timely and the datasets are relevant; however, there are some methodological aspects that may limit generalizability and the potential added value over existing approaches. My main comments are below.

1) The preprocessing step normalizes bin-level fragmentation ratios via LOESS as a function of sequencing depth “across samples”. In Figure 2, it appears that LOESS models are fitted within each dataset. This would imply dataset-specific normalization and may limit real-world implementation. More generally, a normalization that requires a cohort to fit depth effects is not directly applicable in prospective settings where samples are processed one-by-one. What happens if the fit is derived from the training set and the fitted function is applied to the validation cohort without refitting? Please discuss this limitation and/or provide an alternative strategy (e.g., a fixed reference fitted on controls/ctDNA-negative samples).

2) In shallow WGS, depth and coverage variability can reflect technical effects but also underlying CNV levels. It is unclear how LOESS normalization operates on biologically meaningful signal, especially in samples with higher CNA burden. Are the ~200 informative bins overlapping with CN-informative regions? Please provide analyses to support that the normalization step is robust to CNA effects, including what happens when restricting to regions not involved in CNA (e.g., segments with CN=2 as provided by ichorCNA). This would support complementarity with CNA-based tools.

3) The comparison to a single median short-fragment ratio regression baseline is useful but not sufficient. Given the feature vector comprises ~200 bins, a regularized linear model (ridge/lasso/elastic net) represents a natural and strong baseline. Please add this baseline to demonstrate that the non-linear FFNN provides a improved performance.

4) The key value proposition is improved ctDNA quantification when CNA-based / other fragmention tools underperform (e.g., low-CNA tumors). Please discuss performance and/or clinical stratification in the subset of samples where ichorCNA is undetected/low (or otherwise challenging), and where ALFAssay should provide the clearest benefit.

5) The manuscript reports complementarity between fragmentation-based tools and mutation/CNA-based assays. This would be strengthened by a focused analysis of discordant samples (e.g., ALFAssay high but mutation VAF low, or vice versa) to assess whether discordance is associated with clinical outcome, sample quality, or biological features.

**********

**Have the authors made all data and (if applicable) computational code underlying the findings in their manuscript fully available?**

The PLOS Data policy requires authors to make all data and code underlying the findings described in their manuscript fully available without restriction, with rare exception (please refer to the Data Availability Statement in the manuscript PDF file). The data and code should be provided as part of the manuscript or its supporting information, or deposited to a public repository. For example, in addition to summary statistics, the data points behind means, medians and variance measures should be available. If there are restrictions on publicly sharing data or code —e.g. participant privacy or use of data from a third party—those must be specified.

Reviewer #1: Yes

Reviewer #2: None

Reviewer #3: **No:**  Data of shallow whole genome sequencing is not publicly available, and are accessible under specific conditions that are reported in Data Availability section.

**********

PLOS authors have the option to publish the peer review history of their article (what does this mean?). If published, this will include your full peer review and any attached files.

If you choose “no”, your identity will remain anonymous but your review may still be made public.

**Do you want your identity to be public for this peer review?**  For information about this choice, including consent withdrawal, please see our Privacy Policy.

Reviewer #1: No

Reviewer #2: No

Reviewer #3: No

[NOTE: If reviewer comments were submitted as an attachment file, they will be attached to this email and accessible via the submission site. Please log into your account, locate the manuscript record, and check for the action link "View Attachments". If this link does not appear, there are no attachment files.]

**Figure resubmission:**

While revising your submission, we strongly recommend that you use PLOS’s NAAS tool (https://ngplosjournals.pagemajik.ai/artanalysis) to test your figure files. NAAS can convert your figure files to the TIFF file type and meet basic requirements (such as print size, resolution), or provide you with a report on issues that do not meet our requirements and that NAAS cannot fix.

After uploading your figures to PLOS’s NAAS tool - https://ngplosjournals.pagemajik.ai/artanalysis, NAAS will process the files provided and display the results in the "Uploaded Files" section of the page as the processing is complete. If the uploaded figures meet our requirements (or NAAS is able to fix the files to meet our requirements), the figure will be marked as "fixed" above. If NAAS is unable to fix the files, a red "failed" label will appear above. When NAAS has confirmed that the figure files meet our requirements, please download the file via the download option, and include these NAAS processed figure files when submitting your revised manuscript.

**Reproducibility:**

To enhance the reproducibility of your results, we recommend that authors of applicable studies deposit laboratory protocols in protocols.io, where a protocol can be assigned its own identifier (DOI) such that it can be cited independently in the future. Additionally, PLOS ONE offers an option to publish peer-reviewed clinical study protocols. Read more information on sharing protocols at https://plos.org/protocols?utm_medium=editorial-email&utm_source=authorletters&utm_campaign=protocols

# Author comment: Author response to Decision Letter 1

**DOI:** 10.1371/journal.pcbi.1014505.r002

10 Jun 2026

Attachment Submitted filename: Response to Reviewers.docx

# Peer review: Decision Letter 1

**DOI:** 10.1371/journal.pcbi.1014505.r003

**Authors:** Can Yang, Ruslan Kalendar

28 Jun 2026

Dear Mrs Stanciu,

We are pleased to inform you that your manuscript 'ALFAssay: a feed-forward neural network for quantitative fragmentomics-based ctDNA profiling in breast cancer' has been provisionally accepted for publication in PLOS Computational Biology.

Before your manuscript can be formally accepted you will need to complete some formatting changes, which you will receive in a follow up email. A member of our team will be in touch with a set of requests.

Please note that your manuscript will not be scheduled for publication until you have made the required changes, so a swift response is appreciated.

IMPORTANT: The editorial review process is now complete. PLOS will only permit corrections to spelling, formatting or significant scientific errors from this point onwards. Requests for major changes, or any which affect the scientific understanding of your work, will cause delays to the publication date of your manuscript.

Should you, your institution's press office or the journal office choose to press release your paper, you will automatically be opted out of early publication. We ask that you notify us now if you or your institution is planning to press release the article. All press must be co-ordinated with PLOS.

Thank you again for supporting Open Access publishing; we are looking forward to publishing your work in PLOS Computational Biology.

Best regards,

Ruslan Kalendar, PhD

Academic Editor

PLOS Computational Biology

Can Yang

Section Editor

PLOS Computational Biology

***********************************************************

Reviewer's Responses to Questions

**Comments to the Authors:**

**Please note here if the review is uploaded as an attachment.**

Reviewer #1: I thank the authors for reporting the distribution of the fragment size, I leave to them to decide whether to add the figures to the supplemental.

Concerning the github repository, I would have preferred to see a functional testing example, consider to add it in the future to promote the usage of your tool. Also, naming all commits "initial commit" makes extremely difficult to follow the evolution of the tool.

Reviewer #2: The authors have nicely addressed my previous comments. Current manuscript is much stronger. My only remining concern is that the equations are still not rendering correctly in some parts of the pdf. On page 29 of the combined pdf, under the section Methods, Tumor Fraction Prediction Model, Model Architecture, none of the equations in this section are rendering correctly.

Reviewer #3: The authors have addressed all of my comments.

**********

**Have the authors made all data and (if applicable) computational code underlying the findings in their manuscript fully available?**

The PLOS Data policy requires authors to make all data and code underlying the findings described in their manuscript fully available without restriction, with rare exception (please refer to the Data Availability Statement in the manuscript PDF file). The data and code should be provided as part of the manuscript or its supporting information, or deposited to a public repository. For example, in addition to summary statistics, the data points behind means, medians and variance measures should be available. If there are restrictions on publicly sharing data or code —e.g. participant privacy or use of data from a third party—those must be specified.

Reviewer #1: **No:**  Raw data are not publicly available, but accessible upon request.

Reviewer #2: Yes

Reviewer #3: **No:**  Raw data is available upon request as reported in the data availability section.

**********

PLOS authors have the option to publish the peer review history of their article (what does this mean?). If published, this will include your full peer review and any attached files.

If you choose “no”, your identity will remain anonymous but your review may still be made public.

**Do you want your identity to be public for this peer review?**  For information about this choice, including consent withdrawal, please see our Privacy Policy.

Reviewer #1: No

Reviewer #2: No

Reviewer #3: No

# Editor report: Acceptance letter

**DOI:** 10.1371/journal.pcbi.1014505.r004

**Authors:** Can Yang, Ruslan Kalendar

PCOMPBIOL-D-26-00112R1

ALFAssay: a feed-forward neural network for quantitative fragmentomics-based ctDNA profiling in breast cancer

Dear Dr Stanciu,

I am pleased to inform you that your manuscript has been formally accepted for publication in PLOS Computational Biology. Your manuscript is now with our production department and you will be notified of the publication date in due course.

The corresponding author will soon be receiving a typeset proof for review, to ensure errors have not been introduced during production. Please review the PDF proof of your manuscript carefully, as this is the last chance to correct any errors. Please note that major changes, or those which affect the scientific understanding of the work, will likely cause delays to the publication date of your manuscript.

Soon after your final files are uploaded, unless you have opted out, the early version of your manuscript will be published online. The date of the early version will be your article's publication date. The final article will be published to the same URL, and all versions of the paper will be accessible to readers.

For Research, Software, and Methods articles, you will receive an invoice from PLOS for your publication fee after your manuscript has reached the completed accept phase. If you receive an email requesting payment before acceptance or for any other service, this may be a phishing scheme. Learn how to identify phishing emails and protect your accounts at https://explore.plos.org/phishing.

Thank you again for supporting PLOS Computational Biology and open-access publishing. We are looking forward to publishing your work!

With kind regards,

Sharmila Kamatchi

PLOS Computational Biology | Carlyle House, Carlyle Road, Cambridge CB4 3DN | United Kingdom ploscompbiol@plos.org | Phone +44 (0) 1223-442824 | ploscompbiol.org | @PLOSCompBiol
